# Robust Diagnostic and Therapeutic Biomarkers for Tuberculosis Identified Through Multi‐Omics and Mendelian Randomization Analysis

**DOI:** 10.1002/mco2.70559

**Published:** 2025-12-29

**Authors:** Chenglin Zhu, Jiaxi Chen, Ying Li, Qi Zhang, Qiqi Lu, Ningxuan Zhang, Hao Fan, Muhammad Mahtab Aslam Khan Khakwani, Lei Zhang, Ji‐Cheng Li

**Affiliations:** ^1^ School of Basic Medical Sciences Henan University Kaifeng Henan People's Republic of China; ^2^ Clinical Laboratory Taizhou Hospital of Zhejiang Province Affiliated to Wenzhou Medical University Linhai Zhejiang Province People's Republic of China; ^3^ Department of Clinical Laboratory The Second Affiliated Hospital, Zhejiang University School of Medicine Hangzhou People's Republic of China

**Keywords:** biological markers, lipidomics, mendelian randomization, plasma proteomics, tuberculosis

## Abstract

Tuberculosis (TB) remains a major global health challenge. In this study, we applied UPLC‐MS/MS lipidomics and data‐independent acquisition proteomics to profile plasma from healthy controls, active TB patients, and cured individuals to identify differentially expressed lipids and proteins. Mendelian randomization prioritized phosphatidylcholine (PC) lipids (PC(18:2/18:2), PC(14:0/20:4) and PC(18:0/20:4)) and proteins (haptoglobin [HP], retinol binding protein 4 [RBP4], coagulation factor XIII B subunit [F13B] and inter‐alpha‐trypsin inhibitor heavy chain 1 [ITIH1]) as candidate diagnostic and cure biomarkers. Binary multi‐omics random‐forest classifiers constructed with these markers achieved strong diagnostic (AUC = 0.967, 95% CI: 0.928–1.000) and cure‐monitoring (AUC = 0.981, 95% CI: 0.956–1.000) performance, which was further assessed with ten‐fold cross‐validation. Integration with transcriptomic data and lipid‐related gene analysis provided additional molecular support for HP. Independent validation in the GSE34608 cohort (AUC = 0.965) and ELISA verification (AUC = 0.969) confirmed HP's diagnostic utility at gene and protein levels. GSVA enrichment implicated HP in iron homeostasis and immune response pathways, suggesting a role in *Mycobacterium tuberculosis* infection and immune evasion through modulation of host iron metabolism. Overall, we present a robust lipid–protein biomarker panel and accurate multi‐omics models for TB diagnosis and monitoring of cure, and propose HP as a promising biomarker and potential therapeutic target. These tools may improve clinical management and treatment evaluation.

## Graphical abstract

We performed UPLC‐MS/MS lipidomics and DIA proteomics on plasma from healthy controls, active TB patients, and cured patients, and used Mendelian randomization to prioritize diagnostic and cure biomarkers. Multi‐omics random‐forest models based on these biomarkers showed excellent performance for diagnosis and cure monitoring. The key biomarker identified through multi‐dimensional screening, haptoglobin (HP), was independently validated in the GSE34608 cohort and by ELISA.

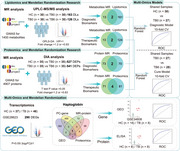

## 1 Introduction

Pulmonary tuberculosis (TB), caused by *Mycobacterium tuberculosis* (Mtb), is a chronic infectious disease [1]. According to a report from the World Health Organization (WHO) in 2024, it is estimated that in 2023, there were 10.8 million new cases of TB globally, with an incidence rate of 134 per 100,000 population [2]. During the same period, the global death toll from TB in 2023 reached 1.25 million, making TB the leading infectious disease cause of death worldwide, nearly twice the number of deaths attributed to HIV/AIDS.

Currently, the diagnosis of pulmonary TB primarily relies on sputum samples, but these present two major challenges: Difficulty in sample collection and poor sensitivity [3]. The WHO report indicates that bacteriologically confirmed TB cases account for only 51% of the total TB cases globally [4]. In addition to sample collection challenges, clinical tests such as smear microscopy, *M. tuberculosis* culture, and chest x‐rays also have limitations, as *M. tuberculosis* grows slowly and is an intracellular pathogen, often resulting in false‐negative results and diagnostic delays [5]. In addition, many diagnostic tests still require specialized equipment and laboratory support, which limits their applicability for large‐scale screening in resource‐constrained settings. Therefore, the development of sensitive and specific biomarkers based on blood, urine, or other non‐sputum specimens that can be implemented at the primary care level is of great significance for the detection of pulmonary TB, expanding screening coverage, and achieving point‐of‐care diagnosis.

Despite improvements in treatment, TB remains difficult to cure, particularly due to significant gaps in assessing treatment efficacy and confirming cure status [6]. TB treatment typically requires at least six months of multidrug therapy. The prolonged duration of treatment and the adverse effects associated with it are contributing to the emergence of multidrug‐resistant TB (MDR‐TB) [7], posing a significant burden on patients and health systems in many regions. Global data on TB treatment outcomes indicate that the success rate of the standard 6‐month regimen for newly diagnosed TB is only 85% [4]. The global recurrence rate of TB ranges from 2.3% to 6.5% [8, 9, 10]. At present, clinical assessment of treatment efficacy and “cure” status mainly relies on nonspecific indicators such as radiological improvement, symptom relief, and completion of therapy. However, an objective and quantifiable laboratory evaluation system is lacking. Therefore, there is an urgent need to identify biomarkers that can accurately evaluate the therapeutic efficacy of TB [11].

Blood, as a source of biomarkers, offers several advantages such as ease of sample collection, low invasiveness, and simple handling, making it highly promising for early disease screening and personalized treatment [12]. Lipids serve as the primary nutritional source for the growth and replication of Mtb, and the host's lipid profile is closely related to the pathogenesis of TB [13, 14]. Lipidomic studies examining changes in blood lipids in TB patients hold significant value. Proteomics can reveal differentially expressed proteins (DEPs) in the context of pulmonary TB [15], providing support for elucidating the molecular pathological mechanisms of TB. Transcriptomics, in turn, contributes molecular evidence from the gene expression level [16]. Integrated multi‐omics analysis allows for cross‐validation, providing valuable insights into biological functions that may not be revealed through single datasets [17]. Recent reviews on TB biomarkers have also emphasized the potential of multi‐omics approaches to enhance biomarker performance [12]. In addition, Mendelian randomization (MR) analysis aids in screening large numbers of candidate molecules to identify those most likely to have causal effects, prioritizing them for future functional validation, target development, and clinical translation. This approach helps reduce false positives due to confounding or reverse causality, thereby enhancing the credibility of research conclusions and their clinical applicability [18, 19, 20, 21].

The study will identify diagnostic and therapeutic biomarkers for TB by integrating multiomics data with MR. Based on the healthy group, TB group, and TB cured group, lipidomic and proteomic data combined with MR were analyzed for biomarker screening. This study first identified lipid and protein biomarkers for TB diagnosis and cure assessment, respectively. Diagnostic and cure models incorporating lipids and proteins were then constructed using the random forest machine learning algorithm, and their efficacy was evaluated. Furthermore, we further explore the biological value and translational potential of core biomarkers by integrating molecular evidence from co‐candidate lipid‐related genes and transcriptomics. These results are expected to provide solid scientific evidence for accurately diagnosing, monitoring treatment, and confirming the cure of TB.

## 2 Results

### 2.1 Plasma Lipidomics Combined With MR for the Identification of Lipid Biomarkers

**FIGURE 1:**
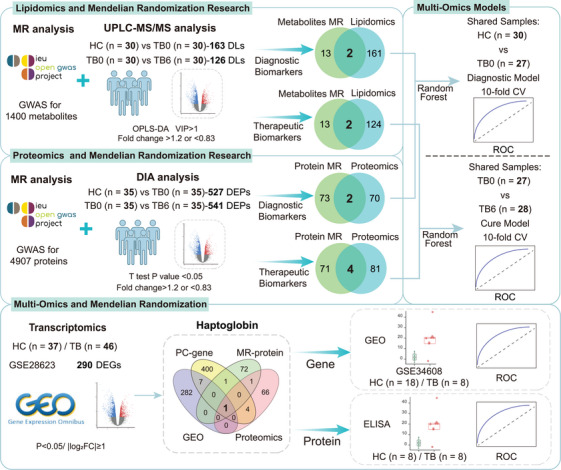
**Study Workflow Diagram**. The study was conducted in four main parts: (1) Identification of diagnostic and therapeutic biomarkers at the metabolic level by integrating Mendelian randomization (MR) analysis of 1400 plasma metabolites with lipidomic screening; (2) Identification of diagnostic and therapeutic biomarkers at the protein level by integrating MR analysis of 4907 plasma proteins with proteomic screening; (3) Construction of multi‐omics diagnostic and therapeutic prediction models using random forest machine learning based on the selected proteins and lipids; (4) Further screening and validation of key biomarkers through the integration of transcriptomics and genes related to the target lipids.

Figure 1 shows the workflow for our analysis. First, 1400 plasma metabolites and their association with TB were subjected to a MR analysis. Based on the inverse variance weighted (IVW) method (*p* < 0.05), with FDR < 0.2, consistent odds ratio (OR) directions across five analytical approaches, and no evidence of pleiotropy, we ultimately identified 15 plasma metabolites (Figure S1; Table S1). These metabolites demonstrated significant causal relationships with TB and may serve as potential biomarkers. Notably, 9 of these 15 metabolites were lipids, suggesting that lipid metabolism plays an important role in initiating and progressing TB.

**FIGURE 2:**
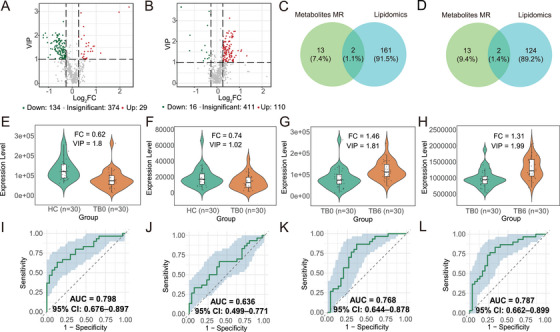
**Mendelian Randomization and Plasma lipidomics for Identifying metabolic biomarkers**. (A) Lipidomic analysis of the TB0/HC groups: Based on the OPLS‐DA model (VIP > 1.0) and screening criteria of fold change ≥ 1.2 or ≤ 0.83, 163 lipid metabolites were significantly altered in the TB0 group (n = 30) compared with the HC group (*n* = 30). (B) Lipidomic analysis of the TB0/TB6 groups: Using the same OPLS‐DA model and criteria, 126 lipid metabolites were significantly altered in the TB6 group (*n* = 30) compared with the TB0 group (*n* = 30). (C) Intersection of MR results with differential metabolites in the TB0/HC groups identified two candidate metabolites: PC(18:2/18:2) and PC(14:0/20:4). (D) Intersection of MR results with differential metabolites in the TB0/TB6 groups identified two candidate metabolites: PC(18:2/18:2) and PC(18:0/20:4). (E) Plasma levels of PC(18:2/18:2) were significantly lower in the TB0 group than in the HC group (*p* < 0.05). (F) Plasma levels of PC(14:0/20:4) were significantly lower in the TB0 group than in the HC group (*p* < 0.05). (G) Plasma levels of PC(18:2/18:2) were significantly higher in the TB0 group than in the TB6 group (*p* < 0.05). (H) Plasma levels of PC(18:0/20:4) were significantly higher in the TB0 group than in the TB6 group (*p* < 0.05). (I) The AUC of PC(18:2/18:2) was 0.798 (95% CI: 0.674–0.897). (J) The AUC of PC(14:0/20:4) was 0.636 (95% CI: 0.499–0.771) (K) The AUC of PC(18:2/18:2) was 0.768 (95% CI: 0.644–0.878). (L) The AUC of PC(18:0/20:4) was 0.787 (95% CI: 0.662–0.899).

We then performed a lipidomic analysis of the plasma samples. Lipids in plasma samples from the TB0 group, the TB6 group and the healthy controls (HC) group were determined using ESI‐QTRAP‐MS/MS. A total of 23 lipid classes and 537 lipid species were identified after rigorous data processing. Based on the OPLS‐DA model (VIP > 1.0) and the criteria of fold change ≥ 1.2 or ≤ 0.83, in comparison to the HC group, 163 lipid species were significantly altered in the TB0 group, with 134 species being down‐regulated and 29 species being up‐regulated (Figure 2A; Table S2). The major classes of differential lipids included triglycerides (TGs), phosphatidylcholines (PCs), phosphatidylethanolamines (PEs), and sphingomyelins (SMs). Similarly, compared with the TB0 group, 126 lipid species were significantly altered in the TB6 group, of which 110 were upregulated and 16 were downregulated (Figure 2B; Table S3). The predominant differential lipid classes comprised TGs, PCs, and PEs.

Next, we intersected the lipidomics differences between the HC and TB0 groups with the MR analysis results (Figure 2C), identifying two lipids, PC (18:2/18:2) and PC(14:0/20:4). Further comparison of TB0 and TB6 groups revealed two lipids, PC(18:2/18:2) and PC(18:0/20:4), as commonly differentially expressed (Figure 2D). Notably, all three significantly altered lipids belonged to PCs, suggesting that PCs may play an important role in the onset and progression of TB.

Scatter plots, funnel plots, forest plots, and leave‐one‐out sensitivity analysis plots were used to visualize the MR analysis results for the target lipids (Figure S2). The IVW method indicated that PC(18:2/18:2) (OR = 1.0954, 95% CI: 1.0148–1.1824, *p* = 0.0195) was a potential risk factor for TB, whereas PC(14:0/20:4) (OR = 0.9002, 95% CI: 0.8253–0.9819, *p* = 0.0176) and PC(18:0/20:4) (OR = 0.9386, 95% CI: 0.9007–0.9781, *p* = 0.0026) were potential protective factors. In addition, reverse MR analysis showed no evidence of reverse causality between these three metabolites and TB (Table S4). Collectively, these findings suggest that PC(18:2/18:2), PC(14:0/20:4), and PC(18:0/20:4) are candidate biomarkers for TB, consistently highlighting the potential importance of PCs in TB pathogenesis. The lipidomic analysis results of the two diagnostic biomarkers and two therapeutic biomarkers are presented in Table 1. Violin plots depict their differential expression between the respective groups (Figure 2E–H). The receiver operating characteristic (ROC) curves demonstrated their good diagnostic performance as biomarkers (Figure 2I–L).

**TABLE 1:** Differential analysis of lipid biomarkers.

Compounds	Class	VIP	Fold_Change	Type	Group
PC(18:2/18:2)	PC	1.80	0.62	down	HC vs. TB0
PC(14:0/20:4)	PC	1.02	0.74	down	HC vs. TB0
PC(18:2/18:2)	PC	1.81	1.46	up	TB0 vs. TB6
PC(18:0/20:4)	PC	1.99	1.31	up	TB0 vs. TB6

### 2.2 Plasma Proteomics Combined With MR for Identifying Protein Biomarkers

**FIGURE 3:**
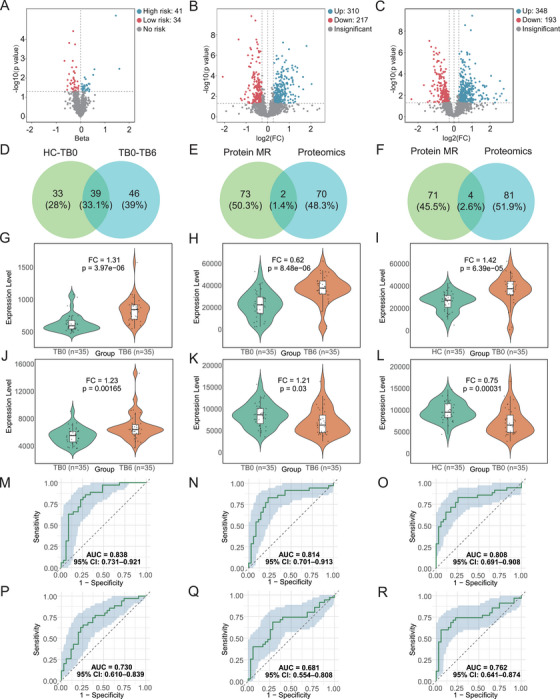
**Plasma Proteomics Combined with Mendelian Randomization Screening for Protein Biomarkers**. (A) Seventy‐five plasma proteins were identified as causally associated with tuberculosis. (B) Volcano plot: Under the criteria of fold change > 1.2 or < 0.83 and *p* < 0.05, analysis of plasma proteins from 35 TB0 samples and 35 HC samples identified 527 differentially expressed proteins (DEPs). (C) Analysis of plasma proteins from 35 TB0 samples and 35 TB6 samples identified 541 DEPs. (D) A Venn diagram illustrated the overlap between DEPs from the HC versus TB0 comparison and those from the TB0 vs. TB6 comparison. (E) Integration of the proteomic results from the HC versus TB0 comparison with MR analysis yielded two potential diagnostic biomarkers. (F) Integration of the proteomic results from the TB0 versus TB6 comparison with MR analysis yielded four potential therapeutic biomarkers. (G) Plasma levels of F13B were significantly higher in the TB6 group than in the TB0 group (*p* < 0.05). (H) Plasma levels of HP were significantly higher in the TB6 group than in the TB0 group (*p* < 0.05). (I) Plasma levels of HP were significantly higher in the TB0 group than in the HC group (*p* < 0.05). (J) Plasma levels of ITIH1 were significantly higher in the TB6 group than in the TB0 group (*p* < 0.05). (K) Plasma levels of RBP4 were significantly lower in the TB6 group compared with the TB0 group (*p* < 0.05). (L) Plasma levels of RBP4 were significantly lower in the TB0 group compared with the HC group (*p* < 0.05). (M) In distinguishing between the TB0 and TB6 groups, the AUC value of F13B was 0.838 (95% CI: 0.731–0.921). (N) In distinguishing between the TB0 and TB6 groups, the AUC value of HP was 0.814 (95% CI: 0.701–0.913). (O) In distinguishing between the HC and TB0 groups, the AUC value of HP was 0.808 (95% CI: 0.691–0.908). (P) In distinguishing between the TB0 and TB6 groups, the AUC value of ITIH1 was 0.730 (95% CI: 0.610–0.839). (Q) For RBP4, the AUC value was 0.681 (95% CI: 0.554–0.808) in distinguishing between the TB0 and TB6 groups. (R) For RBP4, the AUC value was 0.762 (95% CI: 0.641–0.874) in distinguishing between the HC and TB0 groups.

First, we performed MR analysis on 4907 plasma proteins to assess their potential causal associations with TB. This resulted in the identification of 75 plasma proteins significantly associated with TB, of which 41 were protective factors and 34 were risk factors for the disease (Figure 3A; Table S5). In the proteomic analysis, a total of 2580 proteins were identified. Using a fold change of > 1.2 or < 0.83 and *p* < 0.05, we identified 527 DEPs (310 up‐regulated, 217 down‐regulated) in the TB0 group compared to the HC group (Figure 3B; Table S6). We also confirmed 72 corresponding gene relationships (Table S7). Further the TB6 group compared to the TB0 group revealed 541 significant DEPs (348 upregulated, 193 downregulated) (Figure 3C; Table S8), with 85 confirmed gene relationships (Table S9). Using a Venn diagram, we intersected differentially expressed genes (DEGs) between the TB6 and TB0 groups with those between TB0 and HC groups, identifying 39 common DEGs (Figure 3D; Table S10). These proteins were differentially expressed between the HC and TB0 groups as well as between the TB0 and TB6 groups, suggesting their involvement in disease progression and pathology, with potential as therapeutic targets.

By combining MR analysis with proteomics data, we intersected the results from the HC and TB0 groups with those from the MR analysis, identifying two potential diagnostic biomarkers: Haptoglobin (HP) and retinol binding protein 4 (RBP4) (Figure 3E). Similarly, we intersected the analysis results of the TB0 and TB6 groups with the MR findings, identifying four potential therapeutic biomarkers: Coagulation factor XIII B subunit (F13B), inter‐alpha‐trypsin inhibitor heavy chain 1 (ITIH1), RBP4, and HP (Figure 3F). Notably, RBP4 and HP were repeatedly validated in both analyses, demonstrating their strong potential as diagnostic and therapeutic biomarkers.

Scatter plots, funnel plots, forest plots and leave‐one‐out sensitivity analyses were used to present the results of the MR analysis for the plasma protein biomarkers (Figure S3). Consistent with the results of the proteomic analysis, the analysis showed that elevated levels of HP were linked to an increased risk of TB, whereas elevated levels of RBP4, F13B, and ITIH1 were associated with a decreased risk of TB. Furthermore, reverse MR showed no reverse causality for these four proteins (Table S4), supporting their reliability and robustness as potential TB biomarkers.

The proteomic analysis results of the two diagnostic biomarkers (HP and RBP4) and four therapeutic biomarkers (HP, RBP4, F13B, and ITIH1) are presented in Table 2. Violin plots depict their differential expression between the respective groups (Figure 3G–L). The ROC curves demonstrated their favorable performance as biomarkers (Figure 3M–R).

**TABLE 2:** Differential analysis of protein biomarkers.

Protein description	Gene name	Fold_Change	*t* test *p* value	Type	Group
Haptoglobin	HP	1.42	6.39E‐05	Up	HC vs. TB0
Retinol‐binding protein 4	RBP4	0.75	3.10E‐04	Down	HC vs. TB0
Coagulation factor XIII B chain	F13B	1.31	3.97E‐06	Up	TB0 vs. TB6
Inter‐alpha‐trypsin inhibitor heavy chain H1	ITIH1	1.23	1.65E‐03	Up	TB0 vs. TB6
Haptoglobin	HP	0.62	8.48E‐06	Down	TB0 vs. TB6
Retinol‐binding protein 4	RBP4	1.21	3.19E‐02	Up	TB0 vs. TB6

### 2.3 Enrichment Analysis Reveals Biological Functions

**FIGURE 4:**
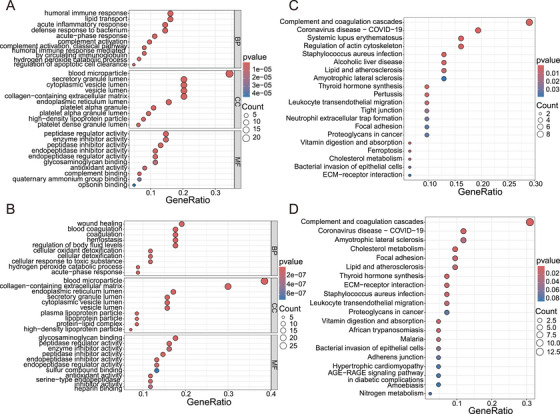
**Enrichment Analysis Reveals Biological Functions**. (A) GO analysis of DEPs between HC group and TB0 group. (B) KEGG analysis of DEPs between HC group and TB0 group. (C) GO analysis of DEPs between TB0 and TB6 groups, (D) KEGG analysis DEPs between TB0 and TB6 groups. The ordinate represents the enriched GO functional classification, which is divided into three major categories: Biological Process (BP), Molecular Function (MF), and Cellular Component (CC). The abscissas indicate the ratio of differential proteins under each functional classification. The color gradient represents the size of the *p* value. The color of bubbles represents the *p* value, and the size of bubbles represents the number of counts.

Gene Ontology (GO) and Kyoto Encyclopedia of Genes and Genomes (KEGG) enrichment analyses were performed on DEPs at different stages of treatment to explore the biological significance of DEPs in TB progression (Table S11) and treatment (Table S12). The GO analysis for biological processes revealed that DEPs in the HC and TB0 groups were significantly involved in processes such as humoral immune response and lipid transport (Figure 4A), while DEPs in the TB0 and TB6 groups were associated with wound healing, coagulation, and hemostasis (Figure 4B). In molecular function, DEPs in the HC and TB0 groups were enriched in processes related to peptidase regulator activity (Figure 4A), whereas those in the TB0 and TB6 groups were linked to enzyme activity and binding (Figure 4B). In terms of cellular components, DEPs in the HC and TB0 groups were primarily located in blood microparticles (Figure 4A), while those in the TB0 and TB6 groups concentrated in the extracellular matrix containing collagen (Figure 4B).

KEGG pathway analysis indicated that DEPs in the HC and TB0 groups were mainly associated with immunity, metabolism, and inflammation regulation (Figure 4C), whereas those in the TB0 and TB6 groups were involved in immune and inflammation regulation, metabolism, and intercellular interactions (Figure 4D). Notably, suggesting a potential link between ferroptosis and TB pathogenesis, KEGG analysis of the HC and TB0 groups revealed significant activation of the ferroptosis pathway.

Pathway enrichment analysis was performed on the 39 common genes identified in the HC, TB0, and TB6 groups to gain further insight into the pathways and biological functions significantly associated with disease progression (Figure S4; Table S13). GO analysis showed significant differences in biological processes related to lipid transport, response to toxic substances, and tissue homeostasis across these groups, highlighting their roles in TB biology. In terms of molecular functions, the DEPs were mainly associated with glycosaminoglycan binding, peptidase regulator activity, and sulfur compound binding. Analysis of cellular components showed that these proteins were mainly enriched in blood microparticles. KEGG pathway enrichment revealed involvement in critical pathways including complement, coagulation cascades, focal adhesion, apoptosis, lipid, and atherosclerosis. These findings provide further understanding of the biological processes involved in the progression of TB, including protein activation, humoral immune reactions, and complement activation.

### 2.4 Multi‐Omics Diagnostic and Therapeutic Models Based on Lipid and Protein Biomarkers

To achieve precise diagnosis and therapeutic prediction of TB, we constructed multi‐omics diagnostic and therapeutic models using a random forest algorithm. For the diagnostic model, two differential lipids (PC (18:2/18:2) and PC (14:0/20:4)) and two DEPs (HP and RBP4) were selected as input features. For the therapeutic model, two differential lipids (PC (18:2/18:2) and PC (18:0/20:4)) and four differential proteins (HP, RBP4, F13B, and ITIH1) were jointly used as features.

**FIGURE 5:**
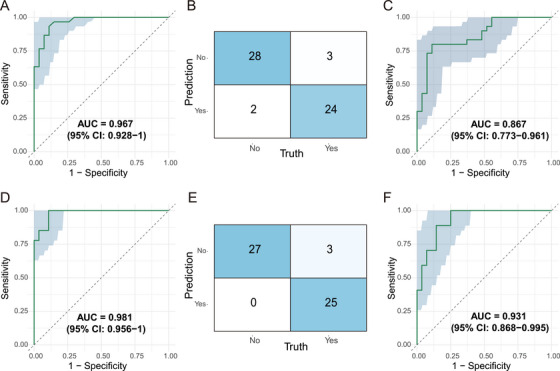
**Performance Evaluation of a Random Forest‐based Model for Predicting the Diagnosis and Cure of Pulmonary Tuberculosis**. (A) The diagnostic model achieved an AUC of 0.967 (95% CI: 0.928–1) in the training set, indicating excellent discriminative performance. (B) The corresponding confusion matrix for the diagnostic model illustrates its classification accuracy in distinguishing positive and negative samples (“Yes” represents TB0 and “No” represents the HC). (C) The mean AUC of the diagnostic model in ten‐fold cross‐validation was 0.867 (95% CI: 0.773–0.961), confirming the robustness of its predictive performance. (D) The ROC curve of the therapeutic prediction model in the training set showed an AUC of 0.981 (95% CI: 0.956–1), demonstrating outstanding discriminative ability. (E) The confusion matrix of the therapeutic prediction model reflects high‐precision classification of samples with and without therapeutic response (“Yes” represents TB6 and “No” represents the TB0). (F) The mean AUC of the therapeutic prediction model in ten‐fold cross‐validation was 0.931 (95% CI: 0.868–0.995), confirming the robustness of its predictive performance.

To objectively evaluate model performance, ten‐fold cross‐validation (10‐fold CV) was employed for all models. The diagnostic model exhibited excellent discriminative ability, with an ROC AUC of 0.967 (95% CI: 0.928–1) (Figure 5A). Confusion matrix analysis further demonstrated high classification accuracy for both positive and negative samples (Figure 5B). The mean AUC from ten‐fold cross‐validation remained high at 0.867 (95% CI: 0.773–0.961) (Figure 5C). Similarly, the therapeutic prediction model achieved outstanding ROC performance and high classification accuracy in both the training set (AUC = 0.981, 95% CI: 0.956–1) (Figure 5D), with the confusion matrix confirming excellent classification of positive and negative samples (Figure 5E). The mean AUC from ten‐fold cross‐validation remained high at 0.931 (95% CI: 0.868–0.995) (Figure 5F). These results demonstrate that the combined lipid and protein biomarkers possess excellent diagnostic and predictive performance for TB and its treatment outcomes.

### 2.5 Multi‐Omics and MR Identifying Robust and Reliable Biological Markers for TB

**FIGURE 6:**
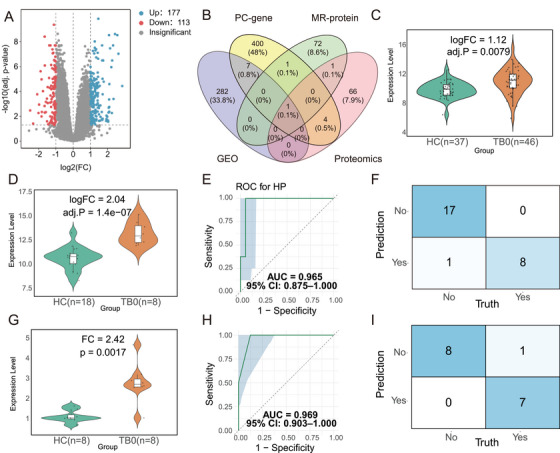
**Multi‐omics combined with Mendelian randomization for robust biomarker discovery in tuberculosis**. (A) Volcano plot of differentially expressed genes (DEGs) between TB patients (*n* = 46) and healthy controls (*n* = 37) from GEO dataset GSE28623. A total of 290 DEGs were identified (177 upregulated and 113 downregulated) under the criteria |log2FC| > 1.2 or < 0.83 and adjusted *p* < 0.05. (B) Integration pipeline: By combining genes related to PC(18:2/18:2) and PC(14:0/20:4) (PC‐gene), MR results for 4907 proteins (MR‐protein), proteomic differential expression results (Proteomics), and GEO transcriptomic data (GEO), we identified haptoglobin (HP) as a target biomarker. (C) Violin plot showing differential expression of HP between TB patients and healthy controls in GSE28623. (D) Violin plot showing differential expression of HP between TB patients (*n* = 8) and healthy controls (*n* = 18) in GSE34608. (E) ROC curve assessing the diagnostic performance of HP expression for discriminating TB patients from healthy controls in the GSE34608 dataset. AUC = 0.965 (95% CI: 0.875–1.000). (F) Confusion matrix for HP‐based classification of TB patients versus healthy controls in the GSE34608 dataset (“Yes” represents tuberculosis patients and “No” represents healthy people). (G) Violin plot showing differential expression of HP between TB patients (*n* = 8) and healthy controls (*n* = 8) in ELISA. (H) ROC curve assessing the diagnostic performance of HP expression for discriminating TB patients from healthy controls in the ELISA. AUC = 0.969 (95% CI: 0.903–1.000). (I) Confusion matrix for HP‐based classification of TB patients versus healthy controls in the ELISA (“Yes” represents tuberculosis patients and “No” represents healthy people).

Through MR and multi‐omics analyses, we successfully identified lipid and protein biomarkers for the diagnosis and therapeutic prediction of TB, demonstrating the considerable potential of their combined application. To further explore the molecular functional mechanisms underlying these biomarkers and strengthen the level of evidence, we incorporated transcriptomic datasets from the GEO database and integrated genes related to the target lipids from the GeneCards database. The key biomarkers were then subjected to in‐depth functional enrichment analysis and validation. We used transcriptomic data from TB patients in the GEO dataset GSE28623 to identify DEGs. A total of 290 DEGs were selected between the TB patient group and HC, including 177 upregulated and 113 downregulated genes (Figure 6A). The results of the differential analysis are shown in a heatmap (Figure S5). GO and KEGG pathway analyses revealed the enriched pathways of these DEGs (Figure S6; Table S14). We then used GeneCards to identify the related genes encoding proteins for PC(18:2/18:2) and PC(14:0/20:4). By integrating the results from MR, Proteomic DEPs (HC vs. TB0), and the transcriptomic analysis of GEO datasets, we ultimately identified the target biomarker HP (Figure 6B). We show the differential expression of HP in the GSE28623 dataset (Figure 6C) and validated this finding in GSE34608 (Figure 6D). ROC analysis (Figure 6E) and the confusion matrix (Figure 6F) demonstrated excellent diagnostic performance for HP (AUC = 0.965, 95% CI 0.875–1.000). Independent ELISA validation similarly showed that HP was significantly upregulated in pulmonary TB patients compared with HC (Figure 6G). ROC analysis (Figure 6H) and the confusion matrix (Figure 6I) based on ELISA‐measured HP levels confirmed its strong discriminatory ability (AUC = 0.969, 95% CI 0.903–1.000). We stratified samples based on HP expression levels and performed Gene Set Variation Analysis (GSVA) to assess pathway differences between groups. The analysis revealed 524 significant GO pathways and 12 KEGG pathways associated with HP expression (Table S15). The results showed that in the HP high‐expression group, pathways related to inflammation and immune response, lipid metabolism and energy regulation, signal transduction and transcriptional regulation, as well as development and differentiation were significantly upregulated, whereas pathways associated with antigen presentation, immune cell migration, antimicrobial secretion, host defensive stress response, and iron homeostasis were significantly downregulated (Figure S7). These findings suggest that HP may modulate oxidative stress, apoptosis, inflammatory pathways, and lipid metabolism, while also influencing immune responses, host defensive stress mechanisms, and the iron supply environment. Collectively, these effects could synergistically impair innate and adaptive immune barriers, creating conditions that facilitate Mtb evasion and persistent infection.

## 3 Discussion

This study systematically revealed potential biomarkers and underlying biological mechanisms of TB through the integration of multi‐omics and MR analyses. First, MR was applied to screen plasma metabolites and proteins that showed causal relationships with TB from a genetic perspective. Their expression changes were subsequently validated in a clinical cohort using lipidomic and proteomic experiments, ultimately identifying lipids (PC(18:2/18:2), PC(14:0/20:4) and PC(18:0/20:4)) and proteins (HP, RBP4, F13B and ITIH1) as candidate diagnostic and cure biomarkers. Furthermore, diagnostic and cure‐prediction models combining lipidomic and proteomic features were constructed and demonstrated excellent performance. By integrating transcriptomic data mining with lipid‐associated genes, HP was further pinpointed as a core molecule. HP was then validated at multiple levels—transcriptomically using GEO datasets and at the protein level by independent ELISA experiments. Functional enrichment analyses revealed that HP may play a critical role in TB pathogenesis by regulating ferroptosis, immune responses, and lipid metabolism. Collectively, this study provides robust multi‐omics evidence and candidate targets for the precise diagnosis, therapeutic monitoring, and mechanistic understanding of TB.

PC constitutes an essential component of cellular membranes and plays a central role in diverse biological processes [22]. As a principal element of pulmonary surfactant, PC is critical for preserving alveolar stability [23]. Previous studies have suggested that abnormalities in PC levels can impact membrane fluidity and functionality [24]. The lipase expressed by MTB can hydrolyze PC in host cells, releasing fatty acids that are crucial for MTB metabolism, survival, and pathogenicity [25]. PC also functions as a carrier for the storage and transport of fatty acids, phosphate, glycerol, and choline. Reduced PC levels may compromise the bioavailability of choline, an essential nutrient. Importantly, MTB relies on host lipid metabolism for growth and can exploit PC and its degradation products, such as choline, as carbon sources [26]. During the progression of TB, specific PC molecules may contribute to the disease via various mechanisms. Infection with MTB provokes immune cells to secrete inflammatory mediators such as Tumor Necrosis Factor‐alpha (TNF‐α) and interleukins (ILs). While these cytokines are critical for the regulation of inflammation, their overproduction can contribute to tissue injury and worsening of disease [27]. In particular, PC serves as the precursor of lysophosphatidylcholine (LysoPC), an inducer of T lymphocytes [28], and specific molecular species, such PC(18:2/18:2) has been shown to promote TNF‐α synthesis [29], suggesting a potential role for PC in TB‐related immune response and inflammation.

F13B is the final enzyme in the coagulation cascade. By associating with the catalytic A subunit (F13A), it generates the active transglutaminase factor XIII (FXIII) [30]. The central biological role of FXIII is to catalyze the covalent cross‐linking of fibrin monomers, thus stabilizing the fibrin clot, which is essential for hemostasis and wound healing [31]. Beyond its role in fibrin cross‐linking, FXIII critically contributes to cell adhesion, stabilization of the extracellular matrix, and modulation of inflammatory responses, ultimately shaping tissue repair and host defense [30]. Studies have shown that TB is linked to systemic activation of the coagulation cascade and impaired anticoagulant mechanisms, thereby placing patients in a sustained “net procoagulant state” [32]. In TB patients, plasma levels of F13B are significantly lower at TB0 compared with TB6. This observation reflects the hypercoagulable status characteristic of TB and further suggests that F13B dysregulation may directly participate in disease pathogenesis. Active TB entails pronounced local and systemic inflammation together with a hypercoagulable milieu, driving extensive microvascular thrombosis and tissue damage, which may account for consumptive depletion of F13B. With treatment, as the inflammatory burden diminishes, F13B synthesis appears to progressively recover and may even increase in a compensatory fashion.

ITIH1 covalently associates with hyaluronic acid to generate a stable complex [33], thereby acting as a principal carrier protein for hyaluronic acid in serum. Furthermore, ITIH1 functions as a bridging protein between hyaluronic acid and other matrix proteins, regulating the localization, synthesis, and degradation of hyaluronic acid and contributing to extracellular matrix remodeling. Through these actions, ITIH1 helps maintain tissue homeostasis and mediates cell migration and signaling [34]. During TB pathology, MTB infection triggers lung tissue inflammation, leading to pronounced tissue damage and extracellular matrix degradation [35]. ITIH1 is likely to be involved in this process, as ITIH1‐mediated alterations in the cross‐linking state of hyaluronic acid markedly influence extracellular matrix permeability and thereby modulate the rate of inflammatory cell migration toward sites of infection [36].

RBP4 may regulate pathological processes during bacterial infections. Characteristic pathological changes in TB include foam macrophages, granulomas, and caseous necrosis [37]. RBP4 contributes to the excessive production of pro‐inflammatory cytokines and leukocyte recruitment by activating mitogen‐activated protein kinases (MAPK), nuclear factor‐kappa‐light‐chain‐enhancer of activated B cells (NF‐κB) and c‐Jun (JUN) signaling pathways [38]. In addition, RBP4 promotes macrophage cholesterol uptake via CD36, contributing to foam macrophage formation [39]. Therefore, during MTB infection, decreased expression of RBP4 may hinder the formation of foam macrophages and slow the development of granulomas and caseous necrosis in TB. Furthermore, RBP4 serves as the specific carrier protein for retinol (vitamin A). Retinoic acid promotes monocyte differentiation, thereby restricting the intracellular growth of Mtb in human macrophages. Notably, RBP4 levels are markedly reduced in whole‐blood supernatants of patients with active pulmonary TB [40]. These findings highlight the importance of RBP4 across different pathological stages of TB. However, the precise role of RBP4 in TB and its clinical relevance require further experimental investigation and clinical validation. Such studies could inform the development of novel therapeutic strategies targeting RBP4‐mediated inflammation and foam cell formation to improve TB treatment outcomes.

HP is an acute‐phase response glycoprotein that binds to hemoglobin (Hb) and enhances its peroxidase activity [41, 42]. Lesions in PTB frequently induce local tissue destruction, inflammatory cell infiltration, and microvascular damage, which collectively leads to respiratory distress and hypoxia [35]. Under hypoxic conditions, the structure of Hb increases its spontaneous oxidation rate, resulting in excessive reactive oxygen species production [43]. HP can bind free Hb in plasma to form a stable complex, thereby inhibiting its pro‐oxidative activity and conferring both antioxidant and anti‐inflammatory effects [44], similar to the functions of PC [45]. Accordingly, the marked increase in plasma HP levels observed in the TB0 group indicates a critical host defense against infection‐driven hemolysis, hypoxia, and oxidative stress. Following effective therapy, the reduction in HP levels mirrors the resolution of inflammation, mitigation of tissue injury, and attenuation of oxidative stress.

There may be a potential link between HP and PC, which could play a role in cellular protection and inflammation regulation under pathological conditions. During the physiological process of erythrocyte turnover, as the major Hb scavenger in plasma, HP binds free Hb with high affinity to form stable complexes, effectively neutralizing its toxic effects [42]. In pathological conditions like TB, sustained inflammation and pathogen‐associated molecular patterns can disrupt cell membrane integrity, and the expression of PC in plasma may change significantly [46]. During inflammatory responses, plasma levels of HP are concomitantly increased [47]. Under inflammatory conditions, plasma HP concentrations rise. This increase not only augments the ability of HP to scavenge excessive Hb released during hemolysis, but also indirectly protects PC‐enriched cell membranes by preventing Hb‐driven lipid peroxidation and membrane disruption. Therefore, HP and PC may participate in the inflammation response and immune modulation, jointly playing a role in the activation of inflammatory cells and membrane remodeling during the development of TB.

Past research has typically concentrated on a single type of omics data. In contrast, this study integrates multiple omics data sources and applies MR to comprehensively screen for potential robust biological biomarkers for TB. MR analysis uses genotype as an instrumental variable to achieve unbiased causal inference, effectively excluding potential confounding factors and reverse causal interference, thereby clarifying the causal association between the screened biomarkers and diseases. This strategy significantly enhances the robustness and reliability of the biomarkers, ensuring that the finally identified biomarkers possess both disease‐driving properties and clinical application potential. Integrative multi‐omics analysis mitigates the weaknesses associated with single‐source datasets. The combined biomarkers, derived from plasma lipids and proteins, provide a more complete picture of the pathological state, reducing errors caused by variations in single factors or other biological influences, and facilitating more accurate disease subtyping and subgroup identification. The integration of this multi‐layered information enhances the overall capture of disease states, making diagnosis more precise. In addition, by integrating lipid‐associated gene signatures with transcriptomic profiling, we provide multidimensional molecular evidence implicating the target protein. This strategy links protein‐level observations to lipidomic findings and gene‐level data, thereby bridging omics layers and enabling cross‐omics integrative analysis. It is noteworthy that the multi‐omics diagnostic model (PC (18:2/18:2), PC (14:0/20:4), HP, RBP4) and cure‐monitoring model (PC (18:2/18:2), PC (18:0/20:4), HP, RBP4, F13B, and ITIH1) developed in this study show higher specificity and sensitivity compared to traditional biomarkers such as C‐reactive protein (sensitivity below 90% and specificity below 70%) for the diagnosis and therapeutic surveillance of TB [48, 49]. Compared with previously reported machine‐learning diagnostic models, the multi‐omics diagnostic model developed in this study outperforms earlier approaches [50, 51]. For treatment‐monitoring models, our multi‐omics model for monitoring cure demonstrated superior specificity and sensitivity over existing molecular signatures [52, 53].

Despite the significant achievements of this research, several limits remain. First, the sample size was limited, which may limit the general applicability of the identified biomarkers. Second, Although HP's diagnostic potential and mechanisms have been preliminarily shown, its specific role in TB have not been fully elucidated. Lastly, independent clinical samples are needed to further validate these findings.

Future studies should focus on expanding sample sizes and validating these findings in multiple centers to establish the generalizability of the biomarkers. Furthermore, exploring the role of HP and other biomarkers in inflammation regulation could provide innovative insights for developing new targeted therapeutic strategies.

## 4 Conclusion

This study successfully identified lipids (PC(18:2/18:2), PC(14:0/20:4) and PC(18:0/20:4)) and proteins (HP, RBP4, F13B and ITIH1) as robust biomarkers for the diagnosis and monitoring of cure in TB, through the integration of lipidomics, proteomics and MR analysis and demonstrate high‐accuracy multi‐omics diagnosis and cure monitoring models. In addition, biomarker HP has obtained multi‐dimensional molecular evidence (e.g., transcriptomics, proteomics), and has been revealed that it may participate in the infection process and immune escape mechanism of Mtb by interfering with host iron homeostasis and regulating immune responses. Our findings not only provide effective tools for TB diagnosis and treatment monitoring, but also lay a solid foundation for a deeper understanding of the disease mechanisms and the exploration of therapeutic targets.

## 5 Materials and Methods

The study design and procedures comply with the Declaration of Helsinki and have been approved by the hospital of Taizhou Enze Medical Center (Group). Informed consent was obtained from all enrolled participants prior to blood sample collection. Furthermore, the methods used in this study adhere to the approved guidelines and regulations.

### 5.1 Lipidomics

#### 5.1.1 Source of Lipidomics Data

Between May and December 2019, plasma samples were collected from 30 patients with a new diagnosis of TB before starting treatment (TB0 group), 30 cured patients after the intensive and consolidation phases of treatment (TB6 group), and 30 HC group at the hospital of Taizhou Enze Medical Center (Group). These samples were used for lipidomics analysis. Between the TB0, TB6, and HC groups, there were no statistically significant differences (*p* > 0.05) in the mean age or gender distribution. In addition, no statistically significant differences were observed in the sputum smear, culture, PCR chip, Xpert MTB/RIF, or T spot positivity rates between the three groups (*p* > 0.05) (Table 3). The diagnosis of TB was based on the presence of one or more of the following criteria (1) Positive sputum test (smear or culture). (2) Negative sputum examination but a chest x‐ray or CT scan showing typical signs of active TB. (3) Pathological diagnosis of TB from lung specimens. (4) Suspected pulmonary TB after clinical follow‐up examination and x‐ray to exclude other lung diseases. (5) Clinically exclude other causes of pleural effusion and diagnose tuberculous pleuritis. Enrolment was based on standard TB treatment regimens and drug response. In particular, the TB6 group included patients receiving two months of intensive treatment and then four months of maintenance treatment. Patients were excluded from this study if they had extrapulmonary tuberculosis, malignancy, chronic disease, autoimmune disease, or HIV infection.

**TABLE 3:** Statistical analysis of the clinical characteristics and laboratory parameters of samples used for lipidomic analysis.

	Healthy controls (*N* = 30)	Untreated TB (*N* = 30)	Cured TB (*N* = 30)	*p*‐value
Age(median, IQR)	33.5(27.7–47.7)	32.0(22.7–54.0)	34.0(25.2–47.5)	0.8577^a^
Gender(male)	17(56%)	13(43%)	20(67%)	0.1892^b^
Sputum smear:Positive, no. (%)	/	16(53%)	14(47%)	0.7963^b^
Xpert MTB/RIF:Positive, no. (%)	/	20(67%)	19(63%)	1^b^
PCR chip:Positive, no. (%)	/	10(33%)	16(53%)	0.1927^b^
Cultivate:Positive, no. (%)	/	16(53%)	16(53%)	1^b^
T‐spot.TB:Positive, no. (%)	/	20(67%)	20(67%)	1^b^

*Note: N*, number of subjects; TB0, untreated TB patients; TB6, cured TB subjects.

Abbreviation: HC, health controls.

^a^ *p* value among four groups from Kruskal–Wallis H test.

^b^ *p* value among four groups from the Chi‐square test.

#### 5.1.2 Quantitative and Statistical Analysis of Lipidomics

Sample preparation was followed by lipid metabolite separation using ultra‐high performance liquid chromatography (UPLC) on a Shim‐pack UFLC SHIMADZU CBM A system coupled to the QTRAP 6500+ MS system. The chromatographic separation was carried out on a Thermo C30 column (2.1 mm × 100 mm, 2.6 µm) with the column temperature set at 45°C. Mobile phase was composed of acetonitrile/water (60/40, v/v) containing 0.04% acetic acid and 5 mmol/L ammonium formate (A), and acetonitrile/isopropanol (10/90, v/v) containing 0.04% acetic acid and 5 mmol/L ammonium formate (B). The elution gradient was as follows: 0 min, 20% B; 3 min, 50% B; 9 min, 75% B; 15 min, 90% B; followed by equilibration at 50% B. The flow rate was 350 µL/min, with an injection volume of 2 µL.

Initially separated plasma samples were entered into the QTRAPLC‐MS/MS system and scanned in a triple quadrupole containing ion trap. The system is equipped with ESI Turbo ion spray port, which can be operated under positive ion and negative ion mode and controlled by Analyst 1.6.3 software. The parameters of the ESI source were set as the following: The temperature of the ion source was set to 550°C, the ion spray voltage was set to 5500 V in positive ion mode (or −4500 V in negative ion mode), the ion source Gas I, the Gas II, the curtain gas were set to 55, 60, and 25 psi, respectively, and the collision gas was set to medium. In total, 10 and 100 µmol/L polypropylene glycol solution were used for instrument tuning and quality PPG calibration, respectively. QQQ scans were acquired as MRM experiments with collision gas (nitrogen) set to 5 psi. Declustering potential (DP) and collision energy (CE) for individual MRM transitions were done with further DP and CE optimization. A specific set of MRM transitions were monitored for each period according to the metabolites within this period [54, 55]. The details of the MRM method are presented in Table S16.

The mass spectrum data were processed by Software Analyst 1.6.3. The reproducibility of the extraction and detection of lipid metabolites in the positive and negative ion mode was determined by the total ion flow chromatogram of the quality control (QC) samples mixed and prepared from extracts of samples. Metabolite structure analysis referred to some existing mass spectrometry public databases, mainly including massbank (http://www.massbank.jp/), knapsack (http://kanaya.naist.jp/knapsack/), HMDB (http://www.hmdb.ca/), and Metlin (http://metlin.scripps.edu/index.php). Qualitative analysis was carried out according to the retention time of the test substance and the mass to charge ratio of parent‐daughter ions. Lipid was classified and named in strict accordance with LIPID MAPS (www.lipidmaps.org). The quantitative analysis was completed by multiple reaction monitoring (MRM) of triple quadrupole mass spectrometry. The signal intensity of characteristic ions was obtained in the detector. MultiQuant was used to integrate and calibrate the chromatographic peaks.

A total of 90 samples were selected and divided into three groups for lipidomic analysis. Overall, 23 lipid classes and 537 lipid species were identified. Chi‐squared tests and Kruskal–Wallis H tests were used to evaluate differences between groups. Lipidomics data were normalized using R software (https://www.r‐project.org/). Multivariable analysis was performed using MetaboAnalyst 4.0 (http://www.metaboanalyst.ca), with lipid analysis conducted using orthogonal partial least squares (OPLS) models.

Based on the results of the orthogonal partial least squares discriminant analysis (OPLS‐DA), lipids with variable importance in projection (VIP) > 1 from the multivariate OPLS‐DA model were preliminarily selected as differential lipids between HC and TB0, and between TB0 and TB6. In addition, differential lipids were further filtered by univariate analysis using fold‐change thresholds of FC > 1.2 or FC < 0.83. Volcano plots were generated with the “ggplot2” package to visualize differential lipids. Finally, ROC curves and confusion matrices were constructed to analyze the AUC, sensitivity and specificities of the candidate markers between groups.

### 5.2 Proteomics

#### 5.2.1 Source of Proteomics Data

A total of 105 plasma samples, including 35 newly diagnosed untreated TB patients (TB0 group), 35 cured patients after intensive and continuous treatment (TB6 group), and 35 HC group, were collected from the hospital of Taizhou Enze Medical Center (Group) for proteomic analysis. Between the TB0, TB6, and HC groups, there were no statistically significant differences (*p* > 0.05) in the mean age or gender distribution. In addition, no statistically significant differences were observed in the sputum smear, culture, PCR chip, Xpert MTB/RIF or T spot positivity rates between the three groups (*p* > 0.05) (Table 4). The diagnostic criteria for TB are consistent with those for lipidomics.

**TABLE 4:** Statistical analysis of the clinical characteristics and laboratory parameters of samples used for proteomic analysis.

	Healthy controls (*N* = 35)	Untreated TB (*N* = 35)	Cured TB (*N* = 35)	*p*‐value
Age (median, IQR)	38(28–50)	34(25–53)	34(24–49)	0.7175^a^
Gender (male)	20(57%)	16(46%)	23(66%)	0.239^b^
Sputum smear: positive, no. (%)	/	19(54%)	16(46%)	0.6326^b^
Xpert MTB/RIF: positive, no. (%)	/	24(68%)	19(54 %)	0.326^b^
PCR chip: positive, no. (%)	/	10(29%)	16(46 %)	0.2162^b^
Cultivate: positive, no. (%)	/	20(57%)	17(49 %)	0.632^b^
T‐spot. TB: positive, no. (%)	/	20(57%)	19(54 %)	1^b^

*Note: N*, number of subjects; TB0, untreated TB patients; TB6, cured TB subjects.

Abbreviation: HC, health controls.

^a^ *p* value among four groups from Kruskal–Wallis H test.

^b^ *p* value among four groups from the Chi‐square test.

#### 5.2.2 Quantitative and Statistical Analysis of Proteomics

A multi‐affinity removal column (Hu‐14, Agilent Technologies) was used to remove high‐abundance proteins and retain low‐abundant proteins. The analysis was performed using a Thermo Scientific Q Exactive HF‐X mass spectrometer coupled to an easy nLC 1200 chromatographic system (Thermo Scientific) to generate data‐dependent acquisition (DDA) spectral libraries. For data‐independent acquisition (DIA) analysis, 1.5 µg of peptide was extracted from each sample. The ratio of indexed retention time (iRT) standard peptide to sample peptide was 3:1. The DDA method was used for the generation of the spectral library and the DIA mode was used for both the qualitative and quantitative analysis of the proteins. MaxQuant 1.5.3.17 software was used for database searching. The spectral library was constructed using Spectronaut 14.4.200727.47784 software. iRT‐related peptide sequences were added to the database (>Biognosys|iRTKit|Sequence_fusionLGGNEQVTRYILAGVENSKGTFIIDPGGVIRGTFIIDPAAVIRGAGSSEPVTGLDAKTPVISGGPYEYRVEATFGVDESNAKTPVITGAPYEYRDGLDAASYYAPVRADVTPADFSEWSKLFLQFGAQGSPFLK). The database was downloaded using the files human_156639_20170105.fasta and human_159691_20170829.fasta from the Universal Protein Resource (Uniprot) (https://www.uniprot.org). Qualitative and quantitative analysis of DIA data was achieved by parsing the MS/MS spectral information. Protein identification was performed with a 99% confidence threshold determined by the false discovery rate (FDR = N (decoy) * 2 / (N (decoy) + N (target)) ≤ 1%). Comprehensive QC was implemented across the entire workflow—from sample preprocessing to data analysis—by removing high‐abundance proteins, calibrating with standard peptides, applying a stringent protein identification false discovery rate (FDR ≤ 1%), and visualizing overall QC metrics, thereby ensuring the reliability of the experimental results (Figure S8). The QC samples in this process are prepared by mixing the sample extracts. Detailed parameters for liquid chromatography (LC) separation and mass spectrometry (MS) acquisition are provided in Table S17.

Proteomic analysis of plasma samples from 105 participants across three groups was performed using a DIA platform, yielding quantitative data for 2580 proteins. Between‐group differences were evaluated using the Chi‐square and Kruskal–Wallis H tests. DEPs were identified by combining fold‐change criteria (FC > 1.2 or FC < 0.83) with Student's *t*‐test (*p* < 0.05). Volcano plots generated with the ggplot2 package were used to visualize differential proteins. Functional enrichment analyses of DEPs in GO terms and KEGG pathways were performed using the clusterProfiler R package. ROC curves and confusion matrices were constructed to assess the AUC, sensitivity, and specificity of candidate biomarkers for between‐group comparisons.

### 5.3 MR Analysis

For the MR analysis, relevant data were obtained from genome‐wide association study (GWAS) databases. Summary statistics for 1400 plasma metabolites were sourced from the Canadian Longitudinal Study on Aging (CLSA), which included 8299 unrelated participants [18]. The GWAS ID used for the outcomes was ebi‐a‐GCST90018892, which includes data from 477,386 individuals (476,491 controls and 895 cases). Data for plasma proteins were derived from a large‐scale integrative study, providing information on 4907 plasma proteins from 35,559 Icelandic individuals [56]. The GWAS ID for this dataset was ebi‐a‐GCST90018672, comprising 178,671 individuals (170,871 controls and 7800 cases).

In MR analysis, plasma metabolites and proteins were treated as exposures, with TB as the outcome. The MR analysis was performed using TwoSampleMR, VariantAnnotation, and gwasglue in R (version 4.4.1). To meet the correlation assumption, we set the genome‐wide significance threshold for blood metabolites and plasma proteins at *p* < 1.0 × 10^−5^, and the linkage disequilibrium threshold at *r* ^2^ = 0.001, kb = 10,000, to ensure single nucleotide polymorphism (SNP) independence. To exclude weak instruments, we included only those with an F‐value > 10 in the MR analysis. Causal effects were analyzed using the IVW method, MR‐Egger regression, weighted median method, simple mode method, and weighted mode method. Significance was considered when the *p*‐value for the IVW method was < 0.05. Consistency in the direction of the OR across all five analysis methods was also required. Heterogeneity was tested using Cochran's Q statistic, with a *p*‐value greater than 0.05 indicating the absence of heterogeneity. Pleiotropy was assessed using the MR‐PRESSO test and the MR‐Egger regression intercept. If the *p*‐value from the MR‐PRESSO global test and the MR‐Egger intercept was greater than 0.05, it indicated the absence of pleiotropy. In addition, the reverse MR analysis P is greater than 0.05 indicating no reverse causation.

### 5.4 Multi‐Omics Integrative Modeling

A total of 85 matched samples from lipidomics and proteomics datasets were selected (HC group: *n* = 30; TB0: *n* = 27; TB6: *n* = 28). First, lipidomic and proteomic expression matrices were integrated by sample, and the combined feature matrix was subjected to Z‐score normalization to eliminate scale differences between the two omics layers. Based on the integrated multi‐omics data, binary random forest models were then constructed using the tidymodels package to discriminate TB patients from HC (HC vs. TB0) and to evaluate treatment efficacy (TB0 vs. TB6). Model stability and generalizability were assessed by 10‐fold cross‐validation. The key parameters of the random forest were set as follows: mtry (number of candidate features) ranged from 2 to 10, trees (number of decision trees) ranged from 200 to 500, and min_n (minimum sample size for node splitting) ranged from 20 to 50. ROC curves and confusion matrices were generated to evaluate the AUC, sensitivity, and specificity of the models.

### 5.5 Transcriptomics Analysis

The transcriptomic data were sourced from the GEO database. We selected and downloaded datasets GSE28623 (HC group: 37 cases; TB group: 46 cases) and GSE34608 (HC group: 18 cases; TB group: 8 cases) for differential analysis and diagnostic performance evaluation, respectively. Selection criteria for datasets were as follows: The experimental design of gene expression profiling datasets must involve whole‐blood studies of patients with active TB and HC; each gene expression dataset must include more than five samples for both groups; and the samples used for gene expression profiling must contain RNA expressed at the whole‐genome level in humans [57].

GEO datasets were downloaded and analyzed in R using the limma package to identify and characterize DEGs in the blood of healthy and TB‐infected individuals. DEGs were screened based on an adjusted *p*‐value < 0.05 and |log_2_ FC| ≥ 1. Volcano plots and heatmaps were generated with the “ggplot2” and “pheatmap” packages to visualize the DEGs.

### 5.6 ELISA

A total of 16 participants were enrolled, including 8 pulmonary TB patients and 8 HC. Plasma HP concentrations were measured using the QuicKey Pro Human HP (Haptoglobin) ELISA Kit (Catalog No. E‐OSEL‐H0044; Elabscience) according to the manufacturer's instructions. Group comparisons of HP concentration were performed using the Student's *t*‐test; two‐sided *p* values < 0.05 were considered statistically significant. ROC curves, AUC, and 95% confidence intervals were generated using the pROC package. The study protocol was approved by the hospital of Taizhou Enze Medical Center (Group).

### 5.7 GSVA and Functional Enrichment Analysis

GSVA is an unparametric and unsupervised way for the assessment of gene set enrichment based on mRNA expression data. In this study, gene sets from the Molecular Signatures Database (v7.0), including c5.go.Hs.symbols.gmt and c2.cp.kegg.Hs.symbols.gmt, were used. The GSVA algorithm was used to calculate enrichment scores for each set of genes and to assess the potential biological differences between the high‐risk and low‐risk groups. KEGG and GO enrichment analyses were performed using the R clusterProfiler package, and GO terms and KEGG pathways were defined as significant when *p* and *q* values were <0.05.

## Author Contributions

L. Z. and JC. L. designed the study; C. Z., Q. L., Y. L. and Q. Z. contributed the experiments and materials; J. C., Y. L. and Q. Z. drafted the original paper; C. Z., M. M. A. K. K., N. Z. and H. F. produced data visualization; J. C., Q. L. and C. Z. analyzed the data; H. F., C. Z., L. Z., and JC. L. revised and edited this paper. All authors have read and approved the final manuscript.

## Funding

This work has been financed by the Key R&D and Promotion Projects in Henan Province (No. 242102310407), Key Scientific Research Projects of Colleges and Universities in Henan Province (No. 23A310011).

## Ethics Statement

This study was approved by the Medical Ethics Committee of the hospital of Taizhou Enze Medical Center (Group) (K20230315) and signed informed consents were obtained from participants or their legal guardians.

## Conflicts of Interest

The authors declare no conflicts of interest.

## Supporting information




**Figure S1** Analysis of 15 plasma metabolites causally associated with tuberculosis.
**Figure S2** MR analysis results for target metabolites. Scatter plots (A, E, I), funnel plots (B, F, J), forest plots (C, G, K), and leave‐one‐out sensitivity analysis plots (D, H, L).
**Figure S3** MR results for target plasma proteins. Scatter plots (A, E, I, M), funnel plots (B, F, J, N), forest plots (C, G, K, O), and leave‐one‐out sensitivity analysis plots (D, H, L, P).
**Figure S4** GO and KEGG enrichment analysis. (A) GO analysis of 39 common DEPs from HC, TB0, and TB6 groups. (B) KEGG analysis of 39 common DEPs from HC, TB0, and TB6 groups. The ordinate represent the enriched GO functional classification, which is divided into three major categories: Biological Process (BP), Molecular Function (MF), and Cellular Component (CC). The abscissas indicates the Ratio of differential proteins under each functional classification. The color gradient represents the size of the *p* value. The color of bubbles represents the *p* value, and the size of bubbles represents the number of counts.
**Figure S5** Heat map of differentially expressed genes in the transcriptome.
**Figure S6** GO and KEGG enrichment analysis. (A) GO analysis of DEGs (B) KEGG analysis of DEGs. The ordinate represent the enriched GO functional classification, which is divided into three major categories: Biological Process (BP), Molecular Function (MF), and Cellular Component (CC). The abscissas indicates the Ratio of differential proteins under each functional classification. The color gradient represents the size of the *p* value. The color of bubbles represents the *p* value, and the size of bubbles represents the number of counts.
**Figure S7** Gene Set Variation Analysis. (A) Distribution of GSVA scores for GO pathways showing significant differences between groups stratified by HP expression level. (B) Distribution of GSVA scores for KEGG pathways showing significant differences between groups stratified by HP expression level. The x‐axis denotes the t value of the GSVA score and the y‐axis lists pathway names.
**Figure S8** Quality control (A) Sample clustering based on the protein expression profile. A three‐dimensional principal component analysis (PCA) scatter plot was generated from the expression levels of all quantified proteins to visualize clustering of healthy controls (HC), QC samples, and the two disease groups (TB0, TB6). QC samples cluster tightly, indicating high experimental stability and low technical variation; samples from different study groups exhibit clear separation trends, revealing distinct proteomic signatures and supporting the reliability of subsequent differential analyses. (B) Distribution of protein quantification CVs in QC samples. The histogram depicts the frequency distribution of CV values for all proteins measured in QC samples, providing an overall assessment of the precision and reproducibility of the proteomic quantification. The red vertical line denotes the median CV (20%), a key metric of assay stability. The majority of proteins show low CVs, indicating high‐quality, reliable quantitative data.
**Table S1**: 15 plasma metabolites screened by Mendelian randomization analysis.
**Table S2**: Differential lipids between the HC group and the TB0 group identified by lipidomics.
**Table S3**: Differential lipids between the TB0 group and the TB6 group identified by lipidomics.
**Table S4**: Results of reverse Mendelian randomization analysis for target lipids and proteins.
**Table S5**: 75 plasma proteins screened by Mendelian randomization analysis.
**Table S6**: Differential proteins between the HC group and the TB0 group identified by proteomics.
**Table S7**: Gene correspondence of differential proteins between the HC group and the TB0 group.
**Table S8**: Differential proteins between the TB0 group and the TB6 group identified by proteomics.
**Table S9**: Gene correspondence of differential proteins between the TB0 group and the TB6 group.
**Table S10**: Common DEGs between the TB6 vs. TB0 group and the TB0 vs. HC group.
**Table S11**: Enrichment analysis results of DEGs in the progression stage of pulmonary tuberculosis.
**Table S12**: Enrichment analysis results of DEGs in the cured stage of pulmonary tuberculosis.
**Table S13**: Enrichment analysis results of common DEGs between the TB6 vs. TB0 group and the TB0 vs. HC group.
**Table S14**: Enrichment analysis results of transcriptomics DEGs.
**Table S15**: GSVA enrichment analysis results.
**Table S16**: Details of the MRM method.
**Table S17**: Detailed parameters for liquid chromatography and mass spectrometry. Chromatographic separation was performed using HPLC  system Easy nLC‐1200 (Thermo Scientific), and mass spectrometry was performed using Q‐Exactive HF (Thermo Scientific). For liquid‐phase separation, buffer A was 0.1% aqueous solution of formic acid, and solution B was 0.1% aqueous solution of acetonitrile and formic acid (acetonitrile of 84%). Maxquant was used of database retrieval and Spectronaut was used of DIA data processing. DDA: Data dependent acquisition, DIA: Data‐independent acquisition.

## Data Availability

The data sets analyzed in this study were derived from the Gene Expression Omnibus database (https://www.ncbi.nlm.nih.gov/gds). The mass spectrometry proteomics data have been deposited to the ProteomeXchange Consortium via the iProX partner repository with the dataset identifier PXD071553. All other data generated and analyzed in this study are available from the corresponding authors upon reasonable request.
