# Supplementary material for: Robust Diagnostic and Therapeutic Biomarkers for Tuberculosis Identified Through Multi‐Omics and Mendelian Randomization Analysis
Source: MedComm (2020). 2025 Dec 29;7(1):e70559. doi: 10.1002/mco2.70559 (PMC12748937; doi:10.1002/mco2.70559)
Supplement: Supplementary file 1 — Figure S1 Analysis of 15 plasma metabolites causally associated with tuberculosis. Figure S2 MR analysis results for target metabolites. Scatter plots (A, E, I), funnel plots (B, F, J), forest plots (C, G, K), and leave‐one‐out sensitivity analysis plots (D, H, L). Figure S3 MR results for target plasma proteins. Scatter plots (A, E, I, M), funnel plots (B, F, J, N), forest plots (C, G, K, O), and leave‐one‐out sensitivity analysis plots (D, H, L, P). Figure S4 GO and KEGG enrichment analysis. (A) GO analysis of 39 common DEPs from HC, TB0, and TB6 groups. (B) KEGG analysis of 39 common DEPs from HC, TB0, and TB6 groups. The ordinate represent the enriched GO functional classification, which is divided into three major categories: Biological Process (BP), Molecular Function (MF), and Cellular Component (CC). The abscissas indicates the Ratio of differential proteins under each functional classification. The color gradient represents the size of the p value. The color of bubbles represents the p value, and the size of bubbles represents the number of counts. Figure S5 Heat map of differentially expressed genes in the transcriptome. Figure S6 GO and KEGG enrichment analysis. (A) GO analysis of DEGs (B) KEGG analysis of DEGs. The ordinate represent the enriched GO functional classification, which is divided into three major categories: Biological Process (BP), Molecular Function (MF), and Cellular Component (CC). The abscissas indicates the Ratio of differential proteins under each functional classification. The color gradient represents the size of the p value. The color of bubbles represents the p value, and the size of bubbles represents the number of counts. Figure S7 Gene Set Variation Analysis. (A) Distribution of GSVA scores for GO pathways showing significant differences between groups stratified by HP expression level. (B) Distribution of GSVA scores for KEGG pathways showing significant differences between groups stratified by HP expression level. The x‐axis [file MCO2-7-e70559-s001.docx]

**Robust Diagnostic and Therapeutic Biomarkers for Tuberculosis Identified through Multi-Omics and Mendelian Randomization Analysis**

Chenglin Zhu^1,#^, Jiaxi Chen^2,#^, Ying Li^1,#^, Qi Zhang^1^, Qiqi Lu^3^, Ningxuan Zhang^1^, Hao Fan^1^, Muhammad Mahtab Aslam Khan Khakwani^1^, Lei Zhang^1^,*, Ji-Cheng Li^1^,*
^1^ School of Basic Medical Sciences, Henan University, Kaifeng, 475004, Henan, China;

^2^ Clinical Laboratory, Taizhou Hospital of Zhejiang Province Affiliated to Wenzhou Medical University, 150 Ximen Street, Linhai, Zhejiang Province, 317000, People’s Republic of China;

^3^ Department of Clinical Laboratory, The Second Affiliated Hospital, Zhejiang University School of Medicine, Hangzhou, China;

^#^ These authors contributed equally to this work.

* Correspondence

Lei Zhang, School of Basic Medical Sciences, Henan University, Kaifeng, 475004, Henan, China. Email: [zhlei@henu.edu.cn](mailto:zhlei@henu.edu.cn); Ji-Cheng Li, School of Basic Medical Sciences, Henan University, Kaifeng, 475004, Henan, China. Emai: [zjulijicheng@163.com](mailto:zjulijicheng@163.com).

**
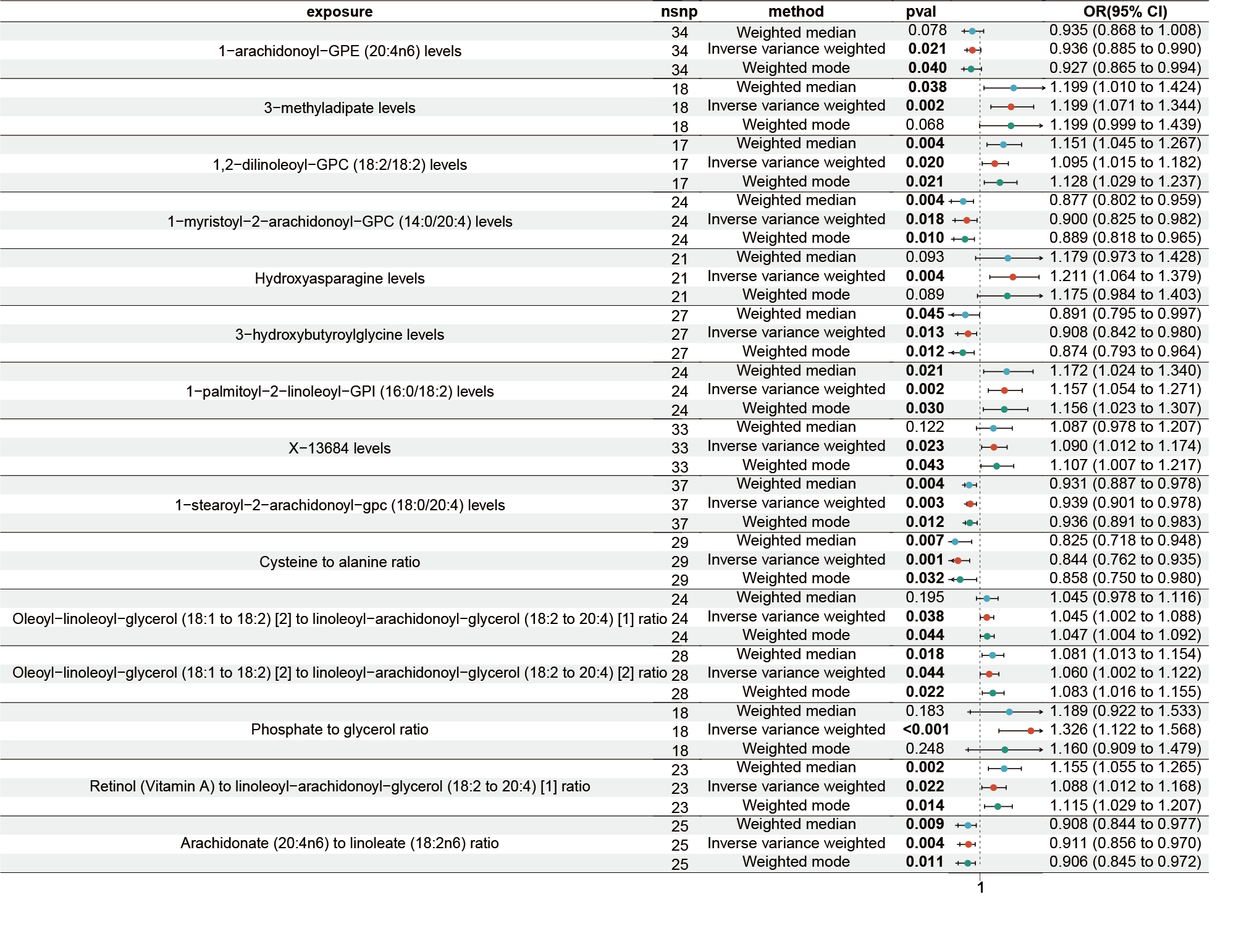
**

**Figure S1** Analysis of 15 plasma metabolites causally associated with tuberculosis.

**
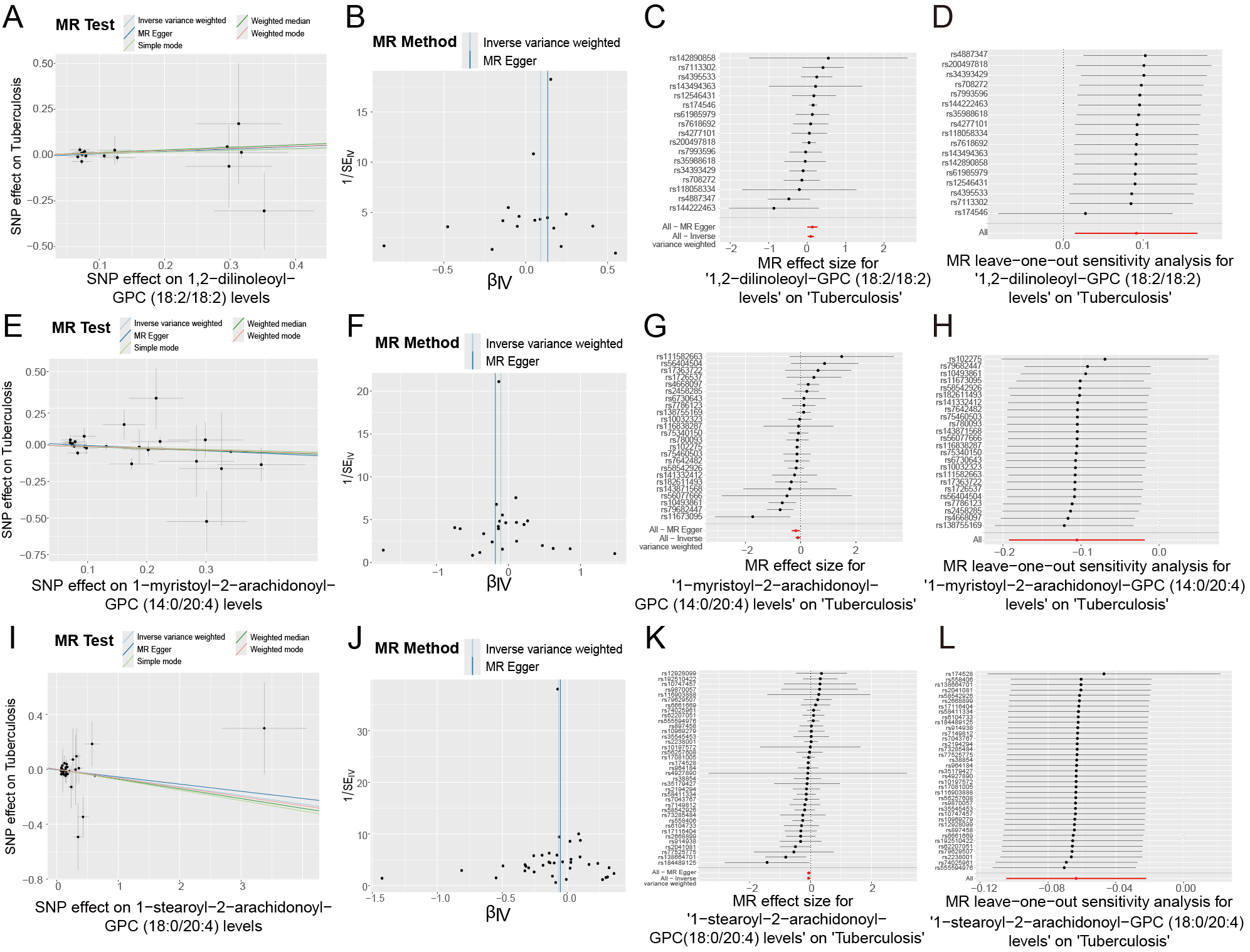
**

**Figure S2** MR analysis results for target metabolites. Scatter plots (A, E, I), funnel plots (B, F, J), forest plots (C, G, K), and leave-one-out sensitivity analysis plots (D, H, L).


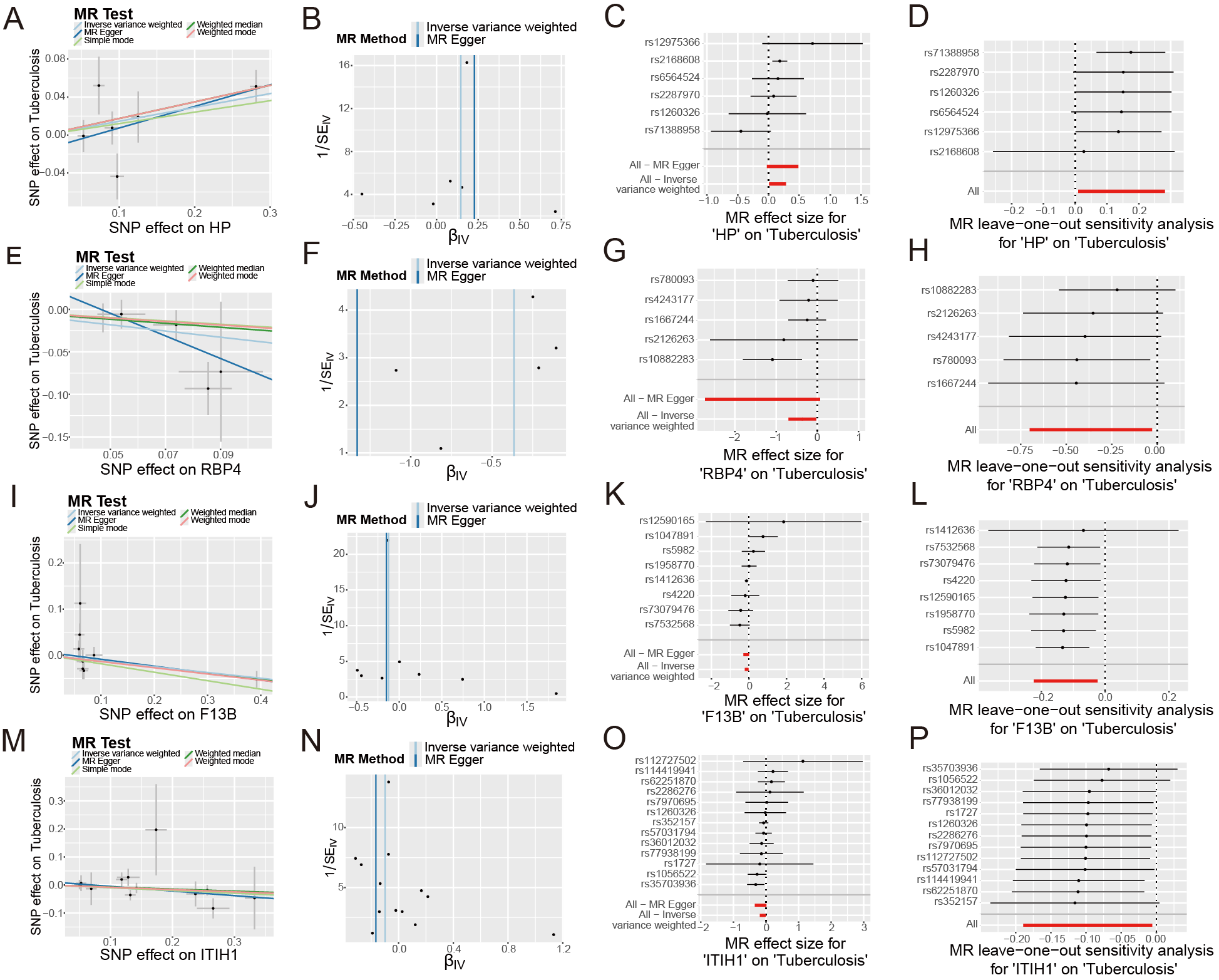


Figure S3 MR results for target plasma proteins. Scatter plots (A, E, I, M), funnel plots (B, F, J, N), forest plots (C, G, K, O), and leave-one-out sensitivity analysis plots (D, H, L, P).


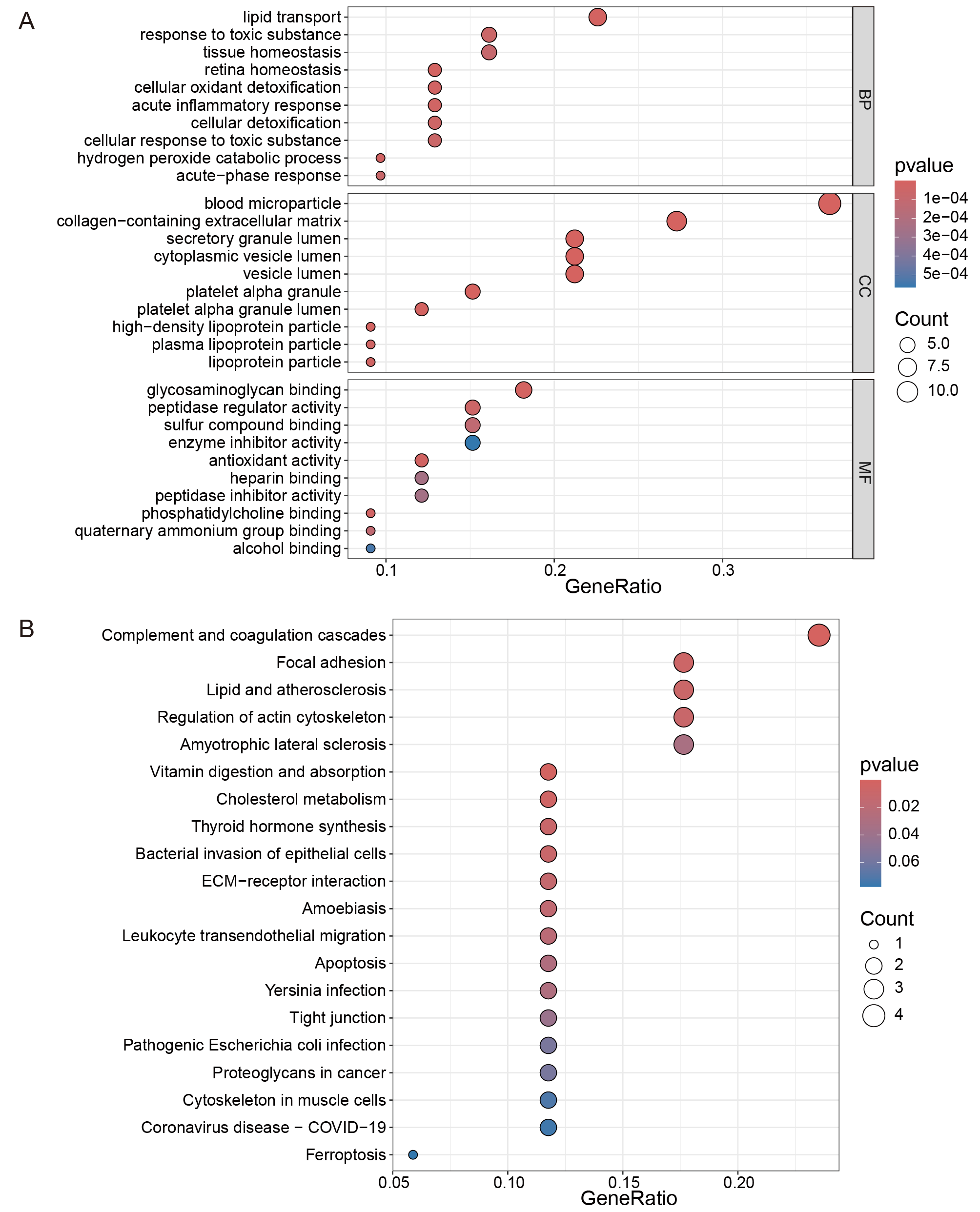


**Figure S4** GO and KEGG e**nrichment analysis.** (A) GO analysis of 39 common DEPs from HC, TB0, and TB6 groups. (B) KEGG analysis of 39 common DEPs from HC, TB0, and TB6 groups. The ordinate represent the enriched GO functional classification, which is divided into three major categories: Biological Process (BP), Molecular Function (MF), and Cellular Component (CC). The abscissas indicates the Ratio of differential proteins under each functional classification. The color gradient represents the size of the P value. The color of bubbles represents the P value, and the size of bubbles represents the number of counts.


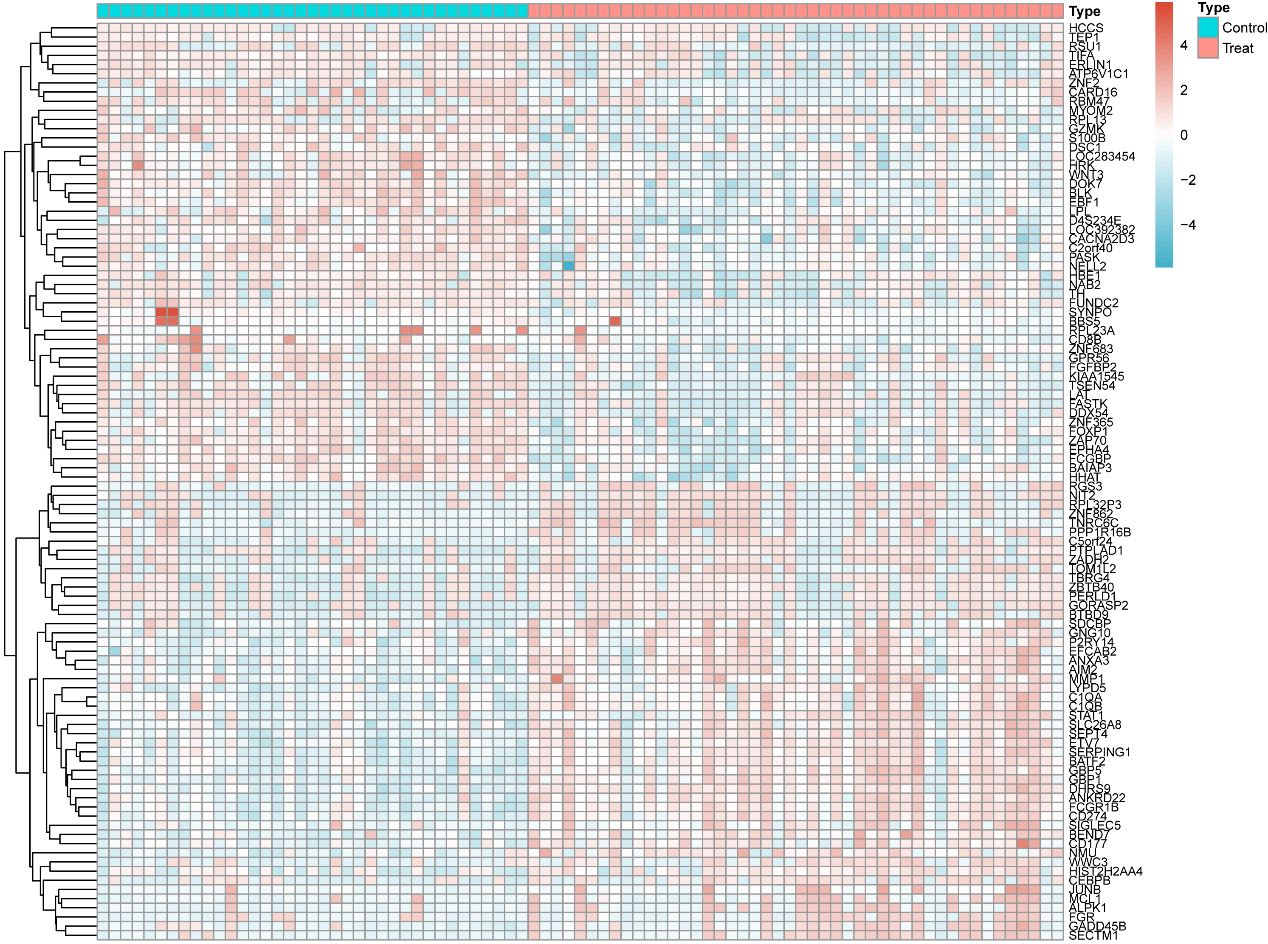


Figure S5 Heat map of differentially expressed genes in the transcriptome.


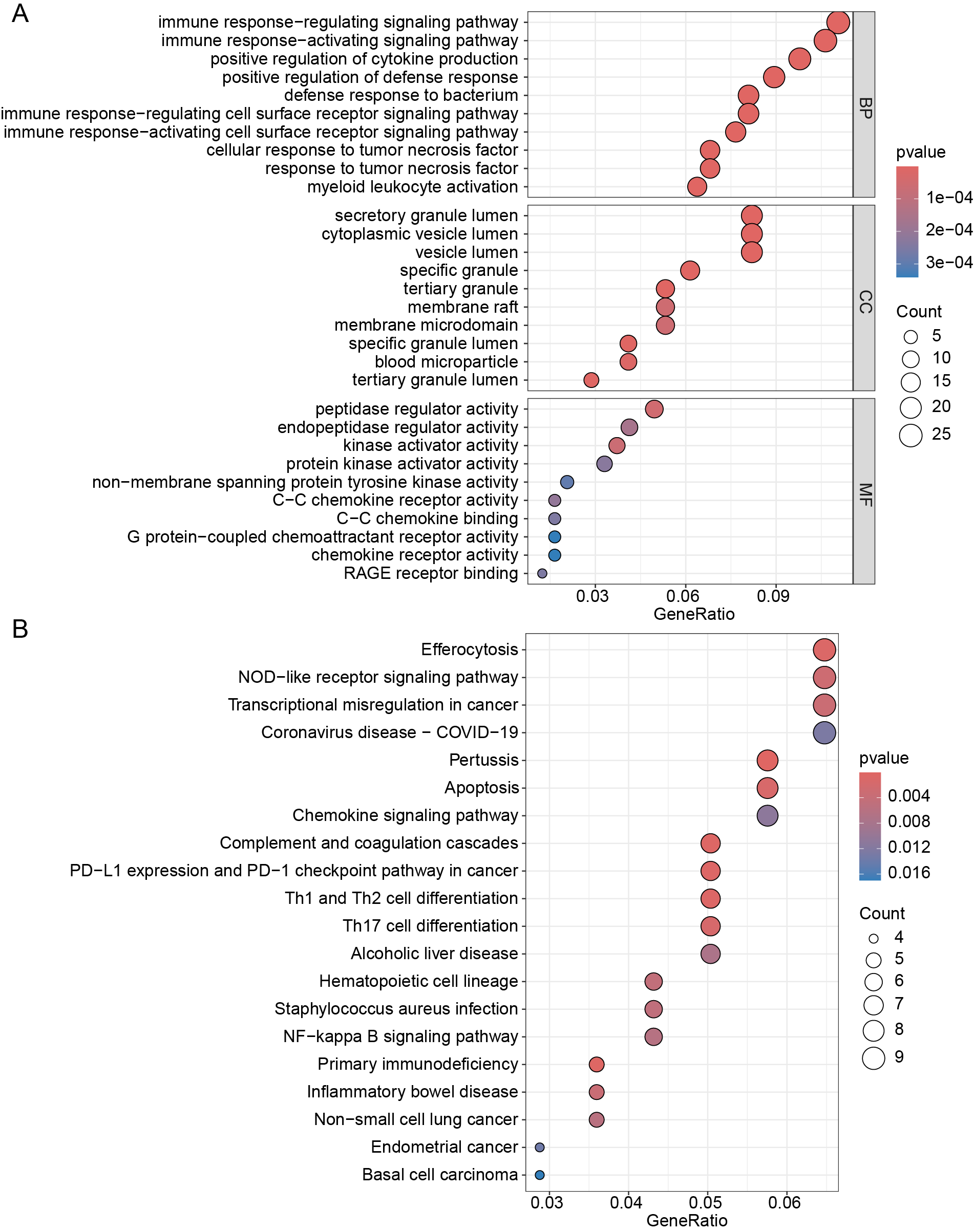


Figure S6 GO and KEGG e**nrichment analysis.** (A) GO analysis of DEGs (B) KEGG analysis of DEGs. The ordinate represent the enriched GO functional classification, which is divided into three major categories: Biological Process (BP), Molecular Function (MF), and Cellular Component (CC). The abscissas indicates the Ratio of differential proteins under each functional classification. The color gradient represents the size of the P value. The color of bubbles represents the P value, and the size of bubbles represents the number of counts.


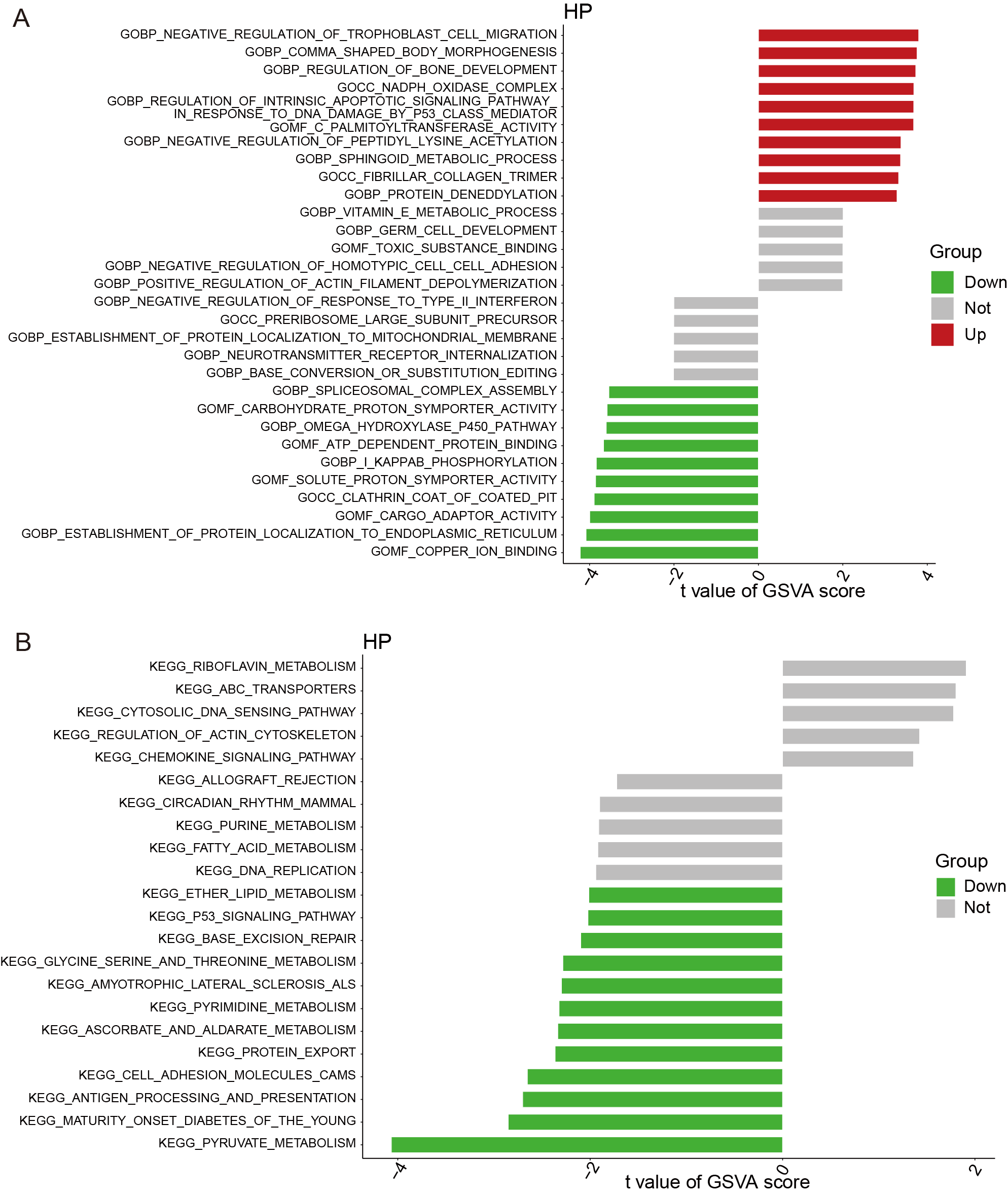


Figure S7 Gene Set Variation Analysis. (A) Distribution of GSVA scores for GO pathways showing significant differences between groups stratified by HP expression level. (B) Distribution of GSVA scores for KEGG pathways showing significant differences between groups stratified by HP expression level. The x-axis denotes the t value of the GSVA score and the y-axis lists pathway names.

**
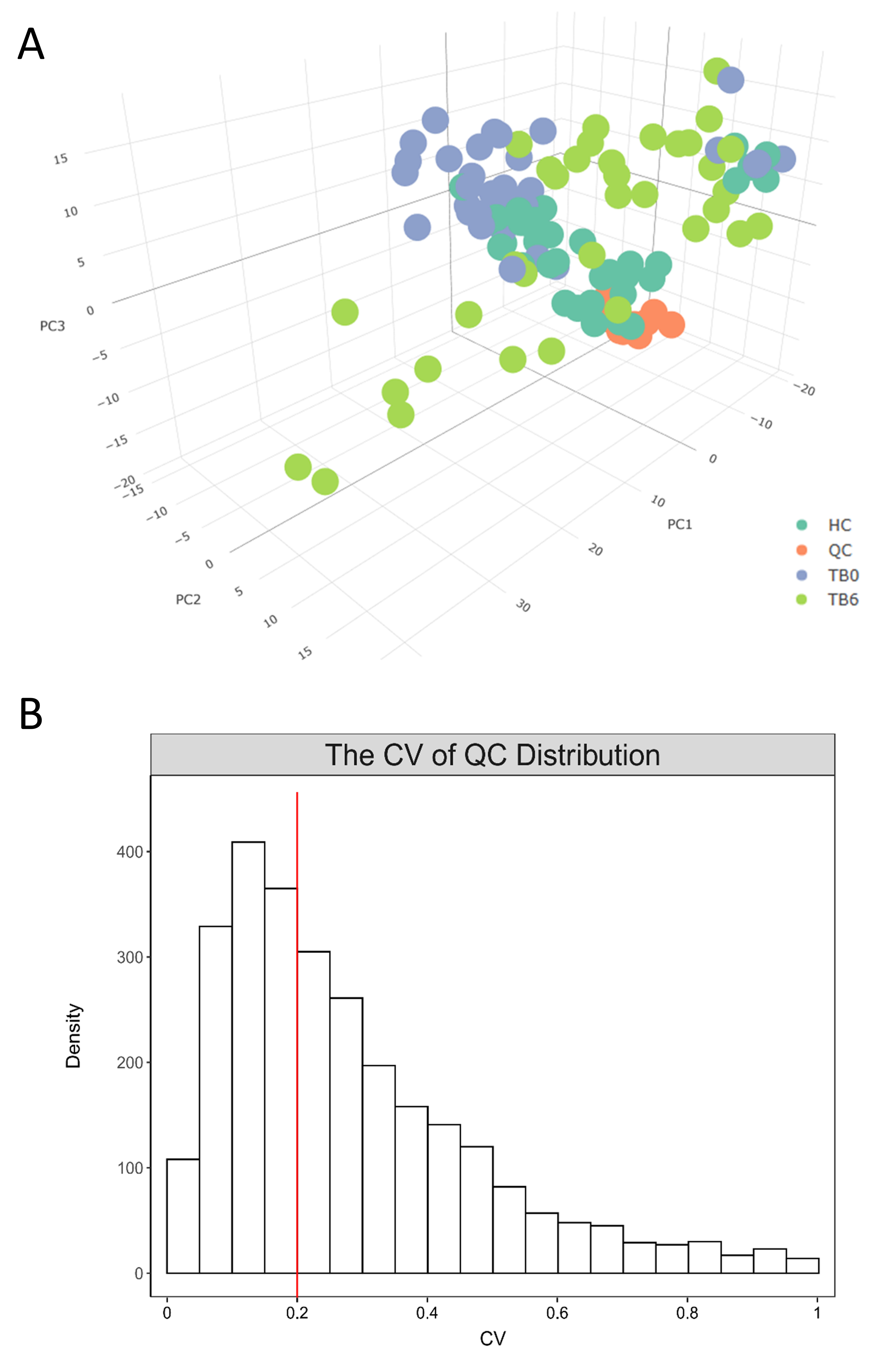
**

Figure S8 Quality control (A) Sample clustering based on the protein expression profile. A three-dimensional principal component analysis (PCA) scatter plot was generated from the expression levels of all quantified proteins to visualize clustering of healthy controls (HC), QC samples, and the two disease groups (TB0, TB6). QC samples cluster tightly, indicating high experimental stability and low technical variation; samples from different study groups exhibit clear separation trends, revealing distinct proteomic signatures and supporting the reliability of subsequent differential analyses. (B) Distribution of protein quantification CVs in QC samples. The histogram depicts the frequency distribution of CV values for all proteins measured in QC samples, providing an overall assessment of the precision and reproducibility of the proteomic quantification. The red vertical line denotes the median CV (20%), a key metric of assay stability. The majority of proteins show low CVs, indicating high-quality, reliable quantitative data.

Table S1: 15 plasma metabolites screened by Mendelian randomization analysis

| id.exposure | id.outcome | outcome | exposure | method | nsnp | pval | or | or_lci95 | or_uci95 |
| --- | --- | --- | --- | --- | --- | --- | --- | --- | --- |
| GCST90199791 | ebi-a-GCST90018892 | Tuberculosis | 1-arachidonoyl-GPE (20:4n6) levels | MR Egger | 37 | 1.92E-03 | 0.880 | 0.817 | 0.948 |
| GCST90199791 | ebi-a-GCST90018892 | Tuberculosis | 1-arachidonoyl-GPE (20:4n6) levels | Weighted median | 37 | 1.32E-03 | 0.912 | 0.863 | 0.965 |
| GCST90199791 | ebi-a-GCST90018892 | Tuberculosis | 1-arachidonoyl-GPE (20:4n6) levels | Inverse variance weighted | 37 | 2.60E-03 | 0.934 | 0.893 | 0.976 |
| GCST90199791 | ebi-a-GCST90018892 | Tuberculosis | 1-arachidonoyl-GPE (20:4n6) levels | Simple mode | 37 | 7.78E-01 | 0.982 | 0.869 | 1.110 |
| GCST90199791 | ebi-a-GCST90018892 | Tuberculosis | 1-arachidonoyl-GPE (20:4n6) levels | Weighted mode | 37 | 2.43E-03 | 0.919 | 0.874 | 0.967 |
| GCST90199829 | ebi-a-GCST90018892 | Tuberculosis | 3-methyladipate levels | MR Egger | 19 | 7.05E-01 | 1.054 | 0.808 | 1.374 |
| GCST90199829 | ebi-a-GCST90018892 | Tuberculosis | 3-methyladipate levels | Weighted median | 19 | 3.56E-02 | 1.210 | 1.013 | 1.445 |
| GCST90199829 | ebi-a-GCST90018892 | Tuberculosis | 3-methyladipate levels | Inverse variance weighted | 19 | 4.62E-04 | 1.219 | 1.091 | 1.361 |
| GCST90199829 | ebi-a-GCST90018892 | Tuberculosis | 3-methyladipate levels | Simple mode | 19 | 5.08E-01 | 1.104 | 0.829 | 1.471 |
| GCST90199829 | ebi-a-GCST90018892 | Tuberculosis | 3-methyladipate levels | Weighted mode | 19 | 7.08E-02 | 1.201 | 0.996 | 1.447 |
| GCST90200030 | ebi-a-GCST90018892 | Tuberculosis | 1,2-dilinoleoyl-GPC (18:2/18:2) levels | MR Egger | 20 | 1.23E-02 | 1.161 | 1.045 | 1.289 |
| GCST90200030 | ebi-a-GCST90018892 | Tuberculosis | 1,2-dilinoleoyl-GPC (18:2/18:2) levels | Weighted median | 20 | 1.99E-04 | 1.165 | 1.075 | 1.263 |
| GCST90200030 | ebi-a-GCST90018892 | Tuberculosis | 1,2-dilinoleoyl-GPC (18:2/18:2) levels | Inverse variance weighted | 20 | 2.66E-04 | 1.121 | 1.054 | 1.192 |
| GCST90200030 | ebi-a-GCST90018892 | Tuberculosis | 1,2-dilinoleoyl-GPC (18:2/18:2) levels | Simple mode | 20 | 1.42E-01 | 1.129 | 0.967 | 1.318 |
| GCST90200030 | ebi-a-GCST90018892 | Tuberculosis | 1,2-dilinoleoyl-GPC (18:2/18:2) levels | Weighted mode | 20 | 1.38E-03 | 1.148 | 1.068 | 1.233 |
| GCST90200078 | ebi-a-GCST90018892 | Tuberculosis | 1-myristoyl-2-arachidonoyl-GPC (14:0/20:4) levels | MR Egger | 29 | 1.64E-03 | 0.833 | 0.752 | 0.923 |
| GCST90200078 | ebi-a-GCST90018892 | Tuberculosis | 1-myristoyl-2-arachidonoyl-GPC (14:0/20:4) levels | Weighted median | 29 | 2.42E-04 | 0.875 | 0.815 | 0.940 |
| GCST90200078 | ebi-a-GCST90018892 | Tuberculosis | 1-myristoyl-2-arachidonoyl-GPC (14:0/20:4) levels | Inverse variance weighted | 29 | 4.25E-04 | 0.894 | 0.839 | 0.951 |
| GCST90200078 | ebi-a-GCST90018892 | Tuberculosis | 1-myristoyl-2-arachidonoyl-GPC (14:0/20:4) levels | Simple mode | 29 | 1.35E-01 | 0.888 | 0.764 | 1.033 |
| GCST90200078 | ebi-a-GCST90018892 | Tuberculosis | 1-myristoyl-2-arachidonoyl-GPC (14:0/20:4) levels | Weighted mode | 29 | 3.88E-04 | 0.881 | 0.829 | 0.937 |
| GCST90200168 | ebi-a-GCST90018892 | Tuberculosis | Hydroxyasparagine levels | MR Egger | 26 | 3.09E-01 | 1.268 | 0.810 | 1.986 |
| GCST90200168 | ebi-a-GCST90018892 | Tuberculosis | Hydroxyasparagine levels | Weighted median | 26 | 5.15E-02 | 1.180 | 0.999 | 1.393 |
| GCST90200168 | ebi-a-GCST90018892 | Tuberculosis | Hydroxyasparagine levels | Inverse variance weighted | 26 | 1.15E-03 | 1.207 | 1.078 | 1.352 |
| GCST90200168 | ebi-a-GCST90018892 | Tuberculosis | Hydroxyasparagine levels | Simple mode | 26 | 1.88E-01 | 1.182 | 0.928 | 1.506 |
| GCST90200168 | ebi-a-GCST90018892 | Tuberculosis | Hydroxyasparagine levels | Weighted mode | 26 | 1.02E-01 | 1.168 | 0.976 | 1.397 |
| GCST90200174 | ebi-a-GCST90018892 | Tuberculosis | 3-hydroxybutyroylglycine levels | MR Egger | 33 | 9.18E-02 | 0.896 | 0.791 | 1.014 |
| GCST90200174 | ebi-a-GCST90018892 | Tuberculosis | 3-hydroxybutyroylglycine levels | Weighted median | 33 | 9.08E-03 | 0.891 | 0.816 | 0.972 |
| GCST90200174 | ebi-a-GCST90018892 | Tuberculosis | 3-hydroxybutyroylglycine levels | Inverse variance weighted | 33 | 1.06E-03 | 0.899 | 0.843 | 0.958 |
| GCST90200174 | ebi-a-GCST90018892 | Tuberculosis | 3-hydroxybutyroylglycine levels | Simple mode | 33 | 6.74E-03 | 0.822 | 0.720 | 0.939 |
| GCST90200174 | ebi-a-GCST90018892 | Tuberculosis | 3-hydroxybutyroylglycine levels | Weighted mode | 33 | 3.03E-03 | 0.876 | 0.809 | 0.950 |
| GCST90200332 | ebi-a-GCST90018892 | Tuberculosis | 1-palmitoyl-2-linoleoyl-GPI (16:0/18:2) levels | MR Egger | 29 | 1.24E-01 | 1.169 | 0.964 | 1.417 |
| GCST90200332 | ebi-a-GCST90018892 | Tuberculosis | 1-palmitoyl-2-linoleoyl-GPI (16:0/18:2) levels | Weighted median | 29 | 4.18E-02 | 1.121 | 1.004 | 1.252 |
| GCST90200332 | ebi-a-GCST90018892 | Tuberculosis | 1-palmitoyl-2-linoleoyl-GPI (16:0/18:2) levels | Inverse variance weighted | 29 | 3.53E-04 | 1.146 | 1.063 | 1.234 |
| GCST90200332 | ebi-a-GCST90018892 | Tuberculosis | 1-palmitoyl-2-linoleoyl-GPI (16:0/18:2) levels | Simple mode | 29 | 9.72E-02 | 1.164 | 0.979 | 1.385 |
| GCST90200332 | ebi-a-GCST90018892 | Tuberculosis | 1-palmitoyl-2-linoleoyl-GPI (16:0/18:2) levels | Weighted mode | 29 | 2.45E-04 | 1.252 | 1.127 | 1.390 |
| GCST90200512 | ebi-a-GCST90018892 | Tuberculosis | X-13684 levels | MR Egger | 37 | 1.33E-02 | 1.231 | 1.053 | 1.440 |
| GCST90200512 | ebi-a-GCST90018892 | Tuberculosis | X-13684 levels | Weighted median | 37 | 1.92E-02 | 1.111 | 1.017 | 1.212 |
| GCST90200512 | ebi-a-GCST90018892 | Tuberculosis | X-13684 levels | Inverse variance weighted | 37 | 1.60E-03 | 1.108 | 1.040 | 1.180 |
| GCST90200512 | ebi-a-GCST90018892 | Tuberculosis | X-13684 levels | Simple mode | 37 | 1.57E-01 | 1.097 | 0.968 | 1.244 |
| GCST90200512 | ebi-a-GCST90018892 | Tuberculosis | X-13684 levels | Weighted mode | 37 | 2.04E-02 | 1.120 | 1.022 | 1.227 |
| GCST90200685 | ebi-a-GCST90018892 | Tuberculosis | 1-stearoyl-2-arachidonoyl-gpc (18:0/20:4) levels | MR Egger | 41 | 6.30E-02 | 0.945 | 0.892 | 1.001 |
| GCST90200685 | ebi-a-GCST90018892 | Tuberculosis | 1-stearoyl-2-arachidonoyl-gpc (18:0/20:4) levels | Weighted median | 41 | 5.14E-03 | 0.931 | 0.886 | 0.979 |
| GCST90200685 | ebi-a-GCST90018892 | Tuberculosis | 1-stearoyl-2-arachidonoyl-gpc (18:0/20:4) levels | Inverse variance weighted | 41 | 2.30E-03 | 0.939 | 0.902 | 0.978 |
| GCST90200685 | ebi-a-GCST90018892 | Tuberculosis | 1-stearoyl-2-arachidonoyl-gpc (18:0/20:4) levels | Simple mode | 41 | 2.30E-01 | 0.920 | 0.805 | 1.052 |
| GCST90200685 | ebi-a-GCST90018892 | Tuberculosis | 1-stearoyl-2-arachidonoyl-gpc (18:0/20:4) levels | Weighted mode | 41 | 1.91E-02 | 0.938 | 0.891 | 0.987 |
| GCST90200786 | ebi-a-GCST90018892 | Tuberculosis | Cysteine to alanine ratio | MR Egger | 29 | 1.94E-01 | 0.829 | 0.630 | 1.092 |
| GCST90200786 | ebi-a-GCST90018892 | Tuberculosis | Cysteine to alanine ratio | Weighted median | 29 | 7.00E-03 | 0.825 | 0.718 | 0.949 |
| GCST90200786 | ebi-a-GCST90018892 | Tuberculosis | Cysteine to alanine ratio | Inverse variance weighted | 29 | 1.19E-03 | 0.844 | 0.762 | 0.935 |
| GCST90200786 | ebi-a-GCST90018892 | Tuberculosis | Cysteine to alanine ratio | Simple mode | 29 | 1.41E-01 | 0.867 | 0.721 | 1.043 |
| GCST90200786 | ebi-a-GCST90018892 | Tuberculosis | Cysteine to alanine ratio | Weighted mode | 29 | 2.39E-02 | 0.858 | 0.756 | 0.973 |
| GCST90200794 | ebi-a-GCST90018892 | Tuberculosis | Oleoyl-linoleoyl-glycerol (18:1 to 18:2) [2] to linoleoyl-arachidonoyl-glycerol (18:2 to 20:4) [1] ratio | MR Egger | 28 | 3.66E-02 | 1.064 | 1.007 | 1.123 |
| GCST90200794 | ebi-a-GCST90018892 | Tuberculosis | Oleoyl-linoleoyl-glycerol (18:1 to 18:2) [2] to linoleoyl-arachidonoyl-glycerol (18:2 to 20:4) [1] ratio | Weighted median | 28 | 8.80E-04 | 1.084 | 1.034 | 1.136 |
| GCST90200794 | ebi-a-GCST90018892 | Tuberculosis | Oleoyl-linoleoyl-glycerol (18:1 to 18:2) [2] to linoleoyl-arachidonoyl-glycerol (18:2 to 20:4) [1] ratio | Inverse variance weighted | 28 | 1.54E-03 | 1.057 | 1.021 | 1.094 |
| GCST90200794 | ebi-a-GCST90018892 | Tuberculosis | Oleoyl-linoleoyl-glycerol (18:1 to 18:2) [2] to linoleoyl-arachidonoyl-glycerol (18:2 to 20:4) [1] ratio | Simple mode | 28 | 3.28E-01 | 1.083 | 0.926 | 1.266 |
| GCST90200794 | ebi-a-GCST90018892 | Tuberculosis | Oleoyl-linoleoyl-glycerol (18:1 to 18:2) [2] to linoleoyl-arachidonoyl-glycerol (18:2 to 20:4) [1] ratio | Weighted mode | 28 | 3.15E-03 | 1.061 | 1.024 | 1.100 |
| GCST90200795 | ebi-a-GCST90018892 | Tuberculosis | Oleoyl-linoleoyl-glycerol (18:1 to 18:2) [2] to linoleoyl-arachidonoyl-glycerol (18:2 to 20:4) [2] ratio | MR Egger | 34 | 1.88E-03 | 1.117 | 1.048 | 1.190 |
| GCST90200795 | ebi-a-GCST90018892 | Tuberculosis | Oleoyl-linoleoyl-glycerol (18:1 to 18:2) [2] to linoleoyl-arachidonoyl-glycerol (18:2 to 20:4) [2] ratio | Weighted median | 34 | 1.43E-04 | 1.105 | 1.049 | 1.163 |
| GCST90200795 | ebi-a-GCST90018892 | Tuberculosis | Oleoyl-linoleoyl-glycerol (18:1 to 18:2) [2] to linoleoyl-arachidonoyl-glycerol (18:2 to 20:4) [2] ratio | Inverse variance weighted | 34 | 1.91E-03 | 1.069 | 1.025 | 1.116 |
| GCST90200795 | ebi-a-GCST90018892 | Tuberculosis | Oleoyl-linoleoyl-glycerol (18:1 to 18:2) [2] to linoleoyl-arachidonoyl-glycerol (18:2 to 20:4) [2] ratio | Simple mode | 34 | 6.29E-01 | 1.031 | 0.912 | 1.165 |
| GCST90200795 | ebi-a-GCST90018892 | Tuberculosis | Oleoyl-linoleoyl-glycerol (18:1 to 18:2) [2] to linoleoyl-arachidonoyl-glycerol (18:2 to 20:4) [2] ratio | Weighted mode | 34 | 1.55E-03 | 1.087 | 1.037 | 1.139 |
| GCST90200903 | ebi-a-GCST90018892 | Tuberculosis | Phosphate to glycerol ratio | MR Egger | 19 | 3.61E-01 | 1.303 | 0.749 | 2.267 |
| GCST90200903 | ebi-a-GCST90018892 | Tuberculosis | Phosphate to glycerol ratio | Weighted median | 19 | 1.91E-01 | 1.177 | 0.922 | 1.502 |
| GCST90200903 | ebi-a-GCST90018892 | Tuberculosis | Phosphate to glycerol ratio | Inverse variance weighted | 19 | 1.19E-03 | 1.317 | 1.115 | 1.555 |
| GCST90200903 | ebi-a-GCST90018892 | Tuberculosis | Phosphate to glycerol ratio | Simple mode | 19 | 7.71E-02 | 1.412 | 0.984 | 2.026 |
| GCST90200903 | ebi-a-GCST90018892 | Tuberculosis | Phosphate to glycerol ratio | Weighted mode | 19 | 3.42E-01 | 1.135 | 0.880 | 1.463 |
| GCST90200907 | ebi-a-GCST90018892 | Tuberculosis | Retinol (Vitamin A) to linoleoyl-arachidonoyl-glycerol (18:2 to 20:4) [1] ratio | MR Egger | 29 | 4.63E-03 | 1.221 | 1.076 | 1.386 |
| GCST90200907 | ebi-a-GCST90018892 | Tuberculosis | Retinol (Vitamin A) to linoleoyl-arachidonoyl-glycerol (18:2 to 20:4) [1] ratio | Weighted median | 29 | 1.15E-04 | 1.161 | 1.076 | 1.253 |
| GCST90200907 | ebi-a-GCST90018892 | Tuberculosis | Retinol (Vitamin A) to linoleoyl-arachidonoyl-glycerol (18:2 to 20:4) [1] ratio | Inverse variance weighted | 29 | 2.81E-04 | 1.106 | 1.048 | 1.168 |
| GCST90200907 | ebi-a-GCST90018892 | Tuberculosis | Retinol (Vitamin A) to linoleoyl-arachidonoyl-glycerol (18:2 to 20:4) [1] ratio | Simple mode | 29 | 7.71E-02 | 1.095 | 0.994 | 1.207 |
| GCST90200907 | ebi-a-GCST90018892 | Tuberculosis | Retinol (Vitamin A) to linoleoyl-arachidonoyl-glycerol (18:2 to 20:4) [1] ratio | Weighted mode | 29 | 1.11E-03 | 1.129 | 1.057 | 1.205 |
| GCST90200979 | ebi-a-GCST90018892 | Tuberculosis | Arachidonate (20:4n6) to linoleate (18:2n6) ratio | MR Egger | 26 | 1.03E-02 | 0.896 | 0.829 | 0.968 |
| GCST90200979 | ebi-a-GCST90018892 | Tuberculosis | Arachidonate (20:4n6) to linoleate (18:2n6) ratio | Weighted median | 26 | 1.80E-04 | 0.895 | 0.844 | 0.948 |
| GCST90200979 | ebi-a-GCST90018892 | Tuberculosis | Arachidonate (20:4n6) to linoleate (18:2n6) ratio | Inverse variance weighted | 26 | 5.17E-05 | 0.905 | 0.862 | 0.950 |
| GCST90200979 | ebi-a-GCST90018892 | Tuberculosis | Arachidonate (20:4n6) to linoleate (18:2n6) ratio | Simple mode | 26 | 2.33E-01 | 0.920 | 0.804 | 1.052 |
| GCST90200979 | ebi-a-GCST90018892 | Tuberculosis | Arachidonate (20:4n6) to linoleate (18:2n6) ratio | Weighted mode | 26 | 1.02E-03 | 0.901 | 0.853 | 0.952 |

Table S2: Differential lipids between the HC group and the TB0 group identified by lipidomics

| Index | Compounds | Class | VIP | Fold_Change | Log2FC | Type |
| --- | --- | --- | --- | --- | --- | --- |
| LIPID-N-0065 | TXB2 | Eicosanoid | 1.043 | 0.688 | -0.539 | down |
| LIPID-N-0080 | FFA(14:0) | FFA | 1.169 | 0.795 | -0.331 | down |
| LIPID-N-0083 | FFA(17:0) | FFA | 1.057 | 0.826 | -0.275 | down |
| LIPID-N-0089 | FFA(14:1) | FFA | 1.708 | 0.488 | -1.036 | down |
| LIPID-N-0090 | FFA(15:1) | FFA | 1.343 | 0.635 | -0.654 | down |
| LIPID-N-0098 | FFA(16:2) | FFA | 1.381 | 0.682 | -0.552 | down |
| LIPID-N-0099 | FFA(18:2) | FFA | 1.419 | 0.738 | -0.438 | down |
| LIPID-N-0108 | FFA(22:4) | FFA | 1.272 | 0.709 | -0.496 | down |
| LIPID-N-0109 | FFA(24:4) | FFA | 1.661 | 0.800 | -0.321 | down |
| LIPID-N-0113 | FFA(22:6) | FFA | 1.695 | 0.552 | -0.859 | down |
| LIPID-N-0115 | LPA(0:0/16:0) | LPA | 2.979 | 3.898 | 1.963 | up |
| LIPID-N-0116 | LPA(0:0/18:0) | LPA | 3.194 | 5.213 | 2.382 | up |
| LIPID-N-0121 | LPC(20:0/0:0) | LPC | 1.540 | 0.767 | -0.383 | down |
| LIPID-N-0129 | LPC(18:2/0:0) | LPC | 1.746 | 0.715 | -0.484 | down |
| LIPID-N-0142 | LPE(0:0/20:0) | LPE | 1.642 | 0.690 | -0.534 | down |
| LIPID-N-0147 | LPE(0:0/20:1) | LPE | 1.047 | 0.785 | -0.349 | down |
| LIPID-N-0149 | LPE(0:0/20:2) | LPE | 1.077 | 0.748 | -0.419 | down |
| LIPID-N-0156 | LPE(0:0/22:6) | LPE | 1.323 | 0.759 | -0.397 | down |
| LIPID-N-0214 | PC(14:0/18:2) | PC | 1.559 | 0.699 | -0.516 | down |
| LIPID-N-0219 | PC(16:0/18:2) | PC | 1.792 | 0.810 | -0.304 | down |
| LIPID-N-0276 | PC(14:0/20:4) | PC | 1.017 | 0.743 | -0.428 | down |
| LIPID-N-0285 | PC(18:2/20:2) | PC | 1.052 | 0.753 | -0.409 | down |
| LIPID-N-0308 | PC(18:2/18:3) | PC | 1.100 | 0.790 | -0.339 | down |
| LIPID-N-0373 | PE(16:1/16:0) | PE | 1.264 | 2.283 | 1.191 | up |
| LIPID-N-0377 | PE(18:1/16:0) | PE | 1.451 | 1.728 | 0.789 | up |
| LIPID-N-0447 | PE(16:0/20:3) | PE | 1.557 | 1.896 | 0.923 | up |
| LIPID-N-0477 | PE(20:4/18:0) | PE | 1.213 | 1.407 | 0.493 | up |
| LIPID-N-0686 | PS(18:0/18:2) | PS | 1.058 | 0.748 | -0.418 | down |
| LIPID-P-0010 | Octanoyl-carnitine | CAR | 2.019 | 0.431 | -1.214 | down |
| LIPID-P-0013 | Decanoyl-carnitine | CAR | 1.908 | 0.430 | -1.216 | down |
| LIPID-P-0015 | Lauroyl-carnitine | CAR | 1.669 | 0.537 | -0.898 | down |
| LIPID-P-0017 | Myristoyl-carnitine | CAR | 1.188 | 0.788 | -0.344 | down |
| LIPID-P-0029 | Decenoyl-carnitine | CAR | 2.099 | 0.458 | -1.126 | down |
| LIPID-P-0031 | Dodecenoyl-carnitine | CAR | 1.210 | 0.695 | -0.525 | down |
| LIPID-P-0040 | Tetradecadienoyl- carnitine | CAR | 1.323 | 0.679 | -0.558 | down |
| LIPID-P-0041 | Palmitodileoyl- carnitine | CAR | 1.459 | 0.719 | -0.475 | down |
| LIPID-P-0042 | Linoleyl-carnitine | CAR | 1.800 | 0.737 | -0.440 | down |
| LIPID-P-0053 | CE(18:2) | CE | 2.013 | 0.758 | -0.401 | down |
| LIPID-P-0062 | CE(22:6) | CE | 1.457 | 0.657 | -0.605 | down |
| LIPID-P-0067 | Cer(d18:0/24:0) | Cer | 1.402 | 0.785 | -0.349 | down |
| LIPID-P-0069 | Cer(d18:1/18:0) | Cer | 1.571 | 1.591 | 0.670 | up |
| LIPID-P-0070 | Cer(d18:1/20:0) | Cer | 1.074 | 1.345 | 0.427 | up |
| LIPID-P-0072 | Cer(d18:1/24:0) | Cer | 1.543 | 0.765 | -0.387 | down |
| LIPID-P-0090 | Cer(m18:1/22:0) | Cerm | 1.059 | 1.525 | 0.609 | up |
| LIPID-P-0099 | CerP(d18:1/18:0) | CerP | 1.711 | 0.696 | -0.524 | down |
| LIPID-P-0108 | CerP(d18:1/20:3) | CerP | 1.170 | 0.744 | -0.427 | down |
| LIPID-P-0111 | Cer(t18:0/22:0) | Cert | 1.496 | 0.661 | -0.598 | down |
| LIPID-P-0112 | Cer(t18:0/24:0) | Cert | 1.986 | 0.647 | -0.629 | down |
| LIPID-P-0116 | Coenzyme Q10 | COQ | 1.267 | 0.713 | -0.487 | down |
| LIPID-P-0126 | DG(16:0/18:0/0:0) | DG | 1.935 | 1.203 | 0.267 | up |
| LIPID-P-0127 | DG(16:0/20:0/0:0) | DG | 2.002 | 1.411 | 0.497 | up |
| LIPID-P-0128 | DG(14:0/22:0/0:0) | DG | 1.853 | 1.312 | 0.391 | up |
| LIPID-P-0129 | DG(18:0/18:0/0:0) | DG | 2.376 | 1.393 | 0.478 | up |
| LIPID-P-0194 | DG(18:2/20:2/0:0) | DG | 1.044 | 0.772 | -0.373 | down |
| LIPID-P-0227 | LPC(22:0/0:0) | LPC | 2.031 | 0.641 | -0.641 | down |
| LIPID-P-0228 | LPC(24:0/0:0) | LPC | 1.659 | 0.751 | -0.413 | down |
| LIPID-P-0232 | LPC(20:1/0:0) | LPC | 1.173 | 0.820 | -0.287 | down |
| LIPID-P-0243 | LPC(22:6/0:0) | LPC | 1.723 | 0.641 | -0.643 | down |
| LIPID-P-0245 | LPC(O-16:0/0:0) | LPC-O | 1.097 | 0.821 | -0.284 | down |
| LIPID-P-0266 | LPE(0:0/22:0) | LPE | 2.068 | 0.684 | -0.549 | down |
| LIPID-P-0274 | LPE(0:0/18:2) | LPE | 1.049 | 0.817 | -0.291 | down |
| LIPID-P-0284 | LPE(0:0/24:6) | LPE | 1.377 | 0.740 | -0.434 | down |
| LIPID-P-0293 | MG(18:0/0:0/0:0) | MG | 2.949 | 1.289 | 0.367 | up |
| LIPID-P-0362 | PC(18:2/22:0) | PC | 1.720 | 0.632 | -0.663 | down |
| LIPID-P-0369 | PC(16:1/18:2) | PC | 1.867 | 0.671 | -0.575 | down |
| LIPID-P-0371 | PC(14:0/20:3) | PC | 1.408 | 0.788 | -0.343 | down |
| LIPID-P-0376 | PC(18:1/18:2) | PC | 1.704 | 0.786 | -0.347 | down |
| LIPID-P-0394 | PC(18:3/14:1) | PC | 1.378 | 0.770 | -0.378 | down |
| LIPID-P-0402 | PC(18:2/18:2) | PC | 1.797 | 0.625 | -0.678 | down |
| LIPID-P-0422 | PC(18:0/22:4) | PC | 1.224 | 1.407 | 0.492 | up |
| LIPID-P-0465 | PC(14:0/22:6) | PC | 1.915 | 0.479 | -1.062 | down |
| LIPID-P-0473 | PC(16:0/22:6) | PC | 2.018 | 0.662 | -0.596 | down |
| LIPID-P-0479 | PC(18:0/22:6) | PC | 1.106 | 0.797 | -0.328 | down |
| LIPID-P-0480 | PC(20:1/20:5) | PC | 1.089 | 0.792 | -0.337 | down |
| LIPID-P-0489 | PC(16:1/22:6) | PC | 1.794 | 0.602 | -0.731 | down |
| LIPID-P-0496 | PC(18:1/22:6) | PC | 2.106 | 0.617 | -0.697 | down |
| LIPID-P-0500 | PC(20:1/22:6) | PC | 2.022 | 0.562 | -0.831 | down |
| LIPID-P-0504 | PC(18:2/22:6) | PC | 1.984 | 0.658 | -0.605 | down |
| LIPID-P-0507 | PC(20:2/22:6) | PC | 1.183 | 0.802 | -0.317 | down |
| LIPID-P-0513 | PC(O-16:0/14:0) | PC-O | 1.672 | 0.678 | -0.561 | down |
| LIPID-P-0523 | PC(O-18:0/18:1) | PC-O | 1.112 | 0.815 | -0.294 | down |
| LIPID-P-0528 | PC(O-16:0/14:2) | PC-O | 1.323 | 0.795 | -0.331 | down |
| LIPID-P-0529 | PC(O-16:0/16:2) | PC-O | 1.123 | 0.829 | -0.271 | down |
| LIPID-P-0530 | PC(O-16:0/18:2) | PC-O | 1.274 | 0.752 | -0.412 | down |
| LIPID-P-0532 | PC(O-18:1/20:1) | PC-O | 1.421 | 0.767 | -0.383 | down |
| LIPID-P-0533 | PC(O-20:1/20:1) | PC-O | 1.670 | 1.502 | 0.587 | up |
| LIPID-P-0535 | PC(O-16:2/18:1) | PC-O | 2.055 | 0.687 | -0.541 | down |
| LIPID-P-0537 | PC(O-18:2/20:1) | PC-O | 1.413 | 0.809 | -0.306 | down |
| LIPID-P-0539 | PC(O-20:2/22:1) | PC-O | 1.262 | 0.822 | -0.283 | down |
| LIPID-P-0540 | PC(O-16:2/18:2) | PC-O | 1.448 | 0.534 | -0.904 | down |
| LIPID-P-0546 | PC(O-16:2/18:3) | PC-O | 1.194 | 0.526 | -0.927 | down |
| LIPID-P-0552 | PC(O-18:3/18:3) | PC-O | 1.207 | 0.707 | -0.500 | down |
| LIPID-P-0553 | PC(O-18:3/20:3) | PC-O | 1.667 | 0.747 | -0.420 | down |
| LIPID-P-0554 | PC(O-20:3/20:3) | PC-O | 1.770 | 0.628 | -0.670 | down |
| LIPID-P-0557 | PC(O-18:3/18:4) | PC-O | 1.810 | 0.679 | -0.559 | down |
| LIPID-P-0558 | PC(O-18:3/20:4) | PC-O | 1.805 | 0.691 | -0.533 | down |
| LIPID-P-0559 | PC(O-20:3/20:4) | PC-O | 1.575 | 0.723 | -0.467 | down |
| LIPID-P-0562 | PC(O-20:4/20:4) | PC-O | 1.919 | 0.643 | -0.638 | down |
| LIPID-P-0569 | PE(16:0/18:0) | PE | 1.231 | 1.313 | 0.393 | up |
| LIPID-P-0577 | PE(16:1/18:0) | PE | 1.331 | 1.697 | 0.763 | up |
| LIPID-P-0580 | PE(18:1/18:0) | PE | 1.038 | 1.470 | 0.556 | up |
| LIPID-P-0588 | PE(22:2/12:0) | PE | 1.551 | 1.840 | 0.880 | up |
| LIPID-P-0589 | PE(16:0/18:2) | PE | 1.704 | 1.613 | 0.690 | up |
| LIPID-P-0594 | PE(18:1/18:1) | PE | 1.037 | 1.398 | 0.483 | up |
| LIPID-P-0605 | PE(20:3/18:0) | PE | 1.126 | 1.393 | 0.478 | up |
| LIPID-P-0612 | PE(16:0/20:4) | PE | 1.538 | 1.495 | 0.580 | up |
| LIPID-P-0616 | PE(18:0/20:4) | PE | 1.326 | 1.425 | 0.511 | up |
| LIPID-P-0617 | PE(18:0/22:4) | PE | 1.345 | 1.799 | 0.847 | up |
| LIPID-P-0622 | PE(20:1/20:4) | PE | 1.332 | 1.580 | 0.660 | up |
| LIPID-P-0637 | PE(20:3/20:5) | PE | 1.117 | 0.829 | -0.270 | down |
| LIPID-P-0638 | PE(P-18:0/16:0) | PE-P | 1.014 | 0.823 | -0.281 | down |
| LIPID-P-0642 | PE(P-18:1/16:0) | PE-P | 1.077 | 0.730 | -0.454 | down |
| LIPID-P-0643 | PE(P-18:1/18:0) | PE-P | 1.285 | 0.661 | -0.597 | down |
| LIPID-P-0645 | PE(P-20:1/20:0) | PE-P | 1.867 | 0.691 | -0.533 | down |
| LIPID-P-0646 | PE(P-18:2/16:0) | PE-P | 1.354 | 0.752 | -0.412 | down |
| LIPID-P-0647 | PE(P-18:2/18:0) | PE-P | 1.474 | 0.715 | -0.483 | down |
| LIPID-P-0653 | PE(P-18:2/20:1) | PE-P | 1.076 | 0.706 | -0.503 | down |
| LIPID-P-0657 | PE(P-18:2/18:2) | PE-P | 1.294 | 0.781 | -0.357 | down |
| LIPID-P-0661 | PE(P-18:2/18:3) | PE-P | 1.176 | 0.722 | -0.470 | down |
| LIPID-P-0662 | PE(P-18:2/20:3) | PE-P | 1.423 | 0.734 | -0.445 | down |
| LIPID-P-0663 | PE(P-20:2/20:3) | PE-P | 1.748 | 0.584 | -0.777 | down |
| LIPID-P-0664 | PE(P-20:2/22:3) | PE-P | 1.754 | 0.652 | -0.618 | down |
| LIPID-P-0665 | PE(P-20:2/24:3) | PE-P | 1.496 | 0.765 | -0.386 | down |
| LIPID-P-0666 | PE(P-18:2/20:4) | PE-P | 1.801 | 0.675 | -0.566 | down |
| LIPID-P-0667 | PE(P-20:2/20:4) | PE-P | 1.588 | 0.638 | -0.648 | down |
| LIPID-P-0670 | PE(P-20:2/20:5) | PE-P | 1.783 | 0.675 | -0.568 | down |
| LIPID-P-0671 | PE(P-20:2/22:5) | PE-P | 1.447 | 0.614 | -0.703 | down |
| LIPID-P-0673 | PE(P-20:2/20:6) | PE-P | 1.623 | 0.679 | -0.558 | down |
| LIPID-P-0675 | PE(P-20:2/24:6) | PE-P | 1.570 | 0.613 | -0.707 | down |
| LIPID-P-0705 | PS(18:0/20:4) | PS | 1.036 | 0.609 | -0.715 | down |
| LIPID-P-0716 | SM(d18:0/12:0) | SM | 2.582 | 0.773 | -0.372 | down |
| LIPID-P-0719 | SM(d18:0/18:0) | SM | 1.007 | 1.447 | 0.533 | up |
| LIPID-P-0724 | SM(d18:1/12:0) | SM | 1.582 | 0.643 | -0.637 | down |
| LIPID-P-0725 | SM(d18:1/14:0) | SM | 1.509 | 0.802 | -0.318 | down |
| LIPID-P-0727 | SM(d18:0/18:1) | SM | 1.526 | 0.632 | -0.663 | down |
| LIPID-P-0732 | SM(d20:0/24:1) | SM | 1.270 | 0.828 | -0.272 | down |
| LIPID-P-0734 | SM(d18:2/14:0) | SM | 1.439 | 0.767 | -0.382 | down |
| LIPID-P-0738 | SM(d18:1/22:1) | SM | 1.256 | 0.819 | -0.288 | down |
| LIPID-P-0741 | SM(d18:2/16:1) | SM | 1.776 | 0.713 | -0.489 | down |
| LIPID-P-0742 | SM(d18:2/18:1) | SM | 1.380 | 0.819 | -0.288 | down |
| LIPID-P-0760 | TG(14:0/18:0/20:0) | TG | 1.023 | 1.510 | 0.595 | up |
| LIPID-P-0897 | TG(12:0/18:0/18:3) | TG | 1.139 | 0.748 | -0.420 | down |
| LIPID-P-0971 | TG(12:0/18:2/18:2) | TG | 1.350 | 0.706 | -0.503 | down |
| LIPID-P-0980 | TG(14:0/18:1/18:3) | TG | 1.333 | 0.712 | -0.490 | down |
| LIPID-P-1034 | TG(14:0/18:2/18:3) | TG | 1.029 | 0.815 | -0.294 | down |
| LIPID-P-1040 | TG(16:1/16:1/20:3) | TG | 1.428 | 0.733 | -0.447 | down |
| LIPID-P-1050 | TG(18:1/18:2/18:2) | TG | 1.156 | 0.817 | -0.292 | down |
| LIPID-P-1083 | TG(14:0/18:2/20:4) | TG | 1.488 | 0.692 | -0.532 | down |
| LIPID-P-1086 | TG(16:1/18:1/18:4) | TG | 1.132 | 0.802 | -0.318 | down |
| LIPID-P-1093 | TG(18:2/18:2/18:2) | TG | 1.174 | 0.786 | -0.347 | down |
| LIPID-P-1094 | TG(18:1/18:2/18:3) | TG | 1.074 | 0.811 | -0.302 | down |
| LIPID-P-1095 | TG(14:0/18:3/22:3) | TG | 1.020 | 0.823 | -0.280 | down |
| LIPID-P-1124 | TG(14:0/16:1/22:6) | TG | 1.081 | 0.497 | -1.009 | down |
| LIPID-P-1125 | TG(18:2/18:2/18:3) | TG | 1.097 | 0.827 | -0.275 | down |
| LIPID-P-1158 | TG(14:0/18:2/22:6) | TG | 1.430 | 0.560 | -0.836 | down |
| LIPID-P-1164 | TG(18:2/18:3/20:3) | TG | 1.353 | 0.617 | -0.696 | down |
| LIPID-P-1165 | TG(16:0/18:3/22:5) | TG | 1.223 | 0.665 | -0.588 | down |
| LIPID-P-1166 | TG(14:0/20:2/22:6) | TG | 1.251 | 0.655 | -0.609 | down |
| LIPID-P-1180 | TG(16:1/20:4/20:4) | TG | 1.515 | 0.599 | -0.739 | down |
| LIPID-P-1182 | TG(14:0/20:3/22:6) | TG | 1.430 | 0.599 | -0.740 | down |
| LIPID-P-1185 | TG(14:0/22:3/22:6) | TG | 1.316 | 0.645 | -0.633 | down |
| LIPID-P-1186 | TG(18:1/18:3/22:5) | TG | 1.214 | 0.699 | -0.516 | down |
| LIPID-P-1196 | TG(16:0/20:4/22:6) | TG | 1.399 | 0.609 | -0.715 | down |

Table S3: Differential lipids between the TB0 group and the TB6 group identified by lipidomics

| Index | Compounds | Class | VIP | Fold_Change | Log2FC | Type |
| --- | --- | --- | --- | --- | --- | --- |
| LIPID-N-0008 | FFA(20:4) | Eicosanoid | 1.588 | 1.374 | 0.459 | up |
| LIPID-N-0089 | FFA(14:1) | FFA | 1.024 | 1.657 | 0.729 | up |
| LIPID-N-0098 | FFA(16:2) | FFA | 1.057 | 1.335 | 0.417 | up |
| LIPID-N-0108 | FFA(22:4) | FFA | 1.687 | 1.446 | 0.532 | up |
| LIPID-N-0115 | LPA(0:0/16:0) | LPA | 3.644 | 0.334 | -1.582 | down |
| LIPID-N-0116 | LPA(0:0/18:0) | LPA | 3.655 | 0.335 | -1.579 | down |
| LIPID-N-0121 | LPC(20:0/0:0) | LPC | 2.214 | 1.488 | 0.573 | up |
| LIPID-N-0129 | LPC(18:2/0:0) | LPC | 1.578 | 1.347 | 0.430 | up |
| LIPID-N-0136 | LPC(22:4/0:0) | LPC | 1.394 | 1.306 | 0.385 | up |
| LIPID-N-0142 | LPE(0:0/20:0) | LPE | 1.860 | 1.547 | 0.630 | up |
| LIPID-N-0189 | PC(16:0/16:1) | PC | 1.072 | 0.663 | -0.594 | down |
| LIPID-N-0246 | PC(16:0/18:3) | PC | 1.216 | 0.786 | -0.347 | down |
| LIPID-N-0287 | PC(18:0/20:4) | PC | 1.986 | 1.313 | 0.393 | up |
| LIPID-N-0308 | PC(18:2/18:3) | PC | 1.029 | 1.381 | 0.465 | up |
| LIPID-N-0312 | PC(18:1/20:4) | PC | 1.750 | 1.315 | 0.395 | up |
| LIPID-N-0335 | PC(18:2/20:4) | PC | 1.489 | 1.259 | 0.332 | up |
| LIPID-N-0373 | PE(16:1/16:0) | PE | 1.558 | 0.434 | -1.205 | down |
| LIPID-N-0377 | PE(18:1/16:0) | PE | 1.174 | 0.753 | -0.410 | down |
| LIPID-N-0413 | PE(20:2/16:0) | PE | 1.484 | 0.729 | -0.456 | down |
| LIPID-N-0653 | PI(18:0/20:4) | PI | 1.594 | 1.297 | 0.375 | up |
| LIPID-N-0686 | PS(18:0/18:2) | PS | 1.861 | 1.371 | 0.456 | up |
| LIPID-P-0010 | Octanoyl-carnitine | CAR | 1.643 | 1.477 | 0.563 | up |
| LIPID-P-0013 | Decanoyl-carnitine | CAR | 1.467 | 1.442 | 0.528 | up |
| LIPID-P-0015 | Lauroyl-carnitine | CAR | 1.431 | 1.531 | 0.614 | up |
| LIPID-P-0029 | Decenoyl-carnitine | CAR | 1.538 | 1.435 | 0.521 | up |
| LIPID-P-0040 | Tetradecadienoyl- carnitine | CAR | 1.123 | 1.305 | 0.384 | up |
| LIPID-P-0053 | CE(18:2) | CE | 1.678 | 1.202 | 0.265 | up |
| LIPID-P-0058 | CE(20:4) | CE | 2.143 | 1.306 | 0.385 | up |
| LIPID-P-0059 | CE(22:4) | CE | 1.257 | 1.202 | 0.266 | up |
| LIPID-P-0062 | CE(22:6) | CE | 1.326 | 1.276 | 0.352 | up |
| LIPID-P-0067 | Cer(d18:0/24:0) | Cer | 2.010 | 1.483 | 0.569 | up |
| LIPID-P-0068 | Cer(d18:1/16:0) | Cer | 1.637 | 1.246 | 0.317 | up |
| LIPID-P-0069 | Cer(d18:1/18:0) | Cer | 1.758 | 1.494 | 0.579 | up |
| LIPID-P-0070 | Cer(d18:1/20:0) | Cer | 2.477 | 1.688 | 0.755 | up |
| LIPID-P-0071 | Cer(d18:1/22:0) | Cer | 2.179 | 1.586 | 0.665 | up |
| LIPID-P-0072 | Cer(d18:1/24:0) | Cer | 2.223 | 1.623 | 0.699 | up |
| LIPID-P-0077 | Cer(d18:1/24:1) | Cer | 1.153 | 1.202 | 0.265 | up |
| LIPID-P-0098 | CerP(d18:1/16:0) | CerP | 1.751 | 1.349 | 0.432 | up |
| LIPID-P-0099 | CerP(d18:1/18:0) | CerP | 1.654 | 1.309 | 0.388 | up |
| LIPID-P-0108 | CerP(d18:1/20:3) | CerP | 2.247 | 1.643 | 0.717 | up |
| LIPID-P-0111 | Cer(t18:0/22:0) | Cert | 1.664 | 1.473 | 0.559 | up |
| LIPID-P-0112 | Cer(t18:0/24:0) | Cert | 2.210 | 1.584 | 0.664 | up |
| LIPID-P-0116 | Coenzyme Q10 | COQ | 1.445 | 1.328 | 0.409 | up |
| LIPID-P-0129 | DG(18:0/18:0/0:0) | DG | 2.763 | 0.752 | -0.412 | down |
| LIPID-P-0225 | LPC(18:0/0:0) | LPC | 1.986 | 1.286 | 0.363 | up |
| LIPID-P-0227 | LPC(22:0/0:0) | LPC | 2.485 | 1.745 | 0.803 | up |
| LIPID-P-0228 | LPC(24:0/0:0) | LPC | 2.406 | 1.550 | 0.632 | up |
| LIPID-P-0243 | LPC(22:6/0:0) | LPC | 1.174 | 1.231 | 0.300 | up |
| LIPID-P-0246 | LPC(O-18:0/0:0) | LPC-O | 1.742 | 1.226 | 0.294 | up |
| LIPID-P-0266 | LPE(0:0/22:0) | LPE | 2.356 | 1.610 | 0.687 | up |
| LIPID-P-0292 | MG(16:0/0:0/0:0) | MG | 3.282 | 0.831 | -0.267 | down |
| LIPID-P-0293 | MG(18:0/0:0/0:0) | MG | 3.442 | 0.789 | -0.342 | down |
| LIPID-P-0352 | PC(18:0/18:2) | PC | 1.995 | 1.241 | 0.311 | up |
| LIPID-P-0362 | PC(18:2/22:0) | PC | 1.561 | 1.624 | 0.700 | up |
| LIPID-P-0376 | PC(18:1/18:2) | PC | 1.786 | 1.241 | 0.312 | up |
| LIPID-P-0388 | PC(18:0/20:3) | PC | 1.122 | 1.200 | 0.264 | up |
| LIPID-P-0394 | PC(18:3/14:1) | PC | 1.317 | 1.231 | 0.300 | up |
| LIPID-P-0402 | PC(18:2/18:2) | PC | 1.809 | 1.462 | 0.548 | up |
| LIPID-P-0417 | PC(22:4/16:0) | PC | 2.259 | 1.331 | 0.413 | up |
| LIPID-P-0422 | PC(18:0/22:4) | PC | 1.504 | 1.348 | 0.431 | up |
| LIPID-P-0445 | PC(16:0/22:5) | PC | 1.936 | 1.229 | 0.297 | up |
| LIPID-P-0454 | PC(18:0/22:5) | PC | 1.985 | 1.437 | 0.523 | up |
| LIPID-P-0465 | PC(14:0/22:6) | PC | 1.446 | 1.578 | 0.658 | up |
| LIPID-P-0473 | PC(16:0/22:6) | PC | 1.433 | 1.276 | 0.352 | up |
| LIPID-P-0479 | PC(18:0/22:6) | PC | 1.598 | 1.380 | 0.465 | up |
| LIPID-P-0480 | PC(20:1/20:5) | PC | 1.605 | 1.410 | 0.496 | up |
| LIPID-P-0483 | PC(20:0/22:6) | PC | 1.505 | 1.577 | 0.657 | up |
| LIPID-P-0489 | PC(16:1/22:6) | PC | 1.362 | 1.346 | 0.428 | up |
| LIPID-P-0496 | PC(18:1/22:6) | PC | 1.404 | 1.310 | 0.390 | up |
| LIPID-P-0504 | PC(18:2/22:6) | PC | 2.016 | 1.464 | 0.550 | up |
| LIPID-P-0507 | PC(20:2/22:6) | PC | 1.435 | 1.293 | 0.370 | up |
| LIPID-P-0529 | PC(O-16:0/16:2) | PC-O | 1.594 | 1.243 | 0.314 | up |
| LIPID-P-0532 | PC(O-18:1/20:1) | PC-O | 1.710 | 1.288 | 0.365 | up |
| LIPID-P-0533 | PC(O-20:1/20:1) | PC-O | 1.337 | 0.790 | -0.339 | down |
| LIPID-P-0535 | PC(O-16:2/18:1) | PC-O | 2.398 | 1.411 | 0.497 | up |
| LIPID-P-0547 | PC(O-18:3/18:2) | PC-O | 1.702 | 1.223 | 0.291 | up |
| LIPID-P-0557 | PC(O-18:3/18:4) | PC-O | 2.269 | 1.589 | 0.668 | up |
| LIPID-P-0562 | PC(O-20:4/20:4) | PC-O | 1.314 | 1.235 | 0.304 | up |
| LIPID-P-0568 | PE(16:0/16:0) | PE | 1.442 | 0.833 | -0.263 | down |
| LIPID-P-0577 | PE(16:1/18:0) | PE | 1.581 | 0.629 | -0.670 | down |
| LIPID-P-0584 | PE(18:2/12:0) | PE | 1.699 | 1.266 | 0.340 | up |
| LIPID-P-0588 | PE(22:2/12:0) | PE | 1.182 | 0.691 | -0.532 | down |
| LIPID-P-0589 | PE(16:0/18:2) | PE | 1.802 | 0.693 | -0.529 | down |
| LIPID-P-0637 | PE(20:3/20:5) | PE | 1.127 | 1.313 | 0.393 | up |
| LIPID-P-0643 | PE(P-18:1/18:0) | PE-P | 1.077 | 1.339 | 0.421 | up |
| LIPID-P-0646 | PE(P-18:2/16:0) | PE-P | 1.443 | 1.342 | 0.425 | up |
| LIPID-P-0647 | PE(P-18:2/18:0) | PE-P | 1.422 | 1.312 | 0.392 | up |
| LIPID-P-0657 | PE(P-18:2/18:2) | PE-P | 1.079 | 1.219 | 0.286 | up |
| LIPID-P-0659 | PE(P-20:2/20:2) | PE-P | 1.045 | 1.214 | 0.280 | up |
| LIPID-P-0663 | PE(P-20:2/20:3) | PE-P | 1.057 | 1.307 | 0.386 | up |
| LIPID-P-0667 | PE(P-20:2/20:4) | PE-P | 1.247 | 1.273 | 0.349 | up |
| LIPID-P-0716 | SM(d18:0/12:0) | SM | 2.248 | 1.212 | 0.277 | up |
| LIPID-P-0719 | SM(d18:0/18:0) | SM | 2.296 | 0.438 | -1.192 | down |
| LIPID-P-0736 | SM(d18:1/18:1) | SM | 2.413 | 1.357 | 0.440 | up |
| LIPID-P-0737 | SM(d18:1/20:1) | SM | 3.120 | 1.477 | 0.563 | up |
| LIPID-P-0738 | SM(d18:1/22:1) | SM | 2.500 | 1.451 | 0.537 | up |
| LIPID-P-0741 | SM(d18:2/16:1) | SM | 1.507 | 1.313 | 0.393 | up |
| LIPID-P-0742 | SM(d18:2/18:1) | SM | 1.843 | 1.221 | 0.288 | up |
| LIPID-P-0894 | TG(14:1/14:1/18:1) | TG | 1.123 | 1.256 | 0.329 | up |
| LIPID-P-0897 | TG(12:0/18:0/18:3) | TG | 1.310 | 2.501 | 1.323 | up |
| LIPID-P-0971 | TG(12:0/18:2/18:2) | TG | 1.529 | 2.088 | 1.062 | up |
| LIPID-P-0976 | TG(14:0/16:1/18:3) | TG | 1.146 | 1.656 | 0.728 | up |
| LIPID-P-0980 | TG(14:0/18:1/18:3) | TG | 1.243 | 1.498 | 0.583 | up |
| LIPID-P-1031 | TG(14:1/16:1/18:3) | TG | 1.593 | 2.131 | 1.092 | up |
| LIPID-P-1032 | TG(16:0/16:1/18:4) | TG | 1.253 | 1.483 | 0.569 | up |
| LIPID-P-1033 | TG(14:1/14:1/22:3) | TG | 1.325 | 1.557 | 0.639 | up |
| LIPID-P-1034 | TG(14:0/18:2/18:3) | TG | 1.253 | 1.570 | 0.650 | up |
| LIPID-P-1037 | TG(14:0/18:1/18:4) | TG | 1.024 | 1.405 | 0.491 | up |
| LIPID-P-1040 | TG(16:1/16:1/20:3) | TG | 1.388 | 1.464 | 0.550 | up |
| LIPID-P-1041 | TG(14:0/18:1/20:4) | TG | 1.209 | 1.375 | 0.460 | up |
| LIPID-P-1050 | TG(18:1/18:2/18:2) | TG | 1.169 | 1.336 | 0.418 | up |
| LIPID-P-1081 | TG(14:0/18:3/18:3) | TG | 1.297 | 1.580 | 0.660 | up |
| LIPID-P-1082 | TG(14:0/18:2/18:4) | TG | 1.077 | 1.610 | 0.687 | up |
| LIPID-P-1083 | TG(14:0/18:2/20:4) | TG | 1.564 | 1.915 | 0.937 | up |
| LIPID-P-1085 | TG(14:0/18:3/20:3) | TG | 1.341 | 1.491 | 0.576 | up |
| LIPID-P-1086 | TG(16:1/18:1/18:4) | TG | 1.418 | 1.553 | 0.635 | up |
| LIPID-P-1093 | TG(18:2/18:2/18:2) | TG | 1.152 | 1.500 | 0.585 | up |
| LIPID-P-1094 | TG(18:1/18:2/18:3) | TG | 1.112 | 1.433 | 0.519 | up |
| LIPID-P-1095 | TG(14:0/18:3/22:3) | TG | 1.291 | 1.469 | 0.555 | up |
| LIPID-P-1097 | TG(14:0/18:4/22:2) | TG | 1.042 | 1.316 | 0.396 | up |
| LIPID-P-1119 | TG(14:0/18:3/20:4) | TG | 1.327 | 1.797 | 0.845 | up |
| LIPID-P-1120 | TG(16:1/16:1/20:5) | TG | 1.436 | 1.552 | 0.634 | up |
| LIPID-P-1121 | TG(14:0/18:2/20:5) | TG | 1.238 | 1.716 | 0.779 | up |
| LIPID-P-1125 | TG(18:2/18:2/18:3) | TG | 1.085 | 1.649 | 0.721 | up |
| LIPID-P-1126 | TG(18:1/18:3/18:3) | TG | 1.103 | 1.608 | 0.685 | up |
| LIPID-P-1152 | TG(18:2/18:3/18:3) | TG | 1.041 | 1.641 | 0.714 | up |

Table S4: Results of reverse Mendelian randomization analysis for target lipids and proteins

| exposure | outcome | method | nsnp | pval | or | or_lci95 | or_uci95 |
| --- | --- | --- | --- | --- | --- | --- | --- |
| Tuberculosis | 1,2-dilinoleoyl-GPC (18:2/18:2) levels | Inverse variance weighted | 2 | 3.06E-01 | 0.875 | 0.678 | 1.129 |
| Tuberculosis | 1-myristoyl-2-arachidonoyl-GPC (14:0/20:4) levels | Inverse variance weighted | 2 | 5.21E-01 | 1.086 | 0.844 | 1.397 |
| Tuberculosis | 1-stearoyl-2-arachidonoyl-gpc (18:0/20:4) levels | Inverse variance weighted | 2 | 4.16E-01 | 1.200 | 0.773 | 1.863 |
| Tuberculosis | F13B | Inverse variance weighted | 2 | 2.84E-01 | 0.926 | 0.804 | 1.066 |
| Tuberculosis | HP | Inverse variance weighted | 2 | 3.19E-01 | 0.954 | 0.869 | 1.047 |
| Tuberculosis | ITIH1 | Inverse variance weighted | 2 | 5.93E-02 | 0.933 | 0.868 | 1.003 |
| Tuberculosis | RBP4 | Inverse variance weighted | 2 | 6.64E-01 | 1.017 | 0.943 | 1.097 |

Table S5: 75 plasma proteins screened by Mendelian randomization analysis

| id.exposure | id.outcome | outcome | exposure | method | nsnp | pval | or | or_lci95 | or_uci95 |
| --- | --- | --- | --- | --- | --- | --- | --- | --- | --- |
| 10036_201_ZHX3_ZHX3 | ebi-a-GCST90018672 | Pulmonary tuberculosis | ZHX3 | MR Egger | 5 | 1.73E-01 | 0.662 | 0.421 | 1.043 |
| 10036_201_ZHX3_ZHX3 | ebi-a-GCST90018672 | Pulmonary tuberculosis | ZHX3 | Weighted median | 5 | 4.43E-02 | 0.792 | 0.631 | 0.994 |
| 10036_201_ZHX3_ZHX3 | ebi-a-GCST90018672 | Pulmonary tuberculosis | ZHX3 | Inverse variance weighted | 5 | 4.26E-02 | 0.816 | 0.671 | 0.993 |
| 10036_201_ZHX3_ZHX3 | ebi-a-GCST90018672 | Pulmonary tuberculosis | ZHX3 | Simple mode | 5 | 1.81E-01 | 0.783 | 0.582 | 1.053 |
| 10036_201_ZHX3_ZHX3 | ebi-a-GCST90018672 | Pulmonary tuberculosis | ZHX3 | Weighted mode | 5 | 1.52E-01 | 0.786 | 0.603 | 1.026 |
| 10438_19_CSF2RA_CSF2R | ebi-a-GCST90018672 | Pulmonary tuberculosis | CSF2RA | MR Egger | 5 | 2.34E-01 | 0.289 | 0.056 | 1.489 |
| 10438_19_CSF2RA_CSF2R | ebi-a-GCST90018672 | Pulmonary tuberculosis | CSF2RA | Weighted median | 5 | 4.35E-02 | 0.702 | 0.498 | 0.990 |
| 10438_19_CSF2RA_CSF2R | ebi-a-GCST90018672 | Pulmonary tuberculosis | CSF2RA | Inverse variance weighted | 5 | 4.82E-02 | 0.754 | 0.570 | 0.998 |
| 10438_19_CSF2RA_CSF2R | ebi-a-GCST90018672 | Pulmonary tuberculosis | CSF2RA | Simple mode | 5 | 1.17E-01 | 0.685 | 0.471 | 0.994 |
| 10438_19_CSF2RA_CSF2R | ebi-a-GCST90018672 | Pulmonary tuberculosis | CSF2RA | Weighted mode | 5 | 9.37E-02 | 0.685 | 0.488 | 0.961 |
| 10561_5_PGLYRP3_PGRP_I_alpha | ebi-a-GCST90018672 | Pulmonary tuberculosis | PGLYRP3 | MR Egger | 4 | 6.26E-01 | 0.835 | 0.449 | 1.553 |
| 10561_5_PGLYRP3_PGRP_I_alpha | ebi-a-GCST90018672 | Pulmonary tuberculosis | PGLYRP3 | Weighted median | 4 | 1.26E-01 | 0.812 | 0.621 | 1.060 |
| 10561_5_PGLYRP3_PGRP_I_alpha | ebi-a-GCST90018672 | Pulmonary tuberculosis | PGLYRP3 | Inverse variance weighted | 4 | 1.68E-02 | 0.770 | 0.621 | 0.954 |
| 10561_5_PGLYRP3_PGRP_I_alpha | ebi-a-GCST90018672 | Pulmonary tuberculosis | PGLYRP3 | Simple mode | 4 | 4.04E-01 | 0.826 | 0.560 | 1.217 |
| 10561_5_PGLYRP3_PGRP_I_alpha | ebi-a-GCST90018672 | Pulmonary tuberculosis | PGLYRP3 | Weighted mode | 4 | 4.67E-01 | 0.835 | 0.546 | 1.278 |
| 10606_34_TOR1AIP1_TOIP1 | ebi-a-GCST90018672 | Pulmonary tuberculosis | TOR1AIP1 | MR Egger | 4 | 8.67E-02 | 0.491 | 0.316 | 0.762 |
| 10606_34_TOR1AIP1_TOIP1 | ebi-a-GCST90018672 | Pulmonary tuberculosis | TOR1AIP1 | Weighted median | 4 | 9.49E-08 | 0.595 | 0.492 | 0.720 |
| 10606_34_TOR1AIP1_TOIP1 | ebi-a-GCST90018672 | Pulmonary tuberculosis | TOR1AIP1 | Inverse variance weighted | 4 | 1.55E-04 | 0.628 | 0.493 | 0.799 |
| 10606_34_TOR1AIP1_TOIP1 | ebi-a-GCST90018672 | Pulmonary tuberculosis | TOR1AIP1 | Simple mode | 4 | 1.11E-01 | 0.578 | 0.358 | 0.934 |
| 10606_34_TOR1AIP1_TOIP1 | ebi-a-GCST90018672 | Pulmonary tuberculosis | TOR1AIP1 | Weighted mode | 4 | 1.33E-02 | 0.578 | 0.471 | 0.709 |
| 11116_16_C11orf87_CK087 | ebi-a-GCST90018672 | Pulmonary tuberculosis | C11orf87 | MR Egger | 10 | 5.20E-01 | 0.753 | 0.330 | 1.718 |
| 11116_16_C11orf87_CK087 | ebi-a-GCST90018672 | Pulmonary tuberculosis | C11orf87 | Weighted median | 10 | 4.77E-01 | 0.928 | 0.756 | 1.140 |
| 11116_16_C11orf87_CK087 | ebi-a-GCST90018672 | Pulmonary tuberculosis | C11orf87 | Inverse variance weighted | 10 | 3.68E-02 | 0.736 | 0.551 | 0.981 |
| 11116_16_C11orf87_CK087 | ebi-a-GCST90018672 | Pulmonary tuberculosis | C11orf87 | Simple mode | 10 | 9.92E-01 | 0.999 | 0.767 | 1.300 |
| 11116_16_C11orf87_CK087 | ebi-a-GCST90018672 | Pulmonary tuberculosis | C11orf87 | Weighted mode | 10 | 9.50E-01 | 0.994 | 0.818 | 1.207 |
| 11278_4_COL11A2_COL11A2 | ebi-a-GCST90018672 | Pulmonary tuberculosis | COL11A2 | MR Egger | 8 | 1.77E-02 | 2.760 | 1.494 | 5.101 |
| 11278_4_COL11A2_COL11A2 | ebi-a-GCST90018672 | Pulmonary tuberculosis | COL11A2 | Weighted median | 8 | 2.23E-02 | 1.471 | 1.057 | 2.048 |
| 11278_4_COL11A2_COL11A2 | ebi-a-GCST90018672 | Pulmonary tuberculosis | COL11A2 | Inverse variance weighted | 8 | 3.99E-02 | 1.443 | 1.017 | 2.047 |
| 11278_4_COL11A2_COL11A2 | ebi-a-GCST90018672 | Pulmonary tuberculosis | COL11A2 | Simple mode | 8 | 5.20E-01 | 1.238 | 0.667 | 2.297 |
| 11278_4_COL11A2_COL11A2 | ebi-a-GCST90018672 | Pulmonary tuberculosis | COL11A2 | Weighted mode | 8 | 1.69E-02 | 1.633 | 1.200 | 2.224 |
| 11315_148_PPM1D_PPM1D | ebi-a-GCST90018672 | Pulmonary tuberculosis | PPM1D | MR Egger | 4 | 2.81E-01 | 0.440 | 0.147 | 1.322 |
| 11315_148_PPM1D_PPM1D | ebi-a-GCST90018672 | Pulmonary tuberculosis | PPM1D | Weighted median | 4 | 2.47E-02 | 0.653 | 0.451 | 0.947 |
| 11315_148_PPM1D_PPM1D | ebi-a-GCST90018672 | Pulmonary tuberculosis | PPM1D | Inverse variance weighted | 4 | 2.08E-02 | 0.671 | 0.478 | 0.941 |
| 11315_148_PPM1D_PPM1D | ebi-a-GCST90018672 | Pulmonary tuberculosis | PPM1D | Simple mode | 4 | 7.73E-01 | 0.906 | 0.490 | 1.674 |
| 11315_148_PPM1D_PPM1D | ebi-a-GCST90018672 | Pulmonary tuberculosis | PPM1D | Weighted mode | 4 | 1.20E-01 | 0.545 | 0.314 | 0.946 |
| 11388_75_WFDC2_HE4 | ebi-a-GCST90018672 | Pulmonary tuberculosis | WFDC2 | MR Egger | 3 | 6.55E-01 | 1.515 | 0.392 | 5.856 |
| 11388_75_WFDC2_HE4 | ebi-a-GCST90018672 | Pulmonary tuberculosis | WFDC2 | Weighted median | 3 | 6.58E-02 | 1.314 | 0.982 | 1.758 |
| 11388_75_WFDC2_HE4 | ebi-a-GCST90018672 | Pulmonary tuberculosis | WFDC2 | Inverse variance weighted | 3 | 4.17E-02 | 1.312 | 1.010 | 1.703 |
| 11388_75_WFDC2_HE4 | ebi-a-GCST90018672 | Pulmonary tuberculosis | WFDC2 | Simple mode | 3 | 3.23E-01 | 1.271 | 0.886 | 1.823 |
| 11388_75_WFDC2_HE4 | ebi-a-GCST90018672 | Pulmonary tuberculosis | WFDC2 | Weighted mode | 3 | 2.16E-01 | 1.333 | 0.973 | 1.826 |
| 11430_49_DTX1_DTX1 | ebi-a-GCST90018672 | Pulmonary tuberculosis | DTX1 | MR Egger | 9 | 4.26E-01 | 0.602 | 0.185 | 1.954 |
| 11430_49_DTX1_DTX1 | ebi-a-GCST90018672 | Pulmonary tuberculosis | DTX1 | Weighted median | 9 | 8.57E-02 | 0.831 | 0.673 | 1.026 |
| 11430_49_DTX1_DTX1 | ebi-a-GCST90018672 | Pulmonary tuberculosis | DTX1 | Inverse variance weighted | 9 | 1.80E-02 | 0.725 | 0.555 | 0.946 |
| 11430_49_DTX1_DTX1 | ebi-a-GCST90018672 | Pulmonary tuberculosis | DTX1 | Simple mode | 9 | 5.97E-01 | 0.925 | 0.699 | 1.223 |
| 11430_49_DTX1_DTX1 | ebi-a-GCST90018672 | Pulmonary tuberculosis | DTX1 | Weighted mode | 9 | 7.10E-01 | 0.942 | 0.696 | 1.276 |
| 11989_35_EMC1_EMC1 | ebi-a-GCST90018672 | Pulmonary tuberculosis | EMC1 | MR Egger | 6 | 4.13E-01 | 0.668 | 0.281 | 1.590 |
| 11989_35_EMC1_EMC1 | ebi-a-GCST90018672 | Pulmonary tuberculosis | EMC1 | Weighted median | 6 | 9.24E-03 | 0.675 | 0.503 | 0.908 |
| 11989_35_EMC1_EMC1 | ebi-a-GCST90018672 | Pulmonary tuberculosis | EMC1 | Inverse variance weighted | 6 | 2.82E-03 | 0.706 | 0.561 | 0.887 |
| 11989_35_EMC1_EMC1 | ebi-a-GCST90018672 | Pulmonary tuberculosis | EMC1 | Simple mode | 6 | 7.75E-01 | 0.931 | 0.587 | 1.479 |
| 11989_35_EMC1_EMC1 | ebi-a-GCST90018672 | Pulmonary tuberculosis | EMC1 | Weighted mode | 6 | 6.01E-02 | 0.647 | 0.455 | 0.921 |
| 12473_48_KCTD5_KCTD5 | ebi-a-GCST90018672 | Pulmonary tuberculosis | KCTD5 | MR Egger | 5 | 3.40E-01 | 0.702 | 0.380 | 1.296 |
| 12473_48_KCTD5_KCTD5 | ebi-a-GCST90018672 | Pulmonary tuberculosis | KCTD5 | Weighted median | 5 | 8.72E-02 | 0.811 | 0.638 | 1.031 |
| 12473_48_KCTD5_KCTD5 | ebi-a-GCST90018672 | Pulmonary tuberculosis | KCTD5 | Inverse variance weighted | 5 | 3.10E-02 | 0.810 | 0.668 | 0.981 |
| 12473_48_KCTD5_KCTD5 | ebi-a-GCST90018672 | Pulmonary tuberculosis | KCTD5 | Simple mode | 5 | 4.44E-01 | 0.848 | 0.580 | 1.241 |
| 12473_48_KCTD5_KCTD5 | ebi-a-GCST90018672 | Pulmonary tuberculosis | KCTD5 | Weighted mode | 5 | 4.78E-01 | 0.859 | 0.588 | 1.257 |
| 12488_9_MCTS1_MCTS1 | ebi-a-GCST90018672 | Pulmonary tuberculosis | MCTS1 | MR Egger | 5 | 5.42E-01 | 0.796 | 0.414 | 1.529 |
| 12488_9_MCTS1_MCTS1 | ebi-a-GCST90018672 | Pulmonary tuberculosis | MCTS1 | Weighted median | 5 | 5.65E-02 | 0.789 | 0.618 | 1.007 |
| 12488_9_MCTS1_MCTS1 | ebi-a-GCST90018672 | Pulmonary tuberculosis | MCTS1 | Inverse variance weighted | 5 | 1.18E-02 | 0.764 | 0.620 | 0.942 |
| 12488_9_MCTS1_MCTS1 | ebi-a-GCST90018672 | Pulmonary tuberculosis | MCTS1 | Simple mode | 5 | 2.04E-01 | 0.798 | 0.597 | 1.068 |
| 12488_9_MCTS1_MCTS1 | ebi-a-GCST90018672 | Pulmonary tuberculosis | MCTS1 | Weighted mode | 5 | 1.49E-01 | 0.793 | 0.614 | 1.024 |
| 12571_14_ARL3_ARL3 | ebi-a-GCST90018672 | Pulmonary tuberculosis | ARL3 | MR Egger | 3 | 4.05E-01 | 1.182 | 0.928 | 1.504 |
| 12571_14_ARL3_ARL3 | ebi-a-GCST90018672 | Pulmonary tuberculosis | ARL3 | Weighted median | 3 | 3.55E-02 | 1.159 | 1.010 | 1.331 |
| 12571_14_ARL3_ARL3 | ebi-a-GCST90018672 | Pulmonary tuberculosis | ARL3 | Inverse variance weighted | 3 | 2.35E-02 | 1.165 | 1.021 | 1.330 |
| 12571_14_ARL3_ARL3 | ebi-a-GCST90018672 | Pulmonary tuberculosis | ARL3 | Simple mode | 3 | 5.11E-01 | 1.104 | 0.865 | 1.408 |
| 12571_14_ARL3_ARL3 | ebi-a-GCST90018672 | Pulmonary tuberculosis | ARL3 | Weighted mode | 3 | 1.80E-01 | 1.158 | 1.005 | 1.334 |
| 12605_1_EXOSC3_Exosome_component_3 | ebi-a-GCST90018672 | Pulmonary tuberculosis | EXOSC3 | MR Egger | 3 | 2.02E-01 | 103811.421 | 60.484 | 178176691.946 |
| 12605_1_EXOSC3_Exosome_component_3 | ebi-a-GCST90018672 | Pulmonary tuberculosis | EXOSC3 | Weighted median | 3 | 2.40E-03 | 3.703 | 1.590 | 8.622 |
| 12605_1_EXOSC3_Exosome_component_3 | ebi-a-GCST90018672 | Pulmonary tuberculosis | EXOSC3 | Inverse variance weighted | 3 | 3.34E-03 | 4.928 | 1.698 | 14.297 |
| 12605_1_EXOSC3_Exosome_component_3 | ebi-a-GCST90018672 | Pulmonary tuberculosis | EXOSC3 | Simple mode | 3 | 8.97E-01 | 1.112 | 0.269 | 4.587 |
| 12605_1_EXOSC3_Exosome_component_3 | ebi-a-GCST90018672 | Pulmonary tuberculosis | EXOSC3 | Weighted mode | 3 | 4.65E-02 | 6.816 | 2.941 | 15.794 |
| 12750_9_ITGB2_LFA_1_beta_2 | ebi-a-GCST90018672 | Pulmonary tuberculosis | ITGB2 | MR Egger | 4 | 1.79E-01 | 0.446 | 0.205 | 0.970 |
| 12750_9_ITGB2_LFA_1_beta_2 | ebi-a-GCST90018672 | Pulmonary tuberculosis | ITGB2 | Weighted median | 4 | 9.22E-03 | 0.640 | 0.457 | 0.896 |
| 12750_9_ITGB2_LFA_1_beta_2 | ebi-a-GCST90018672 | Pulmonary tuberculosis | ITGB2 | Inverse variance weighted | 4 | 1.86E-02 | 0.656 | 0.462 | 0.932 |
| 12750_9_ITGB2_LFA_1_beta_2 | ebi-a-GCST90018672 | Pulmonary tuberculosis | ITGB2 | Simple mode | 4 | 7.97E-01 | 0.905 | 0.451 | 1.816 |
| 12750_9_ITGB2_LFA_1_beta_2 | ebi-a-GCST90018672 | Pulmonary tuberculosis | ITGB2 | Weighted mode | 4 | 3.42E-02 | 0.532 | 0.381 | 0.743 |
| 12768_3_PPP1R3B_PPR3B | ebi-a-GCST90018672 | Pulmonary tuberculosis | PPP1R3B | MR Egger | 3 | 6.69E-01 | 0.918 | 0.686 | 1.229 |
| 12768_3_PPP1R3B_PPR3B | ebi-a-GCST90018672 | Pulmonary tuberculosis | PPP1R3B | Weighted median | 3 | 8.56E-02 | 0.865 | 0.733 | 1.021 |
| 12768_3_PPP1R3B_PPR3B | ebi-a-GCST90018672 | Pulmonary tuberculosis | PPP1R3B | Inverse variance weighted | 3 | 3.23E-02 | 0.843 | 0.721 | 0.986 |
| 12768_3_PPP1R3B_PPR3B | ebi-a-GCST90018672 | Pulmonary tuberculosis | PPP1R3B | Simple mode | 3 | 3.47E-01 | 0.863 | 0.682 | 1.093 |
| 12768_3_PPP1R3B_PPR3B | ebi-a-GCST90018672 | Pulmonary tuberculosis | PPP1R3B | Weighted mode | 3 | 2.27E-01 | 0.866 | 0.735 | 1.020 |
| 13025_4_FRS2_FRS2 | ebi-a-GCST90018672 | Pulmonary tuberculosis | FRS2 | MR Egger | 5 | 4.65E-01 | 0.725 | 0.341 | 1.541 |
| 13025_4_FRS2_FRS2 | ebi-a-GCST90018672 | Pulmonary tuberculosis | FRS2 | Weighted median | 5 | 6.50E-02 | 0.769 | 0.582 | 1.016 |
| 13025_4_FRS2_FRS2 | ebi-a-GCST90018672 | Pulmonary tuberculosis | FRS2 | Inverse variance weighted | 5 | 2.17E-02 | 0.780 | 0.631 | 0.964 |
| 13025_4_FRS2_FRS2 | ebi-a-GCST90018672 | Pulmonary tuberculosis | FRS2 | Simple mode | 5 | 1.71E-01 | 0.691 | 0.447 | 1.067 |
| 13025_4_FRS2_FRS2 | ebi-a-GCST90018672 | Pulmonary tuberculosis | FRS2 | Weighted mode | 5 | 1.44E-01 | 0.676 | 0.443 | 1.032 |
| 13113_7_SPP1_Osteopontin | ebi-a-GCST90018672 | Pulmonary tuberculosis | SPP1 | MR Egger | 3 | 9.44E-01 | 0.984 | 0.684 | 1.416 |
| 13113_7_SPP1_Osteopontin | ebi-a-GCST90018672 | Pulmonary tuberculosis | SPP1 | Weighted median | 3 | 3.84E-02 | 0.830 | 0.695 | 0.990 |
| 13113_7_SPP1_Osteopontin | ebi-a-GCST90018672 | Pulmonary tuberculosis | SPP1 | Inverse variance weighted | 3 | 3.03E-02 | 0.828 | 0.698 | 0.982 |
| 13113_7_SPP1_Osteopontin | ebi-a-GCST90018672 | Pulmonary tuberculosis | SPP1 | Simple mode | 3 | 1.36E-01 | 0.468 | 0.253 | 0.865 |
| 13113_7_SPP1_Osteopontin | ebi-a-GCST90018672 | Pulmonary tuberculosis | SPP1 | Weighted mode | 3 | 1.76E-01 | 0.842 | 0.715 | 0.992 |
| 13439_6_IQCF3_IQCF3 | ebi-a-GCST90018672 | Pulmonary tuberculosis | IQCF3 | MR Egger | 4 | 3.43E-01 | 0.547 | 0.209 | 1.429 |
| 13439_6_IQCF3_IQCF3 | ebi-a-GCST90018672 | Pulmonary tuberculosis | IQCF3 | Weighted median | 4 | 1.59E-01 | 0.791 | 0.571 | 1.096 |
| 13439_6_IQCF3_IQCF3 | ebi-a-GCST90018672 | Pulmonary tuberculosis | IQCF3 | Inverse variance weighted | 4 | 3.68E-02 | 0.754 | 0.578 | 0.983 |
| 13439_6_IQCF3_IQCF3 | ebi-a-GCST90018672 | Pulmonary tuberculosis | IQCF3 | Simple mode | 4 | 7.52E-01 | 0.913 | 0.545 | 1.529 |
| 13439_6_IQCF3_IQCF3 | ebi-a-GCST90018672 | Pulmonary tuberculosis | IQCF3 | Weighted mode | 4 | 5.87E-01 | 0.864 | 0.539 | 1.385 |
| 14131_37_EFNB2_EFNB2 | ebi-a-GCST90018672 | Pulmonary tuberculosis | EFNB2 | MR Egger | 8 | 9.04E-01 | 1.019 | 0.759 | 1.369 |
| 14131_37_EFNB2_EFNB2 | ebi-a-GCST90018672 | Pulmonary tuberculosis | EFNB2 | Weighted median | 8 | 1.24E-01 | 1.151 | 0.962 | 1.377 |
| 14131_37_EFNB2_EFNB2 | ebi-a-GCST90018672 | Pulmonary tuberculosis | EFNB2 | Inverse variance weighted | 8 | 3.50E-02 | 1.170 | 1.011 | 1.353 |
| 14131_37_EFNB2_EFNB2 | ebi-a-GCST90018672 | Pulmonary tuberculosis | EFNB2 | Simple mode | 8 | 2.97E-01 | 1.195 | 0.877 | 1.628 |
| 14131_37_EFNB2_EFNB2 | ebi-a-GCST90018672 | Pulmonary tuberculosis | EFNB2 | Weighted mode | 8 | 2.00E-01 | 1.142 | 0.950 | 1.371 |
| 15580_2_EPHA7_EPHA7 | ebi-a-GCST90018672 | Pulmonary tuberculosis | EPHA7 | MR Egger | 4 | 6.70E-02 | 0.386 | 0.232 | 0.642 |
| 15580_2_EPHA7_EPHA7 | ebi-a-GCST90018672 | Pulmonary tuberculosis | EPHA7 | Weighted median | 4 | 4.47E-03 | 0.652 | 0.485 | 0.876 |
| 15580_2_EPHA7_EPHA7 | ebi-a-GCST90018672 | Pulmonary tuberculosis | EPHA7 | Inverse variance weighted | 4 | 6.20E-03 | 0.624 | 0.446 | 0.875 |
| 15580_2_EPHA7_EPHA7 | ebi-a-GCST90018672 | Pulmonary tuberculosis | EPHA7 | Simple mode | 4 | 7.07E-01 | 0.889 | 0.508 | 1.555 |
| 15580_2_EPHA7_EPHA7 | ebi-a-GCST90018672 | Pulmonary tuberculosis | EPHA7 | Weighted mode | 4 | 3.67E-02 | 0.579 | 0.430 | 0.780 |
| 15633_6_RBP4_RBP | ebi-a-GCST90018672 | Pulmonary tuberculosis | RBP4 | MR Egger | 5 | 1.59E-01 | 0.265 | 0.066 | 1.068 |
| 15633_6_RBP4_RBP | ebi-a-GCST90018672 | Pulmonary tuberculosis | RBP4 | Weighted median | 5 | 1.98E-01 | 0.791 | 0.553 | 1.131 |
| 15633_6_RBP4_RBP | ebi-a-GCST90018672 | Pulmonary tuberculosis | RBP4 | Inverse variance weighted | 5 | 3.33E-02 | 0.694 | 0.496 | 0.972 |
| 15633_6_RBP4_RBP | ebi-a-GCST90018672 | Pulmonary tuberculosis | RBP4 | Simple mode | 5 | 4.49E-01 | 0.823 | 0.522 | 1.298 |
| 15633_6_RBP4_RBP | ebi-a-GCST90018672 | Pulmonary tuberculosis | RBP4 | Weighted mode | 5 | 3.71E-01 | 0.814 | 0.545 | 1.215 |
| 16805_5_PDE5A_PDE5A | ebi-a-GCST90018672 | Pulmonary tuberculosis | PDE5A | MR Egger | 3 | 2.57E-01 | 0.634 | 0.433 | 0.928 |
| 16805_5_PDE5A_PDE5A | ebi-a-GCST90018672 | Pulmonary tuberculosis | PDE5A | Weighted median | 3 | 1.18E-03 | 0.778 | 0.668 | 0.905 |
| 16805_5_PDE5A_PDE5A | ebi-a-GCST90018672 | Pulmonary tuberculosis | PDE5A | Inverse variance weighted | 3 | 5.49E-03 | 0.792 | 0.672 | 0.934 |
| 16805_5_PDE5A_PDE5A | ebi-a-GCST90018672 | Pulmonary tuberculosis | PDE5A | Simple mode | 3 | 1.06E-01 | 0.763 | 0.632 | 0.921 |
| 16805_5_PDE5A_PDE5A | ebi-a-GCST90018672 | Pulmonary tuberculosis | PDE5A | Weighted mode | 3 | 9.11E-02 | 0.774 | 0.658 | 0.911 |
| 17152_10_KIR2DS4_KI2S4 | ebi-a-GCST90018672 | Pulmonary tuberculosis | KIR2DS4 | MR Egger | 5 | 2.15E-01 | 0.707 | 0.458 | 1.091 |
| 17152_10_KIR2DS4_KI2S4 | ebi-a-GCST90018672 | Pulmonary tuberculosis | KIR2DS4 | Weighted median | 5 | 4.13E-02 | 0.777 | 0.610 | 0.990 |
| 17152_10_KIR2DS4_KI2S4 | ebi-a-GCST90018672 | Pulmonary tuberculosis | KIR2DS4 | Inverse variance weighted | 5 | 4.73E-03 | 0.759 | 0.626 | 0.919 |
| 17152_10_KIR2DS4_KI2S4 | ebi-a-GCST90018672 | Pulmonary tuberculosis | KIR2DS4 | Simple mode | 5 | 4.90E-01 | 0.875 | 0.620 | 1.235 |
| 17152_10_KIR2DS4_KI2S4 | ebi-a-GCST90018672 | Pulmonary tuberculosis | KIR2DS4 | Weighted mode | 5 | 1.27E-01 | 0.776 | 0.599 | 1.005 |
| 17153_46_KIR2DL3_KI2L3 | ebi-a-GCST90018672 | Pulmonary tuberculosis | KIR2DL3 | MR Egger | 9 | 2.42E-02 | 0.726 | 0.582 | 0.904 |
| 17153_46_KIR2DL3_KI2L3 | ebi-a-GCST90018672 | Pulmonary tuberculosis | KIR2DL3 | Weighted median | 9 | 5.79E-05 | 0.805 | 0.724 | 0.895 |
| 17153_46_KIR2DL3_KI2L3 | ebi-a-GCST90018672 | Pulmonary tuberculosis | KIR2DL3 | Inverse variance weighted | 9 | 1.84E-04 | 0.801 | 0.714 | 0.900 |
| 17153_46_KIR2DL3_KI2L3 | ebi-a-GCST90018672 | Pulmonary tuberculosis | KIR2DL3 | Simple mode | 9 | 4.57E-02 | 0.792 | 0.653 | 0.961 |
| 17153_46_KIR2DL3_KI2L3 | ebi-a-GCST90018672 | Pulmonary tuberculosis | KIR2DL3 | Weighted mode | 9 | 1.93E-03 | 0.792 | 0.716 | 0.876 |
| 18285_6_SVIP_SVIP | ebi-a-GCST90018672 | Pulmonary tuberculosis | SVIP | MR Egger | 3 | 1.68E-01 | 0.582 | 0.437 | 0.775 |
| 18285_6_SVIP_SVIP | ebi-a-GCST90018672 | Pulmonary tuberculosis | SVIP | Weighted median | 3 | 4.15E-04 | 0.747 | 0.635 | 0.878 |
| 18285_6_SVIP_SVIP | ebi-a-GCST90018672 | Pulmonary tuberculosis | SVIP | Inverse variance weighted | 3 | 1.02E-02 | 0.747 | 0.598 | 0.933 |
| 18285_6_SVIP_SVIP | ebi-a-GCST90018672 | Pulmonary tuberculosis | SVIP | Simple mode | 3 | 9.69E-01 | 0.989 | 0.613 | 1.597 |
| 18285_6_SVIP_SVIP | ebi-a-GCST90018672 | Pulmonary tuberculosis | SVIP | Weighted mode | 3 | 5.44E-02 | 0.692 | 0.581 | 0.825 |
| 18925_24_PSMA5_Proteasome_subunit_alpha_type_5 | ebi-a-GCST90018672 | Pulmonary tuberculosis | PSMA5 | MR Egger | 10 | 9.31E-01 | 0.974 | 0.551 | 1.722 |
| 18925_24_PSMA5_Proteasome_subunit_alpha_type_5 | ebi-a-GCST90018672 | Pulmonary tuberculosis | PSMA5 | Weighted median | 10 | 2.90E-01 | 0.918 | 0.785 | 1.075 |
| 18925_24_PSMA5_Proteasome_subunit_alpha_type_5 | ebi-a-GCST90018672 | Pulmonary tuberculosis | PSMA5 | Inverse variance weighted | 10 | 4.53E-02 | 0.802 | 0.647 | 0.995 |
| 18925_24_PSMA5_Proteasome_subunit_alpha_type_5 | ebi-a-GCST90018672 | Pulmonary tuberculosis | PSMA5 | Simple mode | 10 | 6.94E-01 | 0.952 | 0.752 | 1.206 |
| 18925_24_PSMA5_Proteasome_subunit_alpha_type_5 | ebi-a-GCST90018672 | Pulmonary tuberculosis | PSMA5 | Weighted mode | 10 | 5.90E-01 | 0.952 | 0.802 | 1.131 |
| 19108_50_MECP2_MECP2 | ebi-a-GCST90018672 | Pulmonary tuberculosis | MECP2 | MR Egger | 3 | 4.81E-01 | 0.324 | 0.040 | 2.595 |
| 19108_50_MECP2_MECP2 | ebi-a-GCST90018672 | Pulmonary tuberculosis | MECP2 | Weighted median | 3 | 7.67E-02 | 0.578 | 0.315 | 1.060 |
| 19108_50_MECP2_MECP2 | ebi-a-GCST90018672 | Pulmonary tuberculosis | MECP2 | Inverse variance weighted | 3 | 2.57E-02 | 0.587 | 0.367 | 0.937 |
| 19108_50_MECP2_MECP2 | ebi-a-GCST90018672 | Pulmonary tuberculosis | MECP2 | Simple mode | 3 | 2.81E-01 | 0.541 | 0.237 | 1.233 |
| 19108_50_MECP2_MECP2 | ebi-a-GCST90018672 | Pulmonary tuberculosis | MECP2 | Weighted mode | 3 | 2.35E-01 | 0.512 | 0.235 | 1.117 |
| 19277_4_TSTD1_KAT | ebi-a-GCST90018672 | Pulmonary tuberculosis | TSTD1 | MR Egger | 3 | 3.67E-01 | 1.766 | 0.855 | 3.648 |
| 19277_4_TSTD1_KAT | ebi-a-GCST90018672 | Pulmonary tuberculosis | TSTD1 | Weighted median | 3 | 1.12E-01 | 1.402 | 0.924 | 2.127 |
| 19277_4_TSTD1_KAT | ebi-a-GCST90018672 | Pulmonary tuberculosis | TSTD1 | Inverse variance weighted | 3 | 4.89E-02 | 1.421 | 1.002 | 2.016 |
| 19277_4_TSTD1_KAT | ebi-a-GCST90018672 | Pulmonary tuberculosis | TSTD1 | Simple mode | 3 | 3.30E-01 | 1.347 | 0.852 | 2.127 |
| 19277_4_TSTD1_KAT | ebi-a-GCST90018672 | Pulmonary tuberculosis | TSTD1 | Weighted mode | 3 | 3.01E-01 | 1.371 | 0.877 | 2.143 |
| 19323_1_EBAG9_RCAS1 | ebi-a-GCST90018672 | Pulmonary tuberculosis | EBAG9 | MR Egger | 4 | 3.18E-01 | 0.725 | 0.449 | 1.170 |
| 19323_1_EBAG9_RCAS1 | ebi-a-GCST90018672 | Pulmonary tuberculosis | EBAG9 | Weighted median | 4 | 2.12E-02 | 0.767 | 0.612 | 0.961 |
| 19323_1_EBAG9_RCAS1 | ebi-a-GCST90018672 | Pulmonary tuberculosis | EBAG9 | Inverse variance weighted | 4 | 1.30E-03 | 0.728 | 0.599 | 0.883 |
| 19323_1_EBAG9_RCAS1 | ebi-a-GCST90018672 | Pulmonary tuberculosis | EBAG9 | Simple mode | 4 | 1.81E-01 | 0.746 | 0.536 | 1.038 |
| 19323_1_EBAG9_RCAS1 | ebi-a-GCST90018672 | Pulmonary tuberculosis | EBAG9 | Weighted mode | 4 | 1.36E-01 | 0.759 | 0.581 | 0.991 |
| 19579_5_DEFA1_HNP_1 | ebi-a-GCST90018672 | Pulmonary tuberculosis | DEFA1 | MR Egger | 5 | 1.61E-01 | 1.763 | 0.969 | 3.207 |
| 19579_5_DEFA1_HNP_1 | ebi-a-GCST90018672 | Pulmonary tuberculosis | DEFA1 | Weighted median | 5 | 2.23E-03 | 1.452 | 1.143 | 1.845 |
| 19579_5_DEFA1_HNP_1 | ebi-a-GCST90018672 | Pulmonary tuberculosis | DEFA1 | Inverse variance weighted | 5 | 3.35E-02 | 1.368 | 1.025 | 1.827 |
| 19579_5_DEFA1_HNP_1 | ebi-a-GCST90018672 | Pulmonary tuberculosis | DEFA1 | Simple mode | 5 | 1.54E-01 | 1.427 | 0.959 | 2.124 |
| 19579_5_DEFA1_HNP_1 | ebi-a-GCST90018672 | Pulmonary tuberculosis | DEFA1 | Weighted mode | 5 | 6.64E-02 | 1.463 | 1.086 | 1.970 |
| 2731_29_POR_NADPH_P450_Oxidoreductase | ebi-a-GCST90018672 | Pulmonary tuberculosis | POR | MR Egger | 5 | 2.67E-01 | 1.284 | 0.896 | 1.839 |
| 2731_29_POR_NADPH_P450_Oxidoreductase | ebi-a-GCST90018672 | Pulmonary tuberculosis | POR | Weighted median | 5 | 4.47E-02 | 1.185 | 1.004 | 1.398 |
| 2731_29_POR_NADPH_P450_Oxidoreductase | ebi-a-GCST90018672 | Pulmonary tuberculosis | POR | Inverse variance weighted | 5 | 2.41E-02 | 1.189 | 1.023 | 1.382 |
| 2731_29_POR_NADPH_P450_Oxidoreductase | ebi-a-GCST90018672 | Pulmonary tuberculosis | POR | Simple mode | 5 | 5.30E-01 | 1.090 | 0.852 | 1.394 |
| 2731_29_POR_NADPH_P450_Oxidoreductase | ebi-a-GCST90018672 | Pulmonary tuberculosis | POR | Weighted mode | 5 | 1.07E-01 | 1.187 | 1.009 | 1.397 |
| 2849_49_AIF1_AIF1 | ebi-a-GCST90018672 | Pulmonary tuberculosis | AIF1 | MR Egger | 4 | 2.20E-01 | 1.662 | 0.944 | 2.926 |
| 2849_49_AIF1_AIF1 | ebi-a-GCST90018672 | Pulmonary tuberculosis | AIF1 | Weighted median | 4 | 7.75E-02 | 1.281 | 0.973 | 1.686 |
| 2849_49_AIF1_AIF1 | ebi-a-GCST90018672 | Pulmonary tuberculosis | AIF1 | Inverse variance weighted | 4 | 2.46E-02 | 1.303 | 1.035 | 1.642 |
| 2849_49_AIF1_AIF1 | ebi-a-GCST90018672 | Pulmonary tuberculosis | AIF1 | Simple mode | 4 | 5.32E-01 | 1.159 | 0.768 | 1.750 |
| 2849_49_AIF1_AIF1 | ebi-a-GCST90018672 | Pulmonary tuberculosis | AIF1 | Weighted mode | 4 | 8.49E-02 | 1.518 | 1.100 | 2.096 |
| 3054_3_HP_Haptoglobin__Mixed_Type | ebi-a-GCST90018672 | Pulmonary tuberculosis | HP | MR Egger | 6 | 1.55E-01 | 1.255 | 0.973 | 1.619 |
| 3054_3_HP_Haptoglobin__Mixed_Type | ebi-a-GCST90018672 | Pulmonary tuberculosis | HP | Weighted median | 6 | 1.91E-03 | 1.191 | 1.066 | 1.330 |
| 3054_3_HP_Haptoglobin__Mixed_Type | ebi-a-GCST90018672 | Pulmonary tuberculosis | HP | Inverse variance weighted | 6 | 3.63E-02 | 1.157 | 1.009 | 1.326 |
| 3054_3_HP_Haptoglobin__Mixed_Type | ebi-a-GCST90018672 | Pulmonary tuberculosis | HP | Simple mode | 6 | 3.18E-01 | 1.128 | 0.911 | 1.397 |
| 3054_3_HP_Haptoglobin__Mixed_Type | ebi-a-GCST90018672 | Pulmonary tuberculosis | HP | Weighted mode | 6 | 3.09E-02 | 1.191 | 1.062 | 1.336 |
| 3171_57_APP_amyloid_precursor_protein | ebi-a-GCST90018672 | Pulmonary tuberculosis | APP | MR Egger | 5 | 2.39E-01 | 1.384 | 0.896 | 2.139 |
| 3171_57_APP_amyloid_precursor_protein | ebi-a-GCST90018672 | Pulmonary tuberculosis | APP | Weighted median | 5 | 9.91E-03 | 1.264 | 1.058 | 1.510 |
| 3171_57_APP_amyloid_precursor_protein | ebi-a-GCST90018672 | Pulmonary tuberculosis | APP | Inverse variance weighted | 5 | 1.14E-02 | 1.239 | 1.049 | 1.462 |
| 3171_57_APP_amyloid_precursor_protein | ebi-a-GCST90018672 | Pulmonary tuberculosis | APP | Simple mode | 5 | 2.07E-01 | 1.217 | 0.942 | 1.571 |
| 3171_57_APP_amyloid_precursor_protein | ebi-a-GCST90018672 | Pulmonary tuberculosis | APP | Weighted mode | 5 | 6.40E-02 | 1.253 | 1.053 | 1.491 |
| 3179_51_CTSA_Cathepsin_A | ebi-a-GCST90018672 | Pulmonary tuberculosis | CTSA | MR Egger | 12 | 7.95E-01 | 1.022 | 0.872 | 1.197 |
| 3179_51_CTSA_Cathepsin_A | ebi-a-GCST90018672 | Pulmonary tuberculosis | CTSA | Weighted median | 12 | 8.51E-02 | 1.102 | 0.987 | 1.232 |
| 3179_51_CTSA_Cathepsin_A | ebi-a-GCST90018672 | Pulmonary tuberculosis | CTSA | Inverse variance weighted | 12 | 3.83E-02 | 1.100 | 1.005 | 1.204 |
| 3179_51_CTSA_Cathepsin_A | ebi-a-GCST90018672 | Pulmonary tuberculosis | CTSA | Simple mode | 12 | 3.66E-01 | 1.088 | 0.913 | 1.295 |
| 3179_51_CTSA_Cathepsin_A | ebi-a-GCST90018672 | Pulmonary tuberculosis | CTSA | Weighted mode | 12 | 1.65E-01 | 1.088 | 0.974 | 1.215 |
| 3461_58_BCAN_PGCB | ebi-a-GCST90018672 | Pulmonary tuberculosis | BCAN | MR Egger | 3 | 4.18E-01 | 1.381 | 0.849 | 2.247 |
| 3461_58_BCAN_PGCB | ebi-a-GCST90018672 | Pulmonary tuberculosis | BCAN | Weighted median | 3 | 2.77E-02 | 1.222 | 1.022 | 1.462 |
| 3461_58_BCAN_PGCB | ebi-a-GCST90018672 | Pulmonary tuberculosis | BCAN | Inverse variance weighted | 3 | 4.60E-02 | 1.206 | 1.003 | 1.450 |
| 3461_58_BCAN_PGCB | ebi-a-GCST90018672 | Pulmonary tuberculosis | BCAN | Simple mode | 3 | 1.89E-01 | 1.328 | 1.000 | 1.763 |
| 3461_58_BCAN_PGCB | ebi-a-GCST90018672 | Pulmonary tuberculosis | BCAN | Weighted mode | 3 | 1.44E-01 | 1.230 | 1.034 | 1.462 |
| 3485_28_B2M_b2_Microglobulin | ebi-a-GCST90018672 | Pulmonary tuberculosis | B2M | MR Egger | 5 | 2.99E-01 | 1.676 | 0.746 | 3.763 |
| 3485_28_B2M_b2_Microglobulin | ebi-a-GCST90018672 | Pulmonary tuberculosis | B2M | Weighted median | 5 | 4.93E-02 | 1.386 | 1.001 | 1.919 |
| 3485_28_B2M_b2_Microglobulin | ebi-a-GCST90018672 | Pulmonary tuberculosis | B2M | Inverse variance weighted | 5 | 1.97E-02 | 1.396 | 1.055 | 1.849 |
| 3485_28_B2M_b2_Microglobulin | ebi-a-GCST90018672 | Pulmonary tuberculosis | B2M | Simple mode | 5 | 1.62E-01 | 1.369 | 0.956 | 1.962 |
| 3485_28_B2M_b2_Microglobulin | ebi-a-GCST90018672 | Pulmonary tuberculosis | B2M | Weighted mode | 5 | 1.19E-01 | 1.408 | 1.003 | 1.977 |
| 3495_15_CXCL6_GCP_2 | ebi-a-GCST90018672 | Pulmonary tuberculosis | CXCL6 | MR Egger | 4 | 2.27E-01 | 0.904 | 0.807 | 1.014 |
| 3495_15_CXCL6_GCP_2 | ebi-a-GCST90018672 | Pulmonary tuberculosis | CXCL6 | Weighted median | 4 | 2.39E-02 | 0.923 | 0.860 | 0.989 |
| 3495_15_CXCL6_GCP_2 | ebi-a-GCST90018672 | Pulmonary tuberculosis | CXCL6 | Inverse variance weighted | 4 | 2.52E-02 | 0.925 | 0.864 | 0.990 |
| 3495_15_CXCL6_GCP_2 | ebi-a-GCST90018672 | Pulmonary tuberculosis | CXCL6 | Simple mode | 4 | 3.28E-01 | 0.928 | 0.818 | 1.052 |
| 3495_15_CXCL6_GCP_2 | ebi-a-GCST90018672 | Pulmonary tuberculosis | CXCL6 | Weighted mode | 4 | 1.29E-01 | 0.928 | 0.865 | 0.996 |
| 3592_4_CAMK1_CAMK1 | ebi-a-GCST90018672 | Pulmonary tuberculosis | CAMK1 | MR Egger | 6 | 2.67E-01 | 0.931 | 0.835 | 1.038 |
| 3592_4_CAMK1_CAMK1 | ebi-a-GCST90018672 | Pulmonary tuberculosis | CAMK1 | Weighted median | 6 | 9.64E-02 | 0.926 | 0.845 | 1.014 |
| 3592_4_CAMK1_CAMK1 | ebi-a-GCST90018672 | Pulmonary tuberculosis | CAMK1 | Inverse variance weighted | 6 | 4.85E-02 | 0.917 | 0.841 | 0.999 |
| 3592_4_CAMK1_CAMK1 | ebi-a-GCST90018672 | Pulmonary tuberculosis | CAMK1 | Simple mode | 6 | 4.08E-01 | 0.909 | 0.740 | 1.117 |
| 3592_4_CAMK1_CAMK1 | ebi-a-GCST90018672 | Pulmonary tuberculosis | CAMK1 | Weighted mode | 6 | 1.66E-01 | 0.928 | 0.847 | 1.016 |
| 4124_24_HSPA1A_HSP_70 | ebi-a-GCST90018672 | Pulmonary tuberculosis | HSPA1A | MR Egger | 6 | 2.08E-01 | 0.410 | 0.128 | 1.315 |
| 4124_24_HSPA1A_HSP_70 | ebi-a-GCST90018672 | Pulmonary tuberculosis | HSPA1A | Weighted median | 6 | 8.14E-07 | 0.471 | 0.350 | 0.636 |
| 4124_24_HSPA1A_HSP_70 | ebi-a-GCST90018672 | Pulmonary tuberculosis | HSPA1A | Inverse variance weighted | 6 | 1.30E-03 | 0.516 | 0.345 | 0.772 |
| 4124_24_HSPA1A_HSP_70 | ebi-a-GCST90018672 | Pulmonary tuberculosis | HSPA1A | Simple mode | 6 | 3.91E-02 | 0.560 | 0.372 | 0.843 |
| 4124_24_HSPA1A_HSP_70 | ebi-a-GCST90018672 | Pulmonary tuberculosis | HSPA1A | Weighted mode | 6 | 4.76E-03 | 0.480 | 0.356 | 0.647 |
| 4155_3_TNC_Tenascin | ebi-a-GCST90018672 | Pulmonary tuberculosis | TNC | MR Egger | 9 | 7.46E-02 | 1.224 | 1.013 | 1.479 |
| 4155_3_TNC_Tenascin | ebi-a-GCST90018672 | Pulmonary tuberculosis | TNC | Weighted median | 9 | 3.03E-02 | 1.123 | 1.011 | 1.247 |
| 4155_3_TNC_Tenascin | ebi-a-GCST90018672 | Pulmonary tuberculosis | TNC | Inverse variance weighted | 9 | 3.94E-02 | 1.101 | 1.005 | 1.206 |
| 4155_3_TNC_Tenascin | ebi-a-GCST90018672 | Pulmonary tuberculosis | TNC | Simple mode | 9 | 2.12E-01 | 1.098 | 0.959 | 1.258 |
| 4155_3_TNC_Tenascin | ebi-a-GCST90018672 | Pulmonary tuberculosis | TNC | Weighted mode | 9 | 6.21E-02 | 1.115 | 1.010 | 1.230 |
| 4324_33_CST2_CYTT | ebi-a-GCST90018672 | Pulmonary tuberculosis | CST2 | MR Egger | 13 | 4.34E-01 | 1.061 | 0.920 | 1.224 |
| 4324_33_CST2_CYTT | ebi-a-GCST90018672 | Pulmonary tuberculosis | CST2 | Weighted median | 13 | 1.28E-01 | 1.082 | 0.978 | 1.197 |
| 4324_33_CST2_CYTT | ebi-a-GCST90018672 | Pulmonary tuberculosis | CST2 | Inverse variance weighted | 13 | 4.17E-02 | 1.086 | 1.003 | 1.176 |
| 4324_33_CST2_CYTT | ebi-a-GCST90018672 | Pulmonary tuberculosis | CST2 | Simple mode | 13 | 5.25E-01 | 1.065 | 0.882 | 1.287 |
| 4324_33_CST2_CYTT | ebi-a-GCST90018672 | Pulmonary tuberculosis | CST2 | Weighted mode | 13 | 1.72E-01 | 1.074 | 0.975 | 1.182 |
| 4549_78_FUT5_FUT5 | ebi-a-GCST90018672 | Pulmonary tuberculosis | FUT5 | MR Egger | 13 | 1.21E-01 | 1.060 | 0.990 | 1.133 |
| 4549_78_FUT5_FUT5 | ebi-a-GCST90018672 | Pulmonary tuberculosis | FUT5 | Weighted median | 13 | 3.21E-02 | 1.059 | 1.005 | 1.116 |
| 4549_78_FUT5_FUT5 | ebi-a-GCST90018672 | Pulmonary tuberculosis | FUT5 | Inverse variance weighted | 13 | 2.02E-02 | 1.060 | 1.009 | 1.114 |
| 4549_78_FUT5_FUT5 | ebi-a-GCST90018672 | Pulmonary tuberculosis | FUT5 | Simple mode | 13 | 3.33E-01 | 1.069 | 0.939 | 1.217 |
| 4549_78_FUT5_FUT5 | ebi-a-GCST90018672 | Pulmonary tuberculosis | FUT5 | Weighted mode | 13 | 7.14E-02 | 1.057 | 1.000 | 1.117 |
| 4703_87_LTA_TNF_b | ebi-a-GCST90018672 | Pulmonary tuberculosis | LTA | MR Egger | 3 | 3.95E-01 | 0.290 | 0.051 | 1.646 |
| 4703_87_LTA_TNF_b | ebi-a-GCST90018672 | Pulmonary tuberculosis | LTA | Weighted median | 3 | 1.18E-01 | 0.700 | 0.447 | 1.095 |
| 4703_87_LTA_TNF_b | ebi-a-GCST90018672 | Pulmonary tuberculosis | LTA | Inverse variance weighted | 3 | 4.06E-02 | 0.578 | 0.342 | 0.977 |
| 4703_87_LTA_TNF_b | ebi-a-GCST90018672 | Pulmonary tuberculosis | LTA | Simple mode | 3 | 5.07E-01 | 0.747 | 0.366 | 1.525 |
| 4703_87_LTA_TNF_b | ebi-a-GCST90018672 | Pulmonary tuberculosis | LTA | Weighted mode | 3 | 6.02E-01 | 0.803 | 0.398 | 1.620 |
| 4968_50_CAPG_CAPG | ebi-a-GCST90018672 | Pulmonary tuberculosis | CAPG | MR Egger | 3 | 4.16E-01 | 1.442 | 0.833 | 2.496 |
| 4968_50_CAPG_CAPG | ebi-a-GCST90018672 | Pulmonary tuberculosis | CAPG | Weighted median | 3 | 4.53E-02 | 1.406 | 1.007 | 1.964 |
| 4968_50_CAPG_CAPG | ebi-a-GCST90018672 | Pulmonary tuberculosis | CAPG | Inverse variance weighted | 3 | 2.04E-02 | 1.453 | 1.060 | 1.992 |
| 4968_50_CAPG_CAPG | ebi-a-GCST90018672 | Pulmonary tuberculosis | CAPG | Simple mode | 3 | 3.20E-01 | 1.340 | 0.865 | 2.078 |
| 4968_50_CAPG_CAPG | ebi-a-GCST90018672 | Pulmonary tuberculosis | CAPG | Weighted mode | 3 | 2.03E-01 | 1.398 | 0.983 | 1.987 |
| 4992_49_GRN_GRN | ebi-a-GCST90018672 | Pulmonary tuberculosis | GRN | MR Egger | 12 | 7.12E-01 | 1.030 | 0.885 | 1.198 |
| 4992_49_GRN_GRN | ebi-a-GCST90018672 | Pulmonary tuberculosis | GRN | Weighted median | 12 | 5.22E-01 | 1.036 | 0.929 | 1.156 |
| 4992_49_GRN_GRN | ebi-a-GCST90018672 | Pulmonary tuberculosis | GRN | Inverse variance weighted | 12 | 4.17E-02 | 1.083 | 1.003 | 1.170 |
| 4992_49_GRN_GRN | ebi-a-GCST90018672 | Pulmonary tuberculosis | GRN | Simple mode | 12 | 9.79E-01 | 1.002 | 0.838 | 1.200 |
| 4992_49_GRN_GRN | ebi-a-GCST90018672 | Pulmonary tuberculosis | GRN | Weighted mode | 12 | 9.71E-01 | 1.002 | 0.879 | 1.143 |
| 5132_71_IL27RA_TCCR | ebi-a-GCST90018672 | Pulmonary tuberculosis | IL27RA | MR Egger | 4 | 8.39E-01 | 1.068 | 0.612 | 1.865 |
| 5132_71_IL27RA_TCCR | ebi-a-GCST90018672 | Pulmonary tuberculosis | IL27RA | Weighted median | 4 | 3.97E-02 | 1.059 | 1.003 | 1.119 |
| 5132_71_IL27RA_TCCR | ebi-a-GCST90018672 | Pulmonary tuberculosis | IL27RA | Inverse variance weighted | 4 | 3.55E-02 | 1.059 | 1.004 | 1.117 |
| 5132_71_IL27RA_TCCR | ebi-a-GCST90018672 | Pulmonary tuberculosis | IL27RA | Simple mode | 4 | 5.43E-01 | 1.050 | 0.914 | 1.206 |
| 5132_71_IL27RA_TCCR | ebi-a-GCST90018672 | Pulmonary tuberculosis | IL27RA | Weighted mode | 4 | 2.04E-01 | 1.050 | 0.990 | 1.113 |
| 5178_5_PDE7A_PDE7A | ebi-a-GCST90018672 | Pulmonary tuberculosis | PDE7A | MR Egger | 3 | 7.40E-01 | 0.239 | 0.000 | 157.532 |
| 5178_5_PDE7A_PDE7A | ebi-a-GCST90018672 | Pulmonary tuberculosis | PDE7A | Weighted median | 3 | 7.06E-02 | 0.736 | 0.528 | 1.026 |
| 5178_5_PDE7A_PDE7A | ebi-a-GCST90018672 | Pulmonary tuberculosis | PDE7A | Inverse variance weighted | 3 | 3.77E-02 | 0.730 | 0.543 | 0.982 |
| 5178_5_PDE7A_PDE7A | ebi-a-GCST90018672 | Pulmonary tuberculosis | PDE7A | Simple mode | 3 | 3.66E-01 | 0.795 | 0.540 | 1.171 |
| 5178_5_PDE7A_PDE7A | ebi-a-GCST90018672 | Pulmonary tuberculosis | PDE7A | Weighted mode | 3 | 1.76E-01 | 0.725 | 0.534 | 0.985 |
| 5463_22_GAS1_GAS1 | ebi-a-GCST90018672 | Pulmonary tuberculosis | GAS1 | MR Egger | 7 | 3.42E-01 | 1.738 | 0.619 | 4.882 |
| 5463_22_GAS1_GAS1 | ebi-a-GCST90018672 | Pulmonary tuberculosis | GAS1 | Weighted median | 7 | 3.93E-01 | 1.130 | 0.853 | 1.497 |
| 5463_22_GAS1_GAS1 | ebi-a-GCST90018672 | Pulmonary tuberculosis | GAS1 | Inverse variance weighted | 7 | 2.25E-02 | 1.286 | 1.036 | 1.596 |
| 5463_22_GAS1_GAS1 | ebi-a-GCST90018672 | Pulmonary tuberculosis | GAS1 | Simple mode | 7 | 8.53E-01 | 1.038 | 0.709 | 1.522 |
| 5463_22_GAS1_GAS1 | ebi-a-GCST90018672 | Pulmonary tuberculosis | GAS1 | Weighted mode | 7 | 8.12E-01 | 1.048 | 0.724 | 1.516 |
| 5658_64_F13B_coagulation_factor_XIII_B | ebi-a-GCST90018672 | Pulmonary tuberculosis | F13B | MR Egger | 8 | 9.98E-02 | 0.861 | 0.740 | 1.001 |
| 5658_64_F13B_coagulation_factor_XIII_B | ebi-a-GCST90018672 | Pulmonary tuberculosis | F13B | Weighted median | 8 | 3.63E-03 | 0.879 | 0.806 | 0.959 |
| 5658_64_F13B_coagulation_factor_XIII_B | ebi-a-GCST90018672 | Pulmonary tuberculosis | F13B | Inverse variance weighted | 8 | 1.49E-02 | 0.883 | 0.799 | 0.976 |
| 5658_64_F13B_coagulation_factor_XIII_B | ebi-a-GCST90018672 | Pulmonary tuberculosis | F13B | Simple mode | 8 | 2.16E-01 | 0.833 | 0.639 | 1.084 |
| 5658_64_F13B_coagulation_factor_XIII_B | ebi-a-GCST90018672 | Pulmonary tuberculosis | F13B | Weighted mode | 8 | 1.56E-02 | 0.874 | 0.804 | 0.950 |
| 5731_1_SPINK6_ISK6 | ebi-a-GCST90018672 | Pulmonary tuberculosis | SPINK6 | MR Egger | 3 | 6.98E-01 | 1.027 | 0.927 | 1.139 |
| 5731_1_SPINK6_ISK6 | ebi-a-GCST90018672 | Pulmonary tuberculosis | SPINK6 | Weighted median | 3 | 4.27E-02 | 1.054 | 1.002 | 1.109 |
| 5731_1_SPINK6_ISK6 | ebi-a-GCST90018672 | Pulmonary tuberculosis | SPINK6 | Inverse variance weighted | 3 | 2.71E-02 | 1.058 | 1.006 | 1.111 |
| 5731_1_SPINK6_ISK6 | ebi-a-GCST90018672 | Pulmonary tuberculosis | SPINK6 | Simple mode | 3 | 2.70E-01 | 1.047 | 0.986 | 1.111 |
| 5731_1_SPINK6_ISK6 | ebi-a-GCST90018672 | Pulmonary tuberculosis | SPINK6 | Weighted mode | 3 | 1.90E-01 | 1.054 | 1.000 | 1.111 |
| 5810_25_TDGF1_Cripto | ebi-a-GCST90018672 | Pulmonary tuberculosis | TDGF1 | MR Egger | 13 | 4.79E-02 | 1.142 | 1.016 | 1.284 |
| 5810_25_TDGF1_Cripto | ebi-a-GCST90018672 | Pulmonary tuberculosis | TDGF1 | Weighted median | 13 | 2.16E-02 | 1.112 | 1.016 | 1.217 |
| 5810_25_TDGF1_Cripto | ebi-a-GCST90018672 | Pulmonary tuberculosis | TDGF1 | Inverse variance weighted | 13 | 3.92E-02 | 1.084 | 1.004 | 1.170 |
| 5810_25_TDGF1_Cripto | ebi-a-GCST90018672 | Pulmonary tuberculosis | TDGF1 | Simple mode | 13 | 3.81E-01 | 1.076 | 0.919 | 1.259 |
| 5810_25_TDGF1_Cripto | ebi-a-GCST90018672 | Pulmonary tuberculosis | TDGF1 | Weighted mode | 13 | 1.20E-02 | 1.142 | 1.046 | 1.247 |
| 5837_49_LIFR_LIF_sR | ebi-a-GCST90018672 | Pulmonary tuberculosis | LIFR | MR Egger | 8 | 3.99E-01 | 1.071 | 0.924 | 1.241 |
| 5837_49_LIFR_LIF_sR | ebi-a-GCST90018672 | Pulmonary tuberculosis | LIFR | Weighted median | 8 | 4.89E-02 | 1.098 | 1.000 | 1.204 |
| 5837_49_LIFR_LIF_sR | ebi-a-GCST90018672 | Pulmonary tuberculosis | LIFR | Inverse variance weighted | 8 | 2.72E-02 | 1.101 | 1.011 | 1.198 |
| 5837_49_LIFR_LIF_sR | ebi-a-GCST90018672 | Pulmonary tuberculosis | LIFR | Simple mode | 8 | 7.42E-02 | 1.180 | 1.011 | 1.377 |
| 5837_49_LIFR_LIF_sR | ebi-a-GCST90018672 | Pulmonary tuberculosis | LIFR | Weighted mode | 8 | 1.55E-01 | 1.083 | 0.982 | 1.196 |
| 6296_36_OIT3_OIT3 | ebi-a-GCST90018672 | Pulmonary tuberculosis | OIT3 | MR Egger | 3 | 9.86E-01 | 1.068 | 0.003 | 416.312 |
| 6296_36_OIT3_OIT3 | ebi-a-GCST90018672 | Pulmonary tuberculosis | OIT3 | Weighted median | 3 | 4.98E-04 | 4.185 | 1.869 | 9.369 |
| 6296_36_OIT3_OIT3 | ebi-a-GCST90018672 | Pulmonary tuberculosis | OIT3 | Inverse variance weighted | 3 | 6.06E-06 | 4.266 | 2.276 | 7.999 |
| 6296_36_OIT3_OIT3 | ebi-a-GCST90018672 | Pulmonary tuberculosis | OIT3 | Simple mode | 3 | 2.58E-01 | 3.205 | 0.746 | 13.768 |
| 6296_36_OIT3_OIT3 | ebi-a-GCST90018672 | Pulmonary tuberculosis | OIT3 | Weighted mode | 3 | 6.69E-02 | 4.202 | 1.951 | 9.047 |
| 6304_8_C1QTNF1_C1QT1 | ebi-a-GCST90018672 | Pulmonary tuberculosis | C1QTNF1 | MR Egger | 10 | 5.96E-01 | 1.129 | 0.733 | 1.741 |
| 6304_8_C1QTNF1_C1QT1 | ebi-a-GCST90018672 | Pulmonary tuberculosis | C1QTNF1 | Weighted median | 10 | 1.67E-02 | 1.222 | 1.037 | 1.440 |
| 6304_8_C1QTNF1_C1QT1 | ebi-a-GCST90018672 | Pulmonary tuberculosis | C1QTNF1 | Inverse variance weighted | 10 | 9.34E-03 | 1.185 | 1.043 | 1.347 |
| 6304_8_C1QTNF1_C1QT1 | ebi-a-GCST90018672 | Pulmonary tuberculosis | C1QTNF1 | Simple mode | 10 | 4.53E-01 | 1.095 | 0.873 | 1.372 |
| 6304_8_C1QTNF1_C1QT1 | ebi-a-GCST90018672 | Pulmonary tuberculosis | C1QTNF1 | Weighted mode | 10 | 5.73E-02 | 1.213 | 1.020 | 1.442 |
| 6321_65_PCDHGA10_PCDGA | ebi-a-GCST90018672 | Pulmonary tuberculosis | PCDHGA10 | MR Egger | 4 | 5.50E-01 | 1.342 | 0.597 | 3.015 |
| 6321_65_PCDHGA10_PCDGA | ebi-a-GCST90018672 | Pulmonary tuberculosis | PCDHGA10 | Weighted median | 4 | 6.62E-02 | 1.347 | 0.980 | 1.852 |
| 6321_65_PCDHGA10_PCDGA | ebi-a-GCST90018672 | Pulmonary tuberculosis | PCDHGA10 | Inverse variance weighted | 4 | 3.47E-02 | 1.339 | 1.021 | 1.754 |
| 6321_65_PCDHGA10_PCDGA | ebi-a-GCST90018672 | Pulmonary tuberculosis | PCDHGA10 | Simple mode | 4 | 3.29E-01 | 1.285 | 0.842 | 1.961 |
| 6321_65_PCDHGA10_PCDGA | ebi-a-GCST90018672 | Pulmonary tuberculosis | PCDHGA10 | Weighted mode | 4 | 1.45E-01 | 1.360 | 1.000 | 1.851 |
| 6383_90_TLL1_TLL1 | ebi-a-GCST90018672 | Pulmonary tuberculosis | TLL1 | MR Egger | 7 | 1.56E-01 | 1.112 | 0.982 | 1.260 |
| 6383_90_TLL1_TLL1 | ebi-a-GCST90018672 | Pulmonary tuberculosis | TLL1 | Weighted median | 7 | 4.59E-02 | 1.100 | 1.002 | 1.208 |
| 6383_90_TLL1_TLL1 | ebi-a-GCST90018672 | Pulmonary tuberculosis | TLL1 | Inverse variance weighted | 7 | 4.41E-02 | 1.100 | 1.003 | 1.208 |
| 6383_90_TLL1_TLL1 | ebi-a-GCST90018672 | Pulmonary tuberculosis | TLL1 | Simple mode | 7 | 2.94E-01 | 1.157 | 0.902 | 1.484 |
| 6383_90_TLL1_TLL1 | ebi-a-GCST90018672 | Pulmonary tuberculosis | TLL1 | Weighted mode | 7 | 1.19E-01 | 1.101 | 0.993 | 1.221 |
| 7210_25_APLP1_Amyloid_like_protein_1 | ebi-a-GCST90018672 | Pulmonary tuberculosis | APLP1 | MR Egger | 3 | 4.18E-01 | 0.729 | 0.453 | 1.174 |
| 7210_25_APLP1_Amyloid_like_protein_1 | ebi-a-GCST90018672 | Pulmonary tuberculosis | APLP1 | Weighted median | 3 | 1.71E-02 | 0.793 | 0.656 | 0.960 |
| 7210_25_APLP1_Amyloid_like_protein_1 | ebi-a-GCST90018672 | Pulmonary tuberculosis | APLP1 | Inverse variance weighted | 3 | 1.42E-02 | 0.794 | 0.661 | 0.955 |
| 7210_25_APLP1_Amyloid_like_protein_1 | ebi-a-GCST90018672 | Pulmonary tuberculosis | APLP1 | Simple mode | 3 | 3.90E-01 | 0.857 | 0.650 | 1.131 |
| 7210_25_APLP1_Amyloid_like_protein_1 | ebi-a-GCST90018672 | Pulmonary tuberculosis | APLP1 | Weighted mode | 3 | 1.46E-01 | 0.779 | 0.630 | 0.962 |
| 7923_41_SEMA4C_SEM4C | ebi-a-GCST90018672 | Pulmonary tuberculosis | SEMA4C | MR Egger | 5 | 4.52E-01 | 1.224 | 0.773 | 1.937 |
| 7923_41_SEMA4C_SEM4C | ebi-a-GCST90018672 | Pulmonary tuberculosis | SEMA4C | Weighted median | 5 | 3.00E-02 | 1.210 | 1.019 | 1.438 |
| 7923_41_SEMA4C_SEM4C | ebi-a-GCST90018672 | Pulmonary tuberculosis | SEMA4C | Inverse variance weighted | 5 | 2.80E-02 | 1.193 | 1.019 | 1.396 |
| 7923_41_SEMA4C_SEM4C | ebi-a-GCST90018672 | Pulmonary tuberculosis | SEMA4C | Simple mode | 5 | 1.62E-01 | 1.222 | 0.971 | 1.537 |
| 7923_41_SEMA4C_SEM4C | ebi-a-GCST90018672 | Pulmonary tuberculosis | SEMA4C | Weighted mode | 5 | 1.05E-01 | 1.211 | 1.012 | 1.449 |
| 7955_195_ITIH1_ITI_heavy_chain_H1 | ebi-a-GCST90018672 | Pulmonary tuberculosis | ITIH1 | MR Egger | 13 | 9.37E-02 | 0.848 | 0.710 | 1.011 |
| 7955_195_ITIH1_ITI_heavy_chain_H1 | ebi-a-GCST90018672 | Pulmonary tuberculosis | ITIH1 | Weighted median | 13 | 2.48E-01 | 0.930 | 0.821 | 1.052 |
| 7955_195_ITIH1_ITI_heavy_chain_H1 | ebi-a-GCST90018672 | Pulmonary tuberculosis | ITIH1 | Inverse variance weighted | 13 | 3.69E-02 | 0.907 | 0.828 | 0.994 |
| 7955_195_ITIH1_ITI_heavy_chain_H1 | ebi-a-GCST90018672 | Pulmonary tuberculosis | ITIH1 | Simple mode | 13 | 3.67E-01 | 0.907 | 0.740 | 1.112 |
| 7955_195_ITIH1_ITI_heavy_chain_H1 | ebi-a-GCST90018672 | Pulmonary tuberculosis | ITIH1 | Weighted mode | 13 | 2.20E-01 | 0.921 | 0.814 | 1.043 |
| 8035_6_C1orf198_CA198 | ebi-a-GCST90018672 | Pulmonary tuberculosis | C1orf198 | MR Egger | 3 | 6.66E-01 | 1.280 | 0.554 | 2.962 |
| 8035_6_C1orf198_CA198 | ebi-a-GCST90018672 | Pulmonary tuberculosis | C1orf198 | Weighted median | 3 | 2.97E-02 | 1.628 | 1.049 | 2.527 |
| 8035_6_C1orf198_CA198 | ebi-a-GCST90018672 | Pulmonary tuberculosis | C1orf198 | Inverse variance weighted | 3 | 3.55E-03 | 1.770 | 1.206 | 2.597 |
| 8035_6_C1orf198_CA198 | ebi-a-GCST90018672 | Pulmonary tuberculosis | C1orf198 | Simple mode | 3 | 2.63E-01 | 1.604 | 0.880 | 2.924 |
| 8035_6_C1orf198_CA198 | ebi-a-GCST90018672 | Pulmonary tuberculosis | C1orf198 | Weighted mode | 3 | 2.36E-01 | 1.551 | 0.928 | 2.591 |
| 8069_85_CD3E_CD3E | ebi-a-GCST90018672 | Pulmonary tuberculosis | CD3E | MR Egger | 17 | 2.94E-01 | 0.760 | 0.464 | 1.246 |
| 8069_85_CD3E_CD3E | ebi-a-GCST90018672 | Pulmonary tuberculosis | CD3E | Weighted median | 17 | 2.69E-01 | 0.907 | 0.764 | 1.078 |
| 8069_85_CD3E_CD3E | ebi-a-GCST90018672 | Pulmonary tuberculosis | CD3E | Inverse variance weighted | 17 | 4.42E-03 | 0.761 | 0.630 | 0.918 |
| 8069_85_CD3E_CD3E | ebi-a-GCST90018672 | Pulmonary tuberculosis | CD3E | Simple mode | 17 | 4.43E-01 | 0.896 | 0.681 | 1.178 |
| 8069_85_CD3E_CD3E | ebi-a-GCST90018672 | Pulmonary tuberculosis | CD3E | Weighted mode | 17 | 4.51E-01 | 0.939 | 0.802 | 1.101 |
| 8080_24_MSMP_PSMP | ebi-a-GCST90018672 | Pulmonary tuberculosis | MSMP | MR Egger | 15 | 4.24E-02 | 0.681 | 0.487 | 0.952 |
| 8080_24_MSMP_PSMP | ebi-a-GCST90018672 | Pulmonary tuberculosis | MSMP | Weighted median | 15 | 3.96E-02 | 0.845 | 0.720 | 0.992 |
| 8080_24_MSMP_PSMP | ebi-a-GCST90018672 | Pulmonary tuberculosis | MSMP | Inverse variance weighted | 15 | 2.74E-03 | 0.837 | 0.746 | 0.941 |
| 8080_24_MSMP_PSMP | ebi-a-GCST90018672 | Pulmonary tuberculosis | MSMP | Simple mode | 15 | 8.00E-01 | 0.971 | 0.775 | 1.216 |
| 8080_24_MSMP_PSMP | ebi-a-GCST90018672 | Pulmonary tuberculosis | MSMP | Weighted mode | 15 | 5.42E-02 | 0.826 | 0.691 | 0.987 |
| 8106_15_SSR1_SSRA | ebi-a-GCST90018672 | Pulmonary tuberculosis | SSR1 | MR Egger | 3 | 1.77E-01 | 0.320 | 0.169 | 0.605 |
| 8106_15_SSR1_SSRA | ebi-a-GCST90018672 | Pulmonary tuberculosis | SSR1 | Weighted median | 3 | 8.98E-03 | 0.630 | 0.446 | 0.891 |
| 8106_15_SSR1_SSRA | ebi-a-GCST90018672 | Pulmonary tuberculosis | SSR1 | Inverse variance weighted | 3 | 1.25E-02 | 0.588 | 0.388 | 0.892 |
| 8106_15_SSR1_SSRA | ebi-a-GCST90018672 | Pulmonary tuberculosis | SSR1 | Simple mode | 3 | 7.94E-01 | 0.893 | 0.423 | 1.885 |
| 8106_15_SSR1_SSRA | ebi-a-GCST90018672 | Pulmonary tuberculosis | SSR1 | Weighted mode | 3 | 6.07E-02 | 0.484 | 0.335 | 0.699 |
| 8253_2_POMGNT1_PMGT1 | ebi-a-GCST90018672 | Pulmonary tuberculosis | POMGNT1 | MR Egger | 3 | 7.35E-01 | 0.281 | 0.001 | 78.026 |
| 8253_2_POMGNT1_PMGT1 | ebi-a-GCST90018672 | Pulmonary tuberculosis | POMGNT1 | Weighted median | 3 | 6.12E-02 | 0.725 | 0.518 | 1.015 |
| 8253_2_POMGNT1_PMGT1 | ebi-a-GCST90018672 | Pulmonary tuberculosis | POMGNT1 | Inverse variance weighted | 3 | 3.67E-02 | 0.719 | 0.528 | 0.980 |
| 8253_2_POMGNT1_PMGT1 | ebi-a-GCST90018672 | Pulmonary tuberculosis | POMGNT1 | Simple mode | 3 | 3.84E-01 | 0.787 | 0.514 | 1.204 |
| 8253_2_POMGNT1_PMGT1 | ebi-a-GCST90018672 | Pulmonary tuberculosis | POMGNT1 | Weighted mode | 3 | 1.66E-01 | 0.712 | 0.522 | 0.972 |
| 8356_88_OXT_NEU1 | ebi-a-GCST90018672 | Pulmonary tuberculosis | OXT | MR Egger | 5 | 3.91E-01 | 1.090 | 0.920 | 1.291 |
| 8356_88_OXT_NEU1 | ebi-a-GCST90018672 | Pulmonary tuberculosis | OXT | Weighted median | 5 | 5.27E-02 | 1.115 | 0.999 | 1.244 |
| 8356_88_OXT_NEU1 | ebi-a-GCST90018672 | Pulmonary tuberculosis | OXT | Inverse variance weighted | 5 | 3.13E-02 | 1.118 | 1.010 | 1.238 |
| 8356_88_OXT_NEU1 | ebi-a-GCST90018672 | Pulmonary tuberculosis | OXT | Simple mode | 5 | 2.49E-01 | 1.158 | 0.936 | 1.432 |
| 8356_88_OXT_NEU1 | ebi-a-GCST90018672 | Pulmonary tuberculosis | OXT | Weighted mode | 5 | 1.04E-01 | 1.115 | 1.007 | 1.234 |
| 8778_3_NOG_Noggin | ebi-a-GCST90018672 | Pulmonary tuberculosis | NOG | MR Egger | 9 | 1.99E-01 | 1.571 | 0.841 | 2.934 |
| 8778_3_NOG_Noggin | ebi-a-GCST90018672 | Pulmonary tuberculosis | NOG | Weighted median | 9 | 2.11E-02 | 1.360 | 1.047 | 1.767 |
| 8778_3_NOG_Noggin | ebi-a-GCST90018672 | Pulmonary tuberculosis | NOG | Inverse variance weighted | 9 | 4.02E-02 | 1.258 | 1.010 | 1.567 |
| 8778_3_NOG_Noggin | ebi-a-GCST90018672 | Pulmonary tuberculosis | NOG | Simple mode | 9 | 1.23E-01 | 1.416 | 0.953 | 2.103 |
| 8778_3_NOG_Noggin | ebi-a-GCST90018672 | Pulmonary tuberculosis | NOG | Weighted mode | 9 | 1.70E-02 | 1.517 | 1.156 | 1.992 |
| 8903_1_COX6C_COX6C | ebi-a-GCST90018672 | Pulmonary tuberculosis | COX6C | MR Egger | 14 | 6.65E-01 | 0.877 | 0.491 | 1.567 |
| 8903_1_COX6C_COX6C | ebi-a-GCST90018672 | Pulmonary tuberculosis | COX6C | Weighted median | 14 | 3.63E-01 | 0.931 | 0.798 | 1.086 |
| 8903_1_COX6C_COX6C | ebi-a-GCST90018672 | Pulmonary tuberculosis | COX6C | Inverse variance weighted | 14 | 2.91E-02 | 0.777 | 0.619 | 0.975 |
| 8903_1_COX6C_COX6C | ebi-a-GCST90018672 | Pulmonary tuberculosis | COX6C | Simple mode | 14 | 4.63E-01 | 0.906 | 0.701 | 1.171 |
| 8903_1_COX6C_COX6C | ebi-a-GCST90018672 | Pulmonary tuberculosis | COX6C | Weighted mode | 14 | 6.35E-01 | 0.963 | 0.829 | 1.120 |
| 9215_117_USP25_UBP25 | ebi-a-GCST90018672 | Pulmonary tuberculosis | USP25 | MR Egger | 8 | 2.14E-01 | 1.078 | 0.970 | 1.198 |
| 9215_117_USP25_UBP25 | ebi-a-GCST90018672 | Pulmonary tuberculosis | USP25 | Weighted median | 8 | 2.85E-02 | 1.095 | 1.010 | 1.188 |
| 9215_117_USP25_UBP25 | ebi-a-GCST90018672 | Pulmonary tuberculosis | USP25 | Inverse variance weighted | 8 | 3.86E-02 | 1.088 | 1.004 | 1.179 |
| 9215_117_USP25_UBP25 | ebi-a-GCST90018672 | Pulmonary tuberculosis | USP25 | Simple mode | 8 | 6.41E-01 | 1.053 | 0.855 | 1.296 |
| 9215_117_USP25_UBP25 | ebi-a-GCST90018672 | Pulmonary tuberculosis | USP25 | Weighted mode | 8 | 6.20E-02 | 1.098 | 1.011 | 1.193 |
| 9509_4_TMEM38B_TM38B | ebi-a-GCST90018672 | Pulmonary tuberculosis | TMEM38B | MR Egger | 4 | 3.22E-01 | 0.621 | 0.304 | 1.269 |
| 9509_4_TMEM38B_TM38B | ebi-a-GCST90018672 | Pulmonary tuberculosis | TMEM38B | Weighted median | 4 | 6.67E-05 | 0.736 | 0.633 | 0.856 |
| 9509_4_TMEM38B_TM38B | ebi-a-GCST90018672 | Pulmonary tuberculosis | TMEM38B | Inverse variance weighted | 4 | 3.82E-05 | 0.741 | 0.643 | 0.855 |
| 9509_4_TMEM38B_TM38B | ebi-a-GCST90018672 | Pulmonary tuberculosis | TMEM38B | Simple mode | 4 | 2.24E-01 | 0.777 | 0.562 | 1.074 |
| 9509_4_TMEM38B_TM38B | ebi-a-GCST90018672 | Pulmonary tuberculosis | TMEM38B | Weighted mode | 4 | 2.64E-02 | 0.735 | 0.634 | 0.852 |
| 9590_10_COX7A2L_COX7R | ebi-a-GCST90018672 | Pulmonary tuberculosis | COX7A2L | MR Egger | 3 | 3.42E-01 | 4.145 | 0.789 | 21.773 |
| 9590_10_COX7A2L_COX7R | ebi-a-GCST90018672 | Pulmonary tuberculosis | COX7A2L | Weighted median | 3 | 7.33E-02 | 1.552 | 0.959 | 2.512 |
| 9590_10_COX7A2L_COX7R | ebi-a-GCST90018672 | Pulmonary tuberculosis | COX7A2L | Inverse variance weighted | 3 | 6.83E-03 | 1.685 | 1.154 | 2.458 |
| 9590_10_COX7A2L_COX7R | ebi-a-GCST90018672 | Pulmonary tuberculosis | COX7A2L | Simple mode | 3 | 4.12E-01 | 1.359 | 0.757 | 2.437 |
| 9590_10_COX7A2L_COX7R | ebi-a-GCST90018672 | Pulmonary tuberculosis | COX7A2L | Weighted mode | 3 | 3.97E-01 | 1.377 | 0.766 | 2.477 |
| 9796_4_CEL_CEL | ebi-a-GCST90018672 | Pulmonary tuberculosis | CEL | MR Egger | 3 | 4.02E-01 | 0.903 | 0.780 | 1.045 |
| 9796_4_CEL_CEL | ebi-a-GCST90018672 | Pulmonary tuberculosis | CEL | Weighted median | 3 | 3.86E-02 | 0.907 | 0.826 | 0.995 |
| 9796_4_CEL_CEL | ebi-a-GCST90018672 | Pulmonary tuberculosis | CEL | Inverse variance weighted | 3 | 3.12E-02 | 0.903 | 0.822 | 0.991 |
| 9796_4_CEL_CEL | ebi-a-GCST90018672 | Pulmonary tuberculosis | CEL | Simple mode | 3 | 8.33E-01 | 0.973 | 0.777 | 1.218 |
| 9796_4_CEL_CEL | ebi-a-GCST90018672 | Pulmonary tuberculosis | CEL | Weighted mode | 3 | 1.91E-01 | 0.908 | 0.824 | 1.001 |
| 9898_161_ARID1A_ARI1A | ebi-a-GCST90018672 | Pulmonary tuberculosis | ARID1A | MR Egger | 4 | 2.64E-01 | 0.465 | 0.175 | 1.234 |
| 9898_161_ARID1A_ARI1A | ebi-a-GCST90018672 | Pulmonary tuberculosis | ARID1A | Weighted median | 4 | 3.70E-02 | 0.668 | 0.458 | 0.976 |
| 9898_161_ARID1A_ARI1A | ebi-a-GCST90018672 | Pulmonary tuberculosis | ARID1A | Inverse variance weighted | 4 | 1.33E-02 | 0.686 | 0.509 | 0.924 |
| 9898_161_ARID1A_ARI1A | ebi-a-GCST90018672 | Pulmonary tuberculosis | ARID1A | Simple mode | 4 | 2.38E-01 | 0.651 | 0.367 | 1.154 |
| 9898_161_ARID1A_ARI1A | ebi-a-GCST90018672 | Pulmonary tuberculosis | ARID1A | Weighted mode | 4 | 1.48E-01 | 0.609 | 0.369 | 1.006 |
| 9969_8_SLC22A16_S22AG | ebi-a-GCST90018672 | Pulmonary tuberculosis | SLC22A16 | MR Egger | 12 | 3.75E-01 | 0.751 | 0.411 | 1.374 |
| 9969_8_SLC22A16_S22AG | ebi-a-GCST90018672 | Pulmonary tuberculosis | SLC22A16 | Weighted median | 12 | 1.14E-01 | 0.877 | 0.745 | 1.032 |
| 9969_8_SLC22A16_S22AG | ebi-a-GCST90018672 | Pulmonary tuberculosis | SLC22A16 | Inverse variance weighted | 12 | 9.41E-03 | 0.751 | 0.605 | 0.932 |
| 9969_8_SLC22A16_S22AG | ebi-a-GCST90018672 | Pulmonary tuberculosis | SLC22A16 | Simple mode | 12 | 2.60E-01 | 0.840 | 0.630 | 1.120 |
| 9969_8_SLC22A16_S22AG | ebi-a-GCST90018672 | Pulmonary tuberculosis | SLC22A16 | Weighted mode | 12 | 3.92E-01 | 0.929 | 0.790 | 1.092 |

Table S6: Differential proteins between the HC group and the TB0 group identified by proteomics

| Protein | Protein Description | Gene Name | TB0/HC | t test p value |
| --- | --- | --- | --- | --- |
| P02741 | C-reactive protein | CRP | 4.086 | 6.47E-04 |
| Q0ZCH9 | Immunglobulin heavy chain variable region (Fragment) |  | 3.462 | 1.27E-07 |
| A0A5C2GHL8 | IG c603_heavy_IGHV5-51_IGHD2-15_IGHJ4 (Fragment) |  | 3.121 | 2.10E-03 |
| A0A5C2GMU5 | IG c1208_light_IGKV3-20_IGKJ1 (Fragment) |  | 3.101 | 1.31E-04 |
| A0A5C2GB20 | IGH + IGL c392_light_IGKV1D-39_IGKJ1 (Fragment) |  | 2.720 | 1.13E-03 |
| A0A0U4DJF7 | Haptoglobin (Fragment) | HP | 2.691 | 1.11E-02 |
| A0A5C2FVH4 | IGL c1031_light_IGLV1-40_IGLJ1 (Fragment) |  | 2.592 | 1.71E-05 |
| A0A5C2G036 | IGL c2244_light_IGKV1-39_IGKJ4 (Fragment) |  | 2.552 | 4.18E-05 |
| A2IPI2 | HRV Fab N27-VL (Fragment) |  | 2.426 | 1.24E-05 |
| A0A5C2G9Y9 | IGL c202_light_IGKV1-9_IGKJ1 (Fragment) |  | 2.421 | 9.48E-03 |
| A0A5C2GXA1 | IG c1583_light_IGKV2D-28_IGKJ2 (Fragment) |  | 2.410 | 3.26E-02 |
| A0A5C2GCW0 | IGH + IGL c528_light_IGKV2D-28_IGKJ5 (Fragment) |  | 2.406 | 1.77E-02 |
| A0A5C2GR08 | IG c970_heavy_IGHV3-48_IGHD3-3_IGHJ6 (Fragment) |  | 2.292 | 4.12E-02 |
| A0A5C2GIR3 | IG c455_light_IGLV2-14_IGLJ3 (Fragment) |  | 2.279 | 6.97E-04 |
| A0A5C2FXY6 | IGL c1752_light_IGKV1D-13_IGKJ4 (Fragment) |  | 2.274 | 2.73E-05 |
| B7Z1F8 | "cDNA FLJ53025, highly similar to Complement C4-B" |  | 2.258 | 6.91E-03 |
| A0A5C2GCB3 | IGL c223_light_IGKV1-27_IGKJ4 (Fragment) |  | 2.240 | 2.46E-02 |
| A0A5C2G2W4 | IGL c2295_light_IGKV3-15_IGKJ3 (Fragment) |  | 2.228 | 2.68E-04 |
| O75460 | Serine/threonine-protein kinase/endoribonuclease IRE1 | ERN1 | 2.225 | 4.02E-04 |
| A0A193CHR0 | 10E8 heavy chain variable region (Fragment) |  | 2.192 | 1.00E-05 |
| A0A5C2G3L5 | IGL c2027_light_IGLV1-40_IGLJ3 (Fragment) |  | 2.187 | 1.73E-05 |
| A0A5C2FZE7 | IGL c1045_light_IGKV3-20_IGKJ1 (Fragment) |  | 2.179 | 1.71E-04 |
| A0A5C2GGJ2 | IG c379_heavy_IGHV4-31_IGHD5-12_IGHJ4 (Fragment) |  | 2.169 | 1.44E-04 |
| Q93033 | Immunoglobulin superfamily member 2 | CD101 | 2.155 | 1.48E-04 |
| A0A5C2GMH4 | IG c652_heavy_IGHV4-34_IGHD1-26_IGHJ6 (Fragment) |  | 2.135 | 1.71E-04 |
| A0A5C2GQC4 | IG c1161_heavy_IGHV5-51_IGHD3-3_IGHJ4 (Fragment) |  | 2.112 | 7.02E-03 |
| A0A384MDQ7 | Epididymis secretory sperm binding protein |  | 2.089 | 7.80E-04 |
| A0A5C2GRW5 | IG c391_heavy_IGHV4-34_IGHD5-24_IGHJ4 (Fragment) |  | 2.075 | 1.97E-02 |
| A0A5C2GUZ0 | IG c1261_light_IGKV2-24_IGKJ1 (Fragment) |  | 2.067 | 4.26E-02 |
| A0A5C2H035 | IG c1430_light_IGKV3-15_IGKJ4 (Fragment) |  | 2.054 | 4.09E-03 |
| A0A5C2GJV2 | IG c983_light_IGLV1-44_IGLJ1 (Fragment) |  | 2.037 | 3.75E-03 |
| A0A1W2PQB1 | Fc of IgG low affinity IIIa receptor isoform 1 (Fragment) | FCGR3A | 1.975 | 2.67E-07 |
| A0A5C2G328 | IGL c3678_light_IGKV1-9_IGKJ2 (Fragment) |  | 1.971 | 2.23E-04 |
| A0A5C2FX08 | IGL c1489_light_IGKV1-12_IGKJ4 (Fragment) |  | 1.966 | 4.27E-02 |
| A0A5C2GGT1 | IGH + IGL c391_light_IGKV4-1_IGKJ1 (Fragment) |  | 1.928 | 1.12E-02 |
| A0A5C2GAA6 | IGH + IGL c156_heavy_IGHV1-69_IGHD4-23_IGHJ6 (Fragment) |  | 1.922 | 2.52E-02 |
| A0A5C2GU42 | IG c1025_light_IGKV1-8_IGKJ2 (Fragment) |  | 1.918 | 2.05E-05 |
| A0A5C2GKT9 | IG c713_light_IGKV1-5_IGKJ4 (Fragment) |  | 1.910 | 1.65E-03 |
| B2R4C5 | Lysozyme | LYZ | 1.905 | 3.71E-05 |
| A0A5C2GQY4 | IG c950_heavy_IGHV4-39_IGHD2-2_IGHJ5 (Fragment) |  | 1.876 | 5.55E-04 |
| A0A5C2H036 | IG c1298_light_IGKV1-27_IGKJ1 (Fragment) |  | 1.874 | 6.62E-04 |
| A0A5C2GGS4 | IG c779_heavy_IGHV5-51_IGHD4-17_IGHJ4 (Fragment) |  | 1.862 | 4.76E-04 |
| A0A5C2GKG3 | IG c273_light_IGKV1-6_IGKJ1 (Fragment) |  | 1.842 | 1.81E-04 |
| A0A5C2G662 | IGH c466_heavy__IGHV3-20_IGHD7-27_IGHJ2 (Fragment) |  | 1.823 | 2.36E-05 |
| A0A5C2G949 | IGH + IGL c360_heavy_IGHV3-48_IGHD3-22_IGHJ6 (Fragment) |  | 1.823 | 5.64E-03 |
| A0A5C2GX30 | IG c258_light_IGKV1D-39_IGKJ2 (Fragment) |  | 1.820 | 1.42E-04 |
| A0A5C2FXH1 | IGL c295_light_IGKV1-12_IGKJ4 (Fragment) |  | 1.817 | 5.36E-04 |
| A0A5C2GY62 | IG c1843_light_IGLV1-51_IGLJ3 (Fragment) |  | 1.797 | 3.11E-06 |
| A0A5C2G0I0 | IGL c2070_light_IGKV3-20_IGKJ4 (Fragment) |  | 1.775 | 8.47E-03 |
| A0A0A0MRA3 | "Titin | TTN | 1.772 | 1.93E-03 |
| A0A5C2GKF6 | IG c35_heavy_IGHV3-7_IGHD5-24_IGHJ4 (Fragment) |  | 1.772 | 5.08E-03 |
| A0A5C2FZJ6 | IGL c1576_light_IGKV3-11_IGKJ4 (Fragment) |  | 1.752 | 2.26E-03 |
| A0A5C2G588 | IGL c2747_light_IGLV1-51_IGLJ2 (Fragment) |  | 1.748 | 3.91E-03 |
| A0A5C2GY12 | IG c538_light_IGLV6-57_IGLJ3 (Fragment) |  | 1.740 | 1.12E-02 |
| A0A5C2FYS6 | IGL c1390_light_IGLV2-23_IGLJ2 (Fragment) |  | 1.739 | 7.83E-03 |
| A0A5C2GTH5 | IG c855_light_IGKV3D-15_IGKJ5 (Fragment) |  | 1.734 | 1.10E-04 |
| A0A5C2G0S8 | IGL c2898_light_IGKV1D-39_IGKJ1 (Fragment) |  | 1.734 | 1.75E-03 |
| A0A5C2G0D8 | IGL c2639_light_IGKV3-20_IGKJ4 (Fragment) |  | 1.731 | 2.40E-02 |
| A0A5C2H0E8 | IG c1550_light_IGKV3-15_IGKJ1 (Fragment) |  | 1.727 | 3.47E-03 |
| A0A5C2GKH6 | IG c1226_heavy_IGHV3-53_IGHD5-12_IGHJ4 (Fragment) |  | 1.725 | 1.90E-02 |
| A0A5C2FXX3 | IGL c1494_light_IGKV3-15_IGKJ5 (Fragment) |  | 1.725 | 4.87E-06 |
| A0A5C2GUR1 | IG c1265_light_IGLV3-25_IGLJ3 (Fragment) |  | 1.714 | 7.87E-04 |
| A0A5C2GSJ7 | IG c290_light_IGLV3-25_IGLJ2 (Fragment) |  | 1.712 | 1.25E-03 |
| A0A5C2GQE4 | IG c100_light_IGKV1D-33_IGKJ2 (Fragment) |  | 1.709 | 4.28E-02 |
| A0A5C2GHS4 | IG c261_heavy_IGHV1-18_IGHD2-2_IGHJ4 (Fragment) |  | 1.709 | 3.95E-02 |
| A0A5C2GBT6 | IGH + IGL c612_light_IGKV3-15_IGKJ1 (Fragment) |  | 1.691 | 2.16E-04 |
| A0A5C2FYN5 | IGL c2081_light_IGKV1-17_IGKJ1 (Fragment) |  | 1.687 | 5.67E-03 |
| A0A5C2FW50 | IGL c416_light_IGKV1D-39_IGKJ2 (Fragment) |  | 1.673 | 3.36E-05 |
| A0A5C2G5W9 | IGH c107_heavy__IGHV4-59B_IGHD1-7_IGHJ4 (Fragment) |  | 1.673 | 6.22E-04 |
| A0A5C2G481 | IGL c2745_light_IGKV1-6_IGKJ1 (Fragment) |  | 1.672 | 7.77E-04 |
| A0A5C2GHB7 | IG c677_heavy_IGHV3-9_IGHD6-13_IGHJ4 (Fragment) |  | 1.670 | 6.68E-03 |
| A0A5C2FUE7 | IGL c618_light_IGKV3-20_IGKJ1 (Fragment) |  | 1.665 | 4.91E-02 |
| P19652 | Alpha-1-acid glycoprotein 2 | ORM2 | 1.660 | 3.02E-05 |
| A0A5C2GA01 | IGH c93_heavy__IGHV1-8_IGHD2-2_IGHJ5 (Fragment) |  | 1.650 | 3.67E-05 |
| A0A5C2GGL7 | IG c468_heavy_IGHV4-61_IGHD6-13_IGHJ6 (Fragment) |  | 1.638 | 4.88E-04 |
| B4E1I8 | "cDNA FLJ54228, highly similar to Leucine-rich alpha-2-glycoprotein |  | 1.636 | 5.81E-08 |
| A0A5C2G6C0 | IGH c459_heavy__IGHV4-31_IGHD3-9_IGHJ6 (Fragment) |  | 1.635 | 2.21E-02 |
| A0A5C2GL90 | IG c96_heavy_IGHV3-48_IGHD4-17_IGHJ6 (Fragment) |  | 1.633 | 4.74E-03 |
| A0A5C2FWG8 | IGL c460_light_IGLV7-43_IGLJ3 (Fragment) |  | 1.629 | 2.35E-03 |
| A0A5C2GWF1 | IG c887_heavy_IGHV4-34_IGHD4-17_IGHJ4 (Fragment) |  | 1.624 | 2.98E-02 |
| A0A5C2G773 | IGH c452_heavy__IGHV3-7_IGHD3-3_IGHJ4 (Fragment) |  | 1.619 | 2.93E-02 |
| A0A5C2GJ50 | IG c643_heavy_IGHV5-51_IGHD6-19_IGHJ3 (Fragment) |  | 1.619 | 6.83E-03 |
| Q9UGM5 | Fetuin-B | FETUB | 1.603 | 1.36E-05 |
| A0A5C2GHY3 | IG c850_light_IGKV3-20_IGKJ4 (Fragment) |  | 1.598 | 2.31E-03 |
| A0A5C2G665 | IGL c4120_light_IGKV2-30_IGKJ2 (Fragment) |  | 1.598 | 3.38E-03 |
| A0A5C2GVX4 | IG c1735_light_IGKV3-20_IGKJ2 (Fragment) |  | 1.593 | 1.02E-03 |
| B2RA39 | "cDNA, FLJ94686, highly similar to Homo sapiens complement factor H-related 5 (CFHL5), mRNA |  | 1.589 | 1.20E-06 |
| A0A5C2GCS5 | IGH + IGL c488_light_IGKV1-8_IGKJ1 (Fragment) |  | 1.588 | 3.98E-04 |
| A0A1W6IYJ8 | N90-VRC38.05 light chain variable region (Fragment) |  | 1.587 | 8.02E-04 |
| A0A5C2FWC0 | IGL c1239_light_IGLV2-23_IGLJ1 (Fragment) |  | 1.578 | 2.36E-03 |
| A0A5C2GBC6 | IGH + IGL c491_heavy_IGHV3-21_IGHD2-2_IGHJ4 (Fragment) |  | 1.577 | 1.60E-04 |
| A0A5C2GDV4 | IG c255_heavy_IGHV3-15_IGHD3-10_IGHJ4 (Fragment) |  | 1.576 | 9.35E-03 |
| A0A5C2GPI1 | IG c382_heavy_IGHV4-39_IGHD3-16_IGHJ3 (Fragment) |  | 1.575 | 2.09E-05 |
| Q92496 | Complement factor H-related protein 4 | CFHR4 | 1.573 | 9.35E-05 |
| A0A5C2GM07 | IG c1213_light_IGKV2D-28_IGKJ2 (Fragment) |  | 1.570 | 1.02E-04 |
| A0A5C2G1P1 | IGL c1387_light_IGKV4-1_IGKJ4 (Fragment) |  | 1.564 | 1.17E-04 |
| A0A2U8J8Y8 | Ig heavy chain variable region (Fragment) | IgH | 1.563 | 3.30E-03 |
| A0N5G5 | Rheumatoid factor D5 light chain (Fragment) | V-kappa-3 | 1.561 | 2.16E-03 |
| A0A5C2GFZ1 | IGL c319_light_IGKV3D-20_IGKJ5 (Fragment) |  | 1.558 | 4.82E-05 |
| A0A5C2G2M3 | IGL c2840_light_IGKV1D-39_IGKJ4 (Fragment) |  | 1.556 | 3.53E-02 |
| A0A5C2GRB7 | IG c5_light_IGKV4-1_IGKJ1 (Fragment) |  | 1.549 | 7.88E-05 |
| A0A5C2FXS9 | IGL c1020_light_IGKV3-20_IGKJ4 (Fragment) |  | 1.548 | 1.40E-04 |
| A0A5C2FZR4 | IGL c1616_light_IGLV2-11_IGLJ1 (Fragment) |  | 1.546 | 3.21E-02 |
| A0A5C2GPG4 | IG c197_heavy_IGHV4-39_IGHD3-3_IGHJ4 (Fragment) |  | 1.544 | 1.27E-03 |
| Q15166 | Serum paraoxonase/lactonase 3 | PON3 | 1.543 | 4.46E-05 |
| A0A0K0K1J1 | Epididymis secretory protein Li 2 | HEL-S-2 | 1.540 | 6.69E-03 |
| A0A5C2G4E6 | IGL c2347_light_IGKV3-20_IGKJ2 (Fragment) |  | 1.538 | 2.06E-02 |
| A0A5C2GB29 | IGH + IGL c302_heavy_IGHV3-23_IGHD3-22_IGHJ5 (Fragment) |  | 1.528 | 6.43E-04 |
| A0A5C2GNT6 | IG c1028_heavy_IGHV3-49_IGHD4-23_IGHJ6 (Fragment) |  | 1.524 | 1.41E-02 |
| A0A5C2FTY9 | IGL c448_light_IGKV3-20_IGKJ2 (Fragment) |  | 1.520 | 5.54E-03 |
| A0A5C2GK52 | IG c101_heavy_IGHV4-34_IGHD3-3_IGHJ6 (Fragment) |  | 1.519 | 7.08E-04 |
| A0A5C2GZG9 | IG c1078_light_IGKV1-27_IGKJ3 (Fragment) |  | 1.517 | 2.68E-04 |
| A0A5C2GL11 | IG c559_heavy_IGHV4-61_IGHD3-3_IGHJ3 (Fragment) |  | 1.515 | 3.42E-02 |
| A0A5C2G5F1 | IGH c150_heavy__IGHV4-31_IGHD2-15_IGHJ6 (Fragment) |  | 1.512 | 1.20E-04 |
| A0A5C2G827 | IGL c3917_light_IGKV1D-39_IGKJ4 (Fragment) |  | 1.507 | 7.38E-03 |
| A0A5C2G982 | IGL c4205_light_IGKV3-15_IGKJ3 (Fragment) |  | 1.506 | 9.24E-03 |
| A0A5C2FXL9 | IGL c1748_light_IGKV3-20_IGKJ3 (Fragment) |  | 1.505 | 2.47E-02 |
| A0A5C2G6X2 | IGL c2183_light_IGKV3-11_IGKJ4 (Fragment) |  | 1.503 | 6.91E-03 |
| A0A5C2GHA2 | IG c1208_heavy_IGHV3-30_IGHD3-3_IGHJ3 (Fragment) |  | 1.499 | 3.90E-03 |
| A0A5C2FUL5 | IGL c559_light_IGKV3-20_IGKJ4 (Fragment) |  | 1.498 | 1.00E-02 |
| A0A5C2FV99 | IGL c534_light_IGKV3-15_IGKJ3 (Fragment) |  | 1.497 | 1.58E-02 |
| A0A5C2GJT6 | IG c831_light_IGKV3-15_IGKJ4 (Fragment) |  | 1.496 | 1.19E-02 |
| A0A5C2G6N4 | IGH c317_heavy__IGHV1-46_IGHD3-22_IGHJ4 (Fragment) |  | 1.495 | 3.10E-03 |
| A0A5C2GW38 | IG c1751_heavy_IGHV3-13_IGHD3-3_IGHJ2 (Fragment) |  | 1.490 | 1.12E-03 |
| A0A5C2FXQ9 | IGL c1719_light_IGKV1-27_IGKJ2 (Fragment) |  | 1.487 | 3.06E-03 |
| A0A5C2G8T5 | IGL c225_light_IGKV3-15_IGKJ4 (Fragment) |  | 1.478 | 3.61E-02 |
| A0A5C2G3V0 | IGL c3166_light_IGKV1D-39_IGKJ4 (Fragment) |  | 1.473 | 6.28E-04 |
| P01591 | Immunoglobulin J chain | JCHAIN | 1.469 | 3.20E-04 |
| A0A5C2GIS8 | IG c220_light_IGKV1-9_IGKJ2 (Fragment) |  | 1.467 | 2.67E-02 |
| A0A5C2FTN4 | IGL c199_light_IGKV3-20_IGKJ2 (Fragment) |  | 1.461 | 1.46E-02 |
| A0A5C2GSF4 | IG c321_light_IGKV3-15_IGKJ4 (Fragment) |  | 1.457 | 4.27E-03 |
| P18428 | Lipopolysaccharide-binding protein | LBP | 1.452 | 2.51E-02 |
| A0A5C2GK17 | IGH + IGL c357_light_IGKV1D-39_IGKJ1 (Fragment) |  | 1.449 | 2.55E-03 |
| A0A5C2G246 | IGL c3358_light_IGKV1-8_IGKJ1 (Fragment) |  | 1.443 | 3.32E-02 |
| A0A5C2GBQ7 | IGL c135_light_IGKV1D-39_IGKJ1 (Fragment) |  | 1.443 | 2.03E-02 |
| A0A5C2GY46 | IG c1012_light_IGLV1-51_IGLJ2 (Fragment) |  | 1.442 | 2.27E-02 |
| A0A5C2GGH7 | IG c408_heavy_IGHV3-33_IGHD3-22_IGHJ6 (Fragment) |  | 1.441 | 1.15E-02 |
| A0A5C2G7J5 | IGL c418_light_IGLV1-51_IGLJ3 (Fragment) |  | 1.434 | 2.24E-02 |
| A0A5C2G7X1 | IGL c578_light_IGKV1-39_IGKJ2 (Fragment) |  | 1.434 | 1.41E-04 |
| A0A5C2G3R3 | IGL c3190_light_IGKV3-20_IGKJ1 (Fragment) |  | 1.432 | 3.43E-02 |
| A0A5C2GAN2 | IGH + IGL c266_light_IGKV1-27_IGKJ4 (Fragment) |  | 1.432 | 3.25E-03 |
| A0A5C2G9G4 | IGH + IGL c148_heavy_IGHV3-48_IGHD3-10_IGHJ4 (Fragment) |  | 1.429 | 2.97E-02 |
| A0A5C2GUM5 | IG c1234_light_IGKV3-20_IGKJ3 (Fragment) |  | 1.428 | 2.91E-02 |
| A0A0C4DH55 | Immunoglobulin kappa variable 3D-7 | IGKV3D-7 | 1.425 | 2.67E-02 |
| A0A5C2GFJ7 | IGH + IGL c139_light_IGKV1D-39_IGKJ4 (Fragment) |  | 1.422 | 1.95E-03 |
| P00738 | Haptoglobin | HP | 1.422 | 6.39E-05 |
| A0A5C2FYP8 | IGL c1925_light_IGKV4-1_IGKJ4 (Fragment) |  | 1.422 | 2.80E-02 |
| A0A5C2GYX9 | IG c1847_heavy_IGHV3-13_IGHD3-3_IGHJ3 (Fragment) |  | 1.417 | 3.06E-03 |
| A0A5C2GY49 | IG c568_light_IGKV3-20_IGKJ2 (Fragment) |  | 1.417 | 7.17E-03 |
| A0A5C2GAW1 | IGL c562_light_IGKV1D-39_IGKJ1 (Fragment) |  | 1.414 | 1.73E-02 |
| A0A5C2G2R3 | IGL c633_light_IGKV3-20_IGKJ4 (Fragment) |  | 1.413 | 4.38E-02 |
| A0A5C2FVV3 | IGL c764_light_IGLV1-51_IGLJ3 (Fragment) |  | 1.410 | 3.50E-02 |
| A0A5C2GUV9 | IG c1325_light_IGLV1-51_IGLJ1 (Fragment) |  | 1.410 | 1.37E-02 |
| A0A5C2FUU7 | IGL c781_light_IGLV1-47_IGLJ1 (Fragment) |  | 1.409 | 3.83E-02 |
| Q06033 | Inter-alpha-trypsin inhibitor heavy chain H3 | ITIH3 | 1.407 | 2.18E-06 |
| A0A5C2GE35 | IG c68_heavy_IGHV3-23_IGHD2-15_IGHJ4 (Fragment) |  | 1.407 | 3.54E-02 |
| A0A5C2FYB3 | IGL c2018_light_IGKV3-11_IGKJ4 (Fragment) |  | 1.402 | 8.84E-03 |
| A0A5C2G9E9 | IGH + IGL c323_heavy_IGHV4-39_IGHD4-4_IGHJ5 (Fragment) |  | 1.401 | 1.79E-03 |
| A0A5C2GNC7 | IG c678_heavy_IGHV3-23_IGHD1-14_IGHJ4 (Fragment) |  | 1.400 | 4.04E-02 |
| A0A5C2FYF1 | IGL c1196_light_IGKV1-5_IGKJ1 (Fragment) |  | 1.399 | 9.34E-04 |
| A0A2U8J8W0 | Ig heavy chain variable region (Fragment) | IgH | 1.395 | 9.57E-04 |
| A0A5C2GHU1 | IG c789_heavy_IGHV1-18_IGHD3-22_IGHJ3 (Fragment) |  | 1.395 | 3.35E-02 |
| A0A5C2GIK6 | IG c1053_heavy_IGHV3-11_IGHD5-18_IGHJ4 (Fragment) |  | 1.395 | 6.77E-05 |
| P02763 | Alpha-1-acid glycoprotein 1 | ORM1 | 1.394 | 7.21E-04 |
| A0A5C2GLK7 | IG c58_heavy_IGHV3-23_IGHD5-18_IGHJ4 (Fragment) |  | 1.393 | 9.71E-03 |
| A0A5C2GFS6 | IG c512_heavy_IGHV3-20_IGHD5-18_IGHJ5 (Fragment) |  | 1.391 | 4.17E-02 |
| A0A5C2GG34 | IG c509_heavy_IGHV4-59B_IGHD6-6_IGHJ6 (Fragment) |  | 1.391 | 3.06E-03 |
| A0A5C2GH93 | IG c1022_light_IGLV1-44_IGLJ2 (Fragment) |  | 1.389 | 3.08E-02 |
| A0A5C2GVV3 | IG c1447_light_IGKV3-20_IGKJ2 (Fragment) |  | 1.389 | 4.57E-02 |
| A0A5C2G4U1 | IGL c2537_light_IGKV3-11_IGKJ4 (Fragment) |  | 1.389 | 1.35E-02 |
| A0A5C2G9I6 | IGL c442_light_IGKV1D-39_IGKJ1 (Fragment) |  | 1.388 | 4.43E-03 |
| A0A5C2GX29 | IG c1503_light_IGKV3-20_IGKJ3 (Fragment) |  | 1.387 | 4.93E-02 |
| A0A5C2H2W5 | IG c1326_light_IGKV1-5_IGKJ1 (Fragment) |  | 1.383 | 3.78E-02 |
| C9JHR8 | Scavenger receptor cysteine-rich type 1 protein M130 | CD163 | 1.382 | 3.61E-02 |
| A0A5C2G6L6 | IGH c232_heavy__IGHV4-39_IGHD3-9_IGHJ4 (Fragment) |  | 1.382 | 2.16E-03 |
| A0A5C2FVW4 | IGL c952_light_IGKV3-15_IGKJ2 (Fragment) |  | 1.380 | 3.98E-02 |
| A0A5C2G309 | IGL c3532_light_IGLV4-69_IGLJ2 (Fragment) |  | 1.379 | 2.88E-02 |
| A0A5C2FXI4 | IGL c976_light_IGKV1D-39_IGKJ1 (Fragment) |  | 1.376 | 3.77E-03 |
| A0A5C2GD14 | IG c725_heavy_IGHV1-18_IGHD6-6_IGHJ4 (Fragment) |  | 1.375 | 1.64E-02 |
| A0A5C2G5P9 | IGH c286_heavy__IGHV1-18_IGHD3-16_IGHJ4 (Fragment) |  | 1.375 | 3.07E-02 |
| A0A5C2H3T0 | IG c1706_light_IGKV3-11_IGKJ2 (Fragment) |  | 1.374 | 4.01E-05 |
| A0A5C2FZ22 | IGL c517_light_IGKV1-39_IGKJ2 (Fragment) |  | 1.374 | 1.24E-02 |
| A0A5C2GUT4 | IG c1191_light_IGKV1-33_IGKJ2 (Fragment) |  | 1.373 | 1.15E-02 |
| A0A5C2GEW9 | IG c1133_heavy_IGHV3-33_IGHD3-3_IGHJ6 (Fragment) |  | 1.372 | 1.81E-02 |
| A0A5C2FYU6 | IGL c825_light_IGKV4-1_IGKJ4 (Fragment) |  | 1.372 | 4.99E-02 |
| A0A5C2GK68 | IG c744_light_IGLV1-51_IGLJ2 (Fragment) |  | 1.371 | 2.46E-03 |
| A0A5C2GBQ8 | IGH + IGL c482_heavy_IGHV1-69_IGHD4-23_IGHJ3 (Fragment) |  | 1.371 | 4.03E-02 |
| A0A5C2GI34 | IG c798_light_IGKV1-5_IGKJ2 (Fragment) |  | 1.366 | 3.17E-02 |
| A0A5C2G8U7 | IGL c256_light_IGKV3-15_IGKJ1 (Fragment) |  | 1.365 | 1.46E-02 |
| A0A5C2FZV6 | IGL c1205_light_IGKV3-15_IGKJ2 (Fragment) |  | 1.364 | 3.98E-02 |
| A0A5C2G2P9 | IGL c2225_light_IGLV1-47_IGLJ2 (Fragment) |  | 1.361 | 2.51E-03 |
| A0A5C2GFG5 | IGH + IGL c629_heavy_IGHV1-69_IGHD2-21_IGHJ6 (Fragment) |  | 1.360 | 1.71E-02 |
| A0A5C2G6E2 | IGL c3217_light_IGKV1-9_IGKJ4 (Fragment) |  | 1.358 | 4.77E-02 |
| A0A5C2FWQ3 | IGL c550_light_IGKV1-39_IGKJ4 (Fragment) |  | 1.356 | 9.53E-04 |
| A0A5C2G3Z3 | IGL c4018_light_IGLV3-19_IGLJ3 (Fragment) |  | 1.356 | 3.34E-02 |
| A0A5C2GI00 | IG c653_light_IGKV1D-17_IGKJ4 (Fragment) |  | 1.355 | 3.73E-02 |
| A0A5C2GS95 | IG c531_heavy_IGHV4-34_IGHD6-13_IGHJ4 (Fragment) |  | 1.355 | 1.26E-03 |
| A0A5C2GZT5 | IG c1909_light_IGKV2D-28_IGKJ3 (Fragment) |  | 1.354 | 1.74E-02 |
| A0A5C2G9I5 | IGL c516_light_IGKV1D-39_IGKJ4 (Fragment) |  | 1.353 | 9.89E-03 |
| A0A5C2GCF7 | IGL c4013_light_IGLV3-1_IGLJ1 (Fragment) |  | 1.353 | 1.44E-02 |
| A0A5C2GP39 | IG c593_heavy_IGHV3-48_IGHD3-3_IGHJ4 (Fragment) |  | 1.352 | 2.55E-02 |
| A0A5C2GUV0 | IG c652_light_IGLV1-40_IGLJ2 (Fragment) |  | 1.352 | 7.31E-03 |
| A0A5C2G261 | IGL c1557_light_IGLV1-40_IGLJ3 (Fragment) |  | 1.349 | 2.01E-02 |
| A0A5C2G137 | IGL c2869_light_IGKV1-12_IGKJ5 (Fragment) |  | 1.348 | 2.94E-02 |
| A0A5C2G711 | IGL c224_light_IGKV3-11_IGKJ4 (Fragment) |  | 1.347 | 4.02E-02 |
| A0A5C2GK65 | IGH + IGL c407_light_IGKV3-20_IGKJ2 (Fragment) |  | 1.344 | 3.42E-02 |
| A0A5C2G8M5 | IGL c166_light_IGKV3-20_IGKJ3 (Fragment) |  | 1.343 | 2.49E-02 |
| A0A5C2G4N9 | IGL c3874_light_IGKV1D-39_IGKJ2 (Fragment) |  | 1.342 | 5.15E-03 |
| A0A5C2GKS1 | IG c122_heavy_IGHV1-18_IGHD1-7_IGHJ4 (Fragment) |  | 1.338 | 7.11E-04 |
| A0A5C2G1N8 | IGL c2376_light_IGKV1-39_IGKJ2 (Fragment) |  | 1.337 | 2.58E-02 |
| A0A5C2G0Y8 | IGL c1137_light_IGKV1-39_IGKJ1 (Fragment) |  | 1.335 | 2.09E-03 |
| A0A5C2FZ58 | IGL c1964_light_IGKV1-39_IGKJ5 (Fragment) |  | 1.334 | 1.81E-03 |
| A0A5C2G267 | IGL c3259_light_IGKV3-11_IGKJ5 (Fragment) |  | 1.334 | 2.85E-02 |
| A0A5C2G0X5 | IGL c2948_light_IGKV3-15_IGKJ3 (Fragment) |  | 1.334 | 5.46E-03 |
| A0A5C2GQX1 | IG c289_heavy_IGHV4-34_IGHD3-3_IGHJ4 (Fragment) |  | 1.333 | 5.95E-03 |
| A0A5C2G542 | IGH c10_heavy__IGHV5-51_IGHD5-18_IGHJ6 (Fragment) |  | 1.333 | 1.66E-02 |
| A0A5C2GD99 | IGH + IGL c563_light_IGKV3-15_IGKJ1 (Fragment) |  | 1.332 | 4.42E-02 |
| A0A5C2GJ67 | IGH + IGL c47_light_IGLV3-19_IGLJ1 (Fragment) |  | 1.331 | 2.32E-03 |
| A0A5C2FYH6 | IGL c2011_light_IGKV1D-33_IGKJ4 (Fragment) |  | 1.331 | 7.88E-04 |
| P0C0L4 | Complement C4-A | C4A | 1.330 | 2.56E-06 |
| A0A5C2G229 | IGL c2506_light_IGKV1-33_IGKJ4 (Fragment) |  | 1.330 | 9.23E-03 |
| Q14520 | Hyaluronan-binding protein 2 | HABP2 | 1.328 | 1.15E-06 |
| A0A5C2GFC3 | IG c332_heavy_IGHV3-7_IGHD2-15_IGHJ1 (Fragment) |  | 1.328 | 3.05E-02 |
| A0A024R035 | "Complement component 9, isoform CRA_a | C9 | 1.327 | 6.78E-05 |
| A0A5C2G719 | IGL c234_light_IGKV3D-20_IGKJ1 (Fragment) |  | 1.326 | 7.98E-03 |
| A0A5C2G6Z4 | IGL c107_light_IGKV1D-39_IGKJ1 (Fragment) |  | 1.325 | 2.79E-02 |
| A0A5C2GA62 | IGH c153_heavy__IGHV1-2_IGHD3-9_IGHJ3 (Fragment) |  | 1.324 | 2.92E-02 |
| A0A5C2GC00 | IGH + IGL c93_light_IGKV1-5_IGKJ1 (Fragment) |  | 1.322 | 7.42E-03 |
| A0A5C2GRQ7 | IG c91_light_IGKV1-27_IGKJ4 (Fragment) |  | 1.321 | 5.56E-03 |
| A0A5C2G441 | IGL c4061_light_IGKV1-39_IGKJ1 (Fragment) |  | 1.320 | 2.34E-02 |
| A0A5C2GRC6 | IG c15_light_IGKV3D-15_IGKJ1 (Fragment) |  | 1.320 | 4.35E-03 |
| A0A5C2GTE2 | IG c617_light_IGKV3-15_IGKJ5 (Fragment) |  | 1.318 | 1.11E-02 |
| A0A5C2GLP0 | IG c606_light_IGKV3-20_IGKJ4 (Fragment) |  | 1.317 | 2.75E-02 |
| A0A5C2GT90 | IG c674_light_IGLV1-40_IGLJ2 (Fragment) |  | 1.315 | 4.53E-02 |
| A0A5C2GIU9 | IG c78_light_IGKV1-39_IGKJ2 (Fragment) |  | 1.310 | 2.52E-03 |
| A0A5C2GU31 | IG c911_light_IGKV1-9_IGKJ2 (Fragment) |  | 1.309 | 4.33E-03 |
| P14625 | Endoplasmin | HSP90B1 | 1.308 | 1.62E-02 |
| A0A5C2G479 | IGL c3919_light_IGKV1D-39_IGKJ2 (Fragment) |  | 1.307 | 1.29E-02 |
| A0A024RDT4 | "Lymphocyte cytosolic protein 1 (L-plastin), isoform CRA_a | LCP1 | 1.306 | 1.80E-04 |
| A0A5C2GJJ4 | IG c470_light_IGKV3-20_IGKJ2 (Fragment) |  | 1.301 | 4.74E-02 |
| A0A5C2GM88 | IG c298_heavy_IGHV3-9_IGHD2-21_IGHJ4 (Fragment) |  | 1.301 | 2.21E-02 |
| A0A5C2GW15 | IG c1457_light_IGKV3-20_IGKJ1 (Fragment) |  | 1.300 | 4.76E-02 |
| A0A5C2FZF5 | IGL c2074_light_IGKV6-21_IGKJ1 (Fragment) |  | 1.298 | 1.13E-02 |
| A0A5C2H0D8 | IG c1540_light_IGKV1D-39_IGKJ4 (Fragment) |  | 1.298 | 1.36E-02 |
| A0A5C2GAR0 | IGL c512_light_IGKV1D-39_IGKJ1 (Fragment) |  | 1.297 | 1.12E-02 |
| A0A5C2GD49 | IGH + IGL c384_light_IGKV1-17_IGKJ1 (Fragment) |  | 1.294 | 5.64E-04 |
| A0A5C2G2K3 | IGL c3518_light_IGKV3-11_IGKJ1 (Fragment) |  | 1.293 | 7.65E-03 |
| A0A5C2GU90 | IG c67_heavy_IGHV3-74_IGHD1-14_IGHJ3 (Fragment) |  | 1.292 | 4.74E-02 |
| A0A5C2GAL0 | IGH + IGL c112_heavy_IGHV3-23_IGHD5-24_IGHJ6 (Fragment) |  | 1.292 | 4.82E-02 |
| A0A5C2G8L6 | IGL c370_light_IGKV1D-39_IGKJ1 (Fragment) |  | 1.291 | 2.25E-02 |
| A0A5C2GDX7 | IG c834_heavy_IGHV3-20_IGHD1-26_IGHJ6 (Fragment) |  | 1.291 | 2.74E-03 |
| A0A5C2GVL8 | IG c1541_light_IGKV1-12_IGKJ1 (Fragment) |  | 1.290 | 1.54E-02 |
| Q9UL92 | Myosin-reactive immunoglobulin heavy chain variable region (Fragment) |  | 1.290 | 1.55E-02 |
| A0A5C2GDM6 | IGH + IGL c19_heavy_IGHV1-18_IGHD3-10_IGHJ5 (Fragment) |  | 1.290 | 4.49E-02 |
| A0A5C2FY46 | IGL c1564_light_IGKV4-1_IGKJ4 (Fragment) |  | 1.288 | 2.42E-02 |
| A0A5C2G298 | IGL c3272_light_IGKV3-15_IGKJ2 (Fragment) |  | 1.287 | 3.01E-02 |
| A0A5C2GAZ1 | IGH + IGL c351_heavy_IGHV3-15_IGHD5-18_IGHJ4 (Fragment) |  | 1.287 | 2.53E-02 |
| A0A5C2GKK0 | IGH + IGL c567_light_IGLV1-40_IGLJ3 (Fragment) |  | 1.286 | 1.60E-02 |
| A0A5C2GZV7 | IG c1939_light_IGLV1-47_IGLJ3 (Fragment) |  | 1.285 | 1.56E-02 |
| A0A5C2GA64 | IGH c155_heavy__IGHV3-33_IGHD1-20_IGHJ6 (Fragment) |  | 1.283 | 2.99E-02 |
| A2J1M3 | Rheumatoid factor RF-ET5 (Fragment) |  | 1.275 | 1.40E-02 |
| A0A5C2GRG0 | IG c1788_heavy_IGHV1-46_IGHD5-18_IGHJ3 (Fragment) |  | 1.274 | 1.13E-02 |
| A0A5C2GER5 | IG c1063_heavy_IGHV3-33_IGHD1-1_IGHJ3 (Fragment) |  | 1.273 | 4.43E-02 |
| A0A5C2GHF4 | IG c486_light_IGKV3-20_IGKJ2 (Fragment) |  | 1.272 | 1.50E-03 |
| A0A5C2GCD9 | IGL c253_light_IGKV3-11_IGKJ3 (Fragment) |  | 1.268 | 1.41E-02 |
| A0A5C2GFT0 | IG c1167_heavy_IGHV1-18_IGHD7-27_IGHJ4 (Fragment) |  | 1.266 | 4.36E-03 |
| P0C0L5 | Complement C4-B | C4B | 1.266 | 3.59E-02 |
| A0A5C2GL81 | IG c325_heavy_IGHV5-51_IGHD5-24_IGHJ4 (Fragment) |  | 1.266 | 1.26E-02 |
| Q65ZC9 | Single-chain Fv (Fragment) | scFv | 1.265 | 1.40E-02 |
| A0A5C2GJE3 | IG c985_light_IGKV3-15_IGKJ1 (Fragment) |  | 1.263 | 2.40E-02 |
| A0A5C2G8N3 | IGL c4067_light_IGKV1-39_IGKJ1 (Fragment) |  | 1.262 | 4.69E-03 |
| A0A5C2GIR6 | IGH + IGL c595_heavy_IGHV3-72_IGHD5-18_IGHJ4 (Fragment) |  | 1.261 | 2.16E-02 |
| A0A5C2GMV9 | IG c217_heavy_IGHV3-23_IGHD6-25_IGHJ4 (Fragment) |  | 1.261 | 4.45E-02 |
| A0A5C2GA74 | IGH + IGL c21_heavy_IGHV4-59_IGHD3-3_IGHJ3 (Fragment) |  | 1.261 | 2.12E-02 |
| A0A5C2G178 | IGL c3021_light_IGKV1D-39_IGKJ2 (Fragment) |  | 1.261 | 2.52E-02 |
| A0A5C2GK63 | IG c306_heavy_IGHV4-34_IGHD3-16_IGHJ1 (Fragment) |  | 1.258 | 2.99E-02 |
| A0A5C2GFK5 | IGH + IGL c21_light_IGKV4-1_IGKJ1 (Fragment) |  | 1.255 | 3.18E-02 |
| Q7M4S4 | Granulocyte inhibitory protein |  | 1.255 | 4.15E-02 |
| A0A5C2G896 | IGH c154_heavy__IGHV5-51_IGHD3-16_IGHJ4 (Fragment) |  | 1.254 | 1.16E-02 |
| A0A5C2GZ92 | IG c1689_light_IGLV3-21_IGLJ1 (Fragment) |  | 1.253 | 1.30E-02 |
| A0A5C2GQI9 | IG c1656_heavy_IGHV4-61_IGHD1-26_IGHJ5 (Fragment) |  | 1.253 | 2.77E-02 |
| A0A5C2GE48 | IGH c271_heavy__IGHV4-59_IGHD2-21_IGHJ6 (Fragment) |  | 1.250 | 1.46E-03 |
| A0A5C2GXS3 | IG c510_light_IGKV3-11_IGKJ5 (Fragment) |  | 1.250 | 1.36E-02 |
| A0A5C2FWX9 | IGL c1459_light_IGKV1-33_IGKJ3 (Fragment) |  | 1.250 | 3.25E-02 |
| A0A0G2JSC0 | Immunoglobulin lambda variable 5-45 (Fragment) | IGLV5-45 | 1.245 | 3.59E-02 |
| A0A5C2GKZ2 | IG c692_heavy_IGHV4-34_IGHD6-13_IGHJ6 (Fragment) |  | 1.243 | 4.74E-02 |
| A0A5C2GGG9 | IG c256_heavy_IGHV3-33_IGHD3-9_IGHJ6 (Fragment) |  | 1.240 | 1.36E-02 |
| A0A5C2G228 | IGL c2005_light_IGKV3-11_IGKJ4 (Fragment) |  | 1.239 | 1.16E-02 |
| A0A5C2G5X1 | IGL c3067_light_IGKV4-1_IGKJ4 (Fragment) |  | 1.234 | 1.92E-02 |
| A5PL27 | CP protein | CP | 1.231 | 5.77E-06 |
| A0A5C2GM68 | IG c201_light_IGKV3-15_IGKJ2 (Fragment) |  | 1.231 | 1.72E-02 |
| A0A5C2FYB1 | IGL c1156_light_IGKV3-20_IGKJ2 (Fragment) |  | 1.231 | 3.71E-02 |
| A0A5C2GAI3 | IGL c432_light_IGKV1-5_IGKJ1 (Fragment) |  | 1.227 | 4.56E-02 |
| A0A5C2GHM6 | IG c322_heavy_IGHV4-31_IGHD1-14_IGHJ4 (Fragment) |  | 1.225 | 1.60E-02 |
| A0A5C2GWD7 | IG c759_light_IGKV1D-33_IGKJ3 (Fragment) |  | 1.224 | 2.30E-02 |
| A0A5C2GI31 | IG c1191_heavy_IGHV4-39_IGHD5-12_IGHJ4 (Fragment) |  | 1.224 | 3.77E-03 |
| A0A5C2GT39 | IG c1660_heavy_IGHV4-34_IGHD5-5_IGHJ6 (Fragment) |  | 1.224 | 3.88E-03 |
| A0A5C2FV09 | IGL c719_light_IGKV1D-13_IGKJ4 (Fragment) |  | 1.223 | 1.68E-02 |
| A0A5C2GGQ6 | IGH + IGL c7_heavy_IGHV4-31_IGHD2-8_IGHJ4 (Fragment) |  | 1.223 | 4.44E-03 |
| A0A5C2GES2 | IGH c541_heavy__IGHV3-21_IGHD2-8_IGHJ4 (Fragment) |  | 1.222 | 4.13E-02 |
| P02675 | Fibrinogen beta chain | FGB | 1.221 | 8.50E-04 |
| B4E1B2 | "cDNA FLJ53691, highly similar to Serotransferrin" |  | 1.220 | 3.06E-02 |
| A0A5C2G9T9 | IGH + IGL c600_heavy_IGHV3-23_IGHD3-3_IGHJ3 (Fragment) |  | 1.217 | 4.02E-02 |
| A0A5C2GH52 | IGH + IGL c65_heavy_IGHV5-51_IGHD5-24_IGHJ5 (Fragment) |  | 1.217 | 2.29E-02 |
| A0A5C2G2Z0 | IGL c1787_light_IGKV1D-17_IGKJ2 (Fragment) |  | 1.214 | 5.77E-03 |
| A0A5C2G7V9 | IGL c100_light_IGKV1-39_IGKJ2 (Fragment) |  | 1.213 | 2.46E-02 |
| A0A5C2GN95 | IG c336_light_IGKV1D-39_IGKJ2 (Fragment) |  | 1.210 | 3.84E-02 |
| A0A5C2GG94 | IGL c439_light_IGKV3-15_IGKJ4 (Fragment) |  | 1.209 | 1.20E-02 |
| A0A5C2FZ15 | IGL c2139_light_IGKV1D-39_IGKJ4 (Fragment) |  | 1.208 | 2.80E-02 |
| A0A5C2H086 | IG c346_light_IGKV3-15_IGKJ3 (Fragment) |  | 1.201 | 3.78E-02 |
| A0A5C2GFT4 | IG c1239_heavy_IGHV3-20_IGHD4-17_IGHJ4 (Fragment) |  | 0.831 | 4.14E-03 |
| A0A024R6N9 | "Serpin peptidase inhibitor, clade A (Alpha-1 antiproteinase, antitrypsin), member 5, isoform CRA_a | SERPINA5 | 0.830 | 8.84E-03 |
| A0A5C2G3L8 | IGL c3086_light_IGKV3-15_IGKJ4 (Fragment) |  | 0.830 | 2.00E-02 |
| G3GAU4 | Anti-H1N1 influenza HA kappa chain variable region (Fragment) |  | 0.828 | 2.58E-02 |
| P23470 | Receptor-type tyrosine-protein phosphatase gamma | PTPRG | 0.827 | 1.66E-02 |
| A0A5C2G7Z6 | IGH c518_heavy__IGHV1-69_IGHD3-22_IGHJ6 (Fragment) |  | 0.826 | 4.62E-02 |
| A0A1U9X793 | APOM |  | 0.826 | 6.60E-03 |
| Q68DR3 | Uncharacterized protein DKFZp779H1622 (Fragment) | DKFZp779H1622 | 0.825 | 2.06E-02 |
| A0A5C2G2B4 | IGL c3309_light_IGKV1-16_IGKJ5 (Fragment) |  | 0.825 | 4.81E-02 |
| B0AZL7 | "cDNA, FLJ79457, highly similar to Insulin-like growth factor-binding proteincomplex acid labile chain |  | 0.824 | 4.12E-04 |
| A0A5C2G3Y3 | IGL c3624_light_IGKV3-20_IGKJ1 (Fragment) |  | 0.822 | 3.35E-02 |
| A0A024R1G8 | "Apolipoprotein L, 1, isoform CRA_b | APOL1 | 0.821 | 4.61E-04 |
| P00488 | Coagulation factor XIII A chain | F13A1 | 0.821 | 5.22E-03 |
| A0A5C2GXI4 | IG c1317_heavy_IGHV3-64_IGHD2-21_IGHJ4 (Fragment) |  | 0.820 | 8.81E-03 |
| A3KPE2 | Apolipoprotein C-III | APOC3 | 0.818 | 6.05E-03 |
| A0A5C2FWN1 | IGL c1222_light_IGKV3-20_IGKJ4 (Fragment) |  | 0.817 | 6.24E-03 |
| A0A5C2G9T5 | IGL c585_light_IGKV3D-15_IGKJ2 (Fragment) |  | 0.817 | 3.95E-02 |
| A0A5C2GG83 | IG c1310_heavy_IGHV4-59_IGHD2-21_IGHJ4 (Fragment) |  | 0.815 | 3.02E-02 |
| A0A5C2GU98 | IG c77_heavy_IGHV4-39_IGHD6-13_IGHJ4 (Fragment) |  | 0.814 | 3.84E-03 |
| A0A5C2GNH1 | IG c1002_heavy_IGHV4-4_IGHD4-17_IGHJ6 (Fragment) |  | 0.813 | 3.07E-02 |
| A0A5C2GHT7 | IG c1037_heavy_IGHV3-53_IGHD5-24_IGHJ4 (Fragment) |  | 0.813 | 3.44E-02 |
| A0A5C2G0L5 | IGL c2504_light_IGLV2-23_IGLJ3 (Fragment) |  | 0.811 | 2.56E-02 |
| A0A5C2G2Y4 | IGL c2315_light_IGKV3-15_IGKJ1 (Fragment) |  | 0.810 | 3.30E-02 |
| A0A5C2GUD9 | IG c1125_light_IGKV1D-17_IGKJ1 (Fragment) |  | 0.809 | 5.30E-03 |
| A0A5C2GJR5 | IG c837_light_IGKV1-39_IGKJ2 (Fragment) |  | 0.809 | 3.57E-02 |
| A0A024R6I9 | "Serpin peptidase inhibitor, clade A (Alpha-1 antiproteinase, antitrypsin), member 4, isoform CRA_a | SERPINA4 | 0.809 | 1.15E-03 |
| A0A5C2FYJ7 | IGL c2041_light_IGLV2-14_IGLJ1 (Fragment) |  | 0.808 | 2.60E-02 |
| A0A5C2GLD4 | IG c846_light_IGKV1-5_IGKJ1 (Fragment) |  | 0.807 | 3.42E-02 |
| A0A5C2GKG9 | IG c571_heavy_IGHV3-53_IGHD7-27_IGHJ4 (Fragment) |  | 0.807 | 4.84E-02 |
| A0A5C2H117 | IG c1790_light_IGKV3-20_IGKJ4 (Fragment) |  | 0.806 | 8.46E-03 |
| P00748 | Coagulation factor XII | F12 | 0.806 | 1.45E-02 |
| A0A5C2GFE8 | IG c282_heavy_IGHV3-23_IGHD3-22_IGHJ4 (Fragment) |  | 0.804 | 2.00E-02 |
| A0A5C2GUD6 | IG c1134_light_IGKV3-15_IGKJ1 (Fragment) |  | 0.802 | 5.05E-03 |
| A0A5C2GPD5 | IG c430_heavy_IGHV3-23_IGHD4-17_IGHJ2 (Fragment) |  | 0.802 | 2.46E-03 |
| A0A140VKF3 | Testis tissue sperm-binding protein Li 70n |  | 0.801 | 3.97E-04 |
| A0A5C2G0A9 | IGL c2592_light_IGKV1-12_IGKJ5 (Fragment) |  | 0.801 | 4.68E-02 |
| A0A5C2GTH9 | IG c1510_light_IGKV3-15_IGKJ1 (Fragment) |  | 0.799 | 1.73E-02 |
| A0A5C2GMT2 | IG c481_light_IGKV1D-8_IGKJ1 (Fragment) |  | 0.796 | 9.42E-03 |
| A0A5C2H0B9 | IG c1520_light_IGKV3-15_IGKJ2 (Fragment) |  | 0.795 | 2.81E-02 |
| A0A5C2G8V1 | IGL c245_light_IGKV3-15_IGKJ1 (Fragment) |  | 0.795 | 2.90E-02 |
| A0A5C2GAK7 | IGL c462_light_IGKV3-15_IGKJ1 (Fragment) |  | 0.794 | 2.99E-02 |
| A0A5C2G9H2 | IGH + IGL c353_heavy_IGHV3-9_IGHD6-13_IGHJ4 (Fragment) |  | 0.794 | 9.55E-03 |
| A0A5C2GBG1 | IGL c51_light_IGLV3-19_IGLJ2 (Fragment) |  | 0.793 | 2.36E-02 |
| A0A5C2G5M0 | IGL c2937_light_IGKV1-16_IGKJ5 (Fragment) |  | 0.793 | 6.63E-03 |
| A0A5C2G7W1 | IGL c2513_light_IGKV1-5_IGKJ1 (Fragment) |  | 0.793 | 4.14E-02 |
| P43251 | Biotinidase | BTD | 0.793 | 4.23E-07 |
| Q13103 | Secreted phosphoprotein 24 | SPP2 | 0.791 | 1.82E-02 |
| A0A5C2GG46 | IGL c389_light_IGKV1D-39_IGKJ1 (Fragment) |  | 0.790 | 3.51E-02 |
| A0A5C2GMJ2 | IG c665_heavy_IGHV3-7_IGHD3-3_IGHJ4 (Fragment) |  | 0.789 | 6.18E-03 |
| A0A5C2GLG3 | IG c1011_light_IGKV3-20_IGKJ5 (Fragment) |  | 0.789 | 3.49E-02 |
| A0A5C2FXS1 | IGL c1434_light_IGKV1-12_IGKJ1 (Fragment) |  | 0.786 | 1.22E-03 |
| P0DOX7 | Immunoglobulin kappa light chain |  | 0.785 | 3.04E-02 |
| A0A5C2G0X9 | IGL c2624_light_IGKV3-11_IGKJ5 (Fragment) |  | 0.784 | 1.07E-02 |
| A0A5C2GPB3 | IG c410_heavy_IGHV3-11_IGHD3-3_IGHJ6 (Fragment) |  | 0.784 | 1.41E-02 |
| A0A5C2GCU8 | IGH + IGL c568_heavy_IGHV3-23_IGHD2-21_IGHJ3 (Fragment) |  | 0.783 | 1.73E-02 |
| A0A140VJR2 | Testicular tissue protein Li 138 |  | 0.781 | 1.73E-02 |
| A0A5C2G9Z0 | IGH + IGL c96_heavy_IGHV3-15_IGHD3-16_IGHJ4 (Fragment) |  | 0.779 | 4.70E-02 |
| A0A5C2GL84 | IG c258_light_IGKV1-5_IGKJ2 (Fragment) |  | 0.778 | 9.00E-03 |
| A0A3B3IUE0 | Insulin-like growth factor-binding protein 6 | IGFBP6 | 0.778 | 1.44E-02 |
| A0A5C2GU60 | IG c37_heavy_IGHV3-11_IGHD5-24_IGHJ5 (Fragment) |  | 0.776 | 2.43E-03 |
| A0A5C2GS62 | IG c491_heavy_IGHV4-39_IGHD1-14_IGHJ4 (Fragment) |  | 0.776 | 8.28E-03 |
| A0A5C2GH03 | IG c736_light_IGKV3-15_IGKJ1 (Fragment) |  | 0.774 | 3.07E-02 |
| A0A5C2GNS0 | IG c1020_heavy_IGHV3-48_IGHD2-2_IGHJ6 (Fragment) |  | 0.774 | 1.62E-03 |
| A0A5C2GE27 | IG c1244_heavy_IGHV3-21_IGHD3-22_IGHJ4 (Fragment) |  | 0.774 | 6.93E-03 |
| C9JF17 | Apolipoprotein D (Fragment) | APOD | 0.773 | 5.64E-06 |
| A0A125QYY4 | GCT-A7 heavy chain variable region (Fragment) |  | 0.772 | 2.34E-03 |
| A0A5C2FY76 | IGL c575_light_IGKV1-12_IGKJ4 (Fragment) |  | 0.771 | 8.77E-03 |
| A0A5C2G946 | IGL c335_light_IGKV1D-39_IGKJ4 (Fragment) |  | 0.771 | 4.29E-02 |
| P78369 | Claudin-10 | CLDN10 | 0.771 | 8.54E-04 |
| Q68DS3 | Uncharacterized protein DKFZp686H17246 (Fragment) | DKFZp686H17246 | 0.770 | 1.70E-02 |
| A0A5C2G2E8 | IGL c3332_light_IGKV1-5_IGKJ1 (Fragment) |  | 0.769 | 2.35E-02 |
| Q6UXB8 | Peptidase inhibitor 16 | PI16 | 0.768 | 2.53E-04 |
| B7WNR0 | Serum albumin | ALB | 0.768 | 1.23E-02 |
| A0A5C2FYD6 | IGL c1240_light_IGLV2-14_IGLJ2 (Fragment) |  | 0.768 | 3.61E-02 |
| A0A5C2GN99 | IG c1046_heavy_IGHV3-30_IGHD4-17_IGHJ3 (Fragment) |  | 0.768 | 8.82E-03 |
| P60709 | "Actin, cytoplasmic 1 | ACTB | 0.765 | 1.81E-02 |
| A0A5C2GEI8 | IGH c441_heavy__IGHV3-72_IGHD3-22_IGHJ4 (Fragment) |  | 0.765 | 5.16E-04 |
| Q6GMX3 | IGL@ protein | IGL@ | 0.764 | 1.06E-03 |
| A0A5C2GU81 | IG c867_light_IGKV3-15_IGKJ2 (Fragment) |  | 0.764 | 2.21E-02 |
| A0A5C2GAB7 | IGH + IGL c62_heavy_IGHV3-7_IGHD3-22_IGHJ5 (Fragment) |  | 0.764 | 5.26E-03 |
| A0A5C2GIY8 | IG c175_heavy_IGHV3-23_IGHD4-11_IGHJ4 (Fragment) |  | 0.763 | 4.36E-02 |
| A0A5C2GGE2 | IG c1016_heavy_IGHV3-30_IGHD3-3_IGHJ6 (Fragment) |  | 0.763 | 1.20E-02 |
| A0A5C2FUP9 | IGL c599_light_IGKV2D-28_IGKJ4 (Fragment) |  | 0.762 | 4.80E-02 |
| A0A5C2G7S1 | IGL c60_light_IGKV3-15_IGKJ1 (Fragment) |  | 0.762 | 1.18E-02 |
| P06727 | Apolipoprotein A-IV | APOA4 | 0.762 | 4.46E-04 |
| A0A5C2G9C6 | IGH + IGL c430_heavy_IGHV3-74_IGHD3-22_IGHJ4 (Fragment) |  | 0.761 | 1.80E-02 |
| A0A5C2FXR4 | IGL c1729_light_IGKV1D-39_IGKJ1 (Fragment) |  | 0.759 | 5.40E-07 |
| A0A5C2GK01 | IG c1003_heavy_IGHV3-30_IGHD5-12_IGHJ6 (Fragment) |  | 0.758 | 2.31E-02 |
| A0A5C2GE66 | IG c431_heavy_IGHV3-23_IGHD4-17_IGHJ5 (Fragment) |  | 0.757 | 4.07E-02 |
| A0A2U8J974 | Ig heavy chain variable region (Fragment) | IgH | 0.756 | 3.22E-02 |
| A0A5C2GJ22 | IG c699_heavy_IGHV3-23_IGHD3-10_IGHJ4 (Fragment) |  | 0.756 | 2.32E-02 |
| A0A087X0M8 | "Neural cell adhesion molecule L1-like protein | CHL1 | 0.754 | 6.70E-05 |
| A0A5C2GSJ8 | IG c1664_heavy_IGHV5-51_IGHD5-5_IGHJ6 (Fragment) |  | 0.753 | 3.67E-02 |
| A0A1U9X8X3 | C2 |  | 0.752 | 1.36E-03 |
| A0A5C2GAD1 | IGH + IGL c186_heavy_IGHV3-23_IGHD2-21_IGHJ4 (Fragment) |  | 0.750 | 4.31E-02 |
| A0A5C2GRJ2 | IG c1334_heavy_IGHV3-73_IGHD6-6_IGHJ4 (Fragment) |  | 0.750 | 1.35E-02 |
| A0A5C2GFE5 | IG c1015_heavy_IGHV3-7_IGHD6-13_IGHJ4 (Fragment) |  | 0.750 | 7.44E-04 |
| Q96PD5 | N-acetylmuramoyl-L-alanine amidase | PGLYRP2 | 0.750 | 1.56E-06 |
| A0A5C2GD92 | IGH + IGL c434_light_IGKV1-5_IGKJ1 (Fragment) |  | 0.750 | 8.23E-04 |
| P02753 | Retinol-binding protein 4 | RBP4 | 0.749 | 3.10E-04 |
| A0A5C2GCY6 | IGH + IGL c453_light_IGKV1D-39_IGKJ1 (Fragment) |  | 0.749 | 4.50E-04 |
| A0A5C2GEN3 | IG c374_heavy_IGHV3-30_IGHD3-9_IGHJ1 (Fragment) |  | 0.749 | 1.06E-02 |
| A0A384MEF1 | Epididymis secretory sperm binding protein |  | 0.748 | 3.92E-07 |
| A0A5C2GDF6 | IGH + IGL c27_heavy_IGHV3-33_IGHD3-22_IGHJ6 (Fragment) |  | 0.748 | 3.36E-02 |
| A0A5C2G919 | IGL c540_light_IGKV1-5_IGKJ1 (Fragment) |  | 0.747 | 2.74E-04 |
| A0A024R104 | "Contactin 1, isoform CRA_a | CNTN1 | 0.746 | 1.12E-03 |
| D6RF35 | Vitamin D-binding protein | GC | 0.745 | 4.71E-02 |
| A0A5C2GQ29 | IG c1318_heavy_IGHV6-1_IGHD4-23_IGHJ2 (Fragment) |  | 0.743 | 3.08E-03 |
| P26038 | Moesin | MSN | 0.743 | 1.36E-02 |
| A0A5C2FTW0 | IGL c4_light_IGKV1-16_IGKJ3 (Fragment) |  | 0.743 | 2.43E-03 |
| A0A5C2GCB9 | IGL c371_light_IGKV3D-20_IGKJ3 (Fragment) |  | 0.743 | 4.08E-02 |
| A0A286YFJ8 | Immunoglobulin heavy constant gamma 4 (Fragment) | IGHG4 | 0.740 | 7.75E-03 |
| A0A2Y9CYF7 | Ig heavy chain variable region (Fragment) | IgH | 0.736 | 8.64E-03 |
| A0A5C2GGR9 | IG c30_heavy_IGHV3-23_IGHD2-2_IGHJ3 (Fragment) |  | 0.736 | 2.84E-03 |
| A0A5C2FW31 | IGL c854_light_IGKV1-5_IGKJ1 (Fragment) |  | 0.732 | 1.62E-06 |
| A0A5C2G6V7 | IGL c67_light_IGKV1-5_IGKJ1 (Fragment) |  | 0.731 | 2.06E-04 |
| A0A087X1J7 | Glutathione peroxidase | GPX3 | 0.731 | 1.61E-04 |
| A6XND0 | Insulin-like growth factor-binding protein 3 | IGFBP3 | 0.731 | 8.69E-08 |
| A0A5C2FVD8 | IGL c30_light_IGKV6-21_IGKJ1 (Fragment) |  | 0.730 | 4.51E-02 |
| A0A5C2G648 | IGH c450_heavy__IGHV3-33_IGHD1-26_IGHJ4 (Fragment) |  | 0.729 | 1.16E-02 |
| A0A5C2G9L4 | IGH + IGL c494_heavy_IGHV3-9_IGHD4-11_IGHJ3 (Fragment) |  | 0.729 | 6.73E-04 |
| A0A5C2G1C7 | IGL c133_light_IGKV1D-12_IGKJ3 (Fragment) |  | 0.728 | 6.43E-04 |
| A0A5C2GE26 | IG c1327_heavy_IGHV3-21_IGHD3-3_IGHJ6 (Fragment) |  | 0.726 | 7.91E-05 |
| A0A5C2G6A9 | IGL c4180_light_IGLV2-14_IGLJ1 (Fragment) |  | 0.725 | 1.65E-02 |
| A0A0C4DH32 | Immunoglobulin heavy variable 3-20 | IGHV3-20 | 0.723 | 1.50E-03 |
| A0A5C2FWG4 | IGL c1371_light_IGKV1-27_IGKJ1 (Fragment) |  | 0.723 | 1.33E-03 |
| A0A5C2G8W6 | IGL c276_light_IGKV1-39_IGKJ2 (Fragment) |  | 0.721 | 2.20E-03 |
| E9PHK0 | Tetranectin | CLEC3B | 0.717 | 2.16E-07 |
| A0A5C2GMA1 | IG c1071_light_IGKV1-5_IGKJ2 (Fragment) |  | 0.717 | 1.28E-03 |
| A0A5C2GN07 | IG c14_heavy_IGHV3-30_IGHD6-19_IGHJ4 (Fragment) |  | 0.717 | 5.66E-03 |
| A0A5C2GWQ0 | IG c1647_light_IGKV3-15_IGKJ4 (Fragment) |  | 0.716 | 5.24E-03 |
| A8K6K4 | "cDNA FLJ77565, highly similar to Homo sapiens interleukin 1 receptor accessory protein (IL1RAP), transcript variant 1, mRNA |  | 0.716 | 1.50E-02 |
| A0A2Y9CYF4 | Ig heavy chain variable region (Fragment) | IgH | 0.715 | 7.58E-03 |
| Q6LDG4 | Complement protein (Fragment) | C2 | 0.714 | 1.90E-03 |
| A0A5C2GW97 | IG c1112_light_IGKV1-5_IGKJ1 (Fragment) |  | 0.714 | 2.25E-03 |
| A0A5C2FWX3 | IGL c1531_light_IGKV4-1_IGKJ4 (Fragment) |  | 0.711 | 5.11E-03 |
| A0A5C2GPL8 | IG c803_heavy_IGHV1-45_IGHD6-6_IGHJ3 (Fragment) |  | 0.709 | 1.39E-02 |
| A0A5C2GD84 | IGH + IGL c543_light_IGKV3-15_IGKJ2 (Fragment) |  | 0.709 | 1.28E-02 |
| A0A5C2GP67 | IG c633_heavy_IGHV3-21_IGHD6-19_IGHJ6 (Fragment) |  | 0.708 | 2.03E-02 |
| A0A5C2GRF6 | IG c1110_heavy_IGHV5-51_IGHD6-6_IGHJ4 (Fragment) |  | 0.708 | 1.56E-02 |
| P27169 | Serum paraoxonase/arylesterase 1 | PON1 | 0.708 | 5.44E-08 |
| A0A5C2GE45 | IG c742_heavy_IGHV3-23_IGHD6-19_IGHJ3 (Fragment) |  | 0.705 | 9.05E-04 |
| A0A5C2FXP9 | IGL c990_light_IGLV3-25_IGLJ2 (Fragment) |  | 0.705 | 9.57E-03 |
| A0A5C2G872 | IGL c220_light_IGKV1-5_IGKJ4 (Fragment) |  | 0.705 | 1.13E-03 |
| A0A5C2FWI6 | IGL c1014_light_IGKV3-15_IGKJ3 (Fragment) |  | 0.704 | 6.92E-03 |
| A0A5C2GGH2 | IGL c519_light_IGKV1D-39_IGKJ4 (Fragment) |  | 0.703 | 3.89E-04 |
| A0A5C2G5N2 | IGH c119_heavy__IGHV3-48_IGHD2-15_IGHJ3 (Fragment) |  | 0.702 | 5.22E-03 |
| Q9UL89 | Myosin-reactive immunoglobulin heavy chain variable region (Fragment) |  | 0.701 | 1.50E-02 |
| A0A5C2GLD0 | IG c345_heavy_IGHV3-53_IGHD1-26_IGHJ5 (Fragment) |  | 0.700 | 3.32E-04 |
| A0A5C2GWU4 | IG c1292_light_IGKV1-12_IGKJ3 (Fragment) |  | 0.699 | 1.44E-03 |
| A0A5C2G4F7 | IGL c3450_light_IGKV1-39_IGKJ1 (Fragment) |  | 0.697 | 4.22E-06 |
| A0A5C2G0U1 | IGL c1888_light_IGKV3-15_IGKJ1 (Fragment) |  | 0.695 | 8.41E-03 |
| A0A5C2G4K3 | IGL c2427_light_IGKV1-9_IGKJ2 (Fragment) |  | 0.695 | 1.32E-03 |
| A0A5C2GZS5 | IG c226_light_IGKV1-5_IGKJ2 (Fragment) |  | 0.695 | 3.02E-08 |
| A0A5C2GX83 | IG c1039_light_IGKV4-1_IGKJ2 (Fragment) |  | 0.693 | 3.91E-02 |
| A0A5C2FY75 | IGL c1842_light_IGKV1D-43_IGKJ1 (Fragment) |  | 0.693 | 4.52E-05 |
| A0A5C2GSJ9 | IG c769_heavy_IGHV3-23_IGHD6-19_IGHJ3 (Fragment) |  | 0.691 | 1.21E-02 |
| A0A5C2GCC3 | IGL c233_light_IGKV4-1_IGKJ4 (Fragment) |  | 0.689 | 9.53E-03 |
| A0A5C2GZA8 | IG c1709_light_IGKV3-15_IGKJ5 (Fragment) |  | 0.688 | 4.61E-03 |
| A0A5C2G1B8 | IGL c1267_light_IGKV4-1_IGKJ4 (Fragment) |  | 0.681 | 2.35E-02 |
| A0A5C2G7T7 | IGL c412_light_IGKV1D-13_IGKJ4 (Fragment) |  | 0.674 | 4.21E-10 |
| A0A5C2FZ39 | IGL c1944_light_IGKV1-39_IGKJ4 (Fragment) |  | 0.673 | 9.72E-04 |
| A0N071 | Delta globin | HBD | 0.670 | 1.61E-02 |
| A0A5C2GFW9 | IG c998_heavy_IGHV4-39_IGHD3-3_IGHJ4 (Fragment) |  | 0.670 | 1.44E-02 |
| A0A5C2G6B6 | IGH c217_heavy__IGHV3-33_IGHD6-19_IGHJ4 (Fragment) |  | 0.669 | 1.18E-03 |
| A8K104 | "cDNA FLJ78726, highly similar to Homo sapiens olfactomedin 1 (OLFM1), transcript variant 1, mRNA |  | 0.668 | 1.15E-03 |
| P07737 | Profilin-1 | PFN1 | 0.664 | 2.89E-02 |
| A0A5C2GGF6 | IG c1019_heavy_IGHV3-23_IGHD3-22_IGHJ4 (Fragment) |  | 0.661 | 3.34E-02 |
| A0A140T9C0 | Tenascin-X | TNXB | 0.660 | 1.27E-03 |
| A0A5C2FY30 | IGL c1554_light_IGKV1-27_IGKJ3 (Fragment) |  | 0.659 | 6.34E-05 |
| A0A5C2FZH0 | IGL c2094_light_IGKV4-1_IGKJ5 (Fragment) |  | 0.659 | 1.28E-04 |
| A0A5C2GE97 | IGH c331_heavy__IGHV3-74_IGHD3-9_IGHJ4 (Fragment) |  | 0.657 | 1.08E-02 |
| A0A5C2G8D0 | IGL c2703_light_IGLV3-21_IGLJ3 (Fragment) |  | 0.656 | 2.37E-02 |
| P02751 | Fibronectin | FN1 | 0.655 | 2.30E-04 |
| A0A5C2G173 | IGL c2300_light_IGKV1D-13_IGKJ4 (Fragment) |  | 0.652 | 3.78E-04 |
| A0A5C2GKG7 | IGH + IGL c527_light_IGKV1D-39_IGKJ1 (Fragment) |  | 0.652 | 6.87E-03 |
| A0A5C2GQK4 | IG c1024_heavy_IGHV3-23_IGHD6-19_IGHJ5 (Fragment) |  | 0.650 | 1.48E-04 |
| A0A5C2GV41 | IG c397_heavy_IGHV3-11_IGHD2-15_IGHJ6 (Fragment) |  | 0.648 | 1.56E-03 |
| A0A5C2GF79 | IG c495_heavy_IGHV3-53_IGHD4-4_IGHJ6 (Fragment) |  | 0.646 | 7.73E-05 |
| A0A5C2GNZ7 | IG c280_heavy_IGHV3-23_IGHD2-21_IGHJ2 (Fragment) |  | 0.643 | 1.10E-02 |
| A0A5C2FZH4 | IGL c2322_light_IGKV1-12_IGKJ3 (Fragment) |  | 0.639 | 7.25E-03 |
| A0A5C2GD23 | IG c891_heavy_IGHV1-18_IGHD6-19_IGHJ3 (Fragment) |  | 0.638 | 4.44E-05 |
| A0A5C2GBK7 | IGL c23_light_IGKV3-15_IGKJ4 (Fragment) |  | 0.636 | 6.21E-05 |
| A0A5C2GMW8 | IG c277_heavy_IGHV3-23_IGHD3-16_IGHJ4 (Fragment) |  | 0.635 | 6.76E-04 |
| A0A5C2GHT4 | IG c608_heavy_IGHV1-18_IGHD6-6_IGHJ4 (Fragment) |  | 0.630 | 9.00E-05 |
| A0A5C2G3Z0 | IGL c4011_light_IGKV1D-13_IGKJ3 (Fragment) |  | 0.630 | 2.13E-02 |
| B2RDL6 | "cDNA, FLJ96669, highly similar to Homo sapiens secreted protein, acidic, cysteine-rich (osteonectin)(SPARC), mRNA |  | 0.628 | 2.91E-02 |
| A0A5C2G176 | IGL c1227_light_IGKV3-11_IGKJ3 (Fragment) |  | 0.625 | 4.10E-02 |
| A0A384MR03 | Epididymis secretory sperm binding protein |  | 0.624 | 5.54E-03 |
| A0A5C2FY84 | IGL c1604_light_IGKV1-39_IGKJ1 (Fragment) |  | 0.613 | 1.31E-04 |
| A0A5C2GV52 | IG c1434_light_IGKV1-5_IGKJ2 (Fragment) |  | 0.612 | 1.06E-06 |
| A5YAK2 | Apolipoprotein C-IV | APOC4 | 0.611 | 2.33E-06 |
| A0A5C2GDV9 | IG c1211_heavy_IGHV3-20_IGHD6-13_IGHJ4 (Fragment) |  | 0.608 | 1.63E-04 |
| A0A5C2FYZ3 | IGL c875_light_IGKV3-20_IGKJ1 (Fragment) |  | 0.604 | 4.85E-02 |
| A0A5C2GHW7 | IG c775_light_IGLV3-21_IGLJ2 (Fragment) |  | 0.603 | 4.82E-02 |
| A6XGL1 | Transthyretin |  | 0.593 | 1.56E-10 |
| A0A5C2GHR5 | IG c336_heavy_IGHV3-53_IGHD2-8_IGHJ3 (Fragment) |  | 0.587 | 2.78E-07 |
| A0A5C2GL58 | IG c895_heavy_IGHV5-51_IGHD5-12_IGHJ3 (Fragment) |  | 0.584 | 5.92E-06 |
| H0YJW9 | Uncharacterized protein (Fragment) |  | 0.572 | 1.42E-05 |
| A0A0C4DGB6 | Serum albumin | ALB | 0.571 | 1.95E-07 |
| A0A5C2GF77 | IGH + IGL c539_heavy_IGHV5-51_IGHD4-17_IGHJ3 (Fragment) |  | 0.565 | 2.64E-03 |
| A0A5C2GAC9 | IGH + IGL c110_light_IGKV1D-39_IGKJ1 (Fragment) |  | 0.555 | 4.61E-02 |
| A0A5C2GHU5 | IGH + IGL c315_heavy_IGHV3-11_IGHD3-3_IGHJ2 (Fragment) |  | 0.552 | 3.77E-02 |
| A0PJA6 | TF protein (Fragment) | TF | 0.550 | 1.24E-05 |
| A0A0X9USK2 | MS-A6 heavy chain variable region (Fragment) |  | 0.549 | 9.55E-04 |
| Q8N6N3 | UPF0690 protein C1orf52 | C1orf52 | 0.548 | 8.59E-04 |
| B3KWB5 | "cDNA FLJ42722 fis, clone BRAMY4000277, highly similar to Alpha-1B-glycoprotein" |  | 0.536 | 1.67E-03 |
| S6BAM6 | IgG H chain |  | 0.533 | 1.31E-02 |
| A0A5C2FUJ1 | IGL c668_light_IGKV3-20_IGKJ1 (Fragment) |  | 0.532 | 3.59E-02 |
| A0A024RBS0 | "RNA helicase | DDX55 | 0.531 | 1.03E-06 |
| Q562R1 | Beta-actin-like protein 2 | ACTBL2 | 0.498 | 3.69E-03 |
| A0N7I9 | F5-20 (Fragment) | F5-20 | 0.484 | 6.38E-03 |
| A0A5C2GTY4 | IG c1186_light_IGKV3-20_IGKJ2 (Fragment) |  | 0.476 | 1.89E-02 |
| A0A5C2GMV5 | IG c775_heavy_IGHV1-69_IGHD1-26_IGHJ2 (Fragment) |  | 0.437 | 2.44E-02 |
| A0A5C2GF35 | IG c13_heavy_IGHV3-15_IGHD3-3_IGHJ4 (Fragment) |  | 0.421 | 1.68E-02 |
| A0A5C2G038 | IGL c1756_light_IGKV3-20_IGKJ1 (Fragment) |  | 0.393 | 2.96E-08 |
| A0A5C2G8A6 | IGH c164_heavy__IGHV3-11_IGHD1-26_IGHJ3 (Fragment) |  | 0.237 | 1.27E-04 |

Table S7: Gene correspondence of differential proteins between the HC group and the TB0 group

| Protein | Protein Description | Gene Name |
| --- | --- | --- |
| P02741 | C-reactive protein | CRP |
| A0A0U4DJF7 | Haptoglobin (Fragment) | HP |
| O75460 | Serine/threonine-protein kinase/endoribonuclease IRE1 | ERN1 |
| Q93033 | Immunoglobulin superfamily member 2 | CD101 |
| A0A1W2PQB1 | Fc of IgG low affinity IIIa receptor isoform 1 (Fragment) | FCGR3A |
| B2R4C5 | Lysozyme | LYZ |
| A0A0A0MRA3 | "Titin | TTN |
| P19652 | Alpha-1-acid glycoprotein 2 | ORM2 |
| Q9UGM5 | Fetuin-B | FETUB |
| Q92496 | Complement factor H-related protein 4 | CFHR4 |
| A0A2U8J8Y8 | Ig heavy chain variable region (Fragment) | IgH |
| A0N5G5 | Rheumatoid factor D5 light chain (Fragment) | V-kappa-3 |
| Q15166 | Serum paraoxonase/lactonase 3 | PON3 |
| A0A0K0K1J1 | Epididymis secretory protein Li 2 | HEL-S-2 |
| P01591 | Immunoglobulin J chain | JCHAIN |
| P18428 | Lipopolysaccharide-binding protein | LBP |
| A0A0C4DH55 | Immunoglobulin kappa variable 3D-7 | IGKV3D-7 |
| P00738 | Haptoglobin | HP |
| Q06033 | Inter-alpha-trypsin inhibitor heavy chain H3 | ITIH3 |
| A0A2U8J8W0 | Ig heavy chain variable region (Fragment) | IgH |
| P02763 | Alpha-1-acid glycoprotein 1 | ORM1 |
| C9JHR8 | Scavenger receptor cysteine-rich type 1 protein M130 | CD163 |
| P0C0L4 | Complement C4-A | C4A |
| Q14520 | Hyaluronan-binding protein 2 | HABP2 |
| A0A024R035 | "Complement component 9, isoform CRA_a | C9 |
| P14625 | Endoplasmin | HSP90B1 |
| A0A024RDT4 | "Lymphocyte cytosolic protein 1 (L-plastin), isoform CRA_a | LCP1 |
| P0C0L5 | Complement C4-B | C4B |
| Q65ZC9 | Single-chain Fv (Fragment) | scFv |
| A0A0G2JSC0 | Immunoglobulin lambda variable 5-45 (Fragment) | IGLV5-45 |
| A5PL27 | CP protein | CP |
| P02675 | Fibrinogen beta chain | FGB |
| A0A024R6N9 | "Serpin peptidase inhibitor, clade A (Alpha-1 antiproteinase, antitrypsin), member 5, isoform CRA_a | SERPINA5 |
| P23470 | Receptor-type tyrosine-protein phosphatase gamma | PTPRG |
| Q68DR3 | Uncharacterized protein DKFZp779H1622 (Fragment) | DKFZp779H1622 |
| A0A024R1G8 | "Apolipoprotein L, 1, isoform CRA_b | APOL1 |
| P00488 | Coagulation factor XIII A chain | F13A1 |
| A3KPE2 | Apolipoprotein C-III | APOC3 |
| A0A024R6I9 | "Serpin peptidase inhibitor, clade A (Alpha-1 antiproteinase, antitrypsin), member 4, isoform CRA_a | SERPINA4 |
| P00748 | Coagulation factor XII | F12 |
| P43251 | Biotinidase | BTD |
| Q13103 | Secreted phosphoprotein 24 | SPP2 |
| A0A3B3IUE0 | Insulin-like growth factor-binding protein 6 | IGFBP6 |
| C9JF17 | Apolipoprotein D (Fragment) | APOD |
| P78369 | Claudin-10 | CLDN10 |
| Q68DS3 | Uncharacterized protein DKFZp686H17246 (Fragment) | DKFZp686H17246 |
| Q6UXB8 | Peptidase inhibitor 16 | PI16 |
| B7WNR0 | Serum albumin | ALB |
| P60709 | "Actin, cytoplasmic 1 | ACTB |
| Q6GMX3 | IGL@ protein | IGL@ |
| P06727 | Apolipoprotein A-IV | APOA4 |
| A0A2U8J974 | Ig heavy chain variable region (Fragment) | IgH |
| A0A087X0M8 | "Neural cell adhesion molecule L1-like protein | CHL1 |
| Q96PD5 | N-acetylmuramoyl-L-alanine amidase | PGLYRP2 |
| P02753 | Retinol-binding protein 4 | RBP4 |
| A0A024R104 | "Contactin 1, isoform CRA_a | CNTN1 |
| D6RF35 | Vitamin D-binding protein | GC |
| P26038 | Moesin | MSN |
| A0A286YFJ8 | Immunoglobulin heavy constant gamma 4 (Fragment) | IGHG4 |
| A0A2Y9CYF7 | Ig heavy chain variable region (Fragment) | IgH |
| A0A087X1J7 | Glutathione peroxidase | GPX3 |
| A6XND0 | Insulin-like growth factor-binding protein 3 | IGFBP3 |
| A0A0C4DH32 | Immunoglobulin heavy variable 3-20 | IGHV3-20 |
| E9PHK0 | Tetranectin | CLEC3B |
| A0A2Y9CYF4 | Ig heavy chain variable region (Fragment) | IgH |
| Q6LDG4 | Complement protein (Fragment) | C2 |
| P27169 | Serum paraoxonase/arylesterase 1 | PON1 |
| A0N071 | Delta globin | HBD |
| P07737 | Profilin-1 | PFN1 |
| A0A140T9C0 | Tenascin-X | TNXB |
| P02751 | Fibronectin | FN1 |
| A5YAK2 | Apolipoprotein C-IV | APOC4 |
| A0A0C4DGB6 | Serum albumin | ALB |
| A0PJA6 | TF protein (Fragment) | TF |
| Q8N6N3 | UPF0690 protein C1orf52 | C1orf52 |
| A0A024RBS0 | "RNA helicase | DDX55 |
| Q562R1 | Beta-actin-like protein 2 | ACTBL2 |
| A0N7I9 | F5-20 (Fragment) | F5-20 |

Table S8: Differential proteins between the TB0 group and the TB6 group identified by proteomics

| Protein | Protein Description | Gene Name | TB6/TB0 | t test p value |
| --- | --- | --- | --- | --- |
| A0A5C2FV18 | IGL c729_light_IGLV1-40_IGLJ2 (Fragment) |  | 7.501 | 2.94E-02 |
| A0A5C2G038 | IGL c1756_light_IGKV3-20_IGKJ1 (Fragment) |  | 6.780 | 2.47E-03 |
| A0A5C2GU23 | IG c253_light_IGLV1-47_IGLJ2 (Fragment) |  | 6.287 | 7.11E-03 |
| A0A5C2G335 | IGL c3562_light_IGLV1-44_IGLJ3 (Fragment) |  | 5.902 | 1.20E-02 |
| A0A5C2GTY4 | IG c1186_light_IGKV3-20_IGKJ2 (Fragment) |  | 5.880 | 4.59E-04 |
| A0A5C2GQK4 | IG c1024_heavy_IGHV3-23_IGHD6-19_IGHJ5 (Fragment) |  | 5.244 | 2.47E-02 |
| A0A5C2G4V2 | IGL c1323_light_IGLV1-44_IGLJ1 (Fragment) |  | 5.036 | 7.12E-03 |
| A0A0X9TD23 | MS-A5 heavy chain variable region (Fragment) |  | 4.848 | 4.80E-02 |
| A0A5C2GUK3 | IG c1191_heavy_IGHV3-33_IGHD2-2_IGHJ4 (Fragment) |  | 4.671 | 1.78E-02 |
| A0A5H1ZRQ7 | Immunoglobulin lambda constant 7 (Fragment) | IGLC7 | 4.168 | 1.53E-04 |
| A0A5C2G922 | IGH c434_heavy__IGHV3-49_IGHD2-21_IGHJ4 (Fragment) |  | 3.766 | 2.25E-02 |
| A2JA17 | Anti-mucin1 heavy chain variable region (Fragment) |  | 3.698 | 2.54E-02 |
| Q9UL84 | Myosin-reactive immunoglobulin heavy chain variable region (Fragment) |  | 3.625 | 1.88E-02 |
| A0A5C2GCN9 | IGH + IGL c448_light_IGKV3-20_IGKJ5 (Fragment) |  | 3.497 | 9.55E-03 |
| A0A5C2GT43 | IG c999_heavy_IGHV3-30_IGHD3-22_IGHJ1 (Fragment) |  | 3.488 | 1.37E-03 |
| A0A024RAB7 | "UDP-galactose-4-epimerase, isoform CRA_b | GALE | 3.308 | 2.12E-02 |
| A0A5C2FWI6 | IGL c1014_light_IGKV3-15_IGKJ3 (Fragment) |  | 3.267 | 1.74E-03 |
| A0A5C2GWQ0 | IG c1647_light_IGKV3-15_IGKJ4 (Fragment) |  | 3.228 | 1.32E-03 |
| A0A5C2FV72 | IGL c672_light_IGKV3-20_IGKJ3 (Fragment) |  | 3.100 | 6.40E-03 |
| A0A5C2GRY3 | IG c245_light_IGKV3-20_IGKJ1 (Fragment) |  | 3.075 | 1.17E-03 |
| A0A5C2G8A6 | IGH c164_heavy__IGHV3-11_IGHD1-26_IGHJ3 (Fragment) |  | 3.029 | 1.22E-03 |
| A0A5C2G0Q9 | IGL c2831_light_IGKV1D-39_IGKJ4 (Fragment) |  | 3.005 | 2.09E-02 |
| A0A5C2FX88 | IGL c1591_light_IGKV2-30_IGKJ4 (Fragment) |  | 2.997 | 1.76E-02 |
| A0A5C2G180 | IGL c83_light_IGKV3-11_IGKJ2 (Fragment) |  | 2.934 | 1.25E-02 |
| A0A5C2FZF0 | IGL c1630_light_IGLV1-51_IGLJ3 (Fragment) |  | 2.879 | 2.42E-02 |
| A0A5C2GKN1 | IG c280_light_IGKV3-20_IGKJ2 (Fragment) |  | 2.779 | 9.76E-03 |
| A0A5C2GR38 | IG c1184_heavy_IGHV3-66_IGHD3-22_IGHJ3 (Fragment) |  | 2.721 | 3.61E-02 |
| A0A5C2GT80 | IG c711_heavy_IGHV4-59B_IGHD1-26_IGHJ6 (Fragment) |  | 2.676 | 5.28E-04 |
| A0A5C2FUJ1 | IGL c668_light_IGKV3-20_IGKJ1 (Fragment) |  | 2.647 | 3.47E-03 |
| A0A5C2GF35 | IG c13_heavy_IGHV3-15_IGHD3-3_IGHJ4 (Fragment) |  | 2.608 | 1.08E-02 |
| S6BAQ4 | IgG H chain |  | 2.605 | 3.20E-03 |
| A0A5C2FV87 | IGL c958_light_IGKV3-15_IGKJ2 (Fragment) |  | 2.534 | 3.36E-03 |
| A0A5C2GAC9 | IGH + IGL c110_light_IGKV1D-39_IGKJ1 (Fragment) |  | 2.365 | 4.97E-03 |
| A0A5C2GBR4 | IGH + IGL c178_heavy_IGHV3-33_IGHD5-12_IGHJ4 (Fragment) |  | 2.295 | 6.26E-04 |
| A0A5C2GN07 | IG c14_heavy_IGHV3-30_IGHD6-19_IGHJ4 (Fragment) |  | 2.294 | 2.57E-02 |
| A0A5C2G176 | IGL c1227_light_IGKV3-11_IGKJ3 (Fragment) |  | 2.272 | 1.07E-05 |
| A0A5C2GNM5 | IG c1072_heavy_IGHV3-33_IGHD3-3_IGHJ4 (Fragment) |  | 2.265 | 2.00E-03 |
| D6RF35 | Vitamin D-binding protein | GC | 2.207 | 2.59E-04 |
| Q6MZU6 | Uncharacterized protein DKFZp686C15213 | DKFZp686C15213 | 2.148 | 7.69E-03 |
| A0A5C2H1S8 | IG c886_light_IGLV3-25_IGLJ3 (Fragment) |  | 2.138 | 2.57E-02 |
| A0A5C2G003 | IGL c867_light_IGKV3-20_IGKJ2 (Fragment) |  | 2.131 | 1.36E-03 |
| A0A5C2GBX8 | IGH + IGL c572_heavy_IGHV1-69_IGHD6-13_IGHJ6 (Fragment) |  | 2.117 | 2.38E-02 |
| A0A5C2G221 | IGL c3321_light_IGKV3-20_IGKJ1 (Fragment) |  | 2.105 | 2.54E-03 |
| A0A5C2GAK7 | IGL c462_light_IGKV3-15_IGKJ1 (Fragment) |  | 2.059 | 8.17E-03 |
| A0A5C2G686 | IGH c506_heavy__IGHV3-43_IGHD2-15_IGHJ4 (Fragment) |  | 2.056 | 1.07E-02 |
| A0A5C2GQE6 | IG c145_heavy_IGHV4-34_IGHD3-16_IGHJ6 (Fragment) |  | 2.056 | 3.16E-02 |
| A0A5C2GEW9 | IG c1133_heavy_IGHV3-33_IGHD3-3_IGHJ6 (Fragment) |  | 2.046 | 2.54E-03 |
| A0A5C2GPY9 | IG c1465_heavy_IGHV3-48_IGHD6-6_IGHJ6 (Fragment) |  | 2.042 | 4.89E-02 |
| A0A5C2GM87 | IG c625_heavy_IGHV3-21_IGHD2-21_IGHJ3 (Fragment) |  | 2.024 | 1.68E-03 |
| A0A384N669 | Epididymis secretory sperm binding protein |  | 2.011 | 3.61E-10 |
| A0A5C2GJ53 | IG c26_heavy_IGHV3-30_IGHD3-3_IGHJ5 (Fragment) |  | 2.007 | 1.27E-03 |
| A0A5C2GPN8 | IG c1158_heavy_IGHV3-48_IGHD7-27_IGHJ6 (Fragment) |  | 1.998 | 2.23E-02 |
| A0A5C2GS71 | IG c335_light_IGKV1-9_IGKJ5 (Fragment) |  | 1.995 | 4.23E-03 |
| Q0ZCH7 | Immunglobulin heavy chain variable region (Fragment) |  | 1.981 | 1.88E-02 |
| A0A5C2GTI7 | IG c800_light_IGLV3-27_IGLJ2 (Fragment) |  | 1.981 | 1.89E-02 |
| A0N071 | Delta globin | HBD | 1.976 | 4.36E-04 |
| A0A5C2G1B8 | IGL c1267_light_IGKV4-1_IGKJ4 (Fragment) |  | 1.971 | 7.84E-04 |
| A0A5C2GPU9 | IG c1228_heavy_IGHV3-33_IGHD1-1_IGHJ4 (Fragment) |  | 1.970 | 7.62E-03 |
| A0A5C2FWT5 | IGL c138_light_IGKV2-30_IGKJ2 (Fragment) |  | 1.960 | 2.98E-03 |
| A0A5C2GRF6 | IG c1110_heavy_IGHV5-51_IGHD6-6_IGHJ4 (Fragment) |  | 1.953 | 4.51E-04 |
| A0A5C2FVZ0 | IGL c1099_light_IGLV3-1_IGLJ2 (Fragment) |  | 1.952 | 1.92E-02 |
| A0A5C2G2F7 | IGL c3104_light_IGKV3-20_IGKJ2 (Fragment) |  | 1.950 | 2.58E-02 |
| A2NYU7 | Heavy chain Fab (Fragment) |  | 1.944 | 1.61E-02 |
| A0A109PT03 | GCT-A9 heavy chain variable region (Fragment) |  | 1.930 | 2.27E-03 |
| A0A5C2G435 | IGL c1063_light_IGKV3-20_IGKJ4 (Fragment) |  | 1.923 | 2.75E-04 |
| A0A125QYY4 | GCT-A7 heavy chain variable region (Fragment) |  | 1.921 | 3.73E-02 |
| A0A5C2GVU1 | IG c1453_light_IGKV3-11_IGKJ5 (Fragment) |  | 1.911 | 4.19E-02 |
| A0A5C2GD23 | IG c891_heavy_IGHV1-18_IGHD6-19_IGHJ3 (Fragment) |  | 1.910 | 8.13E-09 |
| A0A5C2GEX7 | IG c378_heavy_IGHV3-11_IGHD4-17_IGHJ4 (Fragment) |  | 1.902 | 3.14E-02 |
| A0A5C2G9L3 | IGH c25_heavy__IGHV4-39_IGHD6-19_IGHJ6 (Fragment) |  | 1.900 | 7.09E-04 |
| A0A5C2GRG5 | IG c376_light_IGLV3-21_IGLJ2 (Fragment) |  | 1.893 | 2.29E-04 |
| A0A5C2GL66 | IG c272_heavy_IGHV3-48_IGHD2-15_IGHJ6 (Fragment) |  | 1.884 | 1.04E-02 |
| Q6ZW64 | "cDNA FLJ41552 fis, clone COLON2004478, highly similar to Protein Tro alpha1 H,myeloma" |  | 1.883 | 7.43E-04 |
| A0A5C2GVG3 | IG c842_light_IGKV1-27_IGKJ1 (Fragment) |  | 1.872 | 3.76E-02 |
| A0A5C2GHD2 | IGH + IGL c145_heavy_IGHV3-15_IGHD3-10_IGHJ4 (Fragment) |  | 1.862 | 9.10E-03 |
| A0A5C2GM94 | IG c476_heavy_IGHV3-23_IGHD3-9_IGHJ3 (Fragment) |  | 1.852 | 1.03E-04 |
| A0A5C2GW09 | IG c1641_light_IGKV1-6_IGKJ4 (Fragment) |  | 1.851 | 1.43E-03 |
| A0A5C2GTJ2 | IG c875_light_IGKV1-8_IGKJ1 (Fragment) |  | 1.846 | 5.24E-03 |
| A0A5C2G985 | IGL c375_light_IGKV3-20_IGKJ1 (Fragment) |  | 1.844 | 2.05E-02 |
| A0A5C2G1W2 | IGL c2904_light_IGKV4-1_IGKJ4 (Fragment) |  | 1.840 | 2.63E-03 |
| A0A5C2GK01 | IG c1003_heavy_IGHV3-30_IGHD5-12_IGHJ6 (Fragment) |  | 1.838 | 7.98E-04 |
| P02751 | Fibronectin | FN1 | 1.830 | 3.47E-03 |
| A0A5C2GLD5 | IG c146_heavy_IGHV3-33_IGHD4-23_IGHJ4 (Fragment) |  | 1.827 | 7.01E-03 |
| A0A3B0J0F2 | "Adiponectin D | ADID | 1.826 | 3.32E-05 |
| A0A5C2GT62 | IG c1690_heavy_IGHV3-21_IGHD5-18_IGHJ6 (Fragment) |  | 1.823 | 2.20E-02 |
| Q0ZCH9 | Immunglobulin heavy chain variable region (Fragment) |  | 1.821 | 6.20E-03 |
| B7Z8Q2 | "cDNA FLJ55606, highly similar to Alpha-2-HS-glycoprotein |  | 1.820 | 1.43E-06 |
| A0A5C2GHT4 | IG c608_heavy_IGHV1-18_IGHD6-6_IGHJ4 (Fragment) |  | 1.819 | 4.67E-06 |
| A0A5C2GY22 | IG c1517_heavy_IGHV3-33_IGHD2-2_IGHJ6 (Fragment) |  | 1.816 | 3.23E-02 |
| A0A0X9USK2 | MS-A6 heavy chain variable region (Fragment) |  | 1.816 | 3.70E-03 |
| A0A5C2GBB1 | IGH + IGL c492_light_IGKV1D-33_IGKJ2 (Fragment) |  | 1.816 | 9.85E-03 |
| A0A5C2FTC1 | IGL c69_light_IGKV3-15_IGKJ4 (Fragment) |  | 1.805 | 2.48E-02 |
| A0A5C2GPB3 | IG c410_heavy_IGHV3-11_IGHD3-3_IGHJ6 (Fragment) |  | 1.803 | 7.32E-03 |
| A0A5C2GDV9 | IG c1211_heavy_IGHV3-20_IGHD6-13_IGHJ4 (Fragment) |  | 1.803 | 4.30E-03 |
| A8K5A4 | "cDNA FLJ76826, highly similar to Homo sapiens ceruloplasmin (ferroxidase) (CP), mRNA" |  | 1.803 | 3.99E-03 |
| A0A5C2GHC2 | IG c308_heavy_IGHV3-33_IGHD3-3_IGHJ4 (Fragment) |  | 1.802 | 7.52E-04 |
| A0A5C2GQ29 | IG c1318_heavy_IGHV6-1_IGHD4-23_IGHJ2 (Fragment) |  | 1.801 | 2.43E-03 |
| A0A5C2GEM0 | IG c266_heavy_IGHV3-33_IGHD6-19_IGHJ4 (Fragment) |  | 1.797 | 9.04E-04 |
| A0A5C2GMA8 | IG c30_light_IGLV2-11_IGLJ2 (Fragment) |  | 1.796 | 3.36E-02 |
| A0A5C2GG35 | IG c776_heavy_IGHV3-23_IGHD1-1_IGHJ4 (Fragment) |  | 1.795 | 2.85E-02 |
| A0A5C2G3G9 | IGL c3828_light_IGKV3-20_IGKJ1 (Fragment) |  | 1.787 | 3.35E-02 |
| A0A193CHR0 | 10E8 heavy chain variable region (Fragment) |  | 1.787 | 2.65E-02 |
| A0A5C2FWZ4 | IGL c65_light_IGKV3-20_IGKJ5 (Fragment) |  | 1.786 | 3.62E-03 |
| A0A5C2GAZ1 | IGH + IGL c351_heavy_IGHV3-15_IGHD5-18_IGHJ4 (Fragment) |  | 1.778 | 4.33E-03 |
| A0A5C2GP62 | IG c1028_heavy_IGHV1-18_IGHD2-21_IGHJ4 (Fragment) |  | 1.765 | 3.11E-02 |
| A0A5C2G7C4 | IGH c228_heavy__IGHV3-48_IGHD5-24_IGHJ6 (Fragment) |  | 1.764 | 2.48E-02 |
| A0A5C2GF77 | IGH + IGL c539_heavy_IGHV5-51_IGHD4-17_IGHJ3 (Fragment) |  | 1.760 | 2.33E-02 |
| A0A5C2GJ43 | IG c1253_heavy_IGHV3-23_IGHD3-22_IGHJ4 (Fragment) |  | 1.759 | 1.20E-03 |
| A0A5C2G298 | IGL c3272_light_IGKV3-15_IGKJ2 (Fragment) |  | 1.757 | 2.77E-02 |
| A0A5C2FYC1 | IGL c1166_light_IGKV3-20_IGKJ2 (Fragment) |  | 1.749 | 2.10E-05 |
| A0A5C2GE97 | IGH c331_heavy__IGHV3-74_IGHD3-9_IGHJ4 (Fragment) |  | 1.747 | 4.61E-03 |
| Q68DS3 | Uncharacterized protein DKFZp686H17246 (Fragment) | DKFZp686H17246 | 1.745 | 4.23E-04 |
| A0A5C2GI59 | IGH + IGL c435_heavy_IGHV1-2_IGHD5-12_IGHJ4 (Fragment) |  | 1.741 | 3.90E-03 |
| A0A2U8J8K8 | Ig heavy chain variable region (Fragment) | IgH | 1.738 | 2.87E-03 |
| A0A5C2GXL1 | IG c378_light_IGKV1-9_IGKJ4 (Fragment) |  | 1.727 | 4.23E-03 |
| A0A5C2GGC0 | IG c135_heavy_IGHV4-61_IGHD6-6_IGHJ1 (Fragment) |  | 1.721 | 4.93E-03 |
| A0A5C2FX45 | IGL c1539_light_IGKV3-15_IGKJ1 (Fragment) |  | 1.710 | 3.51E-02 |
| A0A087WWT3 | Serum albumin | ALB | 1.703 | 7.15E-04 |
| A0A1B0GU03 | "Peptidase A1 domain-containing protein |  | 1.701 | 5.76E-03 |
| A0A5C2GFV9 | IG c19_heavy_IGHV1-2_IGHD5-12_IGHJ5 (Fragment) |  | 1.697 | 7.38E-03 |
| A0A5C2G006 | IGL c2512_light_IGKV2D-29_IGKJ2 (Fragment) |  | 1.696 | 1.68E-03 |
| A0A2U8J923 | Ig heavy chain variable region (Fragment) | IgH | 1.679 | 5.17E-03 |
| A0A5C2GDV4 | IG c255_heavy_IGHV3-15_IGHD3-10_IGHJ4 (Fragment) |  | 1.678 | 2.20E-02 |
| A0A140VJI7 | Testicular tissue protein Li 61 |  | 1.677 | 1.14E-06 |
| A0A5C2G9Q1 | IGH c116_heavy__IGHV4-59_IGHD1-14_IGHJ5 (Fragment) |  | 1.675 | 1.67E-03 |
| A0A5C2GEB7 | IGH c361_heavy__IGHV4-39_IGHD5-24_IGHJ4 (Fragment) |  | 1.672 | 1.08E-03 |
| A0A0C4DGB6 | Serum albumin | ALB | 1.664 | 1.55E-06 |
| Q9UHG3 | Prenylcysteine oxidase 1 | PCYOX1 | 1.645 | 9.60E-07 |
| A0A5C2G984 | IGH + IGL c324_heavy_IGHV3-15_IGHD3-16_IGHJ5 (Fragment) |  | 1.642 | 4.68E-02 |
| B3KWB5 | "cDNA FLJ42722 fis, clone BRAMY4000277, highly similar to Alpha-1B-glycoprotein" |  | 1.641 | 4.55E-03 |
| A0A5C2G6G1 | IGL c3946_light_IGLV3-10_IGLJ3 (Fragment) |  | 1.639 | 1.56E-03 |
| B7Z539 | "cDNA FLJ56954, highly similar to Inter-alpha-trypsin inhibitor heavy chain H1" |  | 1.635 | 5.11E-03 |
| A0A5C2GJR4 | IG c548_heavy_IGHV3-53_IGHD5-12_IGHJ4 (Fragment) |  | 1.626 | 1.19E-02 |
| H0YJW9 | Uncharacterized protein (Fragment) |  | 1.618 | 3.58E-04 |
| A0A5C2GNC7 | IG c678_heavy_IGHV3-23_IGHD1-14_IGHJ4 (Fragment) |  | 1.617 | 1.59E-02 |
| A0A5C2GSJ8 | IG c1664_heavy_IGHV5-51_IGHD5-5_IGHJ6 (Fragment) |  | 1.615 | 1.21E-02 |
| A0A2U8J8K4 | Ig heavy chain variable region (Fragment) | IgH | 1.612 | 1.62E-02 |
| A0A5C2GGH1 | IG c1213_heavy_IGHV3-11_IGHD3-22_IGHJ6 (Fragment) |  | 1.608 | 3.71E-02 |
| A2J1N7 | Rheumatoid factor RF-ET10 (Fragment) |  | 1.605 | 9.17E-04 |
| A0A5C2G0F0 | IGL c2642_light_IGKV1D-33_IGKJ3 (Fragment) |  | 1.595 | 1.06E-02 |
| A8K335 | Folate gamma-glutamyl hydrolase |  | 1.592 | 6.57E-03 |
| A0A5C2FZB6 | IGL c1506_light_IGKV1-5_IGKJ1 (Fragment) |  | 1.591 | 1.60E-02 |
| A0A5C2G7Y9 | IGH c508_heavy__IGHV3-33_IGHD1-7_IGHJ4 (Fragment) |  | 1.590 | 1.79E-02 |
| A0A5C2GGE2 | IG c1016_heavy_IGHV3-30_IGHD3-3_IGHJ6 (Fragment) |  | 1.589 | 4.49E-03 |
| A0A5C2GMQ6 | IG c840_light_IGKV3-11_IGKJ4 (Fragment) |  | 1.586 | 1.34E-02 |
| A0A5C2G5C2 | IGH c189_heavy__IGHV3-33_IGHD6-19_IGHJ5 (Fragment) |  | 1.585 | 4.27E-02 |
| A0A5C2GMI8 | IG c63_heavy_IGHV4-39_IGHD7-27_IGHJ4 (Fragment) |  | 1.585 | 1.46E-03 |
| A0A5C2GU90 | IG c67_heavy_IGHV3-74_IGHD1-14_IGHJ3 (Fragment) |  | 1.584 | 1.25E-03 |
| B4DR57 | "cDNA FLJ60818, highly similar to Complement C3" |  | 1.583 | 7.76E-04 |
| A0A2U8J970 | Ig heavy chain variable region (Fragment) | IgH | 1.578 | 4.72E-02 |
| Q6MZQ6 | Uncharacterized protein DKFZp686G11190 | DKFZp686G11190 | 1.575 | 1.50E-03 |
| Q8N355 | IGL@ protein | IGL@ | 1.572 | 3.60E-03 |
| A0A5C2FY53 | IGL c197_light_IGKV1-12_IGKJ2 (Fragment) |  | 1.571 | 5.38E-03 |
| A2J1M5 | Rheumatoid factor RF-IP4 (Fragment) |  | 1.570 | 2.09E-05 |
| A0A5C2GQ05 | IG c1476_heavy_IGHV3-7_IGHD4-11_IGHJ4 (Fragment) |  | 1.563 | 6.11E-04 |
| A0A5C2GYK2 | IG c1707_heavy_IGHV3-15_IGHD1-7_IGHJ5 (Fragment) |  | 1.559 | 3.08E-02 |
| P01833 | Polymeric immunoglobulin receptor | PIGR | 1.557 | 1.95E-03 |
| A0A5C2G3Q6 | IGL c3769_light_IGKV1-16_IGKJ1 (Fragment) |  | 1.557 | 1.01E-02 |
| A0A5C2G615 | IGH c399_heavy__IGHV3-21_IGHD3-9_IGHJ5 (Fragment) |  | 1.557 | 1.25E-02 |
| A0A5C2FWP4 | IGL c1369_light_IGKV1-5_IGKJ2 (Fragment) |  | 1.557 | 1.48E-02 |
| A0A5C2GLC2 | IG c459_light_IGKV1-9_IGKJ2 (Fragment) |  | 1.555 | 2.02E-02 |
| A0A5C2GX83 | IG c1039_light_IGKV4-1_IGKJ2 (Fragment) |  | 1.552 | 2.95E-02 |
| P00748 | Coagulation factor XII | F12 | 1.551 | 1.11E-04 |
| Q5EFE5 | Anti-RhD monoclonal T125 gamma1 heavy chain |  | 1.541 | 1.36E-02 |
| A0A5C2GFW9 | IG c998_heavy_IGHV4-39_IGHD3-3_IGHJ4 (Fragment) |  | 1.537 | 1.11E-02 |
| A0A5C2GET5 | IG c120_heavy_IGHV3-49_IGHD2-2_IGHJ3 (Fragment) |  | 1.537 | 3.97E-02 |
| A0A5C2GF46 | IG c1011_heavy_IGHV3-7_IGHD3-3_IGHJ1 (Fragment) |  | 1.535 | 4.22E-02 |
| A0A5C2GJF3 | IG c1186_heavy_IGHV3-20_IGHD6-13_IGHJ6 (Fragment) |  | 1.533 | 3.24E-03 |
| A0A384MEF1 | Epididymis secretory sperm binding protein |  | 1.533 | 2.77E-09 |
| B4E2Z3 | "cDNA FLJ54090, highly similar to 4F2 cell-surface antigen heavy chain |  | 1.532 | 7.30E-03 |
| A0A024RBS0 | "RNA helicase | DDX55 | 1.525 | 2.66E-03 |
| A0A5C2GCU8 | IGH + IGL c568_heavy_IGHV3-23_IGHD2-21_IGHJ3 (Fragment) |  | 1.525 | 8.19E-03 |
| Q8N6N3 | UPF0690 protein C1orf52 | C1orf52 | 1.519 | 4.66E-02 |
| P06727 | Apolipoprotein A-IV | APOA4 | 1.518 | 8.13E-06 |
| A0A5C2GVT7 | IG c1611_heavy_IGHV3-33_IGHD3-22_IGHJ4 (Fragment) |  | 1.516 | 1.36E-03 |
| A0A5C2GUD6 | IG c1134_light_IGKV3-15_IGKJ1 (Fragment) |  | 1.516 | 1.19E-03 |
| A0A5C2G1U7 | IGL c3139_light_IGKV1-5_IGKJ4 (Fragment) |  | 1.508 | 2.74E-02 |
| P60709 | "Actin, cytoplasmic 1 | ACTB | 1.507 | 4.90E-02 |
| A8K104 | "cDNA FLJ78726, highly similar to Homo sapiens olfactomedin 1 (OLFM1), transcript variant 1, mRNA |  | 1.507 | 1.19E-03 |
| A0A5C2GTY5 | IG c1339_heavy_IGHV3-9_IGHD6-6_IGHJ6 (Fragment) |  | 1.503 | 2.07E-02 |
| A0A5C2GJG3 | IG c830_light_IGKV3-20_IGKJ3 (Fragment) |  | 1.502 | 1.80E-02 |
| A0A2U8J974 | Ig heavy chain variable region (Fragment) | IgH | 1.501 | 2.80E-03 |
| A0A5C2GG66 | IG c858_heavy_IGHV3-21_IGHD5-18_IGHJ5 (Fragment) |  | 1.499 | 1.50E-02 |
| A0A5C2GTS1 | IG c1269_heavy_IGHV3-21_IGHD2-15_IGHJ3 (Fragment) |  | 1.499 | 1.76E-02 |
| A0A5C2GA70 | IGH c163_heavy__IGHV3-43_IGHD6-25_IGHJ6 (Fragment) |  | 1.496 | 5.20E-03 |
| A0A5C2GD92 | IGH + IGL c434_light_IGKV1-5_IGKJ1 (Fragment) |  | 1.495 | 2.97E-03 |
| A0A5C2G8Z6 | IGL c4177_light_IGKV1D-13_IGKJ4 (Fragment) |  | 1.493 | 1.25E-02 |
| A0A5C2GG43 | IG c988_heavy_IGHV3-23_IGHD4-17_IGHJ4 (Fragment) |  | 1.488 | 4.90E-02 |
| C9JF17 | Apolipoprotein D (Fragment) | APOD | 1.488 | 4.71E-05 |
| A0A5C2GE34 | IGH + IGL c380_light_IGKV3-11_IGKJ2 (Fragment) |  | 1.487 | 4.43E-02 |
| A0A248RGE3 | "40S ribosomal protein (Fragment) |  | 1.485 | 2.40E-03 |
| A0A5C2GNK1 | IG c975_heavy_IGHV3-30_IGHD5-24_IGHJ4 (Fragment) |  | 1.483 | 1.32E-02 |
| A8K6C9 | "cDNA FLJ78037, highly similar to Homo sapiens insulin-like growth factor 2 (somatomedin A), mRNA |  | 1.479 | 1.48E-05 |
| A0A5C2GEN9 | IG c579_heavy_IGHV3-33_IGHD1-26_IGHJ3 (Fragment) |  | 1.471 | 1.56E-02 |
| A0A5C2GE66 | IG c431_heavy_IGHV3-23_IGHD4-17_IGHJ5 (Fragment) |  | 1.468 | 1.74E-02 |
| E9PHK0 | Tetranectin | CLEC3B | 1.468 | 7.63E-06 |
| A0A5C2GQ76 | IG c99_heavy_IGHV4-59_IGHD3-10_IGHJ3 (Fragment) |  | 1.468 | 2.51E-03 |
| A0PJA6 | TF protein (Fragment) | TF | 1.467 | 2.04E-02 |
| A0A5C2FUQ0 | IGL c748_light_IGKV4-1_IGKJ1 (Fragment) |  | 1.467 | 4.80E-02 |
| A0A5C2G290 | IGL c2065_light_IGKV3-15_IGKJ1 (Fragment) |  | 1.464 | 1.98E-02 |
| A0A5C2FYU6 | IGL c825_light_IGKV4-1_IGKJ4 (Fragment) |  | 1.463 | 4.70E-02 |
| A0A5C2GU19 | IG c1014_light_IGKV3-15_IGKJ1 (Fragment) |  | 1.460 | 1.02E-02 |
| P02768 | Albumin | ALB | 1.458 | 2.31E-04 |
| A0A5C2H0S9 | IG c536_light_IGKV3-15_IGKJ2 (Fragment) |  | 1.456 | 8.02E-03 |
| A0A087X0M8 | "Neural cell adhesion molecule L1-like protein | CHL1 | 1.454 | 9.93E-03 |
| A0A5C2FX14 | IGL c680_light_IGKV1-39_IGKJ2 (Fragment) |  | 1.452 | 2.13E-02 |
| A0A5C2GVC7 | IG c1525_light_IGLV3-21_IGLJ2 (Fragment) |  | 1.446 | 4.19E-02 |
| A2NYU8 | Heavy chain Fab (Fragment) |  | 1.446 | 3.70E-03 |
| A0A5C2GDN3 | IG c481_heavy_IGHV3-11_IGHD3-22_IGHJ4 (Fragment) |  | 1.443 | 4.20E-03 |
| A0A5C2FY84 | IGL c1604_light_IGKV1-39_IGKJ1 (Fragment) |  | 1.441 | 2.93E-02 |
| Q9UGM5 | Fetuin-B | FETUB | 1.441 | 4.85E-04 |
| A6XND0 | Insulin-like growth factor-binding protein 3 | IGFBP3 | 1.436 | 1.78E-04 |
| A8K6K4 | "cDNA FLJ77565, highly similar to Homo sapiens interleukin 1 receptor accessory protein (IL1RAP), transcript variant 1, mRNA |  | 1.436 | 3.78E-02 |
| A0A5C2GE10 | IGH + IGL c277_heavy_IGHV3-48_IGHD2-15_IGHJ6 (Fragment) |  | 1.436 | 1.91E-03 |
| A0A5C2GLA6 | IG c474_heavy_IGHV4-39_IGHD6-6_IGHJ4 (Fragment) |  | 1.434 | 9.15E-04 |
| A0A140VK24 | Testicular secretory protein Li 24 |  | 1.427 | 3.86E-05 |
| B4DPQ3 | "cDNA FLJ51034, highly similar to Vitamin K-dependent protein C |  | 1.426 | 4.75E-02 |
| Q6UXB8 | Peptidase inhibitor 16 | PI16 | 1.424 | 1.01E-03 |
| A0A5C2GRT8 | IG c1613_heavy_IGHV4-34_IGHD1-7_IGHJ3 (Fragment) |  | 1.424 | 1.35E-02 |
| A0A5C2GD84 | IGH + IGL c543_light_IGKV3-15_IGKJ2 (Fragment) |  | 1.422 | 2.35E-02 |
| A8K3I0 | "cDNA FLJ78437, highly similar to Homo sapiens cartilage oligomeric matrix protein (COMP), mRNA |  | 1.422 | 4.02E-02 |
| A0A5C2GGM0 | IG c55_heavy_IGHV3-48_IGHD3-10_IGHJ3 (Fragment) |  | 1.421 | 1.82E-02 |
| A0A5C2GST2 | IG c859_heavy_IGHV3-13_IGHD3-3_IGHJ3 (Fragment) |  | 1.421 | 4.57E-02 |
| P80108 | Phosphatidylinositol-glycan-specific phospholipase D | GPLD1 | 1.420 | 2.02E-04 |
| A0A5C2GV41 | IG c397_heavy_IGHV3-11_IGHD2-15_IGHJ6 (Fragment) |  | 1.414 | 1.29E-02 |
| A0A5C2FXG2 | IGL c285_light_IGKV3-15_IGKJ3 (Fragment) |  | 1.412 | 2.22E-02 |
| A8K5T0 | "cDNA FLJ75416, highly similar to Homo sapiens complement factor H (CFH), mRNA" |  | 1.412 | 2.36E-02 |
| A0A5C2GGH5 | IG c1305_heavy_IGHV3-23_IGHD3-9_IGHJ4 (Fragment) |  | 1.411 | 1.97E-03 |
| A0A024R6N9 | "Serpin peptidase inhibitor, clade A (Alpha-1 antiproteinase, antitrypsin), member 5, isoform CRA_a | SERPINA5 | 1.411 | 4.94E-06 |
| A0A5C2G5Y6 | IGH c369_heavy__IGHV4-39_IGHD1-7_IGHJ4 (Fragment) |  | 1.410 | 6.23E-04 |
| A0A5C2FXP9 | IGL c990_light_IGLV3-25_IGLJ2 (Fragment) |  | 1.410 | 2.02E-02 |
| A0A5C2GBK7 | IGL c23_light_IGKV3-15_IGKJ4 (Fragment) |  | 1.410 | 2.12E-02 |
| P00488 | Coagulation factor XIII A chain | F13A1 | 1.409 | 2.24E-04 |
| B7WNR0 | Serum albumin | ALB | 1.409 | 4.18E-03 |
| A0A5C2GHR5 | IG c336_heavy_IGHV3-53_IGHD2-8_IGHJ3 (Fragment) |  | 1.404 | 2.23E-03 |
| A0A5C2G0U1 | IGL c1888_light_IGKV3-15_IGKJ1 (Fragment) |  | 1.403 | 4.35E-03 |
| B4DQA0 | L-selectin |  | 1.402 | 3.55E-02 |
| A0A5C2GGD2 | IG c937_heavy_IGHV3-30_IGHD1-26_IGHJ4 (Fragment) |  | 1.402 | 1.30E-02 |
| A0A5C2G9L4 | IGH + IGL c494_heavy_IGHV3-9_IGHD4-11_IGHJ3 (Fragment) |  | 1.400 | 4.04E-04 |
| A0A5C2G7H4 | IGL c388_light_IGKV2-28_IGKJ2 (Fragment) |  | 1.400 | 3.15E-03 |
| A0A5C2GCS5 | IGH + IGL c488_light_IGKV1-8_IGKJ1 (Fragment) |  | 1.399 | 4.92E-02 |
| D6RFY8 | T-complex protein 11 homolog | TCP11 | 1.398 | 4.80E-02 |
| A0A024RAA7 | Adiponectin B | ADIB | 1.397 | 3.98E-04 |
| P11226 | Mannose-binding protein C | MBL2 | 1.390 | 1.20E-02 |
| A0A5C2GN99 | IG c1046_heavy_IGHV3-30_IGHD4-17_IGHJ3 (Fragment) |  | 1.390 | 3.48E-03 |
| A0A5C2GN52 | IG c882_heavy_IGHV3-49_IGHD3-3_IGHJ6 (Fragment) |  | 1.386 | 6.05E-03 |
| A0A5C2G1L7 | IGL c1835_light_IGKV1-5_IGKJ1 (Fragment) |  | 1.384 | 1.95E-02 |
| P02760 | Protein AMBP | AMBP | 1.384 | 8.73E-06 |
| A0A024R6I9 | "Serpin peptidase inhibitor, clade A (Alpha-1 antiproteinase, antitrypsin), member 4, isoform CRA_a | SERPINA4 | 1.384 | 7.20E-05 |
| A0A5C2GHT9 | IG c106_light_IGKV3-15_IGKJ4 (Fragment) |  | 1.383 | 4.04E-03 |
| A0A2Y9CYF7 | Ig heavy chain variable region (Fragment) | IgH | 1.383 | 9.21E-03 |
| A0A087X1J7 | Glutathione peroxidase | GPX3 | 1.381 | 9.33E-04 |
| A0A5C2GDM9 | IGH c71_heavy__IGHV3-7_IGHD4-23_IGHJ3 (Fragment) |  | 1.380 | 3.02E-02 |
| A2RTY6 | "Inter-alpha (Globulin) inhibitor H2 | ITIH2 | 1.380 | 9.46E-04 |
| A0A5C2GGI4 | IG c750_heavy_IGHV5-51_IGHD6-19_IGHJ4 (Fragment) |  | 1.378 | 4.35E-03 |
| A0A0F7TD49 | IGHV3-7 protein (Fragment) | IGHV3-7 | 1.378 | 3.21E-02 |
| P00915 | "Carbonic anhydrase 1 | CA1 | 1.377 | 1.26E-02 |
| Q14520 | Hyaluronan-binding protein 2 | HABP2 | 1.376 | 6.16E-06 |
| A0A5C2GLX0 | IG c157_heavy_IGHV1-2_IGHD7-27_IGHJ4 (Fragment) |  | 1.374 | 1.96E-02 |
| A0A5C2GDJ9 | IGH c51_heavy__IGHV3-43_IGHD3-3_IGHJ4 (Fragment) |  | 1.373 | 3.38E-03 |
| A0A5C2GN18 | IG c225_light_IGKV2D-29_IGKJ4 (Fragment) |  | 1.373 | 1.72E-02 |
| A0A5C2G9H2 | IGH + IGL c353_heavy_IGHV3-9_IGHD6-13_IGHJ4 (Fragment) |  | 1.371 | 3.05E-02 |
| A0A5C2GN44 | IG c54_heavy_IGHV3-7_IGHD3-9_IGHJ5 (Fragment) |  | 1.365 | 1.06E-02 |
| A0A5C2GKG7 | IGH + IGL c527_light_IGKV1D-39_IGKJ1 (Fragment) |  | 1.365 | 6.36E-03 |
| P33151 | "Cadherin-5 | CDH5 | 1.363 | 6.76E-05 |
| A0A5C2GGS8 | IG c894_heavy_IGHV3-30_IGHD3-3_IGHJ6 (Fragment) |  | 1.360 | 6.86E-03 |
| A0A068LN03 | Ig heavy chain variable region (Fragment) |  | 1.356 | 6.23E-05 |
| A0A068LKQ2 | Ig heavy chain variable region (Fragment) |  | 1.354 | 1.64E-02 |
| A0A5C2GE69 | IG c229_heavy_IGHV3-23_IGHD3-3_IGHJ4 (Fragment) |  | 1.354 | 2.42E-02 |
| A0A5C2GU81 | IG c867_light_IGKV3-15_IGKJ2 (Fragment) |  | 1.353 | 2.82E-02 |
| P27169 | Serum paraoxonase/arylesterase 1 | PON1 | 1.353 | 1.89E-04 |
| A8K9V7 | Neuropilin |  | 1.346 | 7.76E-03 |
| A0A5C2GQV0 | IG c269_heavy_IGHV3-13_IGHD4-23_IGHJ6 (Fragment) |  | 1.345 | 2.91E-02 |
| P00742 | Coagulation factor X | F10 | 1.343 | 3.25E-03 |
| A8K1K1 | "cDNA FLJ76342, highly similar to Homo sapiens carnosine dipeptidase 1 (metallopeptidase M20 family)(CNDP1), mRNA |  | 1.343 | 1.44E-02 |
| A0A2U8J9A8 | Ig heavy chain variable region (Fragment) | IgH | 1.342 | 1.97E-02 |
| A0A0S2Z3Y1 | Lectin galactoside-binding soluble 3 binding protein isoform 1 (Fragment) | LGALS3BP | 1.341 | 1.58E-03 |
| A0A1S5UZ39 | Hemoglobin subunit alpha | HBA2 | 1.341 | 6.65E-03 |
| A0A5C2GI34 | IG c798_light_IGKV1-5_IGKJ2 (Fragment) |  | 1.340 | 3.63E-02 |
| A0A140T9C0 | Tenascin-X | TNXB | 1.338 | 2.72E-02 |
| Q68DR3 | Uncharacterized protein DKFZp779H1622 (Fragment) | DKFZp779H1622 | 1.338 | 2.02E-03 |
| A0A5C2G6E9 | IGH c489_heavy__IGHV3-33_IGHD3-16_IGHJ6 (Fragment) |  | 1.336 | 8.34E-03 |
| P78369 | Claudin-10 | CLDN10 | 1.335 | 5.50E-04 |
| O75882 | Attractin | ATRN | 1.334 | 8.10E-07 |
| A0A5C2GRJ2 | IG c1334_heavy_IGHV3-73_IGHD6-6_IGHJ4 (Fragment) |  | 1.331 | 3.29E-02 |
| A0A5C2GGL5 | IG c92_heavy_IGHV4-34_IGHD4-17_IGHJ6 (Fragment) |  | 1.330 | 4.62E-02 |
| A0A5C2FYM3 | IGL c2071_light_IGKV4-1_IGKJ1 (Fragment) |  | 1.330 | 2.83E-02 |
| A0A5C2G462 | IGL c4088_light_IGKV1-39_IGKJ4 (Fragment) |  | 1.329 | 3.55E-02 |
| A0A0S2Z4F1 | "EGF containing fibulin-like extracellular matrix protein 1 isoform 1 (Fragment) | EFEMP1 | 1.327 | 1.43E-04 |
| A0A5C2GUD9 | IG c1125_light_IGKV1D-17_IGKJ1 (Fragment) |  | 1.326 | 3.88E-03 |
| P11021 | Endoplasmic reticulum chaperone BiP | HSPA5 | 1.318 | 1.96E-02 |
| A0A140VKF3 | Testis tissue sperm-binding protein Li 70n |  | 1.317 | 1.64E-04 |
| A0A5C2GQ04 | IG c9_heavy_IGHV4-59B_IGHD3-16_IGHJ4 (Fragment) |  | 1.315 | 4.16E-02 |
| Q53YY1 | "Angiotensinogen (Serine (Or cysteine) proteinase inhibitor, clade A (Alpha-1 antiproteinase, antitrypsin), member 8) | AGT | 1.314 | 1.07E-02 |
| P05160 | Coagulation factor XIII B chain | F13B | 1.313 | 3.97E-06 |
| A0A5C2G7Z6 | IGH c518_heavy__IGHV1-69_IGHD3-22_IGHJ6 (Fragment) |  | 1.312 | 1.47E-02 |
| B0AZL7 | "cDNA, FLJ79457, highly similar to Insulin-like growth factor-binding proteincomplex acid labile chain |  | 1.311 | 3.05E-03 |
| A0A5C2G712 | IGH c98_heavy__IGHV1-18_IGHD3-22_IGHJ4 (Fragment) |  | 1.310 | 1.09E-03 |
| A5PKX5 | Alpha-mannosidase | MAN2A1 | 1.309 | 6.65E-03 |
| P00747 | Plasminogen | PLG | 1.309 | 3.10E-05 |
| A0A5C2GQ75 | IG c680_heavy_IGHV4-34_IGHD2-15_IGHJ4 (Fragment) |  | 1.309 | 2.19E-02 |
| A0A5C2GE26 | IG c1327_heavy_IGHV3-21_IGHD3-3_IGHJ6 (Fragment) |  | 1.308 | 2.94E-03 |
| A0A5C2G7G6 | IGL c3591_light_IGKV1D-39_IGKJ2 (Fragment) |  | 1.305 | 4.24E-02 |
| A0A5C2GXI4 | IG c1317_heavy_IGHV3-64_IGHD2-21_IGHJ4 (Fragment) |  | 1.305 | 1.54E-02 |
| D9YZU5 | Beta-globin | HBB | 1.304 | 4.57E-03 |
| A0A5C2G5M0 | IGL c2937_light_IGKV1-16_IGKJ5 (Fragment) |  | 1.301 | 5.53E-03 |
| A0A5C2GC19 | IGL c241_light_IGKV1-16_IGKJ5 (Fragment) |  | 1.300 | 3.08E-02 |
| A0A384NKS6 | Clusterin |  | 1.299 | 9.37E-04 |
| A0A0S2Z3D5 | Apolipoprotein E isoform 1 (Fragment) | APOE | 1.299 | 4.82E-02 |
| Q03591 | Complement factor H-related protein 1 | CFHR1 | 1.296 | 5.63E-04 |
| A0A5C2G4F1 | IGL c4181_light_IGLV3-25_IGLJ2 (Fragment) |  | 1.295 | 3.71E-02 |
| A0A024RAG6 | Adiponectin C | ADIC | 1.294 | 1.69E-04 |
| A0A2Z4LCH4 | "Corticosteroid-binding globulin |  | 1.293 | 5.70E-04 |
| A0A5C2GUB3 | IG c532_light_IGKV3-11_IGKJ3 (Fragment) |  | 1.290 | 3.09E-02 |
| A0A5C2G172 | IGL c73_light_IGKV2D-28_IGKJ5 (Fragment) |  | 1.289 | 2.72E-02 |
| P48740 | Mannan-binding lectin serine protease 1 | MASP1 | 1.289 | 7.07E-03 |
| A3KPE2 | Apolipoprotein C-III | APOC3 | 1.286 | 3.67E-02 |
| A0A024RAB9 | Adiponectin A | ADIA | 1.281 | 1.25E-04 |
| A0A5C2GRE3 | IG c54_light_IGKV2-28_IGKJ2 (Fragment) |  | 1.280 | 2.03E-02 |
| A0A5C2G3L6 | IGL c3878_light_IGKV1-5_IGKJ1 (Fragment) |  | 1.275 | 1.69E-02 |
| A0A5C2GDV3 | IGH + IGL c197_heavy_IGHV1-69_IGHD3-10_IGHJ4 (Fragment) |  | 1.275 | 3.63E-02 |
| A0A5C2GA74 | IGH + IGL c21_heavy_IGHV4-59_IGHD3-3_IGHJ3 (Fragment) |  | 1.273 | 1.87E-02 |
| A8KAP5 | "cDNA FLJ76426, highly similar to Homo sapiens intercellular adhesion molecule 2 (ICAM2), mRNA |  | 1.268 | 1.08E-02 |
| P43251 | Biotinidase | BTD | 1.264 | 9.49E-05 |
| A0A5C2FTS3 | IGL c102_light_IGKV3-15_IGKJ2 (Fragment) |  | 1.259 | 4.23E-02 |
| A0A5C2GZ77 | IG c1090_light_IGKV1-12_IGKJ3 (Fragment) |  | 1.259 | 1.93E-02 |
| A0A5C2FZR2 | IGL c2194_light_IGLV3-19_IGLJ3 (Fragment) |  | 1.258 | 4.07E-02 |
| A0A5C2GWJ7 | IG c1303_light_IGKV1-9_IGKJ2 (Fragment) |  | 1.258 | 3.00E-02 |
| A0A0S2Z3F6 | Cholesteryl ester transfer protein plasma isoform 1 (Fragment) | CETP | 1.257 | 1.86E-02 |
| A0A5C2G340 | IGL c2375_light_IGLV3-25_IGLJ3 (Fragment) |  | 1.257 | 2.61E-02 |
| A0A5C2GL86 | IG c86_heavy_IGHV5-51_IGHD3-16_IGHJ6 (Fragment) |  | 1.255 | 2.95E-02 |
| A0A5C2G1J4 | IGL c2326_light_IGKV1-39_IGKJ1 (Fragment) |  | 1.255 | 3.01E-02 |
| Q04756 | Hepatocyte growth factor activator | HGFAC | 1.240 | 1.09E-02 |
| A0A5C2GKA2 | IG c756_light_IGKV1-12_IGKJ3 (Fragment) |  | 1.237 | 2.53E-02 |
| A0A5C2GE27 | IG c1244_heavy_IGHV3-21_IGHD3-22_IGHJ4 (Fragment) |  | 1.236 | 1.90E-02 |
| P19827 | Inter-alpha-trypsin inhibitor heavy chain H1 | ITIH1 | 1.235 | 1.65E-03 |
| A0A5C2GFE5 | IG c1015_heavy_IGHV3-7_IGHD6-13_IGHJ4 (Fragment) |  | 1.234 | 4.35E-02 |
| A0A5C2GBG1 | IGL c51_light_IGLV3-19_IGLJ2 (Fragment) |  | 1.232 | 2.20E-02 |
| E9KL23 | Epididymis secretory sperm binding protein Li 44a | SERPINA1 | 1.230 | 8.71E-03 |
| V9HWI6 | Epididymis secretory protein Li 51 | HEL-S-51 | 1.230 | 1.34E-06 |
| A0A024R8G3 | "Prostaglandin D2 synthase 21kDa (Brain), isoform CRA_a | PTGDS | 1.229 | 1.42E-02 |
| P22792 | Carboxypeptidase N subunit 2 | CPN2 | 1.223 | 8.23E-03 |
| A0A5C2G5Z2 | IGH c376_heavy__IGHV3-11_IGHD6-19_IGHJ5 (Fragment) |  | 1.221 | 4.18E-02 |
| A0A024R944 | "Serpin peptidase inhibitor, clade C (Antithrombin), member 1, isoform CRA_a | SERPINC1 | 1.217 | 1.03E-02 |
| P02753 | Retinol-binding protein 4 | RBP4 | 1.208 | 3.19E-02 |
| D9ZGG2 | Vitronectin | VTN | 1.204 | 4.20E-03 |
| A0A5C2FY30 | IGL c1554_light_IGKV1-27_IGKJ3 (Fragment) |  | 1.204 | 2.74E-02 |
| A0A5C2GU98 | IG c77_heavy_IGHV4-39_IGHD6-13_IGHJ4 (Fragment) |  | 1.201 | 3.34E-02 |
| A0A5C2GFV2 | IGL c279_light_IGKV3-20_IGKJ2 (Fragment) |  | 0.832 | 2.57E-02 |
| A0A5C2GTQ5 | IG c874_light_IGKV3-20_IGKJ5 (Fragment) |  | 0.828 | 3.67E-02 |
| A0A5C2GT13 | IG c655_light_IGKV1D-13_IGKJ5 (Fragment) |  | 0.818 | 1.78E-02 |
| A0A024RDT4 | "Lymphocyte cytosolic protein 1 (L-plastin), isoform CRA_a | LCP1 | 0.818 | 1.58E-02 |
| A0A5C2FW31 | IGL c854_light_IGKV1-5_IGKJ1 (Fragment) |  | 0.815 | 4.57E-03 |
| A2KBC4 | Anti-TN-C scFv (Fragment) |  | 0.808 | 7.98E-03 |
| A0A5C2G219 | IGL c1862_light_IGKV1-5_IGKJ2 (Fragment) |  | 0.807 | 2.06E-02 |
| A0A5C2H3T0 | IG c1706_light_IGKV3-11_IGKJ2 (Fragment) |  | 0.804 | 1.30E-02 |
| G3GAU4 | Anti-H1N1 influenza HA kappa chain variable region (Fragment) |  | 0.800 | 9.58E-03 |
| P02763 | Alpha-1-acid glycoprotein 1 | ORM1 | 0.800 | 2.79E-02 |
| A2NV55 | Precursor (AA -19 to 113) (Fragment) |  | 0.799 | 4.72E-02 |
| A0A5C2G7P4 | IGL c377_light_IGKV3D-20_IGKJ1 (Fragment) |  | 0.799 | 2.33E-02 |
| P03950 | Angiogenin | ANG | 0.795 | 4.44E-02 |
| A0A5C2G4F7 | IGL c3450_light_IGKV1-39_IGKJ1 (Fragment) |  | 0.795 | 1.65E-02 |
| A0A5C2G7H3 | IGL c3697_light_IGLV6-57_IGLJ2 (Fragment) |  | 0.789 | 8.63E-03 |
| A0A5C2FYD0 | IGL c1899_light_IGKV3-20_IGKJ4 (Fragment) |  | 0.786 | 3.30E-02 |
| A0A5C2GU54 | IG c1409_heavy_IGHV4-39_IGHD3-3_IGHJ4 (Fragment) |  | 0.782 | 3.29E-02 |
| A0A5C2G807 | IGL c3887_light_IGKV3-20_IGKJ3 (Fragment) |  | 0.782 | 2.94E-02 |
| A0A5C2GMF2 | IG c998_light_IGKV4-1_IGKJ2 (Fragment) |  | 0.782 | 3.32E-02 |
| A0A5C2GJR5 | IG c837_light_IGKV1-39_IGKJ2 (Fragment) |  | 0.779 | 1.26E-02 |
| A2J1M3 | Rheumatoid factor RF-ET5 (Fragment) |  | 0.777 | 7.47E-03 |
| A0A5C2GK68 | IG c744_light_IGLV1-51_IGLJ2 (Fragment) |  | 0.777 | 3.31E-02 |
| A0A5C2GVU7 | IG c1621_heavy_IGHV3-49_IGHD2-2_IGHJ5 (Fragment) |  | 0.776 | 2.90E-02 |
| A0A5C2FXJ0 | IGL c325_light_IGKV2-24_IGKJ4 (Fragment) |  | 0.773 | 4.09E-03 |
| A0A5C2G3I9 | IGL c3848_light_IGLV6-57_IGLJ3 (Fragment) |  | 0.769 | 2.15E-02 |
| A0A5C2GQX1 | IG c289_heavy_IGHV4-34_IGHD3-3_IGHJ4 (Fragment) |  | 0.766 | 1.06E-02 |
| A0A2U8J969 | Ig heavy chain variable region (Fragment) | IgH | 0.764 | 7.87E-03 |
| A0A5C2GXP9 | IG c1387_heavy_IGHV4-39_IGHD6-13_IGHJ2 (Fragment) |  | 0.762 | 2.42E-02 |
| A0A024R035 | "Complement component 9, isoform CRA_a | C9 | 0.759 | 4.36E-03 |
| A0A5C2GIX9 | IG c1267_light_IGLV2-14_IGLJ1 (Fragment) |  | 0.758 | 4.86E-02 |
| A0A5C2GR61 | IG c1832_heavy_IGHV5-51_IGHD2-21_IGHJ4 (Fragment) |  | 0.757 | 4.46E-02 |
| A0A5C2GUG4 | IG c1155_light_IGKV2-30_IGKJ4 (Fragment) |  | 0.755 | 9.52E-03 |
| A0A5C2GGH2 | IGL c519_light_IGKV1D-39_IGKJ4 (Fragment) |  | 0.754 | 3.83E-03 |
| A0A5C2GHF8 | IGH + IGL c621_light_IGKV4-1_IGKJ1 (Fragment) |  | 0.753 | 3.88E-02 |
| A0A5C2GLK7 | IG c58_heavy_IGHV3-23_IGHD5-18_IGHJ4 (Fragment) |  | 0.753 | 3.57E-02 |
| A0A5C2G2U0 | IGL c3588_light_IGKV3-11_IGKJ4 (Fragment) |  | 0.751 | 3.17E-02 |
| A0A5C2GKK0 | IGH + IGL c567_light_IGLV1-40_IGLJ3 (Fragment) |  | 0.751 | 1.67E-02 |
| A0A5C2GED4 | IG c373_heavy_IGHV3-30_IGHD2-8_IGHJ4 (Fragment) |  | 0.751 | 3.20E-02 |
| A0A5C2G1I8 | IGL c2992_light_IGLV3-25_IGLJ2 (Fragment) |  | 0.750 | 3.56E-03 |
| A0A5C2GZU5 | IG c1300_light_IGKV1-12_IGKJ1 (Fragment) |  | 0.749 | 2.09E-02 |
| A0A5C2GM07 | IG c1213_light_IGKV2D-28_IGKJ2 (Fragment) |  | 0.748 | 2.72E-02 |
| P01591 | Immunoglobulin J chain | JCHAIN | 0.747 | 6.70E-03 |
| A0A5C2GKA1 | IGH + IGL c447_light_IGLV3-25_IGLJ3 (Fragment) |  | 0.747 | 2.18E-02 |
| A0A5C2GQT8 | IG c1190_light_IGKV3-20_IGKJ2 (Fragment) |  | 0.744 | 4.40E-03 |
| A0A5C2G8X7 | IGL c286_light_IGKV1-5_IGKJ1 (Fragment) |  | 0.744 | 3.27E-02 |
| A2N2F4 | VK3 protein (Fragment) | VK3 | 0.743 | 4.30E-02 |
| A0A5C2FUX9 | IGL c811_light_IGKV3-20_IGKJ1 (Fragment) |  | 0.743 | 6.39E-04 |
| A0A5C2G6P0 | IGL c2093_light_IGKV1-39_IGKJ3 (Fragment) |  | 0.743 | 2.46E-03 |
| A0A5C2GH52 | IGH + IGL c65_heavy_IGHV5-51_IGHD5-24_IGHJ5 (Fragment) |  | 0.739 | 5.73E-03 |
| A0A5C2GM54 | IG c595_heavy_IGHV5-51_IGHD2-2_IGHJ4 (Fragment) |  | 0.732 | 3.47E-05 |
| A0A5C2GUC4 | IG c542_light_IGKV2D-29_IGKJ3 (Fragment) |  | 0.729 | 3.82E-02 |
| A0A5C2GS95 | IG c531_heavy_IGHV4-34_IGHD6-13_IGHJ4 (Fragment) |  | 0.728 | 1.54E-03 |
| A0A5C2H108 | IG c1743_light_IGLV6-57_IGLJ3 (Fragment) |  | 0.728 | 4.52E-02 |
| A0A5C2G1Z2 | IGL c3291_light_IGKV2D-29_IGKJ2 (Fragment) |  | 0.728 | 4.23E-03 |
| A0A5C2FU15 | IGL c222_light_IGKV3-20_IGKJ1 (Fragment) |  | 0.728 | 7.67E-04 |
| A0A5C2GEW3 | IGH c591_heavy__IGHV1-18_IGHD2-8_IGHJ4 (Fragment) |  | 0.728 | 3.00E-02 |
| A0A5C2GM42 | IG c288_heavy_IGHV1-69_IGHD3-22_IGHJ4 (Fragment) |  | 0.727 | 3.01E-03 |
| A0A5C2G2I2 | IGL c3498_light_IGKV4-1_IGKJ3 (Fragment) |  | 0.727 | 1.19E-02 |
| A0A5C2GCA5 | IGL c213_light_IGKV3D-20_IGKJ4 (Fragment) |  | 0.725 | 1.45E-02 |
| A0A5C2GAW1 | IGL c562_light_IGKV1D-39_IGKJ1 (Fragment) |  | 0.724 | 4.75E-02 |
| A0A5C2FWY2 | IGL c1332_light_IGKV3-11_IGKJ5 (Fragment) |  | 0.722 | 1.19E-02 |
| A0A5C2G2S7 | IGL c2766_light_IGKV1-5_IGKJ2 (Fragment) |  | 0.718 | 3.05E-03 |
| A0A5C2GYE9 | IG c1409_light_IGKV3-20_IGKJ2 (Fragment) |  | 0.714 | 1.25E-03 |
| A0A5C2GQI9 | IG c1656_heavy_IGHV4-61_IGHD1-26_IGHJ5 (Fragment) |  | 0.713 | 6.95E-03 |
| A0A5C2GC00 | IGH + IGL c93_light_IGKV1-5_IGKJ1 (Fragment) |  | 0.713 | 1.25E-02 |
| A0A5C2GPA8 | IG c673_heavy_IGHV3-33_IGHD2-8_IGHJ4 (Fragment) |  | 0.713 | 8.07E-03 |
| A0A5C2GJQ5 | IG c477_light_IGLV1-40_IGLJ3 (Fragment) |  | 0.712 | 8.35E-03 |
| A0A5C2GJ30 | IG c883_heavy_IGHV3-23_IGHD7-27_IGHJ4 (Fragment) |  | 0.712 | 2.32E-02 |
| A0A5C2GLZ3 | IG c1022_heavy_IGHV1-2_IGHD3-16_IGHJ6 (Fragment) |  | 0.711 | 1.18E-03 |
| A0A5C2GRJ6 | IG c31_light_IGLV1-44_IGLJ3 (Fragment) |  | 0.709 | 8.17E-04 |
| A0A5C2G982 | IGL c4205_light_IGKV3-15_IGKJ3 (Fragment) |  | 0.708 | 2.05E-02 |
| A0A5C2GUY6 | IG c583_light_IGKV1-5_IGKJ4 (Fragment) |  | 0.708 | 6.25E-05 |
| A0A5C2G382 | IGL c2405_light_IGKV3-15_IGKJ4 (Fragment) |  | 0.708 | 1.84E-02 |
| A0A5C2GJ67 | IGH + IGL c47_light_IGLV3-19_IGLJ1 (Fragment) |  | 0.708 | 6.27E-04 |
| A0A5C2GHW5 | IG c466_light_IGKV2-30_IGKJ2 (Fragment) |  | 0.707 | 5.17E-03 |
| A0A5C2FXX3 | IGL c1494_light_IGKV3-15_IGKJ5 (Fragment) |  | 0.706 | 9.92E-03 |
| A0A5C2GL90 | IG c96_heavy_IGHV3-48_IGHD4-17_IGHJ6 (Fragment) |  | 0.705 | 3.44E-02 |
| A0A5C2FY76 | IGL c575_light_IGKV1-12_IGKJ4 (Fragment) |  | 0.703 | 1.52E-02 |
| A0A5C2FTC4 | IGL c208_light_IGKV1-5_IGKJ1 (Fragment) |  | 0.701 | 1.34E-02 |
| A0A5C2GJH7 | IGH + IGL c157_light_IGLV1-51_IGLJ3 (Fragment) |  | 0.699 | 2.57E-02 |
| B4E1I8 | "cDNA FLJ54228, highly similar to Leucine-rich alpha-2-glycoprotein |  | 0.698 | 1.59E-03 |
| A0A4P8J559 | IgK_IGKV3-11 (Fragment) |  | 0.698 | 1.38E-02 |
| A0A5C2GKH0 | IG c678_light_IGKV4-1_IGKJ1 (Fragment) |  | 0.695 | 1.15E-03 |
| A0A5C2GCN1 | IGH + IGL c438_light_IGLV1-47_IGLJ3 (Fragment) |  | 0.694 | 1.49E-02 |
| A0A5C2FVE4 | IGL c40_light_IGKV3-20_IGKJ2 (Fragment) |  | 0.690 | 4.62E-04 |
| A0A5C2G127 | IGL c33_light_IGKV2-30_IGKJ1 (Fragment) |  | 0.689 | 4.30E-03 |
| A0A2U8J8Y8 | Ig heavy chain variable region (Fragment) | IgH | 0.687 | 4.50E-02 |
| A0A5C2GHG1 | IG c906_light_IGLV4-69_IGLJ2 (Fragment) |  | 0.687 | 1.43E-02 |
| A0A5C2FXA4 | IGL c1611_light_IGKV3-11_IGKJ5 (Fragment) |  | 0.686 | 9.98E-04 |
| A0A5C2GB27 | IGH + IGL c285_light_IGKV1-5_IGKJ2 (Fragment) |  | 0.684 | 4.60E-06 |
| A0A5C2GLS9 | IG c138_heavy_IGHV5-51_IGHD6-13_IGHJ3 (Fragment) |  | 0.681 | 3.29E-02 |
| A0A5C2G5W9 | IGH c107_heavy__IGHV4-59B_IGHD1-7_IGHJ4 (Fragment) |  | 0.681 | 3.05E-02 |
| A0A5C2FZT5 | IGL c2528_light_IGKV3-20_IGKJ2 (Fragment) |  | 0.680 | 4.46E-03 |
| A0A5C2G047 | IGL c1920_light_IGKV3-11_IGKJ4 (Fragment) |  | 0.679 | 4.53E-03 |
| A0A5C2GUV0 | IG c652_light_IGLV1-40_IGLJ2 (Fragment) |  | 0.679 | 2.66E-03 |
| A0A5C2GQ45 | IG c1516_heavy_IGHV3-9_IGHD3-22_IGHJ3 (Fragment) |  | 0.675 | 2.64E-04 |
| A0A5C2GHK4 | IG c602_heavy_IGHV3-48_IGHD6-13_IGHJ4 (Fragment) |  | 0.675 | 4.21E-03 |
| A0A5C2G6E2 | IGL c3217_light_IGKV1-9_IGKJ4 (Fragment) |  | 0.674 | 3.20E-02 |
| A0A5C2GI31 | IG c1191_heavy_IGHV4-39_IGHD5-12_IGHJ4 (Fragment) |  | 0.671 | 2.03E-04 |
| A0A5C2GE41 | IG c125_heavy_IGHV3-23_IGHD2-8_IGHJ4 (Fragment) |  | 0.671 | 1.76E-03 |
| A0A5C2GT39 | IG c1660_heavy_IGHV4-34_IGHD5-5_IGHJ6 (Fragment) |  | 0.671 | 2.02E-04 |
| A0A5C2G711 | IGL c224_light_IGKV3-11_IGKJ4 (Fragment) |  | 0.671 | 8.02E-03 |
| A0A5C2GJN0 | IG c859_light_IGKV1-39_IGKJ2 (Fragment) |  | 0.671 | 7.64E-03 |
| A0A5C2G2P9 | IGL c2225_light_IGLV1-47_IGLJ2 (Fragment) |  | 0.671 | 7.30E-05 |
| A0A5C2GK52 | IG c101_heavy_IGHV4-34_IGHD3-3_IGHJ6 (Fragment) |  | 0.667 | 2.47E-03 |
| A0A5C2G5X1 | IGL c3067_light_IGKV4-1_IGKJ4 (Fragment) |  | 0.665 | 6.28E-05 |
| A0A1W6IYJ8 | N90-VRC38.05 light chain variable region (Fragment) |  | 0.664 | 6.45E-03 |
| A0A5C2GFC3 | IG c332_heavy_IGHV3-7_IGHD2-15_IGHJ1 (Fragment) |  | 0.659 | 3.37E-03 |
| A0A5C2GKH6 | IG c1226_heavy_IGHV3-53_IGHD5-12_IGHJ4 (Fragment) |  | 0.658 | 4.95E-02 |
| A0A5C2GBW1 | IGL c3833_light_IGLV6-57_IGLJ3 (Fragment) |  | 0.656 | 1.62E-02 |
| A0A5C2GX29 | IG c1503_light_IGKV3-20_IGKJ3 (Fragment) |  | 0.655 | 3.06E-02 |
| A0A5C2FTY9 | IGL c448_light_IGKV3-20_IGKJ2 (Fragment) |  | 0.647 | 9.69E-03 |
| A0A5C2G1C2 | IGL c3061_light_IGKV3-11_IGKJ4 (Fragment) |  | 0.646 | 5.82E-03 |
| A0A5C2GVX4 | IG c1735_light_IGKV3-20_IGKJ2 (Fragment) |  | 0.644 | 8.08E-03 |
| A0A5C2G0D8 | IGL c2639_light_IGKV3-20_IGKJ4 (Fragment) |  | 0.639 | 4.28E-02 |
| A0A5C2GRQ8 | IG c1210_heavy_IGHV3-33_IGHD1-7_IGHJ6 (Fragment) |  | 0.639 | 1.10E-02 |
| A0A5C2GMP7 | IG c645_light_IGKV3-11_IGKJ4 (Fragment) |  | 0.636 | 4.07E-03 |
| A0A5C2G3U1 | IGL c3584_light_IGLV4-69_IGLJ1 (Fragment) |  | 0.630 | 1.15E-03 |
| A0A5C2FZF5 | IGL c2074_light_IGKV6-21_IGKJ1 (Fragment) |  | 0.628 | 9.41E-05 |
| A0A5C2GGS9 | IG c79_heavy_IGHV3-23_IGHD5-24_IGHJ5 (Fragment) |  | 0.628 | 4.57E-03 |
| A0A5C2GKX8 | IG c1161_light_IGKV1-5_IGKJ2 (Fragment) |  | 0.625 | 1.89E-04 |
| A0A5C2GIR3 | IG c455_light_IGLV2-14_IGLJ3 (Fragment) |  | 0.624 | 1.57E-02 |
| A0A5C2G309 | IGL c3532_light_IGLV4-69_IGLJ2 (Fragment) |  | 0.623 | 7.19E-03 |
| P00738 | Haptoglobin | HP | 0.617 | 8.48E-06 |
| A0A5C2GJS3 | IG c1127_light_IGKV1-12_IGKJ4 (Fragment) |  | 0.617 | 2.02E-02 |
| A0A5C2FX48 | IGL c806_light_IGKV1-5_IGKJ2 (Fragment) |  | 0.616 | 1.20E-03 |
| P18428 | Lipopolysaccharide-binding protein | LBP | 0.612 | 1.13E-02 |
| A0A5C2FYA5 | IGL c1146_light_IGKV1D-33_IGKJ2 (Fragment) |  | 0.609 | 2.55E-04 |
| Q65ZC9 | Single-chain Fv (Fragment) | scFv | 0.608 | 3.41E-05 |
| A0A5C2GGJ2 | IG c379_heavy_IGHV4-31_IGHD5-12_IGHJ4 (Fragment) |  | 0.607 | 2.59E-02 |
| A0A5C2GJ84 | IGH + IGL c67_light_IGKV3-20_IGKJ1 (Fragment) |  | 0.606 | 1.34E-03 |
| A0A5C2GMH4 | IG c652_heavy_IGHV4-34_IGHD1-26_IGHJ6 (Fragment) |  | 0.605 | 6.21E-03 |
| A0A5C2G267 | IGL c3259_light_IGKV3-11_IGKJ5 (Fragment) |  | 0.604 | 3.54E-03 |
| A0A5C2GGL7 | IG c468_heavy_IGHV4-61_IGHD6-13_IGHJ6 (Fragment) |  | 0.604 | 7.29E-04 |
| A0A5C2G4N9 | IGL c3874_light_IGKV1D-39_IGKJ2 (Fragment) |  | 0.600 | 1.60E-04 |
| A0A5C2FVH4 | IGL c1031_light_IGLV1-40_IGLJ1 (Fragment) |  | 0.599 | 1.46E-02 |
| A0A5C2GL81 | IG c325_heavy_IGHV5-51_IGHD5-24_IGHJ4 (Fragment) |  | 0.599 | 4.79E-05 |
| A2VDG3 | "CSF1R protein (Fragment) | CSF1R | 0.598 | 7.01E-04 |
| A0A5C2G3L5 | IGL c2027_light_IGLV1-40_IGLJ3 (Fragment) |  | 0.591 | 2.39E-03 |
| A0A5C2G8U7 | IGL c256_light_IGKV3-15_IGKJ1 (Fragment) |  | 0.591 | 1.30E-03 |
| A0A5C2GFK5 | IGH + IGL c21_light_IGKV4-1_IGKJ1 (Fragment) |  | 0.591 | 1.94E-05 |
| A0A5C2GNC4 | IG c742_light_IGKV1-5_IGKJ1 (Fragment) |  | 0.590 | 7.77E-04 |
| A0A5C2GAN2 | IGH + IGL c266_light_IGKV1-27_IGKJ4 (Fragment) |  | 0.585 | 1.11E-04 |
| A0A5C2GAQ3 | IGH + IGL c282_light_IGKV4-1_IGKJ1 (Fragment) |  | 0.584 | 2.44E-02 |
| B6EDE2 | Epididymis luminal protein 180 (Fragment) | HEL180 | 0.584 | 1.40E-03 |
| A0A5C2GYC3 | IG c1742_light_IGKV1-5_IGKJ2 (Fragment) |  | 0.580 | 1.11E-04 |
| A0A5C2G3S0 | IGL c3136_light_IGKV3-11_IGKJ1 (Fragment) |  | 0.575 | 1.49E-04 |
| A0A5C2GT79 | IG c664_light_IGKV2-30_IGKJ3 (Fragment) |  | 0.574 | 1.92E-04 |
| A0A5C2GKG3 | IG c273_light_IGKV1-6_IGKJ1 (Fragment) |  | 0.573 | 7.02E-04 |
| A0A5C2H271 | IG c1046_light_IGKV1-5_IGKJ3 (Fragment) |  | 0.570 | 2.83E-04 |
| A0A5C2FZA5 | IGL c2229_light_IGKV4-1_IGKJ2 (Fragment) |  | 0.569 | 1.02E-06 |
| A0A5C2FZX0 | IGL c2511_light_IGKV3-11_IGKJ1 (Fragment) |  | 0.562 | 4.18E-02 |
| A0A5C2G7C2 | IGH c467_heavy__IGHV5-51_IGHD5-12_IGHJ4 (Fragment) |  | 0.560 | 4.46E-02 |
| A0A5C2GWS2 | IG c1272_light_IGLV2-11_IGLJ3 (Fragment) |  | 0.556 | 4.83E-02 |
| A0A5C2GMV9 | IG c217_heavy_IGHV3-23_IGHD6-25_IGHJ4 (Fragment) |  | 0.556 | 6.15E-06 |
| A0A0A0MS14 | Immunoglobulin heavy variable 1-45 | IGHV1-45 | 0.551 | 6.03E-04 |
| A0A5C2FYB3 | IGL c2018_light_IGKV3-11_IGKJ4 (Fragment) |  | 0.550 | 1.04E-04 |
| A0A5C2G6C0 | IGH c459_heavy__IGHV4-31_IGHD3-9_IGHJ6 (Fragment) |  | 0.549 | 1.22E-02 |
| A0A5C2G175 | IGL c3048_light_IGKV1-39_IGKJ2 (Fragment) |  | 0.546 | 9.65E-03 |
| A0A5C2GA01 | IGH c93_heavy__IGHV1-8_IGHD2-2_IGHJ5 (Fragment) |  | 0.537 | 1.51E-06 |
| A0A5C2GKF6 | IG c35_heavy_IGHV3-7_IGHD5-24_IGHJ4 (Fragment) |  | 0.536 | 3.26E-03 |
| A0A5C2FZ22 | IGL c517_light_IGKV1-39_IGKJ2 (Fragment) |  | 0.535 | 5.87E-06 |
| O75460 | Serine/threonine-protein kinase/endoribonuclease IRE1 | ERN1 | 0.531 | 3.89E-03 |
| A0A5C2GLG0 | IG c8_heavy_IGHV3-33_IGHD2-2_IGHJ4 (Fragment) |  | 0.531 | 2.19E-03 |
| A0A5C2FZE7 | IGL c1045_light_IGKV3-20_IGKJ1 (Fragment) |  | 0.527 | 1.19E-03 |
| A0A5C2GJK3 | IGH + IGL c187_light_IGLV1-44_IGLJ2 (Fragment) |  | 0.526 | 3.63E-02 |
| Q7M4S4 | Granulocyte inhibitory protein |  | 0.520 | 7.68E-06 |
| A0A5C2GTH5 | IG c855_light_IGKV3D-15_IGKJ5 (Fragment) |  | 0.512 | 1.08E-04 |
| A0A5C2GSF4 | IG c321_light_IGKV3-15_IGKJ4 (Fragment) |  | 0.512 | 8.90E-05 |
| A0A5C2G9I6 | IGL c442_light_IGKV1D-39_IGKJ1 (Fragment) |  | 0.507 | 1.18E-06 |
| P0DOX7 | Immunoglobulin kappa light chain |  | 0.494 | 3.72E-07 |
| A0A5C2FZJ6 | IGL c1576_light_IGKV3-11_IGKJ4 (Fragment) |  | 0.486 | 6.98E-05 |
| A0A5C2GCW0 | IGH + IGL c528_light_IGKV2D-28_IGKJ5 (Fragment) |  | 0.481 | 4.13E-02 |
| A0A5C2GMY4 | IG c604_light_IGKV2D-30_IGKJ1 (Fragment) |  | 0.479 | 4.29E-05 |
| A0A5C2G774 | IGL c4186_light_IGLV2-11_IGLJ1 (Fragment) |  | 0.478 | 2.94E-03 |
| A0A5C2FVK1 | IGL c644_light_IGKV3-20_IGKJ4 (Fragment) |  | 0.467 | 9.85E-04 |
| A0A5C2GA04 | IGH + IGL c22_light_IGLV3-25_IGLJ1 (Fragment) |  | 0.463 | 4.21E-02 |
| A0A5C2FZV6 | IGL c1205_light_IGKV3-15_IGKJ2 (Fragment) |  | 0.463 | 2.00E-05 |
| A0A5C2G4U1 | IGL c2537_light_IGKV3-11_IGKJ4 (Fragment) |  | 0.462 | 1.64E-06 |
| A0A5C2FVB0 | IGL c961_light_IGKV1D-39_IGKJ2 (Fragment) |  | 0.459 | 8.31E-07 |
| A0A5C2GUW4 | IG c1311_heavy_IGHV4-39_IGHD6-19_IGHJ4 (Fragment) |  | 0.459 | 1.15E-02 |
| A0A5C2GW38 | IG c1751_heavy_IGHV3-13_IGHD3-3_IGHJ2 (Fragment) |  | 0.457 | 4.10E-06 |
| A0A5C2FZR4 | IGL c1616_light_IGLV2-11_IGLJ1 (Fragment) |  | 0.447 | 4.44E-04 |
| A0A5C2GIK7 | IG c117_light_IGLV4-60_IGLJ2 (Fragment) |  | 0.441 | 1.39E-03 |
| A0A5C2H035 | IG c1430_light_IGKV3-15_IGKJ4 (Fragment) |  | 0.429 | 1.35E-03 |
| A0A5C2GAA6 | IGH + IGL c156_heavy_IGHV1-69_IGHD4-23_IGHJ6 (Fragment) |  | 0.419 | 6.73E-03 |
| A0A5C2FXQ9 | IGL c1719_light_IGKV1-27_IGKJ2 (Fragment) |  | 0.415 | 2.86E-07 |
| A0A5C2GJW1 | IG c409_light_IGKV3D-15_IGKJ4 (Fragment) |  | 0.399 | 4.22E-03 |
| A0A0U4DJF7 | Haptoglobin (Fragment) | HP | 0.391 | 3.35E-02 |
| A0A5C2GB29 | IGH + IGL c302_heavy_IGHV3-23_IGHD3-22_IGHJ5 (Fragment) |  | 0.387 | 8.07E-08 |
| A0A5C2G8D9 | IGL c290_light_IGLV1-47_IGLJ2 (Fragment) |  | 0.363 | 4.35E-02 |
| A0A5C2G9R7 | IGH + IGL c554_heavy_IGHV3-53_IGHD6-13_IGHJ5 (Fragment) |  | 0.314 | 1.13E-03 |
| B2R983 | "cDNA, FLJ94267, highly similar to Homo sapiens glutathione S-transferase omega 1 (GSTO1), mRNA |  | 0.194 | 2.34E-02 |

Table S9: Gene correspondence of differential proteins between the TB0 group and the TB6 group

| Protein | Protein Description | Gene Name |
| --- | --- | --- |
| A0A5H1ZRQ7 | Immunoglobulin lambda constant 7 (Fragment) | IGLC7 |
| A0A024RAB7 | "UDP-galactose-4-epimerase, isoform CRA_b | GALE |
| D6RF35 | Vitamin D-binding protein | GC |
| Q6MZU6 | Uncharacterized protein DKFZp686C15213 | DKFZp686C15213 |
| A0N071 | Delta globin | HBD |
| P02751 | Fibronectin | FN1 |
| A0A3B0J0F2 | "Adiponectin D | ADID |
| Q68DS3 | Uncharacterized protein DKFZp686H17246 (Fragment) | DKFZp686H17246 |
| A0A2U8J8K8 | Ig heavy chain variable region (Fragment) | IgH |
| A0A087WWT3 | Serum albumin | ALB |
| A0A2U8J923 | Ig heavy chain variable region (Fragment) | IgH |
| A0A0C4DGB6 | Serum albumin | ALB |
| Q9UHG3 | Prenylcysteine oxidase 1 | PCYOX1 |
| A0A2U8J8K4 | Ig heavy chain variable region (Fragment) | IgH |
| A0A2U8J970 | Ig heavy chain variable region (Fragment) | IgH |
| Q6MZQ6 | Uncharacterized protein DKFZp686G11190 | DKFZp686G11190 |
| Q8N355 | IGL@ protein | IGL@ |
| P01833 | Polymeric immunoglobulin receptor | PIGR |
| P00748 | Coagulation factor XII | F12 |
| A0A024RBS0 | "RNA helicase | DDX55 |
| Q8N6N3 | UPF0690 protein C1orf52 | C1orf52 |
| P06727 | Apolipoprotein A-IV | APOA4 |
| P60709 | "Actin, cytoplasmic 1 | ACTB |
| A0A2U8J974 | Ig heavy chain variable region (Fragment) | IgH |
| C9JF17 | Apolipoprotein D (Fragment) | APOD |
| E9PHK0 | Tetranectin | CLEC3B |
| A0PJA6 | TF protein (Fragment) | TF |
| P02768 | Albumin | ALB |
| A0A087X0M8 | "Neural cell adhesion molecule L1-like protein | CHL1 |
| Q9UGM5 | Fetuin-B | FETUB |
| A6XND0 | Insulin-like growth factor-binding protein 3 | IGFBP3 |
| Q6UXB8 | Peptidase inhibitor 16 | PI16 |
| P80108 | Phosphatidylinositol-glycan-specific phospholipase D | GPLD1 |
| A0A024R6N9 | "Serpin peptidase inhibitor, clade A (Alpha-1 antiproteinase, antitrypsin), member 5, isoform CRA_a | SERPINA5 |
| P00488 | Coagulation factor XIII A chain | F13A1 |
| B7WNR0 | Serum albumin | ALB |
| D6RFY8 | T-complex protein 11 homolog | TCP11 |
| A0A024RAA7 | Adiponectin B | ADIB |
| P11226 | Mannose-binding protein C | MBL2 |
| P02760 | Protein AMBP | AMBP |
| A0A024R6I9 | "Serpin peptidase inhibitor, clade A (Alpha-1 antiproteinase, antitrypsin), member 4, isoform CRA_a | SERPINA4 |
| A0A2Y9CYF7 | Ig heavy chain variable region (Fragment) | IgH |
| A0A087X1J7 | Glutathione peroxidase | GPX3 |
| A2RTY6 | "Inter-alpha (Globulin) inhibitor H2 | ITIH2 |
| A0A0F7TD49 | IGHV3-7 protein (Fragment) | IGHV3-7 |
| P00915 | "Carbonic anhydrase 1 | CA1 |
| Q14520 | Hyaluronan-binding protein 2 | HABP2 |
| P33151 | "Cadherin-5 | CDH5 |
| P27169 | Serum paraoxonase/arylesterase 1 | PON1 |
| P00742 | Coagulation factor X | F10 |
| A0A2U8J9A8 | Ig heavy chain variable region (Fragment) | IgH |
| A0A0S2Z3Y1 | Lectin galactoside-binding soluble 3 binding protein isoform 1 (Fragment) | LGALS3BP |
| A0A1S5UZ39 | Hemoglobin subunit alpha | HBA2 |
| A0A140T9C0 | Tenascin-X | TNXB |
| Q68DR3 | Uncharacterized protein DKFZp779H1622 (Fragment) | DKFZp779H1622 |
| P78369 | Claudin-10 | CLDN10 |
| O75882 | Attractin | ATRN |
| A0A0S2Z4F1 | "EGF containing fibulin-like extracellular matrix protein 1 isoform 1 (Fragment) | EFEMP1 |
| P11021 | Endoplasmic reticulum chaperone BiP | HSPA5 |
| Q53YY1 | "Angiotensinogen (Serine (Or cysteine) proteinase inhibitor, clade A (Alpha-1 antiproteinase, antitrypsin), member 8) | AGT |
| P05160 | Coagulation factor XIII B chain | F13B |
| A5PKX5 | Alpha-mannosidase | MAN2A1 |
| P00747 | Plasminogen | PLG |
| D9YZU5 | Beta-globin | HBB |
| A0A0S2Z3D5 | Apolipoprotein E isoform 1 (Fragment) | APOE |
| Q03591 | Complement factor H-related protein 1 | CFHR1 |
| A0A024RAG6 | Adiponectin C | ADIC |
| P48740 | Mannan-binding lectin serine protease 1 | MASP1 |
| A3KPE2 | Apolipoprotein C-III | APOC3 |
| A0A024RAB9 | Adiponectin A | ADIA |
| P43251 | Biotinidase | BTD |
| A0A0S2Z3F6 | Cholesteryl ester transfer protein plasma isoform 1 (Fragment) | CETP |
| Q04756 | Hepatocyte growth factor activator | HGFAC |
| P19827 | Inter-alpha-trypsin inhibitor heavy chain H1 | ITIH1 |
| E9KL23 | Epididymis secretory sperm binding protein Li 44a | SERPINA1 |
| V9HWI6 | Epididymis secretory protein Li 51 | HEL-S-51 |
| A0A024R8G3 | "Prostaglandin D2 synthase 21kDa (Brain), isoform CRA_a | PTGDS |
| P22792 | Carboxypeptidase N subunit 2 | CPN2 |
| A0A024R944 | "Serpin peptidase inhibitor, clade C (Antithrombin), member 1, isoform CRA_a | SERPINC1 |
| P02753 | Retinol-binding protein 4 | RBP4 |
| D9ZGG2 | Vitronectin | VTN |
| A0A024RDT4 | "Lymphocyte cytosolic protein 1 (L-plastin), isoform CRA_a | LCP1 |
| P02763 | Alpha-1-acid glycoprotein 1 | ORM1 |
| P03950 | Angiogenin | ANG |
| A0A2U8J969 | Ig heavy chain variable region (Fragment) | IgH |
| A0A024R035 | "Complement component 9, isoform CRA_a | C9 |
| P01591 | Immunoglobulin J chain | JCHAIN |
| A2N2F4 | VK3 protein (Fragment) | VK3 |
| A0A2U8J8Y8 | Ig heavy chain variable region (Fragment) | IgH |
| P00738 | Haptoglobin | HP |
| P18428 | Lipopolysaccharide-binding protein | LBP |
| Q65ZC9 | Single-chain Fv (Fragment) | scFv |
| A2VDG3 | "CSF1R protein (Fragment) | CSF1R |
| B6EDE2 | Epididymis luminal protein 180 (Fragment) | HEL180 |
| A0A0A0MS14 | Immunoglobulin heavy variable 1-45 | IGHV1-45 |
| O75460 | Serine/threonine-protein kinase/endoribonuclease IRE1 | ERN1 |
| A0A0U4DJF7 | Haptoglobin (Fragment) | HP |

Table S10: Common DEGs between the TB6 vs. TB0 group and the TB0 vs. HC group

| Gene |
| --- |
| GC |
| HBD |
| FN1 |
| DKFZp686H17246 |
| IgH |
| ALB |
| IGL@ |
| F12 |
| DDX55 |
| C1orf52 |
| APOA4 |
| ACTB |
| APOD |
| CLEC3B |
| TF |
| CHL1 |
| FETUB |
| IGFBP3 |
| PI16 |
| SERPINA5 |
| F13A1 |
| SERPINA4 |
| GPX3 |
| HABP2 |
| PON1 |
| TNXB |
| DKFZp779H1622 |
| CLDN10 |
| APOC3 |
| BTD |
| RBP4 |
| LCP1 |
| ORM1 |
| C9 |
| JCHAIN |
| HP |
| LBP |
| scFv |
| ERN1 |

Table S11: Enrichment analysis results of DEGs in the progression stage of pulmonary tuberculosis

| Category | ID | Description | GeneRatio | FoldEnrichment | pvalue | p.adjust | geneID | Count |
| --- | --- | --- | --- | --- | --- | --- | --- | --- |
| BP | GO:0002526 | acute inflammatory response | 9/62 | 25.60 | 6.80E-11 | 8.30E-08 | CRP/HP/FCGR3A/ORM2/LBP/ORM1/CD163/F12/FN1 | 9 |
| BP | GO:0006953 | acute-phase response | 7/62 | 44.39 | 1.93E-10 | 1.18E-07 | CRP/HP/ORM2/LBP/ORM1/CD163/FN1 | 7 |
| BP | GO:0006959 | humoral immune response | 10/62 | 11.80 | 1.10E-08 | 4.49E-06 | LYZ/CFHR4/JCHAIN/C4A/C9/C4B/FGB/IGHG4/C2/TF | 10 |
| BP | GO:0006956 | complement activation | 6/62 | 27.26 | 8.38E-08 | 2.56E-05 | CFHR4/C4A/C9/C4B/IGHG4/C2 | 6 |
| BP | GO:0006958 | complement activation, classical pathway | 5/62 | 36.23 | 2.52E-07 | 6.15E-05 | C4A/C9/C4B/IGHG4/C2 | 5 |
| BP | GO:0002455 | humoral immune response mediated by circulating immunoglobulin | 5/62 | 27.67 | 9.96E-07 | 2.03E-04 | C4A/C9/C4B/IGHG4/C2 | 5 |
| BP | GO:0042742 | defense response to bacterium | 9/62 | 8.30 | 1.23E-06 | 2.15E-04 | CRP/HP/LYZ/JCHAIN/LBP/FGB/PGLYRP2/IGHG4/TF | 9 |
| BP | GO:0006869 | lipid transport | 10/62 | 6.72 | 2.03E-06 | 3.09E-04 | CFHR4/LBP/SERPINA5/APOL1/APOC3/APOD/APOA4/RBP4/PON1/APOC4 | 10 |
| BP | GO:0042744 | hydrogen peroxide catabolic process | 4/62 | 39.27 | 3.11E-06 | 4.22E-04 | HP/APOA4/GPX3/HBD | 4 |
| BP | GO:2000425 | regulation of apoptotic cell clearance | 3/62 | 91.31 | 3.99E-06 | 4.87E-04 | C4A/C4B/C2 | 3 |
| BP | GO:0016064 | immunoglobulin mediated immune response | 7/62 | 10.39 | 4.78E-06 | 5.31E-04 | FCGR3A/C4A/C9/C4B/IGHG4/IGHV3-20/C2 | 7 |
| BP | GO:0019724 | B cell mediated immunity | 7/62 | 10.24 | 5.26E-06 | 5.35E-04 | FCGR3A/C4A/C9/C4B/IGHG4/IGHV3-20/C2 | 7 |
| BP | GO:0001895 | retina homeostasis | 5/62 | 18.33 | 7.75E-06 | 7.28E-04 | LYZ/JCHAIN/ALB/ACTB/TF | 5 |
| BP | GO:0098869 | cellular oxidant detoxification | 5/62 | 15.37 | 1.83E-05 | 1.49E-03 | HP/ALB/APOA4/GPX3/HBD | 5 |
| BP | GO:0072378 | blood coagulation, fibrin clot formation | 3/62 | 57.07 | 1.83E-05 | 1.49E-03 | FGB/F13A1/F12 | 3 |
| BP | GO:0009636 | response to toxic substance | 7/62 | 8.26 | 2.13E-05 | 1.60E-03 | HP/PON3/ALB/APOA4/GPX3/PON1/HBD | 7 |
| BP | GO:0008228 | opsonization | 3/62 | 53.71 | 2.22E-05 | 1.60E-03 | CRP/LBP/C4B | 3 |
| BP | GO:0042743 | hydrogen peroxide metabolic process | 4/62 | 22.54 | 2.95E-05 | 2.00E-03 | HP/APOA4/GPX3/HBD | 4 |
| BP | GO:0072376 | protein activation cascade | 3/62 | 48.06 | 3.15E-05 | 2.03E-03 | FGB/F13A1/F12 | 3 |
| BP | GO:1990748 | cellular detoxification | 5/62 | 12.79 | 4.45E-05 | 2.72E-03 | HP/ALB/APOA4/GPX3/HBD | 5 |
| BP | GO:0097237 | cellular response to toxic substance | 5/62 | 11.89 | 6.30E-05 | 3.67E-03 | HP/ALB/APOA4/GPX3/HBD | 5 |
| BP | GO:0019730 | antimicrobial humoral response | 5/62 | 11.44 | 7.56E-05 | 4.20E-03 | LYZ/JCHAIN/FGB/IGHG4/TF | 5 |
| BP | GO:0019731 | antibacterial humoral response | 4/62 | 17.39 | 8.23E-05 | 4.23E-03 | JCHAIN/FGB/IGHG4/TF | 4 |
| BP | GO:0098581 | detection of external biotic stimulus | 3/62 | 35.12 | 8.32E-05 | 4.23E-03 | LBP/C4B/PGLYRP2 | 3 |
| BP | GO:0031639 | plasminogen activation | 3/62 | 33.82 | 9.34E-05 | 4.56E-03 | FGB/F12/CLEC3B | 3 |
| BP | GO:0006910 | phagocytosis, recognition | 3/62 | 31.48 | 1.16E-04 | 5.31E-03 | CRP/LBP/C4B | 3 |
| BP | GO:0072593 | reactive oxygen species metabolic process | 6/62 | 7.80 | 1.17E-04 | 5.31E-03 | CRP/HP/PON3/APOA4/GPX3/HBD | 6 |
| BP | GO:0008037 | cell recognition | 5/62 | 9.88 | 1.51E-04 | 6.58E-03 | CRP/FETUB/LBP/C4B/MSN | 5 |
| BP | GO:0098754 | detoxification | 5/62 | 9.63 | 1.70E-04 | 7.16E-03 | HP/ALB/APOA4/GPX3/HBD | 5 |
| BP | GO:0002449 | lymphocyte mediated immunity | 7/62 | 5.79 | 1.98E-04 | 8.07E-03 | FCGR3A/C4A/C9/C4B/IGHG4/IGHV3-20/C2 | 7 |
| BP | GO:0002460 | adaptive immune response based on somatic recombination of immune receptors built from immunoglobulin superfamily domains | 7/62 | 5.61 | 2.41E-04 | 9.49E-03 | FCGR3A/C4A/C9/C4B/IGHG4/IGHV3-20/C2 | 7 |
| BP | GO:0001894 | tissue homeostasis | 6/62 | 6.55 | 3.04E-04 | 1.10E-02 | LYZ/JCHAIN/ALB/ACTB/RBP4/TF | 6 |
| BP | GO:0060249 | anatomical structure homeostasis | 6/62 | 6.55 | 3.04E-04 | 1.10E-02 | LYZ/JCHAIN/ALB/ACTB/RBP4/TF | 6 |
| BP | GO:0009595 | detection of biotic stimulus | 3/62 | 22.83 | 3.06E-04 | 1.10E-02 | LBP/C4B/PGLYRP2 | 3 |
| BP | GO:1905952 | regulation of lipid localization | 5/62 | 8.32 | 3.36E-04 | 1.17E-02 | CRP/APOC3/APOA4/PON1/APOC4 | 5 |
| BP | GO:0055090 | acylglycerol homeostasis | 3/62 | 21.23 | 3.80E-04 | 1.22E-02 | APOC3/APOA4/APOC4 | 3 |
| BP | GO:0070328 | triglyceride homeostasis | 3/62 | 21.23 | 3.80E-04 | 1.22E-02 | APOC3/APOA4/APOC4 | 3 |
| BP | GO:0030100 | regulation of endocytosis | 6/62 | 6.28 | 3.80E-04 | 1.22E-02 | C4A/C4B/APOC3/ACTB/C2/TF | 6 |
| BP | GO:0032760 | positive regulation of tumor necrosis factor production | 4/62 | 11.49 | 4.08E-04 | 1.28E-02 | FCGR3A/ORM2/LBP/ORM1 | 4 |
| BP | GO:0014012 | peripheral nervous system axon regeneration | 2/62 | 60.87 | 4.70E-04 | 1.37E-02 | APOD/APOA4 | 2 |
| BP | GO:0071803 | positive regulation of podosome assembly | 2/62 | 60.87 | 4.70E-04 | 1.37E-02 | LCP1/MSN | 2 |
| BP | GO:1903557 | positive regulation of tumor necrosis factor superfamily cytokine production | 4/62 | 11.07 | 4.70E-04 | 1.37E-02 | FCGR3A/ORM2/LBP/ORM1 | 4 |
| BP | GO:0043277 | apoptotic cell clearance | 3/62 | 19.02 | 5.26E-04 | 1.49E-02 | C4A/C4B/C2 | 3 |
| BP | GO:0050830 | defense response to Gram-positive bacterium | 4/62 | 10.32 | 6.13E-04 | 1.70E-02 | CRP/LYZ/LBP/PGLYRP2 | 4 |
| BP | GO:0034370 | triglyceride-rich lipoprotein particle remodeling | 2/62 | 50.73 | 6.86E-04 | 1.82E-02 | APOC3/APOA4 | 2 |
| BP | GO:0034372 | very-low-density lipoprotein particle remodeling | 2/62 | 50.73 | 6.86E-04 | 1.82E-02 | APOC3/APOA4 | 2 |
| BP | GO:0010896 | regulation of triglyceride catabolic process | 2/62 | 46.82 | 8.09E-04 | 1.99E-02 | APOC3/APOA4 | 2 |
| BP | GO:0032490 | detection of molecule of bacterial origin | 2/62 | 46.82 | 8.09E-04 | 1.99E-02 | LBP/C4B | 2 |
| BP | GO:0071801 | regulation of podosome assembly | 2/62 | 46.82 | 8.09E-04 | 1.99E-02 | LCP1/MSN | 2 |
| BP | GO:0002443 | leukocyte mediated immunity | 7/62 | 4.57 | 8.15E-04 | 1.99E-02 | FCGR3A/C4A/C9/C4B/IGHG4/IGHV3-20/C2 | 7 |
| BP | GO:0033700 | phospholipid efflux | 2/62 | 43.48 | 9.42E-04 | 2.26E-02 | APOC3/APOA4 | 2 |
| BP | GO:0031638 | zymogen activation | 3/62 | 15.48 | 9.63E-04 | 2.26E-02 | FGB/F12/CLEC3B | 3 |
| BP | GO:0042157 | lipoprotein metabolic process | 4/62 | 9.02 | 1.01E-03 | 2.34E-02 | APOL1/APOC3/APOD/APOA4 | 4 |
| BP | GO:0006909 | phagocytosis | 5/62 | 6.42 | 1.08E-03 | 2.45E-02 | CRP/LBP/C4A/C4B/C2 | 5 |
| BP | GO:0034375 | high-density lipoprotein particle remodeling | 2/62 | 38.04 | 1.24E-03 | 2.70E-02 | APOC3/APOA4 | 2 |
| BP | GO:0051004 | regulation of lipoprotein lipase activity | 2/62 | 38.04 | 1.24E-03 | 2.70E-02 | APOC3/APOA4 | 2 |
| BP | GO:0098974 | postsynaptic actin cytoskeleton organization | 2/62 | 35.81 | 1.40E-03 | 3.00E-02 | ACTB/ACTBL2 | 2 |
| BP | GO:0010755 | regulation of plasminogen activation | 2/62 | 33.82 | 1.57E-03 | 3.20E-02 | F12/CLEC3B | 2 |
| BP | GO:0033194 | response to hydroperoxide | 2/62 | 33.82 | 1.57E-03 | 3.20E-02 | APOA4/GPX3 | 2 |
| BP | GO:0060263 | regulation of respiratory burst | 2/62 | 33.82 | 1.57E-03 | 3.20E-02 | JCHAIN/LBP | 2 |
| BP | GO:0045807 | positive regulation of endocytosis | 4/62 | 7.85 | 1.69E-03 | 3.29E-02 | C4A/C4B/C2/TF | 4 |
| BP | GO:0006801 | superoxide metabolic process | 3/62 | 12.68 | 1.71E-03 | 3.29E-02 | CRP/PON3/APOA4 | 3 |
| BP | GO:0050766 | positive regulation of phagocytosis | 3/62 | 12.68 | 1.71E-03 | 3.29E-02 | C4A/C4B/C2 | 3 |
| BP | GO:0032928 | regulation of superoxide anion generation | 2/62 | 32.04 | 1.75E-03 | 3.29E-02 | CRP/PON3 | 2 |
| BP | GO:0071800 | podosome assembly | 2/62 | 32.04 | 1.75E-03 | 3.29E-02 | LCP1/MSN | 2 |
| BP | GO:0033344 | cholesterol efflux | 3/62 | 12.51 | 1.78E-03 | 3.30E-02 | APOC3/APOA4/PON1 | 3 |
| BP | GO:0043691 | reverse cholesterol transport | 2/62 | 30.44 | 1.94E-03 | 3.49E-02 | APOC3/APOA4 | 2 |
| BP | GO:0099188 | postsynaptic cytoskeleton organization | 2/62 | 30.44 | 1.94E-03 | 3.49E-02 | ACTB/ACTBL2 | 2 |
| BP | GO:0032371 | regulation of sterol transport | 3/62 | 11.41 | 2.32E-03 | 4.04E-02 | APOC3/APOA4/PON1 | 3 |
| BP | GO:0032374 | regulation of cholesterol transport | 3/62 | 11.41 | 2.32E-03 | 4.04E-02 | APOC3/APOA4/PON1 | 3 |
| BP | GO:0010954 | positive regulation of protein processing | 2/62 | 24.35 | 3.04E-03 | 5.15E-02 | F12/CLEC3B | 2 |
| BP | GO:0043567 | regulation of insulin-like growth factor receptor signaling pathway | 2/62 | 24.35 | 3.04E-03 | 5.15E-02 | IGFBP6/IGFBP3 | 2 |
| BP | GO:0032640 | tumor necrosis factor production | 4/62 | 6.55 | 3.27E-03 | 5.35E-02 | FCGR3A/ORM2/LBP/ORM1 | 4 |
| BP | GO:0032680 | regulation of tumor necrosis factor production | 4/62 | 6.55 | 3.27E-03 | 5.35E-02 | FCGR3A/ORM2/LBP/ORM1 | 4 |
| BP | GO:0015850 | organic hydroxy compound transport | 5/62 | 4.94 | 3.39E-03 | 5.35E-02 | APOC3/ACTB/APOA4/RBP4/PON1 | 5 |
| BP | GO:0031640 | killing of cells of another organism | 3/62 | 9.92 | 3.45E-03 | 5.35E-02 | LYZ/C9/APOL1 | 3 |
| BP | GO:0141061 | disruption of cell in another organism | 3/62 | 9.92 | 3.45E-03 | 5.35E-02 | LYZ/C9/APOL1 | 3 |
| BP | GO:0034377 | plasma lipoprotein particle assembly | 2/62 | 22.54 | 3.54E-03 | 5.35E-02 | APOC3/APOA4 | 2 |
| BP | GO:0042730 | fibrinolysis | 2/62 | 22.54 | 3.54E-03 | 5.35E-02 | FGB/F12 | 2 |
| BP | GO:1903319 | positive regulation of protein maturation | 2/62 | 22.54 | 3.54E-03 | 5.35E-02 | F12/CLEC3B | 2 |
| BP | GO:0071706 | tumor necrosis factor superfamily cytokine production | 4/62 | 6.37 | 3.59E-03 | 5.35E-02 | FCGR3A/ORM2/LBP/ORM1 | 4 |
| BP | GO:1903555 | regulation of tumor necrosis factor superfamily cytokine production | 4/62 | 6.37 | 3.59E-03 | 5.35E-02 | FCGR3A/ORM2/LBP/ORM1 | 4 |
| BP | GO:0018149 | peptide cross-linking | 2/62 | 21.74 | 3.80E-03 | 5.40E-02 | F13A1/FN1 | 2 |
| BP | GO:0019433 | triglyceride catabolic process | 2/62 | 21.74 | 3.80E-03 | 5.40E-02 | APOC3/APOA4 | 2 |
| BP | GO:0055022 | negative regulation of cardiac muscle tissue growth | 2/62 | 21.74 | 3.80E-03 | 5.40E-02 | PI16/RBP4 | 2 |
| BP | GO:0061117 | negative regulation of heart growth | 2/62 | 21.74 | 3.80E-03 | 5.40E-02 | PI16/RBP4 | 2 |
| BP | GO:0141060 | disruption of anatomical structure in another organism | 3/62 | 9.22 | 4.23E-03 | 5.94E-02 | LYZ/C9/APOL1 | 3 |
| BP | GO:0065005 | protein-lipid complex assembly | 2/62 | 20.29 | 4.35E-03 | 5.97E-02 | APOC3/APOA4 | 2 |
| BP | GO:0050764 | regulation of phagocytosis | 3/62 | 9.13 | 4.35E-03 | 5.97E-02 | C4A/C4B/C2 | 3 |
| BP | GO:0000302 | response to reactive oxygen species | 4/62 | 5.94 | 4.62E-03 | 6.23E-02 | HP/ERN1/APOD/APOA4 | 4 |
| BP | GO:0090322 | regulation of superoxide metabolic process | 2/62 | 19.64 | 4.65E-03 | 6.23E-02 | CRP/PON3 | 2 |
| BP | GO:0006641 | triglyceride metabolic process | 3/62 | 8.61 | 5.12E-03 | 6.80E-02 | APOC3/APOA4/TNXB | 3 |
| BP | GO:0034368 | protein-lipid complex remodeling | 2/62 | 18.45 | 5.25E-03 | 6.82E-02 | APOC3/APOA4 | 2 |
| BP | GO:0034369 | plasma lipoprotein particle remodeling | 2/62 | 18.45 | 5.25E-03 | 6.82E-02 | APOC3/APOA4 | 2 |
| BP | GO:0002323 | natural killer cell activation involved in immune response | 2/62 | 17.90 | 5.57E-03 | 7.16E-02 | FCGR3A/PGLYRP2 | 2 |
| BP | GO:0051346 | negative regulation of hydrolase activity | 4/62 | 5.58 | 5.73E-03 | 7.29E-02 | FETUB/SERPINA5/APOC3/SERPINA4 | 4 |
| BP | GO:0034367 | protein-containing complex remodeling | 2/62 | 17.39 | 5.89E-03 | 7.42E-02 | APOC3/APOA4 | 2 |
| BP | GO:0001906 | cell killing | 4/62 | 5.46 | 6.20E-03 | 7.73E-02 | FCGR3A/LYZ/C9/APOL1 | 4 |
| BP | GO:0007596 | blood coagulation | 4/62 | 5.43 | 6.30E-03 | 7.77E-02 | FGB/F13A1/F12/ACTB | 4 |
| BP | GO:0046621 | negative regulation of organ growth | 2/62 | 16.45 | 6.57E-03 | 8.02E-02 | PI16/RBP4 | 2 |
| BP | GO:0050817 | coagulation | 4/62 | 5.32 | 6.80E-03 | 8.12E-02 | FGB/F13A1/F12/ACTB | 4 |
| BP | GO:0010737 | protein kinase A signaling | 2/62 | 16.02 | 6.92E-03 | 8.12E-02 | TTN/LCP1 | 2 |
| BP | GO:0046461 | neutral lipid catabolic process | 2/62 | 16.02 | 6.92E-03 | 8.12E-02 | APOC3/APOA4 | 2 |
| BP | GO:0046464 | acylglycerol catabolic process | 2/62 | 16.02 | 6.92E-03 | 8.12E-02 | APOC3/APOA4 | 2 |
| BP | GO:0007599 | hemostasis | 4/62 | 5.27 | 7.01E-03 | 8.15E-02 | FGB/F13A1/F12/ACTB | 4 |
| BP | GO:0045730 | respiratory burst | 2/62 | 15.22 | 7.65E-03 | 8.81E-02 | JCHAIN/LBP | 2 |
| BP | GO:0007160 | cell-matrix adhesion | 4/62 | 5.09 | 7.88E-03 | 9.00E-02 | FGB/APOD/TNXB/FN1 | 4 |
| BP | GO:0042554 | superoxide anion generation | 2/62 | 14.85 | 8.02E-03 | 9.07E-02 | CRP/PON3 | 2 |
| BP | GO:0001818 | negative regulation of cytokine production | 5/62 | 3.99 | 8.25E-03 | 9.24E-02 | LBP/ORM1/APOD/PGLYRP2/FN1 | 5 |
| BP | GO:0071634 | regulation of transforming growth factor beta production | 2/62 | 14.49 | 8.40E-03 | 9.33E-02 | TNXB/FN1 | 2 |
| BP | GO:0010951 | negative regulation of endopeptidase activity | 3/62 | 7.13 | 8.62E-03 | 9.37E-02 | FETUB/SERPINA5/SERPINA4 | 3 |
| BP | GO:0030301 | cholesterol transport | 3/62 | 7.13 | 8.62E-03 | 9.37E-02 | APOC3/APOA4/PON1 | 3 |
| BP | GO:0032814 | regulation of natural killer cell activation | 2/62 | 14.16 | 8.80E-03 | 9.37E-02 | FCGR3A/PGLYRP2 | 2 |
| BP | GO:0045663 | positive regulation of myoblast differentiation | 2/62 | 14.16 | 8.80E-03 | 9.37E-02 | ACTB/IGFBP3 | 2 |
| BP | GO:0033002 | muscle cell proliferation | 4/62 | 4.93 | 8.83E-03 | 9.37E-02 | ERN1/APOD/RBP4/IGFBP3 | 4 |
| BP | GO:0090207 | regulation of triglyceride metabolic process | 2/62 | 13.83 | 9.20E-03 | 9.68E-02 | APOC3/APOA4 | 2 |
| BP | GO:0071604 | transforming growth factor beta production | 2/62 | 13.53 | 9.60E-03 | 1.00E-01 | TNXB/FN1 | 2 |
| BP | GO:0006639 | acylglycerol metabolic process | 3/62 | 6.81 | 9.76E-03 | 1.01E-01 | APOC3/APOA4/TNXB | 3 |
| BP | GO:0006638 | neutral lipid metabolic process | 3/62 | 6.76 | 9.96E-03 | 1.02E-01 | APOC3/APOA4/TNXB | 3 |
| BP | GO:0006979 | response to oxidative stress | 5/62 | 3.80 | 1.00E-02 | 1.02E-01 | HP/ERN1/APOD/APOA4/GPX3 | 5 |
| BP | GO:0010466 | negative regulation of peptidase activity | 3/62 | 6.66 | 1.04E-02 | 1.05E-01 | FETUB/SERPINA5/SERPINA4 | 3 |
| BP | GO:0030195 | negative regulation of blood coagulation | 2/62 | 12.68 | 1.09E-02 | 1.09E-01 | FGB/F12 | 2 |
| BP | GO:0008203 | cholesterol metabolic process | 3/62 | 6.52 | 1.10E-02 | 1.09E-01 | APOL1/APOA4/PON1 | 3 |
| BP | GO:0015918 | sterol transport | 3/62 | 6.48 | 1.12E-02 | 1.10E-01 | APOC3/APOA4/PON1 | 3 |
| BP | GO:1900047 | negative regulation of hemostasis | 2/62 | 12.42 | 1.13E-02 | 1.10E-01 | FGB/F12 | 2 |
| BP | GO:2000378 | negative regulation of reactive oxygen species metabolic process | 2/62 | 12.42 | 1.13E-02 | 1.10E-01 | HP/PON3 | 2 |
| BP | GO:0071827 | plasma lipoprotein particle organization | 2/62 | 12.17 | 1.18E-02 | 1.13E-01 | APOC3/APOA4 | 2 |
| BP | GO:0050714 | positive regulation of protein secretion | 3/62 | 6.34 | 1.19E-02 | 1.13E-01 | TTN/FGB/RBP4 | 3 |
| BP | GO:2000377 | regulation of reactive oxygen species metabolic process | 3/62 | 6.21 | 1.25E-02 | 1.16E-01 | CRP/HP/PON3 | 3 |
| BP | GO:0042060 | wound healing | 5/62 | 3.60 | 1.26E-02 | 1.16E-01 | FGB/F13A1/F12/ACTB/FN1 | 5 |
| BP | GO:0031103 | axon regeneration | 2/62 | 11.71 | 1.27E-02 | 1.16E-01 | APOD/APOA4 | 2 |
| BP | GO:0048009 | insulin-like growth factor receptor signaling pathway | 2/62 | 11.71 | 1.27E-02 | 1.16E-01 | IGFBP6/IGFBP3 | 2 |
| BP | GO:0050819 | negative regulation of coagulation | 2/62 | 11.71 | 1.27E-02 | 1.16E-01 | FGB/F12 | 2 |
| BP | GO:0071825 | protein-lipid complex organization | 2/62 | 11.49 | 1.31E-02 | 1.20E-01 | APOC3/APOA4 | 2 |
| BP | GO:1902652 | secondary alcohol metabolic process | 3/62 | 6.09 | 1.32E-02 | 1.20E-01 | APOL1/APOA4/PON1 | 3 |
| BP | GO:0010883 | regulation of lipid storage | 2/62 | 11.27 | 1.36E-02 | 1.22E-01 | CRP/APOC4 | 2 |
| BP | GO:0032368 | regulation of lipid transport | 3/62 | 5.97 | 1.39E-02 | 1.24E-01 | APOC3/APOA4/PON1 | 3 |
| BP | GO:0042304 | regulation of fatty acid biosynthetic process | 2/62 | 11.07 | 1.41E-02 | 1.25E-01 | APOC3/APOA4 | 2 |
| BP | GO:0016125 | sterol metabolic process | 3/62 | 5.93 | 1.42E-02 | 1.25E-01 | APOL1/APOA4/PON1 | 3 |
| BP | GO:0031102 | neuron projection regeneration | 2/62 | 10.49 | 1.56E-02 | 1.36E-01 | APOD/APOA4 | 2 |
| BP | GO:0007409 | axonogenesis | 5/62 | 3.40 | 1.58E-02 | 1.37E-01 | ACTB/CHL1/CNTN1/FN1/ACTBL2 | 5 |
| BP | GO:0051017 | actin filament bundle assembly | 3/62 | 5.67 | 1.60E-02 | 1.37E-01 | HSP90B1/LCP1/PFN1 | 3 |
| BP | GO:0006826 | iron ion transport | 2/62 | 10.32 | 1.61E-02 | 1.38E-01 | CP/TF | 2 |
| BP | GO:0061572 | actin filament bundle organization | 3/62 | 5.53 | 1.70E-02 | 1.44E-01 | HSP90B1/LCP1/PFN1 | 3 |
| BP | GO:0002366 | leukocyte activation involved in immune response | 4/62 | 4.04 | 1.72E-02 | 1.44E-01 | FCGR3A/LBP/LCP1/PGLYRP2 | 4 |
| BP | GO:0050994 | regulation of lipid catabolic process | 2/62 | 9.98 | 1.72E-02 | 1.44E-01 | APOC3/APOA4 | 2 |
| BP | GO:0032731 | positive regulation of interleukin-1 beta production | 2/62 | 9.82 | 1.77E-02 | 1.47E-01 | ORM2/ORM1 | 2 |
| BP | GO:0002263 | cell activation involved in immune response | 4/62 | 3.99 | 1.79E-02 | 1.48E-01 | FCGR3A/LBP/LCP1/PGLYRP2 | 4 |
| BP | GO:0007015 | actin filament organization | 5/62 | 3.28 | 1.81E-02 | 1.48E-01 | TTN/HSP90B1/LCP1/PFN1/TF | 5 |
| BP | GO:0048660 | regulation of smooth muscle cell proliferation | 3/62 | 5.34 | 1.87E-02 | 1.52E-01 | ERN1/APOD/IGFBP3 | 3 |
| BP | GO:0046503 | glycerolipid catabolic process | 2/62 | 9.36 | 1.93E-02 | 1.55E-01 | APOC3/APOA4 | 2 |
| BP | GO:0055021 | regulation of cardiac muscle tissue growth | 2/62 | 9.36 | 1.93E-02 | 1.55E-01 | PI16/RBP4 | 2 |
| BP | GO:0048659 | smooth muscle cell proliferation | 3/62 | 5.22 | 1.99E-02 | 1.57E-01 | ERN1/APOD/IGFBP3 | 3 |
| BP | GO:0055088 | lipid homeostasis | 3/62 | 5.22 | 1.99E-02 | 1.57E-01 | APOC3/APOA4/APOC4 | 3 |
| BP | GO:1905953 | negative regulation of lipid localization | 2/62 | 9.22 | 1.99E-02 | 1.57E-01 | CRP/APOC3 | 2 |
| BP | GO:0070613 | regulation of protein processing | 2/62 | 9.09 | 2.05E-02 | 1.60E-01 | F12/CLEC3B | 2 |
| BP | GO:0043086 | negative regulation of catalytic activity | 5/62 | 3.17 | 2.06E-02 | 1.60E-01 | HP/FETUB/SERPINA5/APOC3/SERPINA4 | 5 |
| BP | GO:0030193 | regulation of blood coagulation | 2/62 | 8.95 | 2.11E-02 | 1.63E-01 | FGB/F12 | 2 |
| BP | GO:2000401 | regulation of lymphocyte migration | 2/62 | 8.82 | 2.16E-02 | 1.66E-01 | APOD/MSN | 2 |
| BP | GO:0048662 | negative regulation of smooth muscle cell proliferation | 2/62 | 8.70 | 2.22E-02 | 1.69E-01 | APOD/IGFBP3 | 2 |
| BP | GO:1900046 | regulation of hemostasis | 2/62 | 8.70 | 2.22E-02 | 1.69E-01 | FGB/F12 | 2 |
| BP | GO:0008202 | steroid metabolic process | 4/62 | 3.72 | 2.25E-02 | 1.70E-01 | APOL1/APOA4/GC/PON1 | 4 |
| BP | GO:0060420 | regulation of heart growth | 2/62 | 8.57 | 2.28E-02 | 1.70E-01 | PI16/RBP4 | 2 |
| BP | GO:1903317 | regulation of protein maturation | 2/62 | 8.57 | 2.28E-02 | 1.70E-01 | F12/CLEC3B | 2 |
| BP | GO:0009988 | cell-cell recognition | 2/62 | 8.45 | 2.34E-02 | 1.71E-01 | FETUB/MSN | 2 |
| BP | GO:0070527 | platelet aggregation | 2/62 | 8.45 | 2.34E-02 | 1.71E-01 | FGB/ACTB | 2 |
| BP | GO:0001819 | positive regulation of cytokine production | 5/62 | 3.05 | 2.39E-02 | 1.71E-01 | FCGR3A/ORM2/LBP/ORM1/TNXB | 5 |
| BP | GO:0006879 | intracellular iron ion homeostasis | 2/62 | 8.34 | 2.40E-02 | 1.71E-01 | CP/TF | 2 |
| BP | GO:0032732 | positive regulation of interleukin-1 production | 2/62 | 8.34 | 2.40E-02 | 1.71E-01 | ORM2/ORM1 | 2 |
| BP | GO:0045661 | regulation of myoblast differentiation | 2/62 | 8.34 | 2.40E-02 | 1.71E-01 | ACTB/IGFBP3 | 2 |
| BP | GO:0050818 | regulation of coagulation | 2/62 | 8.34 | 2.40E-02 | 1.71E-01 | FGB/F12 | 2 |
| BP | GO:0072678 | T cell migration | 2/62 | 8.34 | 2.40E-02 | 1.71E-01 | APOD/MSN | 2 |
| BP | GO:0031099 | regeneration | 3/62 | 4.78 | 2.50E-02 | 1.76E-01 | LCP1/APOD/APOA4 | 3 |
| BP | GO:0035924 | cellular response to vascular endothelial growth factor stimulus | 2/62 | 8.12 | 2.53E-02 | 1.76E-01 | ERN1/TNXB | 2 |
| BP | GO:0061045 | negative regulation of wound healing | 2/62 | 8.12 | 2.53E-02 | 1.76E-01 | FGB/F12 | 2 |
| BP | GO:0006006 | glucose metabolic process | 3/62 | 4.73 | 2.56E-02 | 1.78E-01 | APOD/RBP4/IGFBP3 | 3 |
| BP | GO:0046470 | phosphatidylcholine metabolic process | 2/62 | 8.01 | 2.59E-02 | 1.79E-01 | APOA4/PON1 | 2 |
| BP | GO:0002377 | immunoglobulin production | 3/62 | 4.66 | 2.67E-02 | 1.83E-01 | IGKV3D-7/IGLV5-45/RBP4 | 3 |
| BP | GO:0032720 | negative regulation of tumor necrosis factor production | 2/62 | 7.80 | 2.72E-02 | 1.84E-01 | LBP/ORM1 | 2 |
| BP | GO:0060191 | regulation of lipase activity | 2/62 | 7.80 | 2.72E-02 | 1.84E-01 | APOC3/APOA4 | 2 |
| BP | GO:0031334 | positive regulation of protein-containing complex assembly | 3/62 | 4.61 | 2.74E-02 | 1.85E-01 | LCP1/MSN/PFN1 | 3 |
| BP | GO:0051235 | maintenance of location | 4/62 | 3.48 | 2.80E-02 | 1.88E-01 | CRP/HSP90B1/ALB/APOC4 | 4 |
| BP | GO:1903556 | negative regulation of tumor necrosis factor superfamily cytokine production | 2/62 | 7.61 | 2.85E-02 | 1.90E-01 | LBP/ORM1 | 2 |
| BP | GO:0031589 | cell-substrate adhesion | 4/62 | 3.42 | 2.95E-02 | 1.96E-01 | FGB/APOD/TNXB/FN1 | 4 |
| BP | GO:0010038 | response to metal ion | 4/62 | 3.39 | 3.03E-02 | 1.98E-01 | TTN/HSP90B1/FGB/TF | 4 |
| BP | GO:0042310 | vasoconstriction | 2/62 | 7.33 | 3.05E-02 | 1.98E-01 | CRP/FGB | 2 |
| BP | GO:1902117 | positive regulation of organelle assembly | 2/62 | 7.33 | 3.05E-02 | 1.98E-01 | LCP1/MSN | 2 |
| BP | GO:0048678 | response to axon injury | 2/62 | 7.25 | 3.12E-02 | 1.98E-01 | APOD/APOA4 | 2 |
| BP | GO:0050878 | regulation of body fluid levels | 4/62 | 3.34 | 3.19E-02 | 1.98E-01 | FGB/F13A1/F12/ACTB | 4 |
| BP | GO:0014866 | skeletal myofibril assembly | 1/62 | 30.44 | 3.24E-02 | 1.98E-01 | TTN | 1 |
| BP | GO:0032815 | negative regulation of natural killer cell activation | 1/62 | 30.44 | 3.24E-02 | 1.98E-01 | PGLYRP2 | 1 |
| BP | GO:0032819 | positive regulation of natural killer cell proliferation | 1/62 | 30.44 | 3.24E-02 | 1.98E-01 | FCGR3A | 1 |
| BP | GO:0048251 | elastic fiber assembly | 1/62 | 30.44 | 3.24E-02 | 1.98E-01 | TNXB | 1 |
| BP | GO:0060068 | vagina development | 1/62 | 30.44 | 3.24E-02 | 1.98E-01 | RBP4 | 1 |
| BP | GO:0070391 | response to lipoteichoic acid | 1/62 | 30.44 | 3.24E-02 | 1.98E-01 | LBP | 1 |
| BP | GO:0071223 | cellular response to lipoteichoic acid | 1/62 | 30.44 | 3.24E-02 | 1.98E-01 | LBP | 1 |
| BP | GO:0071287 | cellular response to manganese ion | 1/62 | 30.44 | 3.24E-02 | 1.98E-01 | HSP90B1 | 1 |
| BP | GO:1901388 | regulation of transforming growth factor beta activation | 1/62 | 30.44 | 3.24E-02 | 1.98E-01 | TNXB | 1 |
| BP | GO:1904729 | regulation of intestinal lipid absorption | 1/62 | 30.44 | 3.24E-02 | 1.98E-01 | APOA4 | 1 |
| BP | GO:1905918 | regulation of CoA-transferase activity | 1/62 | 30.44 | 3.24E-02 | 1.98E-01 | APOA4 | 1 |
| BP | GO:0055013 | cardiac muscle cell development | 2/62 | 7.08 | 3.25E-02 | 1.98E-01 | TTN/PI16 | 2 |
| BP | GO:0002285 | lymphocyte activation involved in immune response | 3/62 | 4.29 | 3.30E-02 | 1.99E-01 | FCGR3A/LCP1/PGLYRP2 | 3 |
| BP | GO:0006066 | alcohol metabolic process | 4/62 | 3.26 | 3.42E-02 | 2.05E-01 | APOL1/APOA4/RBP4/PON1 | 4 |
| BP | GO:0046849 | bone remodeling | 2/62 | 6.84 | 3.46E-02 | 2.05E-01 | SPP2/TF | 2 |
| BP | GO:0050829 | defense response to Gram-negative bacterium | 2/62 | 6.84 | 3.46E-02 | 2.05E-01 | LYZ/LBP | 2 |
| BP | GO:0055017 | cardiac muscle tissue growth | 2/62 | 6.84 | 3.46E-02 | 2.05E-01 | PI16/RBP4 | 2 |
| BP | GO:0010982 | regulation of high-density lipoprotein particle clearance | 1/62 | 27.67 | 3.56E-02 | 2.07E-01 | APOC3 | 1 |
| BP | GO:0034975 | protein folding in endoplasmic reticulum | 1/62 | 27.67 | 3.56E-02 | 2.07E-01 | HSP90B1 | 1 |
| BP | GO:0043320 | natural killer cell degranulation | 1/62 | 27.67 | 3.56E-02 | 2.07E-01 | FCGR3A | 1 |
| BP | GO:0098885 | modification of postsynaptic actin cytoskeleton | 1/62 | 27.67 | 3.56E-02 | 2.07E-01 | PFN1 | 1 |
| BP | GO:0010810 | regulation of cell-substrate adhesion | 3/62 | 4.15 | 3.58E-02 | 2.07E-01 | FGB/APOD/FN1 | 3 |
| BP | GO:0097006 | regulation of plasma lipoprotein particle levels | 2/62 | 6.69 | 3.61E-02 | 2.08E-01 | APOC3/APOA4 | 2 |
| BP | GO:0001738 | morphogenesis of a polarized epithelium | 2/62 | 6.62 | 3.68E-02 | 2.08E-01 | ACTB/MSN | 2 |
| BP | GO:0055006 | cardiac cell development | 2/62 | 6.55 | 3.75E-02 | 2.08E-01 | TTN/PI16 | 2 |
| BP | GO:0019915 | lipid storage | 2/62 | 6.48 | 3.83E-02 | 2.08E-01 | CRP/APOC4 | 2 |
| BP | GO:0032817 | regulation of natural killer cell proliferation | 1/62 | 25.36 | 3.87E-02 | 2.08E-01 | FCGR3A | 1 |
| BP | GO:0034379 | very-low-density lipoprotein particle assembly | 1/62 | 25.36 | 3.87E-02 | 2.08E-01 | APOC3 | 1 |
| BP | GO:0035112 | genitalia morphogenesis | 1/62 | 25.36 | 3.87E-02 | 2.08E-01 | RBP4 | 1 |
| BP | GO:0042117 | monocyte activation | 1/62 | 25.36 | 3.87E-02 | 2.08E-01 | FN1 | 1 |
| BP | GO:0071394 | cellular response to testosterone stimulus | 1/62 | 25.36 | 3.87E-02 | 2.08E-01 | MSN | 1 |
| BP | GO:0071635 | negative regulation of transforming growth factor beta production | 1/62 | 25.36 | 3.87E-02 | 2.08E-01 | FN1 | 1 |
| BP | GO:1902946 | protein localization to early endosome | 1/62 | 25.36 | 3.87E-02 | 2.08E-01 | MSN | 1 |
| BP | GO:1904478 | regulation of intestinal absorption | 1/62 | 25.36 | 3.87E-02 | 2.08E-01 | APOA4 | 1 |
| BP | GO:1905668 | positive regulation of protein localization to endosome | 1/62 | 25.36 | 3.87E-02 | 2.08E-01 | MSN | 1 |
| BP | GO:0007044 | cell-substrate junction assembly | 2/62 | 6.41 | 3.90E-02 | 2.08E-01 | APOD/FN1 | 2 |
| BP | GO:0046486 | glycerolipid metabolic process | 4/62 | 3.12 | 3.92E-02 | 2.08E-01 | APOC3/APOA4/PON1/TNXB | 4 |
| BP | GO:0034109 | homotypic cell-cell adhesion | 2/62 | 6.34 | 3.98E-02 | 2.08E-01 | FGB/ACTB | 2 |
| BP | GO:0002685 | regulation of leukocyte migration | 3/62 | 3.97 | 4.00E-02 | 2.08E-01 | LBP/APOD/MSN | 3 |
| BP | GO:0060419 | heart growth | 2/62 | 6.28 | 4.05E-02 | 2.08E-01 | PI16/RBP4 | 2 |
| BP | GO:1903035 | negative regulation of response to wounding | 2/62 | 6.28 | 4.05E-02 | 2.08E-01 | FGB/F12 | 2 |
| BP | GO:0019318 | hexose metabolic process | 3/62 | 3.92 | 4.13E-02 | 2.08E-01 | APOD/RBP4/IGFBP3 | 3 |
| BP | GO:0010642 | negative regulation of platelet-derived growth factor receptor signaling pathway | 1/62 | 23.41 | 4.19E-02 | 2.08E-01 | APOD | 1 |
| BP | GO:0032823 | regulation of natural killer cell differentiation | 1/62 | 23.41 | 4.19E-02 | 2.08E-01 | PGLYRP2 | 1 |
| BP | GO:0034145 | positive regulation of toll-like receptor 4 signaling pathway | 1/62 | 23.41 | 4.19E-02 | 2.08E-01 | LBP | 1 |
| BP | GO:0034333 | adherens junction assembly | 1/62 | 23.41 | 4.19E-02 | 2.08E-01 | ACTB | 1 |
| BP | GO:0036363 | transforming growth factor beta activation | 1/62 | 23.41 | 4.19E-02 | 2.08E-01 | TNXB | 1 |
| BP | GO:0043568 | positive regulation of insulin-like growth factor receptor signaling pathway | 1/62 | 23.41 | 4.19E-02 | 2.08E-01 | IGFBP3 | 1 |
| BP | GO:0051657 | maintenance of organelle location | 1/62 | 23.41 | 4.19E-02 | 2.08E-01 | ALB | 1 |
| BP | GO:0051764 | actin crosslink formation | 1/62 | 23.41 | 4.19E-02 | 2.08E-01 | LCP1 | 1 |
| BP | GO:0061052 | negative regulation of cell growth involved in cardiac muscle cell development | 1/62 | 23.41 | 4.19E-02 | 2.08E-01 | PI16 | 1 |
| BP | GO:0070486 | leukocyte aggregation | 1/62 | 23.41 | 4.19E-02 | 2.08E-01 | MSN | 1 |
| BP | GO:1900103 | positive regulation of endoplasmic reticulum unfolded protein response | 1/62 | 23.41 | 4.19E-02 | 2.08E-01 | ERN1 | 1 |
| BP | GO:1905666 | regulation of protein localization to endosome | 1/62 | 23.41 | 4.19E-02 | 2.08E-01 | MSN | 1 |
| BP | GO:0003300 | cardiac muscle hypertrophy | 2/62 | 6.15 | 4.20E-02 | 2.08E-01 | TTN/PI16 | 2 |
| BP | GO:0030101 | natural killer cell activation | 2/62 | 6.15 | 4.20E-02 | 2.08E-01 | FCGR3A/PGLYRP2 | 2 |
| BP | GO:0046620 | regulation of organ growth | 2/62 | 6.15 | 4.20E-02 | 2.08E-01 | PI16/RBP4 | 2 |
| BP | GO:0045861 | negative regulation of proteolysis | 3/62 | 3.89 | 4.22E-02 | 2.08E-01 | FETUB/SERPINA5/SERPINA4 | 3 |
| BP | GO:0000041 | transition metal ion transport | 2/62 | 6.09 | 4.28E-02 | 2.08E-01 | CP/TF | 2 |
| BP | GO:0019217 | regulation of fatty acid metabolic process | 2/62 | 6.09 | 4.28E-02 | 2.08E-01 | APOC3/APOA4 | 2 |
| BP | GO:0034446 | substrate adhesion-dependent cell spreading | 2/62 | 6.09 | 4.28E-02 | 2.08E-01 | FGB/FN1 | 2 |
| BP | GO:0042632 | cholesterol homeostasis | 2/62 | 6.09 | 4.28E-02 | 2.08E-01 | APOC3/APOA4 | 2 |
| BP | GO:0042542 | response to hydrogen peroxide | 2/62 | 6.03 | 4.36E-02 | 2.09E-01 | HP/ERN1 | 2 |
| BP | GO:0055092 | sterol homeostasis | 2/62 | 6.03 | 4.36E-02 | 2.09E-01 | APOC3/APOA4 | 2 |
| BP | GO:0014897 | striated muscle hypertrophy | 2/62 | 5.97 | 4.44E-02 | 2.09E-01 | TTN/PI16 | 2 |
| BP | GO:0150115 | cell-substrate junction organization | 2/62 | 5.97 | 4.44E-02 | 2.09E-01 | APOD/FN1 | 2 |
| BP | GO:0002274 | myeloid leukocyte activation | 3/62 | 3.79 | 4.49E-02 | 2.09E-01 | FCGR3A/LBP/FN1 | 3 |
| BP | GO:0000394 | RNA splicing, via endonucleolytic cleavage and ligation | 1/62 | 21.74 | 4.50E-02 | 2.09E-01 | ERN1 | 1 |
| BP | GO:0002524 | hypersensitivity | 1/62 | 21.74 | 4.50E-02 | 2.09E-01 | FCGR3A | 1 |
| BP | GO:0010745 | negative regulation of macrophage derived foam cell differentiation | 1/62 | 21.74 | 4.50E-02 | 2.09E-01 | CRP | 1 |
| BP | GO:0034116 | positive regulation of heterotypic cell-cell adhesion | 1/62 | 21.74 | 4.50E-02 | 2.09E-01 | FGB | 1 |
| BP | GO:0015914 | phospholipid transport | 2/62 | 5.91 | 4.51E-02 | 2.09E-01 | APOC3/APOA4 | 2 |
| BP | GO:0022600 | digestive system process | 2/62 | 5.91 | 4.51E-02 | 2.09E-01 | APOA4/RBP4 | 2 |
| BP | GO:0032677 | regulation of interleukin-8 production | 2/62 | 5.91 | 4.51E-02 | 2.09E-01 | CRP/LBP | 2 |
| BP | GO:0014896 | muscle hypertrophy | 2/62 | 5.85 | 4.59E-02 | 2.09E-01 | TTN/PI16 | 2 |
| BP | GO:0032637 | interleukin-8 production | 2/62 | 5.85 | 4.59E-02 | 2.09E-01 | CRP/LBP | 2 |
| BP | GO:0032642 | regulation of chemokine production | 2/62 | 5.85 | 4.59E-02 | 2.09E-01 | LBP/APOD | 2 |
| BP | GO:0016485 | protein processing | 3/62 | 3.73 | 4.67E-02 | 2.09E-01 | FGB/F12/CLEC3B | 3 |
| BP | GO:0032602 | chemokine production | 2/62 | 5.80 | 4.67E-02 | 2.09E-01 | LBP/APOD | 2 |
| BP | GO:0002532 | production of molecular mediator involved in inflammatory response | 2/62 | 5.74 | 4.75E-02 | 2.09E-01 | LBP/APOD | 2 |
| BP | GO:0048738 | cardiac muscle tissue development | 3/62 | 3.70 | 4.77E-02 | 2.09E-01 | TTN/PI16/RBP4 | 3 |
| BP | GO:0001771 | immunological synapse formation | 1/62 | 20.29 | 4.82E-02 | 2.09E-01 | MSN | 1 |
| BP | GO:0001787 | natural killer cell proliferation | 1/62 | 20.29 | 4.82E-02 | 2.09E-01 | FCGR3A | 1 |
| BP | GO:0010889 | regulation of sequestering of triglyceride | 1/62 | 20.29 | 4.82E-02 | 2.09E-01 | APOC4 | 1 |
| BP | GO:0010989 | negative regulation of low-density lipoprotein particle clearance | 1/62 | 20.29 | 4.82E-02 | 2.09E-01 | APOC3 | 1 |
| BP | GO:0015670 | carbon dioxide transport | 1/62 | 20.29 | 4.82E-02 | 2.09E-01 | HBD | 1 |
| BP | GO:0015671 | oxygen transport | 1/62 | 20.29 | 4.82E-02 | 2.09E-01 | HBD | 1 |
| BP | GO:0042308 | negative regulation of protein import into nucleus | 1/62 | 20.29 | 4.82E-02 | 2.09E-01 | APOD | 1 |
| BP | GO:0045176 | apical protein localization | 1/62 | 20.29 | 4.82E-02 | 2.09E-01 | ACTB | 1 |
| BP | GO:0060347 | heart trabecula formation | 1/62 | 20.29 | 4.82E-02 | 2.09E-01 | RBP4 | 1 |
| BP | GO:0090209 | negative regulation of triglyceride metabolic process | 1/62 | 20.29 | 4.82E-02 | 2.09E-01 | APOC3 | 1 |
| BP | GO:1900029 | positive regulation of ruffle assembly | 1/62 | 20.29 | 4.82E-02 | 2.09E-01 | PFN1 | 1 |
| BP | GO:2000402 | negative regulation of lymphocyte migration | 1/62 | 20.29 | 4.82E-02 | 2.09E-01 | APOD | 1 |
| BP | GO:0006766 | vitamin metabolic process | 2/62 | 5.58 | 5.00E-02 | 2.11E-01 | BTD/GC | 2 |
| BP | GO:0090277 | positive regulation of peptide hormone secretion | 2/62 | 5.58 | 5.00E-02 | 2.11E-01 | FGB/RBP4 | 2 |
| CC | GO:0072562 | blood microparticle | 22/64 | 47.47 | 9.64E-32 | 1.54E-29 | HP/ORM2/JCHAIN/ORM1/C4A/C9/C4B/CP/FGB/APOL1/F13A1/ALB/ACTB/APOA4/GC/MSN/IGHG4/PON1/HBD/PFN1/FN1/TF | 22 |
| CC | GO:0034774 | secretory granule lumen | 13/64 | 12.54 | 2.59E-11 | 1.21E-09 | HP/LYZ/ORM2/ITIH3/ORM1/FGB/F13A1/SERPINA4/SPP2/ALB/CLEC3B/FN1/TF | 13 |
| CC | GO:0060205 | cytoplasmic vesicle lumen | 13/64 | 12.43 | 2.90E-11 | 1.21E-09 | HP/LYZ/ORM2/ITIH3/ORM1/FGB/F13A1/SERPINA4/SPP2/ALB/CLEC3B/FN1/TF | 13 |
| CC | GO:0031983 | vesicle lumen | 13/64 | 12.39 | 3.02E-11 | 1.21E-09 | HP/LYZ/ORM2/ITIH3/ORM1/FGB/F13A1/SERPINA4/SPP2/ALB/CLEC3B/FN1/TF | 13 |
| CC | GO:0062023 | collagen-containing extracellular matrix | 13/64 | 9.42 | 8.85E-10 | 2.83E-08 | ORM2/ORM1/HSP90B1/FGB/SERPINA5/F13A1/APOC3/F12/SPP2/APOA4/CLEC3B/TNXB/FN1 | 13 |
| CC | GO:0031091 | platelet alpha granule | 7/64 | 23.90 | 1.67E-08 | 4.20E-07 | ORM2/ORM1/FGB/SERPINA5/F13A1/ALB/FN1 | 7 |
| CC | GO:0034364 | high-density lipoprotein particle | 5/64 | 59.75 | 1.84E-08 | 4.20E-07 | APOL1/APOC3/APOA4/PON1/APOC4 | 5 |
| CC | GO:0005788 | endoplasmic reticulum lumen | 10/64 | 9.93 | 5.78E-08 | 1.16E-06 | C4A/HSP90B1/CP/APOL1/SPP2/ALB/APOA4/IGFBP3/FN1/TF | 10 |
| CC | GO:0031093 | platelet alpha granule lumen | 6/64 | 27.83 | 7.48E-08 | 1.33E-06 | ORM2/ORM1/FGB/F13A1/ALB/FN1 | 6 |
| CC | GO:0031089 | platelet dense granule lumen | 4/64 | 88.78 | 9.53E-08 | 1.37E-06 | ITIH3/SERPINA4/SPP2/CLEC3B | 4 |
| CC | GO:0034358 | plasma lipoprotein particle | 5/64 | 43.16 | 1.03E-07 | 1.37E-06 | APOL1/APOC3/APOA4/PON1/APOC4 | 5 |
| CC | GO:1990777 | lipoprotein particle | 5/64 | 43.16 | 1.03E-07 | 1.37E-06 | APOL1/APOC3/APOA4/PON1/APOC4 | 5 |
| CC | GO:0032994 | protein-lipid complex | 5/64 | 39.84 | 1.56E-07 | 1.92E-06 | APOL1/APOC3/APOA4/PON1/APOC4 | 5 |
| CC | GO:0034361 | very-low-density lipoprotein particle | 4/64 | 62.14 | 4.55E-07 | 4.85E-06 | APOL1/APOC3/APOA4/APOC4 | 4 |
| CC | GO:0034385 | triglyceride-rich plasma lipoprotein particle | 4/64 | 62.14 | 4.55E-07 | 4.85E-06 | APOL1/APOC3/APOA4/APOC4 | 4 |
| CC | GO:0042827 | platelet dense granule | 4/64 | 59.18 | 5.60E-07 | 5.60E-06 | ITIH3/SERPINA4/SPP2/CLEC3B | 4 |
| CC | GO:0035580 | specific granule lumen | 4/64 | 20.05 | 4.73E-05 | 4.45E-04 | HP/LYZ/ORM2/ORM1 | 4 |
| CC | GO:0019814 | immunoglobulin complex | 5/64 | 9.42 | 1.90E-04 | 1.69E-03 | JCHAIN/IGKV3D-7/IGLV5-45/IGHG4/IGHV3-20 | 5 |
| CC | GO:0031838 | haptoglobin-hemoglobin complex | 2/64 | 56.49 | 5.50E-04 | 4.64E-03 | HP/HBD | 2 |
| CC | GO:0042571 | immunoglobulin complex, circulating | 2/64 | 51.79 | 6.59E-04 | 5.02E-03 | JCHAIN/IGHG4 | 2 |
| CC | GO:0042627 | chylomicron | 2/64 | 51.79 | 6.59E-04 | 5.02E-03 | APOC3/APOA4 | 2 |
| CC | GO:1904724 | tertiary granule lumen | 3/64 | 16.95 | 7.40E-04 | 5.38E-03 | HP/LYZ/ORM1 | 3 |
| CC | GO:0042581 | specific granule | 4/64 | 7.77 | 1.76E-03 | 1.23E-02 | HP/LYZ/ORM2/ORM1 | 4 |
| CC | GO:0071682 | endocytic vesicle lumen | 2/64 | 27.02 | 2.47E-03 | 1.65E-02 | HP/HSP90B1 | 2 |
| CC | GO:0035267 | NuA4 histone acetyltransferase complex | 2/64 | 20.05 | 4.46E-03 | 2.75E-02 | ACTB/ACTBL2 | 2 |
| CC | GO:0043189 | H4/H2A histone acetyltransferase complex | 2/64 | 20.05 | 4.46E-03 | 2.75E-02 | ACTB/ACTBL2 | 2 |
| CC | GO:0005884 | actin filament | 3/64 | 7.90 | 6.52E-03 | 3.87E-02 | LCP1/ACTB/ACTBL2 | 3 |
| CC | GO:1902562 | H4 histone acetyltransferase complex | 2/64 | 13.81 | 9.23E-03 | 5.28E-02 | ACTB/ACTBL2 | 2 |
| CC | GO:0005925 | focal adhesion | 5/64 | 3.69 | 1.14E-02 | 6.29E-02 | HSP90B1/LCP1/ACTB/MSN/PFN1 | 5 |
| CC | GO:0030055 | cell-substrate junction | 5/64 | 3.60 | 1.25E-02 | 6.67E-02 | HSP90B1/LCP1/ACTB/MSN/PFN1 | 5 |
| CC | GO:0070820 | tertiary granule | 3/64 | 5.68 | 1.59E-02 | 8.20E-02 | HP/LYZ/ORM1 | 3 |
| CC | GO:0005775 | vacuolar lumen | 3/64 | 5.30 | 1.91E-02 | 9.57E-02 | LYZ/ORM2/GC | 3 |
| CC | GO:0030139 | endocytic vesicle | 4/64 | 3.57 | 2.58E-02 | 1.25E-01 | HP/CD163/HSP90B1/TF | 4 |
| CC | GO:0071745 | IgA immunoglobulin complex | 1/64 | 31.07 | 3.17E-02 | 1.39E-01 | JCHAIN | 1 |
| CC | GO:0099571 | postsynaptic cytoskeleton | 1/64 | 31.07 | 3.17E-02 | 1.39E-01 | ACTB | 1 |
| CC | GO:0140092 | bBAF complex | 1/64 | 31.07 | 3.17E-02 | 1.39E-01 | ACTB | 1 |
| CC | GO:0035578 | azurophil granule lumen | 2/64 | 6.83 | 3.48E-02 | 1.39E-01 | LYZ/ORM2 | 2 |
| CC | GO:0031094 | platelet dense tubular network | 1/64 | 28.25 | 3.48E-02 | 1.39E-01 | SERPINA5 | 1 |
| CC | GO:0034663 | endoplasmic reticulum chaperone complex | 1/64 | 28.25 | 3.48E-02 | 1.39E-01 | HSP90B1 | 1 |
| CC | GO:1904090 | peptidase inhibitor complex | 1/64 | 28.25 | 3.48E-02 | 1.39E-01 | SERPINA5 | 1 |
| CC | GO:0009897 | external side of plasma membrane | 4/64 | 3.21 | 3.60E-02 | 1.39E-01 | FCGR3A/CD163/FGB/SERPINA5 | 4 |
| CC | GO:0000123 | histone acetyltransferase complex | 2/64 | 6.61 | 3.69E-02 | 1.39E-01 | ACTB/ACTBL2 | 2 |
| CC | GO:0005833 | hemoglobin complex | 1/64 | 25.89 | 3.80E-02 | 1.39E-01 | HBD | 1 |
| CC | GO:0001931 | uropod | 1/64 | 23.90 | 4.11E-02 | 1.39E-01 | MSN | 1 |
| CC | GO:0071735 | IgG immunoglobulin complex | 1/64 | 23.90 | 4.11E-02 | 1.39E-01 | IGHG4 | 1 |
| CC | GO:0031254 | cell trailing edge | 1/64 | 22.19 | 4.41E-02 | 1.39E-01 | MSN | 1 |
| CC | GO:0035060 | brahma complex | 1/64 | 22.19 | 4.41E-02 | 1.39E-01 | ACTB | 1 |
| CC | GO:0071564 | npBAF complex | 1/64 | 22.19 | 4.41E-02 | 1.39E-01 | ACTB | 1 |
| CC | GO:0097524 | sperm plasma membrane | 1/64 | 22.19 | 4.41E-02 | 1.39E-01 | HSP90B1 | 1 |
| CC | GO:0140288 | GBAF complex | 1/64 | 22.19 | 4.41E-02 | 1.39E-01 | ACTB | 1 |
| CC | GO:0031248 | protein acetyltransferase complex | 2/64 | 5.98 | 4.43E-02 | 1.39E-01 | ACTB/ACTBL2 | 2 |
| CC | GO:1902493 | acetyltransferase complex | 2/64 | 5.92 | 4.50E-02 | 1.39E-01 | ACTB/ACTBL2 | 2 |
| CC | GO:0016586 | RSC-type complex | 1/64 | 20.71 | 4.72E-02 | 1.40E-01 | ACTB | 1 |
| CC | GO:0030175 | filopodium | 2/64 | 5.75 | 4.74E-02 | 1.40E-01 | LCP1/MSN | 2 |
| MF | GO:0061134 | peptidase regulator activity | 9/61 | 11.97 | 5.58E-08 | 9.88E-06 | FETUB/ITIH3/C4A/C4B/SERPINA5/SERPINA4/SPP2/PI16/FN1 | 9 |
| MF | GO:0030414 | peptidase inhibitor activity | 8/61 | 13.86 | 1.05E-07 | 9.88E-06 | FETUB/ITIH3/C4A/C4B/SERPINA5/SERPINA4/SPP2/PI16 | 8 |
| MF | GO:0004866 | endopeptidase inhibitor activity | 7/61 | 12.63 | 1.30E-06 | 7.12E-05 | FETUB/ITIH3/C4A/C4B/SERPINA5/SERPINA4/SPP2 | 7 |
| MF | GO:0001848 | complement binding | 4/61 | 46.65 | 1.52E-06 | 7.12E-05 | CRP/CFHR4/C4A/C4B | 4 |
| MF | GO:0061135 | endopeptidase regulator activity | 7/61 | 11.54 | 2.38E-06 | 8.97E-05 | FETUB/ITIH3/C4A/C4B/SERPINA5/SERPINA4/SPP2 | 7 |
| MF | GO:0004857 | enzyme inhibitor activity | 9/61 | 7.07 | 4.55E-06 | 1.43E-04 | FETUB/ITIH3/C4A/C4B/SERPINA5/APOC3/SERPINA4/SPP2/PI16 | 9 |
| MF | GO:0016209 | antioxidant activity | 5/61 | 18.27 | 7.87E-06 | 2.11E-04 | HP/ALB/APOA4/GPX3/HBD | 5 |
| MF | GO:0005539 | glycosaminoglycan binding | 7/61 | 8.84 | 1.36E-05 | 3.20E-04 | JCHAIN/HABP2/SERPINA5/PGLYRP2/CLEC3B/TNXB/FN1 | 7 |
| MF | GO:0050997 | quaternary ammonium group binding | 4/61 | 22.46 | 2.99E-05 | 6.25E-04 | CRP/JCHAIN/SERPINA5/APOA4 | 4 |
| MF | GO:0001846 | opsonin binding | 3/61 | 43.32 | 4.35E-05 | 8.18E-04 | CRP/CFHR4/C4A | 3 |
| MF | GO:0070325 | lipoprotein particle receptor binding | 3/61 | 31.37 | 1.17E-04 | 2.01E-03 | CRP/HSP90B1/APOC3 | 3 |
| MF | GO:0031210 | phosphatidylcholine binding | 3/61 | 30.32 | 1.30E-04 | 2.04E-03 | JCHAIN/SERPINA5/APOA4 | 3 |
| MF | GO:0005319 | lipid transporter activity | 5/61 | 8.81 | 2.56E-04 | 3.71E-03 | CFHR4/APOD/APOA4/RBP4/APOC4 | 5 |
| MF | GO:0098973 | structural constituent of postsynaptic actin cytoskeleton | 2/61 | 60.64 | 4.73E-04 | 6.36E-03 | ACTB/ACTBL2 | 2 |
| MF | GO:0031994 | insulin-like growth factor I binding | 2/61 | 46.65 | 8.15E-04 | 1.02E-02 | IGFBP6/IGFBP3 | 2 |
| MF | GO:0061783 | peptidoglycan muralytic activity | 2/61 | 43.32 | 9.49E-04 | 1.07E-02 | LYZ/PGLYRP2 | 2 |
| MF | GO:0051087 | protein-folding chaperone binding | 4/61 | 9.12 | 9.71E-04 | 1.07E-02 | ERN1/CP/FGB/ALB | 4 |
| MF | GO:0005507 | copper ion binding | 3/61 | 14.44 | 1.18E-03 | 1.17E-02 | CP/ALB/APOA4 | 3 |
| MF | GO:0002020 | protease binding | 4/61 | 8.54 | 1.24E-03 | 1.17E-02 | TTN/SERPINA5/CHL1/FN1 | 4 |
| MF | GO:0034987 | immunoglobulin receptor binding | 2/61 | 37.90 | 1.25E-03 | 1.17E-02 | JCHAIN/IGHG4 | 2 |
| MF | GO:0005520 | insulin-like growth factor binding | 2/61 | 31.92 | 1.76E-03 | 1.51E-02 | IGFBP6/IGFBP3 | 2 |
| MF | GO:0042834 | peptidoglycan binding | 2/61 | 31.92 | 1.76E-03 | 1.51E-02 | JCHAIN/PGLYRP2 | 2 |
| MF | GO:1901681 | sulfur compound binding | 5/61 | 5.51 | 2.11E-03 | 1.69E-02 | SERPINA5/CLEC3B/TNXB/FN1/TF | 5 |
| MF | GO:0099186 | structural constituent of postsynapse | 2/61 | 28.88 | 2.16E-03 | 1.69E-02 | ACTB/ACTBL2 | 2 |
| MF | GO:0004252 | serine-type endopeptidase activity | 4/61 | 6.97 | 2.60E-03 | 1.88E-02 | HP/HABP2/F12/C2 | 4 |
| MF | GO:0008201 | heparin binding | 4/61 | 6.97 | 2.60E-03 | 1.88E-02 | SERPINA5/CLEC3B/TNXB/FN1 | 4 |
| MF | GO:0019865 | immunoglobulin binding | 2/61 | 25.27 | 2.82E-03 | 1.89E-02 | FCGR3A/JCHAIN | 2 |
| MF | GO:0050750 | low-density lipoprotein particle receptor binding | 2/61 | 25.27 | 2.82E-03 | 1.89E-02 | CRP/HSP90B1 | 2 |
| MF | GO:0043178 | alcohol binding | 3/61 | 10.22 | 3.17E-03 | 2.05E-02 | APOC3/APOD/RBP4 | 3 |
| MF | GO:0098918 | structural constituent of synapse | 2/61 | 22.46 | 3.56E-03 | 2.23E-02 | ACTB/ACTBL2 | 2 |
| MF | GO:0008236 | serine-type peptidase activity | 4/61 | 6.32 | 3.70E-03 | 2.25E-02 | HP/HABP2/F12/C2 | 4 |
| MF | GO:0017171 | serine hydrolase activity | 4/61 | 6.19 | 3.98E-03 | 2.34E-02 | HP/HABP2/F12/C2 | 4 |
| MF | GO:0001968 | fibronectin binding | 2/61 | 20.91 | 4.10E-03 | 2.34E-02 | IGFBP6/IGFBP3 | 2 |
| MF | GO:0004867 | serine-type endopeptidase inhibitor activity | 3/61 | 9.01 | 4.52E-03 | 2.50E-02 | ITIH3/SERPINA5/SERPINA4 | 3 |
| MF | GO:0005496 | steroid binding | 3/61 | 8.92 | 4.65E-03 | 2.50E-02 | APOC3/APOD/GC | 3 |
| MF | GO:0005543 | phospholipid binding | 6/61 | 3.76 | 5.12E-03 | 2.68E-02 | JCHAIN/SERPINA5/APOC3/APOA4/PON1/PFN1 | 6 |
| MF | GO:0090482 | vitamin transmembrane transporter activity | 2/61 | 17.33 | 5.94E-03 | 2.98E-02 | RBP4/GC | 2 |
| MF | GO:0005200 | structural constituent of cytoskeleton | 3/61 | 8.12 | 6.03E-03 | 2.98E-02 | ACTB/MSN/ACTBL2 | 3 |
| MF | GO:0005501 | retinoid binding | 2/61 | 15.96 | 6.97E-03 | 3.28E-02 | SERPINA5/RBP4 | 2 |
| MF | GO:0019840 | isoprenoid binding | 2/61 | 15.96 | 6.97E-03 | 3.28E-02 | SERPINA5/RBP4 | 2 |
| MF | GO:0019825 | oxygen binding | 2/61 | 15.55 | 7.33E-03 | 3.36E-02 | ALB/HBD | 2 |
| MF | GO:0016810 | hydrolase activity, acting on carbon-nitrogen (but not peptide) bonds | 3/61 | 6.69 | 1.03E-02 | 4.59E-02 | CD101/BTD/PGLYRP2 | 3 |
| MF | GO:0015485 | cholesterol binding | 2/61 | 11.66 | 1.28E-02 | 5.58E-02 | APOC3/APOD | 2 |
| MF | GO:0019842 | vitamin binding | 3/61 | 6.06 | 1.34E-02 | 5.70E-02 | ALB/RBP4/GC | 3 |
| MF | GO:0004601 | peroxidase activity | 2/61 | 11.03 | 1.42E-02 | 5.85E-02 | GPX3/HBD | 2 |
| MF | GO:0005178 | integrin binding | 3/61 | 5.91 | 1.43E-02 | 5.85E-02 | LCP1/TNXB/FN1 | 3 |
| MF | GO:0003779 | actin binding | 5/61 | 3.46 | 1.46E-02 | 5.85E-02 | TTN/LCP1/GC/MSN/PFN1 | 5 |
| MF | GO:0016684 | oxidoreductase activity, acting on peroxide as acceptor | 2/61 | 10.64 | 1.52E-02 | 5.95E-02 | GPX3/HBD | 2 |
| MF | GO:0005201 | extracellular matrix structural constituent | 3/61 | 5.48 | 1.75E-02 | 6.70E-02 | FGB/TNXB/FN1 | 3 |
| MF | GO:0032934 | sterol binding | 2/61 | 9.78 | 1.78E-02 | 6.70E-02 | APOC3/APOD | 2 |
| MF | GO:0003823 | antigen binding | 3/61 | 5.11 | 2.10E-02 | 7.66E-02 | JCHAIN/IGHG4/IGHV3-20 | 3 |
| MF | GO:0005518 | collagen binding | 2/61 | 8.92 | 2.12E-02 | 7.66E-02 | TNXB/FN1 | 2 |
| MF | GO:0016811 | hydrolase activity, acting on carbon-nitrogen (but not peptide) bonds, in linear amides | 2/61 | 8.54 | 2.30E-02 | 8.15E-02 | BTD/PGLYRP2 | 2 |
| MF | GO:0033293 | monocarboxylic acid binding | 2/61 | 7.68 | 2.80E-02 | 9.76E-02 | SERPINA5/ALB | 2 |
| MF | GO:0043177 | organic acid binding | 3/61 | 4.39 | 3.09E-02 | 1.02E-01 | SERPINA5/ALB/HBD | 3 |
| MF | GO:0003796 | lysozyme activity | 1/61 | 30.32 | 3.25E-02 | 1.02E-01 | LYZ | 1 |
| MF | GO:0015643 | toxic substance binding | 1/61 | 30.32 | 3.25E-02 | 1.02E-01 | ALB | 1 |
| MF | GO:0030957 | Tat protein binding | 1/61 | 30.32 | 3.25E-02 | 1.02E-01 | ACTB | 1 |
| MF | GO:0031720 | haptoglobin binding | 1/61 | 30.32 | 3.25E-02 | 1.02E-01 | HBD | 1 |
| MF | GO:0071723 | lipopeptide binding | 1/61 | 30.32 | 3.25E-02 | 1.02E-01 | LBP | 1 |
| MF | GO:0001851 | complement component C3b binding | 1/61 | 27.56 | 3.57E-02 | 1.03E-01 | CFHR4 | 1 |
| MF | GO:0008199 | ferric iron binding | 1/61 | 27.56 | 3.57E-02 | 1.03E-01 | TF | 1 |
| MF | GO:0019864 | IgG binding | 1/61 | 27.56 | 3.57E-02 | 1.03E-01 | FCGR3A | 1 |
| MF | GO:0050998 | nitric-oxide synthase binding | 1/61 | 27.56 | 3.57E-02 | 1.03E-01 | ACTB | 1 |
| MF | GO:1990459 | transferrin receptor binding | 1/61 | 27.56 | 3.57E-02 | 1.03E-01 | TF | 1 |
| MF | GO:0005527 | macrolide binding | 1/61 | 25.27 | 3.89E-02 | 1.07E-01 | ALB | 1 |
| MF | GO:0097493 | structural molecule activity conferring elasticity | 1/61 | 25.27 | 3.89E-02 | 1.07E-01 | TTN | 1 |
| MF | GO:0140104 | molecular carrier activity | 2/61 | 6.38 | 3.93E-02 | 1.07E-01 | HBD/TF | 2 |
| MF | GO:0004175 | endopeptidase activity | 4/61 | 3.12 | 3.94E-02 | 1.07E-01 | HP/HABP2/F12/C2 | 4 |
| MF | GO:0005161 | platelet-derived growth factor receptor binding | 1/61 | 21.66 | 4.52E-02 | 1.18E-01 | ERN1 | 1 |
| MF | GO:0005344 | oxygen carrier activity | 1/61 | 21.66 | 4.52E-02 | 1.18E-01 | HBD | 1 |
| MF | GO:0016530 | metallochaperone activity | 1/61 | 21.66 | 4.52E-02 | 1.18E-01 | TF | 1 |
| MF | GO:0000774 | adenyl-nucleotide exchange factor activity | 1/61 | 20.21 | 4.84E-02 | 1.21E-01 | PFN1 | 1 |
| MF | GO:0016812 | hydrolase activity, acting on carbon-nitrogen (but not peptide) bonds, in cyclic amides | 1/61 | 20.21 | 4.84E-02 | 1.21E-01 | CD101 | 1 |
| MF | GO:0051371 | muscle alpha-actinin binding | 1/61 | 20.21 | 4.84E-02 | 1.21E-01 | TTN | 1 |
| Organismal Systems | hsa04610 | Complement and coagulation cascades | 9/31 | 29.18 | 1.06E-11 | 9.22E-10 | CFHR4/C4A/C9/C4B/FGB/SERPINA5/F13A1/F12/C2 | 9 |
| Human Diseases | hsa05322 | Systemic lupus erythematosus | 5/31 | 10.26 | 1.09E-04 | 4.32E-03 | FCGR3A/C4A/C9/C4B/C2 | 5 |
| Human Diseases | hsa05171 | Coronavirus disease - COVID-19 | 6/31 | 7.19 | 1.49E-04 | 4.32E-03 | C4A/C9/C4B/FGB/F13A1/C2 | 6 |
| Human Diseases | hsa05150 | Staphylococcus aureus infection | 4/31 | 11.41 | 3.83E-04 | 8.32E-03 | FCGR3A/C4A/C4B/C2 | 4 |
| Cellular Processes | hsa04810 | Regulation of actin cytoskeleton | 5/31 | 6.20 | 1.11E-03 | 1.94E-02 | C9/ACTB/MSN/PFN1/FN1 | 5 |
| Human Diseases | hsa04936 | Alcoholic liver disease | 4/31 | 7.93 | 1.51E-03 | 2.18E-02 | LBP/C4A/C4B/C2 | 4 |
| Organismal Systems | hsa04918 | Thyroid hormone synthesis | 3/31 | 11.41 | 2.22E-03 | 2.70E-02 | HSP90B1/ALB/GPX3 | 3 |
| Human Diseases | hsa05133 | Pertussis | 3/31 | 10.98 | 2.48E-03 | 2.70E-02 | C4A/C4B/C2 | 3 |
| Organismal Systems | hsa04977 | Vitamin digestion and absorption | 2/31 | 21.95 | 3.67E-03 | 3.54E-02 | BTD/APOA4 | 2 |
| Human Diseases | hsa05417 | Lipid and atherosclerosis | 4/31 | 5.28 | 6.49E-03 | 5.64E-02 | ERN1/LBP/HSP90B1/APOA4 | 4 |
| Organismal Systems | hsa04670 | Leukocyte transendothelial migration | 3/31 | 7.38 | 7.56E-03 | 5.98E-02 | CLDN10/ACTB/MSN | 3 |
| Cellular Processes | hsa04216 | Ferroptosis | 2/31 | 13.59 | 9.38E-03 | 6.80E-02 | CP/TF | 2 |
| Organismal Systems | hsa04979 | Cholesterol metabolism | 2/31 | 11.19 | 1.36E-02 | 9.12E-02 | APOC3/APOA4 | 2 |
| Cellular Processes | hsa04530 | Tight junction | 3/31 | 5.04 | 2.11E-02 | 1.31E-01 | CLDN10/ACTB/MSN | 3 |
| Organismal Systems | hsa04613 | Neutrophil extracellular trap formation | 3/31 | 4.46 | 2.90E-02 | 1.63E-01 | FCGR3A/FGB/ACTB | 3 |
| Human Diseases | hsa05100 | Bacterial invasion of epithelial cells | 2/31 | 7.32 | 3.03E-02 | 1.63E-01 | ACTB/FN1 | 2 |
| Cellular Processes | hsa04510 | Focal adhesion | 3/31 | 4.22 | 3.34E-02 | 1.63E-01 | ACTB/TNXB/FN1 | 3 |
| Human Diseases | hsa05205 | Proteoglycans in cancer | 3/31 | 4.20 | 3.38E-02 | 1.63E-01 | ACTB/MSN/FN1 | 3 |
| Environmental Information Processing | hsa04512 | ECM-receptor interaction | 2/31 | 6.41 | 3.85E-02 | 1.70E-01 | TNXB/FN1 | 2 |
| Human Diseases | hsa05014 | Amyotrophic lateral sclerosis | 4/31 | 3.08 | 3.92E-02 | 1.70E-01 | ERN1/ACTB/GPX3/PFN1 | 4 |
| NA | hsa04820 | Cytoskeleton in muscle cells | 3/31 | 3.69 | 4.67E-02 | 1.85E-01 | TTN/ACTB/FN1 | 3 |
| Human Diseases | hsa05410 | Hypertrophic cardiomyopathy | 2/31 | 5.76 | 4.67E-02 | 1.85E-01 | TTN/ACTB | 2 |

Table S12: Enrichment analysis results of DEGs in the cured stage of pulmonary tuberculosis

| Category | ID | Description | GeneRatio | FoldEnrichment | pvalue | p.adjust | geneID | Count |
| --- | --- | --- | --- | --- | --- | --- | --- | --- |
| BP | GO:0007596 | blood coagulation | 12/68 | 14.87 | 2.36E-11 | 1.76E-08 | F12/ACTB/F13A1/F10/F13B/PLG/HBB/APOE/HGFAC/SERPINA1/SERPINC1/VTN | 12 |
| BP | GO:0050817 | coagulation | 12/68 | 14.54 | 3.06E-11 | 1.76E-08 | F12/ACTB/F13A1/F10/F13B/PLG/HBB/APOE/HGFAC/SERPINA1/SERPINC1/VTN | 12 |
| BP | GO:0007599 | hemostasis | 12/68 | 14.42 | 3.39E-11 | 1.76E-08 | F12/ACTB/F13A1/F10/F13B/PLG/HBB/APOE/HGFAC/SERPINA1/SERPINC1/VTN | 12 |
| BP | GO:0042744 | hydrogen peroxide catabolic process | 6/68 | 53.71 | 1.20E-09 | 4.68E-07 | HBD/APOA4/GPX3/HBA2/HBB/HP | 6 |
| BP | GO:0098869 | cellular oxidant detoxification | 8/68 | 22.42 | 2.46E-09 | 7.68E-07 | HBD/ALB/APOA4/GPX3/HBA2/HBB/APOE/HP | 8 |
| BP | GO:0042060 | wound healing | 13/68 | 8.53 | 3.08E-09 | 8.02E-07 | FN1/F12/ACTB/F13A1/F10/F13B/PLG/HBB/APOE/HGFAC/SERPINA1/SERPINC1/VTN | 13 |
| BP | GO:0050878 | regulation of body fluid levels | 12/68 | 9.12 | 6.30E-09 | 1.41E-06 | F12/ACTB/F13A1/F10/F13B/PLG/HBB/APOE/HGFAC/SERPINA1/SERPINC1/VTN | 12 |
| BP | GO:1990748 | cellular detoxification | 8/68 | 18.66 | 1.06E-08 | 2.08E-06 | HBD/ALB/APOA4/GPX3/HBA2/HBB/APOE/HP | 8 |
| BP | GO:0097237 | cellular response to toxic substance | 8/68 | 17.34 | 1.89E-08 | 2.98E-06 | HBD/ALB/APOA4/GPX3/HBA2/HBB/APOE/HP | 8 |
| BP | GO:0006953 | acute-phase response | 6/68 | 34.69 | 1.90E-08 | 2.98E-06 | FN1/MBL2/SERPINA1/ORM1/HP/LBP | 6 |
| BP | GO:0042743 | hydrogen peroxide metabolic process | 6/68 | 30.83 | 3.94E-08 | 5.60E-06 | HBD/APOA4/GPX3/HBA2/HBB/HP | 6 |
| BP | GO:0034370 | triglyceride-rich lipoprotein particle remodeling | 4/68 | 92.50 | 7.47E-08 | 8.98E-06 | APOA4/APOE/APOC3/CETP | 4 |
| BP | GO:0034372 | very-low-density lipoprotein particle remodeling | 4/68 | 92.50 | 7.47E-08 | 8.98E-06 | APOA4/APOE/APOC3/CETP | 4 |
| BP | GO:0098754 | detoxification | 8/68 | 14.05 | 9.76E-08 | 1.06E-05 | HBD/ALB/APOA4/GPX3/HBA2/HBB/APOE/HP | 8 |
| BP | GO:0002526 | acute inflammatory response | 7/68 | 18.15 | 1.13E-07 | 1.06E-05 | FN1/F12/MBL2/SERPINA1/ORM1/HP/LBP | 7 |
| BP | GO:0034368 | protein-lipid complex remodeling | 5/68 | 42.05 | 1.15E-07 | 1.06E-05 | APOA4/AGT/APOE/APOC3/CETP | 5 |
| BP | GO:0034369 | plasma lipoprotein particle remodeling | 5/68 | 42.05 | 1.15E-07 | 1.06E-05 | APOA4/AGT/APOE/APOC3/CETP | 5 |
| BP | GO:0034367 | protein-containing complex remodeling | 5/68 | 39.64 | 1.56E-07 | 1.36E-05 | APOA4/AGT/APOE/APOC3/CETP | 5 |
| BP | GO:0034375 | high-density lipoprotein particle remodeling | 4/68 | 69.38 | 2.72E-07 | 2.23E-05 | APOA4/APOE/APOC3/CETP | 4 |
| BP | GO:0009636 | response to toxic substance | 9/68 | 9.68 | 3.54E-07 | 2.77E-05 | HBD/ALB/APOA4/GPX3/PON1/HBA2/HBB/APOE/HP | 9 |
| BP | GO:0006639 | acylglycerol metabolic process | 7/68 | 14.50 | 5.26E-07 | 3.91E-05 | APOA4/GPLD1/TNXB/APOE/APOC3/CETP/ANG | 7 |
| BP | GO:0006638 | neutral lipid metabolic process | 7/68 | 14.39 | 5.53E-07 | 3.93E-05 | APOA4/GPLD1/TNXB/APOE/APOC3/CETP/ANG | 7 |
| BP | GO:0043691 | reverse cholesterol transport | 4/68 | 55.50 | 7.15E-07 | 4.86E-05 | APOA4/APOE/APOC3/CETP | 4 |
| BP | GO:0097006 | regulation of plasma lipoprotein particle levels | 6/68 | 18.30 | 9.16E-07 | 5.97E-05 | APOA4/GPLD1/AGT/APOE/APOC3/CETP | 6 |
| BP | GO:0071827 | plasma lipoprotein particle organization | 5/68 | 27.75 | 9.78E-07 | 6.11E-05 | APOA4/AGT/APOE/APOC3/CETP | 5 |
| BP | GO:0071825 | protein-lipid complex organization | 5/68 | 26.18 | 1.31E-06 | 7.90E-05 | APOA4/AGT/APOE/APOC3/CETP | 5 |
| BP | GO:0006641 | triglyceride metabolic process | 6/68 | 15.71 | 2.25E-06 | 1.30E-04 | APOA4/GPLD1/TNXB/APOE/APOC3/CETP | 6 |
| BP | GO:0030193 | regulation of blood coagulation | 5/68 | 20.40 | 4.58E-06 | 2.53E-04 | F12/PLG/APOE/SERPINC1/VTN | 5 |
| BP | GO:0006869 | lipid transport | 10/68 | 6.13 | 4.82E-06 | 2.53E-04 | APOA4/APOD/SERPINA5/PON1/AGT/APOE/APOC3/CETP/RBP4/LBP | 10 |
| BP | GO:0051702 | biological process involved in interaction with symbiont | 6/68 | 13.76 | 4.86E-06 | 2.53E-04 | FN1/MBL2/PLG/APOE/CFHR1/CSF1R | 6 |
| BP | GO:1905918 | regulation of CoA-transferase activity | 3/68 | 83.25 | 5.27E-06 | 2.58E-04 | APOA4/AGT/APOE | 3 |
| BP | GO:1900046 | regulation of hemostasis | 5/68 | 19.82 | 5.28E-06 | 2.58E-04 | F12/PLG/APOE/SERPINC1/VTN | 5 |
| BP | GO:0033344 | cholesterol efflux | 5/68 | 19.01 | 6.50E-06 | 2.99E-04 | APOA4/PON1/APOE/APOC3/CETP | 5 |
| BP | GO:0050818 | regulation of coagulation | 5/68 | 19.01 | 6.50E-06 | 2.99E-04 | F12/PLG/APOE/SERPINC1/VTN | 5 |
| BP | GO:0001894 | tissue homeostasis | 8/68 | 7.96 | 7.09E-06 | 3.08E-04 | ALB/PIGR/ACTB/TF/CDH5/RBP4/JCHAIN/CSF1R | 8 |
| BP | GO:0060249 | anatomical structure homeostasis | 8/68 | 7.96 | 7.09E-06 | 3.08E-04 | ALB/PIGR/ACTB/TF/CDH5/RBP4/JCHAIN/CSF1R | 8 |
| BP | GO:0000302 | response to reactive oxygen species | 7/68 | 9.48 | 8.92E-06 | 3.77E-04 | APOA4/APOD/HBA2/HBB/APOE/HP/ERN1 | 7 |
| BP | GO:0032371 | regulation of sterol transport | 5/68 | 17.34 | 1.02E-05 | 4.09E-04 | APOA4/PON1/APOE/APOC3/CETP | 5 |
| BP | GO:0032374 | regulation of cholesterol transport | 5/68 | 17.34 | 1.02E-05 | 4.09E-04 | APOA4/PON1/APOE/APOC3/CETP | 5 |
| BP | GO:0001895 | retina homeostasis | 5/68 | 16.72 | 1.22E-05 | 4.76E-04 | ALB/PIGR/ACTB/TF/JCHAIN | 5 |
| BP | GO:0010896 | regulation of triglyceride catabolic process | 3/68 | 64.04 | 1.25E-05 | 4.76E-04 | APOA4/GPLD1/APOC3 | 3 |
| BP | GO:0006979 | response to oxidative stress | 9/68 | 6.24 | 1.29E-05 | 4.80E-04 | APOA4/APOD/MBL2/GPX3/HBA2/HBB/APOE/HP/ERN1 | 9 |
| BP | GO:0015850 | organic hydroxy compound transport | 8/68 | 7.21 | 1.45E-05 | 5.28E-04 | APOA4/ACTB/PON1/AGT/APOE/APOC3/CETP/RBP4 | 8 |
| BP | GO:0033700 | phospholipid efflux | 3/68 | 59.46 | 1.58E-05 | 5.63E-04 | APOA4/APOE/APOC3 | 3 |
| BP | GO:0055090 | acylglycerol homeostasis | 4/68 | 25.81 | 1.71E-05 | 5.82E-04 | APOA4/APOE/APOC3/CETP | 4 |
| BP | GO:0070328 | triglyceride homeostasis | 4/68 | 25.81 | 1.71E-05 | 5.82E-04 | APOA4/APOE/APOC3/CETP | 4 |
| BP | GO:0032368 | regulation of lipid transport | 6/68 | 10.88 | 1.86E-05 | 6.05E-04 | APOA4/PON1/AGT/APOE/APOC3/CETP | 6 |
| BP | GO:0090207 | regulation of triglyceride metabolic process | 4/68 | 25.23 | 1.88E-05 | 6.05E-04 | APOA4/GPLD1/APOE/APOC3 | 4 |
| BP | GO:0015670 | carbon dioxide transport | 3/68 | 55.50 | 1.97E-05 | 6.05E-04 | HBD/HBA2/HBB | 3 |
| BP | GO:0015671 | oxygen transport | 3/68 | 55.50 | 1.97E-05 | 6.05E-04 | HBD/HBA2/HBB | 3 |
| BP | GO:0090209 | negative regulation of triglyceride metabolic process | 3/68 | 55.50 | 1.97E-05 | 6.05E-04 | GPLD1/APOE/APOC3 | 3 |
| BP | GO:0072593 | reactive oxygen species metabolic process | 7/68 | 8.30 | 2.10E-05 | 6.32E-04 | HBD/APOA4/GPX3/HBA2/AGT/HBB/HP | 7 |
| BP | GO:0034374 | low-density lipoprotein particle remodeling | 3/68 | 52.03 | 2.42E-05 | 7.02E-04 | AGT/APOE/CETP | 3 |
| BP | GO:0072378 | blood coagulation, fibrin clot formation | 3/68 | 52.03 | 2.42E-05 | 7.02E-04 | F12/F13A1/F13B | 3 |
| BP | GO:0030195 | negative regulation of blood coagulation | 4/68 | 23.13 | 2.66E-05 | 7.57E-04 | F12/PLG/APOE/VTN | 4 |
| BP | GO:1900047 | negative regulation of hemostasis | 4/68 | 22.65 | 2.89E-05 | 8.07E-04 | F12/PLG/APOE/VTN | 4 |
| BP | GO:0034384 | high-density lipoprotein particle clearance | 3/68 | 46.25 | 3.51E-05 | 9.47E-04 | GPLD1/APOE/APOC3 | 3 |
| BP | GO:0051917 | regulation of fibrinolysis | 3/68 | 46.25 | 3.51E-05 | 9.47E-04 | F12/PLG/VTN | 3 |
| BP | GO:0050819 | negative regulation of coagulation | 4/68 | 21.35 | 3.66E-05 | 9.71E-04 | F12/PLG/APOE/VTN | 4 |
| BP | GO:0006959 | humoral immune response | 7/68 | 7.53 | 3.92E-05 | 1.02E-03 | TF/MBL2/CFHR1/MASP1/ANG/C9/JCHAIN | 7 |
| BP | GO:0072376 | protein activation cascade | 3/68 | 43.82 | 4.16E-05 | 1.07E-03 | F12/F13A1/F13B | 3 |
| BP | GO:1905952 | regulation of lipid localization | 6/68 | 9.10 | 5.09E-05 | 1.28E-03 | APOA4/PON1/AGT/APOE/APOC3/CETP | 6 |
| BP | GO:0043086 | negative regulation of catalytic activity | 9/68 | 5.20 | 5.38E-05 | 1.34E-03 | FETUB/SERPINA5/SERPINA4/AGT/APOE/APOC3/SERPINA1/VTN/HP | 9 |
| BP | GO:0031638 | zymogen activation | 4/68 | 18.81 | 6.05E-05 | 1.48E-03 | F12/CLEC3B/MASP1/HGFAC | 4 |
| BP | GO:0015669 | gas transport | 3/68 | 37.84 | 6.56E-05 | 1.58E-03 | HBD/HBA2/HBB | 3 |
| BP | GO:0046486 | glycerolipid metabolic process | 8/68 | 5.69 | 7.76E-05 | 1.84E-03 | APOA4/GPLD1/PON1/TNXB/APOE/APOC3/CETP/ANG | 8 |
| BP | GO:0010951 | negative regulation of endopeptidase activity | 5/68 | 10.84 | 9.83E-05 | 2.26E-03 | FETUB/SERPINA5/SERPINA4/SERPINA1/VTN | 5 |
| BP | GO:0030301 | cholesterol transport | 5/68 | 10.84 | 9.83E-05 | 2.26E-03 | APOA4/PON1/APOE/APOC3/CETP | 5 |
| BP | GO:0006956 | complement activation | 4/68 | 16.57 | 9.96E-05 | 2.26E-03 | MBL2/CFHR1/MASP1/C9 | 4 |
| BP | GO:0061041 | regulation of wound healing | 5/68 | 10.43 | 1.18E-04 | 2.60E-03 | F12/PLG/APOE/SERPINC1/VTN | 5 |
| BP | GO:0048662 | negative regulation of smooth muscle cell proliferation | 4/68 | 15.86 | 1.18E-04 | 2.60E-03 | APOD/IGFBP3/APOE/ANG | 4 |
| BP | GO:0034377 | plasma lipoprotein particle assembly | 3/68 | 30.83 | 1.23E-04 | 2.63E-03 | APOA4/APOE/APOC3 | 3 |
| BP | GO:0042730 | fibrinolysis | 3/68 | 30.83 | 1.23E-04 | 2.63E-03 | F12/PLG/VTN | 3 |
| BP | GO:0051346 | negative regulation of hydrolase activity | 6/68 | 7.64 | 1.34E-04 | 2.75E-03 | FETUB/SERPINA5/SERPINA4/APOC3/SERPINA1/VTN | 6 |
| BP | GO:0010466 | negative regulation of peptidase activity | 5/68 | 10.13 | 1.35E-04 | 2.75E-03 | FETUB/SERPINA5/SERPINA4/SERPINA1/VTN | 5 |
| BP | GO:0019433 | triglyceride catabolic process | 3/68 | 29.73 | 1.37E-04 | 2.75E-03 | APOA4/GPLD1/APOC3 | 3 |
| BP | GO:0030194 | positive regulation of blood coagulation | 3/68 | 29.73 | 1.37E-04 | 2.75E-03 | F12/PLG/VTN | 3 |
| BP | GO:1900048 | positive regulation of hemostasis | 3/68 | 29.73 | 1.37E-04 | 2.75E-03 | F12/PLG/VTN | 3 |
| BP | GO:0061045 | negative regulation of wound healing | 4/68 | 14.80 | 1.55E-04 | 3.03E-03 | F12/PLG/APOE/VTN | 4 |
| BP | GO:0015918 | sterol transport | 5/68 | 9.84 | 1.55E-04 | 3.03E-03 | APOA4/PON1/APOE/APOC3/CETP | 5 |
| BP | GO:0046470 | phosphatidylcholine metabolic process | 4/68 | 14.61 | 1.63E-04 | 3.14E-03 | APOA4/GPLD1/PON1/CETP | 4 |
| BP | GO:0050820 | positive regulation of coagulation | 3/68 | 27.75 | 1.69E-04 | 3.19E-03 | F12/PLG/VTN | 3 |
| BP | GO:0065005 | protein-lipid complex assembly | 3/68 | 27.75 | 1.69E-04 | 3.19E-03 | APOA4/APOE/APOC3 | 3 |
| BP | GO:0019318 | hexose metabolic process | 6/68 | 7.15 | 1.92E-04 | 3.57E-03 | GALE/APOD/IGFBP3/GPLD1/MAN2A1/RBP4 | 6 |
| BP | GO:0033002 | muscle cell proliferation | 6/68 | 6.74 | 2.63E-04 | 4.83E-03 | APOD/IGFBP3/APOE/RBP4/ANG/ERN1 | 6 |
| BP | GO:0032373 | positive regulation of sterol transport | 3/68 | 21.91 | 3.45E-04 | 6.06E-03 | PON1/APOE/CETP | 3 |
| BP | GO:0032376 | positive regulation of cholesterol transport | 3/68 | 21.91 | 3.45E-04 | 6.06E-03 | PON1/APOE/CETP | 3 |
| BP | GO:0046461 | neutral lipid catabolic process | 3/68 | 21.91 | 3.45E-04 | 6.06E-03 | APOA4/GPLD1/APOC3 | 3 |
| BP | GO:0046464 | acylglycerol catabolic process | 3/68 | 21.91 | 3.45E-04 | 6.06E-03 | APOA4/GPLD1/APOC3 | 3 |
| BP | GO:0005996 | monosaccharide metabolic process | 6/68 | 6.38 | 3.53E-04 | 6.13E-03 | GALE/APOD/IGFBP3/GPLD1/MAN2A1/RBP4 | 6 |
| BP | GO:0048660 | regulation of smooth muscle cell proliferation | 5/68 | 8.11 | 3.79E-04 | 6.51E-03 | APOD/IGFBP3/APOE/ANG/ERN1 | 5 |
| BP | GO:1903034 | regulation of response to wounding | 5/68 | 8.02 | 4.00E-04 | 6.79E-03 | F12/PLG/APOE/SERPINC1/VTN | 5 |
| BP | GO:1903035 | negative regulation of response to wounding | 4/68 | 11.44 | 4.15E-04 | 6.98E-03 | F12/PLG/APOE/VTN | 4 |
| BP | GO:0048659 | smooth muscle cell proliferation | 5/68 | 7.93 | 4.21E-04 | 7.00E-03 | APOD/IGFBP3/APOE/ANG/ERN1 | 5 |
| BP | GO:0071634 | regulation of transforming growth factor beta production | 3/68 | 19.82 | 4.65E-04 | 7.59E-03 | FN1/TNXB/AGT | 3 |
| BP | GO:0042632 | cholesterol homeostasis | 4/68 | 11.10 | 4.66E-04 | 7.59E-03 | APOA4/APOE/APOC3/CETP | 4 |
| BP | GO:0042542 | response to hydrogen peroxide | 4/68 | 10.99 | 4.84E-04 | 7.72E-03 | HBA2/HBB/HP/ERN1 | 4 |
| BP | GO:0055092 | sterol homeostasis | 4/68 | 10.99 | 4.84E-04 | 7.72E-03 | APOA4/APOE/APOC3/CETP | 4 |
| BP | GO:0015914 | phospholipid transport | 4/68 | 10.78 | 5.21E-04 | 8.23E-03 | APOA4/APOE/APOC3/CETP | 4 |
| BP | GO:0014012 | peripheral nervous system axon regeneration | 2/68 | 55.50 | 5.65E-04 | 8.73E-03 | APOA4/APOD | 2 |
| BP | GO:1901388 | regulation of transforming growth factor beta activation | 2/68 | 55.50 | 5.65E-04 | 8.73E-03 | TNXB/AGT | 2 |
| BP | GO:0071604 | transforming growth factor beta production | 3/68 | 18.50 | 5.70E-04 | 8.73E-03 | FN1/TNXB/AGT | 3 |
| BP | GO:0030100 | regulation of endocytosis | 6/68 | 5.72 | 6.27E-04 | 9.52E-03 | ACTB/TF/MBL2/APOE/APOC3/VTN | 6 |
| BP | GO:0001867 | complement activation, lectin pathway | 2/68 | 50.45 | 6.89E-04 | 1.03E-02 | MBL2/MASP1 | 2 |
| BP | GO:0010982 | regulation of high-density lipoprotein particle clearance | 2/68 | 50.45 | 6.89E-04 | 1.03E-02 | GPLD1/APOC3 | 2 |
| BP | GO:0045833 | negative regulation of lipid metabolic process | 4/68 | 9.91 | 7.15E-04 | 1.05E-02 | APOD/GPLD1/APOE/APOC3 | 4 |
| BP | GO:0036363 | transforming growth factor beta activation | 2/68 | 42.69 | 9.73E-04 | 1.38E-02 | TNXB/AGT | 2 |
| BP | GO:0051918 | negative regulation of fibrinolysis | 2/68 | 42.69 | 9.73E-04 | 1.38E-02 | PLG/VTN | 2 |
| BP | GO:0010874 | regulation of cholesterol efflux | 3/68 | 15.42 | 9.73E-04 | 1.38E-02 | PON1/APOE/CETP | 3 |
| BP | GO:0120009 | intermembrane lipid transfer | 3/68 | 15.42 | 9.73E-04 | 1.38E-02 | APOA4/APOE/CETP | 3 |
| BP | GO:0034381 | plasma lipoprotein particle clearance | 3/68 | 15.14 | 1.03E-03 | 1.45E-02 | GPLD1/APOE/APOC3 | 3 |
| BP | GO:0045216 | cell-cell junction organization | 5/68 | 6.48 | 1.04E-03 | 1.46E-02 | ACTB/CDH5/CLDN10/AGT/CSF1R | 5 |
| BP | GO:0044403 | biological process involved in symbiotic interaction | 6/68 | 5.17 | 1.06E-03 | 1.47E-02 | FN1/MBL2/PLG/APOE/CFHR1/CSF1R | 6 |
| BP | GO:2001140 | positive regulation of phospholipid transport | 2/68 | 39.64 | 1.13E-03 | 1.55E-02 | APOE/CETP | 2 |
| BP | GO:0042742 | defense response to bacterium | 6/68 | 5.05 | 1.20E-03 | 1.64E-02 | TF/MBL2/ANG/JCHAIN/HP/LBP | 6 |
| BP | GO:2001138 | regulation of phospholipid transport | 2/68 | 37.00 | 1.30E-03 | 1.76E-02 | APOE/CETP | 2 |
| BP | GO:0019755 | one-carbon compound transport | 3/68 | 13.65 | 1.39E-03 | 1.84E-02 | HBD/HBA2/HBB | 3 |
| BP | GO:0050994 | regulation of lipid catabolic process | 3/68 | 13.65 | 1.39E-03 | 1.84E-02 | APOA4/GPLD1/APOC3 | 3 |
| BP | GO:0042157 | lipoprotein metabolic process | 4/68 | 8.22 | 1.43E-03 | 1.88E-02 | APOA4/APOD/APOE/APOC3 | 4 |
| BP | GO:0090303 | positive regulation of wound healing | 3/68 | 13.43 | 1.45E-03 | 1.89E-02 | F12/PLG/VTN | 3 |
| BP | GO:0051004 | regulation of lipoprotein lipase activity | 2/68 | 34.69 | 1.49E-03 | 1.90E-02 | APOA4/APOC3 | 2 |
| BP | GO:0051044 | positive regulation of membrane protein ectodomain proteolysis | 2/68 | 34.69 | 1.49E-03 | 1.90E-02 | GPLD1/APOE | 2 |
| BP | GO:0045861 | negative regulation of proteolysis | 5/68 | 5.90 | 1.58E-03 | 2.01E-02 | FETUB/SERPINA5/SERPINA4/SERPINA1/VTN | 5 |
| BP | GO:0008203 | cholesterol metabolic process | 4/68 | 7.93 | 1.64E-03 | 2.06E-02 | APOA4/PON1/APOE/CETP | 4 |
| BP | GO:0046503 | glycerolipid catabolic process | 3/68 | 12.81 | 1.67E-03 | 2.07E-02 | APOA4/GPLD1/APOC3 | 3 |
| BP | GO:0008228 | opsonization | 2/68 | 32.65 | 1.68E-03 | 2.07E-02 | MBL2/LBP | 2 |
| BP | GO:0032488 | Cdc42 protein signal transduction | 2/68 | 32.65 | 1.68E-03 | 2.07E-02 | APOE/APOC3 | 2 |
| BP | GO:0043393 | regulation of protein binding | 4/68 | 7.82 | 1.72E-03 | 2.10E-02 | ACTB/APOE/CFHR1/VTN | 4 |
| BP | GO:0010755 | regulation of plasminogen activation | 2/68 | 30.83 | 1.89E-03 | 2.25E-02 | F12/CLEC3B | 2 |
| BP | GO:0033194 | response to hydroperoxide | 2/68 | 30.83 | 1.89E-03 | 2.25E-02 | APOA4/GPX3 | 2 |
| BP | GO:0060263 | regulation of respiratory burst | 2/68 | 30.83 | 1.89E-03 | 2.25E-02 | JCHAIN/LBP | 2 |
| BP | GO:0019731 | antibacterial humoral response | 3/68 | 11.89 | 2.06E-03 | 2.44E-02 | TF/ANG/JCHAIN | 3 |
| BP | GO:1902652 | secondary alcohol metabolic process | 4/68 | 7.40 | 2.11E-03 | 2.47E-02 | APOA4/PON1/APOE/CETP | 4 |
| BP | GO:0015748 | organophosphate ester transport | 4/68 | 7.30 | 2.21E-03 | 2.58E-02 | APOA4/APOE/APOC3/CETP | 4 |
| BP | GO:0016125 | sterol metabolic process | 4/68 | 7.21 | 2.32E-03 | 2.68E-02 | APOA4/PON1/APOE/CETP | 4 |
| BP | GO:0036498 | IRE1-mediated unfolded protein response | 2/68 | 27.75 | 2.33E-03 | 2.68E-02 | HSPA5/ERN1 | 2 |
| BP | GO:0045807 | positive regulation of endocytosis | 4/68 | 7.16 | 2.37E-03 | 2.71E-02 | TF/MBL2/APOE/VTN | 4 |
| BP | GO:0007043 | cell-cell junction assembly | 4/68 | 7.07 | 2.48E-03 | 2.81E-02 | ACTB/CDH5/CLDN10/AGT | 4 |
| BP | GO:0051098 | regulation of binding | 5/68 | 5.30 | 2.54E-03 | 2.86E-02 | ACTB/PON1/APOE/CFHR1/VTN | 5 |
| BP | GO:0044794 | positive regulation by host of viral process | 2/68 | 26.43 | 2.57E-03 | 2.87E-02 | APOE/CSF1R | 2 |
| BP | GO:1903036 | positive regulation of response to wounding | 3/68 | 10.81 | 2.71E-03 | 3.00E-02 | F12/PLG/VTN | 3 |
| BP | GO:0060191 | regulation of lipase activity | 3/68 | 10.67 | 2.81E-03 | 3.06E-02 | APOA4/APOC3/ANG | 3 |
| BP | GO:0090208 | positive regulation of triglyceride metabolic process | 2/68 | 25.23 | 2.82E-03 | 3.06E-02 | APOA4/GPLD1 | 2 |
| BP | GO:0150105 | protein localization to cell-cell junction | 2/68 | 25.23 | 2.82E-03 | 3.06E-02 | ACTB/CDH5 | 2 |
| BP | GO:0071636 | positive regulation of transforming growth factor beta production | 2/68 | 24.13 | 3.08E-03 | 3.32E-02 | TNXB/AGT | 2 |
| BP | GO:0051341 | regulation of oxidoreductase activity | 3/68 | 10.15 | 3.23E-03 | 3.46E-02 | AGT/APOE/HP | 3 |
| BP | GO:0051043 | regulation of membrane protein ectodomain proteolysis | 2/68 | 23.13 | 3.36E-03 | 3.57E-02 | GPLD1/APOE | 2 |
| BP | GO:0051897 | positive regulation of phosphatidylinositol 3-kinase/protein kinase B signal transduction | 4/68 | 6.49 | 3.38E-03 | 3.57E-02 | FN1/F10/AGT/CSF1R | 4 |
| BP | GO:0043542 | endothelial cell migration | 5/68 | 4.89 | 3.59E-03 | 3.76E-02 | GPLD1/CDH5/AGT/PLG/APOE | 5 |
| BP | GO:0010954 | positive regulation of protein processing | 2/68 | 22.20 | 3.64E-03 | 3.79E-02 | F12/CLEC3B | 2 |
| BP | GO:0055088 | lipid homeostasis | 4/68 | 6.34 | 3.67E-03 | 3.80E-02 | APOA4/APOE/APOC3/CETP | 4 |
| BP | GO:0032370 | positive regulation of lipid transport | 3/68 | 9.68 | 3.70E-03 | 3.80E-02 | PON1/APOE/CETP | 3 |
| BP | GO:0051347 | positive regulation of transferase activity | 6/68 | 4.02 | 3.74E-03 | 3.82E-02 | APOA4/AGT/APOE/ANG/CSF1R/ERN1 | 6 |
| BP | GO:0052548 | regulation of endopeptidase activity | 5/68 | 4.82 | 3.81E-03 | 3.86E-02 | FETUB/SERPINA5/SERPINA4/SERPINA1/VTN | 5 |
| BP | GO:0043534 | blood vessel endothelial cell migration | 4/68 | 6.13 | 4.14E-03 | 4.14E-02 | GPLD1/CDH5/PLG/APOE | 4 |
| BP | GO:0046889 | positive regulation of lipid biosynthetic process | 3/68 | 9.25 | 4.20E-03 | 4.14E-02 | APOA4/GPLD1/APOE | 3 |
| BP | GO:0010875 | positive regulation of cholesterol efflux | 2/68 | 20.56 | 4.24E-03 | 4.14E-02 | PON1/APOE | 2 |
| BP | GO:0031639 | plasminogen activation | 2/68 | 20.56 | 4.24E-03 | 4.14E-02 | F12/CLEC3B | 2 |
| BP | GO:0050995 | negative regulation of lipid catabolic process | 2/68 | 20.56 | 4.24E-03 | 4.14E-02 | GPLD1/APOC3 | 2 |
| BP | GO:1903319 | positive regulation of protein maturation | 2/68 | 20.56 | 4.24E-03 | 4.14E-02 | F12/CLEC3B | 2 |
| BP | GO:0048771 | tissue remodeling | 4/68 | 6.03 | 4.38E-03 | 4.26E-02 | TF/AGT/PLG/CSF1R | 4 |
| BP | GO:0031640 | killing of cells of another organism | 3/68 | 9.05 | 4.47E-03 | 4.26E-02 | MBL2/CFHR1/C9 | 3 |
| BP | GO:0141061 | disruption of cell in another organism | 3/68 | 9.05 | 4.47E-03 | 4.26E-02 | MBL2/CFHR1/C9 | 3 |
| BP | GO:0018149 | peptide cross-linking | 2/68 | 19.82 | 4.55E-03 | 4.26E-02 | FN1/F13A1 | 2 |
| BP | GO:0051873 | killing by host of symbiont cells | 2/68 | 19.82 | 4.55E-03 | 4.26E-02 | MBL2/CFHR1 | 2 |
| BP | GO:0055022 | negative regulation of cardiac muscle tissue growth | 2/68 | 19.82 | 4.55E-03 | 4.26E-02 | PI16/RBP4 | 2 |
| BP | GO:0061117 | negative regulation of heart growth | 2/68 | 19.82 | 4.55E-03 | 4.26E-02 | PI16/RBP4 | 2 |
| BP | GO:0046890 | regulation of lipid biosynthetic process | 4/68 | 5.87 | 4.82E-03 | 4.48E-02 | APOA4/GPLD1/APOE/APOC3 | 4 |
| BP | GO:0006910 | phagocytosis, recognition | 2/68 | 19.14 | 4.88E-03 | 4.51E-02 | MBL2/LBP | 2 |
| BP | GO:0031099 | regeneration | 4/68 | 5.81 | 5.00E-03 | 4.60E-02 | APOA4/APOD/VTN/LCP1 | 4 |
| BP | GO:0006006 | glucose metabolic process | 4/68 | 5.75 | 5.19E-03 | 4.66E-02 | APOD/IGFBP3/GPLD1/RBP4 | 4 |
| BP | GO:0007263 | nitric oxide mediated signal transduction | 2/68 | 18.50 | 5.22E-03 | 4.66E-02 | AGT/APOE | 2 |
| BP | GO:0035640 | exploration behavior | 2/68 | 18.50 | 5.22E-03 | 4.66E-02 | CHL1/APOE | 2 |
| BP | GO:0061098 | positive regulation of protein tyrosine kinase activity | 2/68 | 18.50 | 5.22E-03 | 4.66E-02 | AGT/CSF1R | 2 |
| BP | GO:1900101 | regulation of endoplasmic reticulum unfolded protein response | 2/68 | 18.50 | 5.22E-03 | 4.66E-02 | HSPA5/ERN1 | 2 |
| BP | GO:0052547 | regulation of peptidase activity | 5/68 | 4.45 | 5.33E-03 | 4.74E-02 | FETUB/SERPINA5/SERPINA4/SERPINA1/VTN | 5 |
| BP | GO:0034329 | cell junction assembly | 6/68 | 3.73 | 5.36E-03 | 4.74E-02 | FN1/ACTB/APOD/CDH5/CLDN10/AGT | 6 |
| BP | GO:0141060 | disruption of anatomical structure in another organism | 3/68 | 8.41 | 5.49E-03 | 4.82E-02 | MBL2/CFHR1/C9 | 3 |
| BP | GO:0030198 | extracellular matrix organization | 5/68 | 4.32 | 6.00E-03 | 5.24E-02 | TNXB/AGT/PLG/VTN/LCP1 | 5 |
| BP | GO:0043062 | extracellular structure organization | 5/68 | 4.31 | 6.08E-03 | 5.28E-02 | TNXB/AGT/PLG/VTN/LCP1 | 5 |
| BP | GO:0045229 | external encapsulating structure organization | 5/68 | 4.30 | 6.16E-03 | 5.32E-02 | TNXB/AGT/PLG/VTN/LCP1 | 5 |
| BP | GO:0010743 | regulation of macrophage derived foam cell differentiation | 2/68 | 16.82 | 6.29E-03 | 5.37E-02 | AGT/CETP | 2 |
| BP | GO:0032642 | regulation of chemokine production | 3/68 | 8.00 | 6.29E-03 | 5.37E-02 | APOD/LBP/CSF1R | 3 |
| BP | GO:0016064 | immunoglobulin mediated immune response | 4/68 | 5.41 | 6.41E-03 | 5.44E-02 | MBL2/IGHV3-7/C9/IGHV1-45 | 4 |
| BP | GO:0032602 | chemokine production | 3/68 | 7.93 | 6.46E-03 | 5.45E-02 | APOD/LBP/CSF1R | 3 |
| BP | GO:0008202 | steroid metabolic process | 5/68 | 4.24 | 6.48E-03 | 5.45E-02 | GC/APOA4/PON1/APOE/CETP | 5 |
| BP | GO:0048638 | regulation of developmental growth | 5/68 | 4.23 | 6.56E-03 | 5.49E-02 | FN1/PI16/ATRN/APOE/RBP4 | 5 |
| BP | GO:0030212 | hyaluronan metabolic process | 2/68 | 16.32 | 6.66E-03 | 5.54E-02 | ITIH2/ITIH1 | 2 |
| BP | GO:0019724 | B cell mediated immunity | 4/68 | 5.34 | 6.74E-03 | 5.57E-02 | MBL2/IGHV3-7/C9/IGHV1-45 | 4 |
| BP | GO:0007173 | epidermal growth factor receptor signaling pathway | 3/68 | 7.78 | 6.80E-03 | 5.59E-02 | PIGR/EFEMP1/AGT | 3 |
| BP | GO:0070633 | transepithelial transport | 2/68 | 15.86 | 7.05E-03 | 5.77E-02 | ACTB/GPLD1 | 2 |
| BP | GO:0062012 | regulation of small molecule metabolic process | 5/68 | 4.12 | 7.34E-03 | 5.97E-02 | APOA4/IGFBP3/GPLD1/APOE/APOC3 | 5 |
| BP | GO:0010984 | regulation of lipoprotein particle clearance | 2/68 | 15.42 | 7.45E-03 | 6.03E-02 | GPLD1/APOC3 | 2 |
| BP | GO:1905954 | positive regulation of lipid localization | 3/68 | 7.50 | 7.52E-03 | 6.06E-02 | PON1/APOE/CETP | 3 |
| BP | GO:0019216 | regulation of lipid metabolic process | 5/68 | 4.06 | 7.79E-03 | 6.25E-02 | APOA4/APOD/GPLD1/APOE/APOC3 | 5 |
| BP | GO:0046621 | negative regulation of organ growth | 2/68 | 15.00 | 7.86E-03 | 6.26E-02 | PI16/RBP4 | 2 |
| BP | GO:0010810 | regulation of cell-substrate adhesion | 4/68 | 5.05 | 8.18E-03 | 6.48E-02 | FN1/APOD/PLG/VTN | 4 |
| BP | GO:0010737 | protein kinase A signaling | 2/68 | 14.61 | 8.27E-03 | 6.48E-02 | TCP11/LCP1 | 2 |
| BP | GO:0035633 | maintenance of blood-brain barrier | 2/68 | 14.61 | 8.27E-03 | 6.48E-02 | ACTB/CDH5 | 2 |
| BP | GO:0048259 | regulation of receptor-mediated endocytosis | 3/68 | 7.24 | 8.29E-03 | 6.48E-02 | TF/APOC3/VTN | 3 |
| BP | GO:0002385 | mucosal immune response | 2/68 | 14.23 | 8.70E-03 | 6.77E-02 | PIGR/APOA4 | 2 |
| BP | GO:0050830 | defense response to Gram-positive bacterium | 3/68 | 7.06 | 8.89E-03 | 6.88E-02 | MBL2/ANG/LBP | 3 |
| BP | GO:0001667 | ameboidal-type cell migration | 6/68 | 3.35 | 8.94E-03 | 6.88E-02 | FN1/GPLD1/CDH5/AGT/PLG/APOE | 6 |
| BP | GO:0010742 | macrophage derived foam cell differentiation | 2/68 | 13.88 | 9.14E-03 | 6.96E-02 | AGT/CETP | 2 |
| BP | GO:0045730 | respiratory burst | 2/68 | 13.88 | 9.14E-03 | 6.96E-02 | JCHAIN/LBP | 2 |
| BP | GO:0031589 | cell-substrate adhesion | 5/68 | 3.90 | 9.18E-03 | 6.96E-02 | FN1/APOD/TNXB/PLG/VTN | 5 |
| BP | GO:0090287 | regulation of cellular response to growth factor stimulus | 5/68 | 3.89 | 9.28E-03 | 7.01E-02 | CDH5/TNXB/HSPA5/AGT/VTN | 5 |
| BP | GO:0090077 | foam cell differentiation | 2/68 | 13.54 | 9.58E-03 | 7.20E-02 | AGT/CETP | 2 |
| BP | GO:0010594 | regulation of endothelial cell migration | 4/68 | 4.81 | 9.67E-03 | 7.23E-02 | GPLD1/AGT/PLG/APOE | 4 |
| BP | GO:0006958 | complement activation, classical pathway | 2/68 | 13.21 | 1.00E-02 | 7.44E-02 | MBL2/C9 | 2 |
| BP | GO:0045124 | regulation of bone resorption | 2/68 | 13.21 | 1.00E-02 | 7.44E-02 | TF/CSF1R | 2 |
| BP | GO:0038127 | ERBB signaling pathway | 3/68 | 6.71 | 1.02E-02 | 7.50E-02 | PIGR/EFEMP1/AGT | 3 |
| BP | GO:0002251 | organ or tissue specific immune response | 2/68 | 12.91 | 1.05E-02 | 7.67E-02 | PIGR/APOA4 | 2 |
| BP | GO:0045663 | positive regulation of myoblast differentiation | 2/68 | 12.91 | 1.05E-02 | 7.67E-02 | ACTB/IGFBP3 | 2 |
| BP | GO:0003014 | renal system process | 3/68 | 6.61 | 1.06E-02 | 7.72E-02 | AGT/HBB/JCHAIN | 3 |
| BP | GO:0007160 | cell-matrix adhesion | 4/68 | 4.64 | 1.09E-02 | 7.80E-02 | FN1/APOD/TNXB/VTN | 4 |
| BP | GO:0051099 | positive regulation of binding | 3/68 | 6.56 | 1.09E-02 | 7.80E-02 | PON1/APOE/VTN | 3 |
| BP | GO:0010631 | epithelial cell migration | 5/68 | 3.73 | 1.10E-02 | 7.80E-02 | GPLD1/CDH5/AGT/PLG/APOE | 5 |
| BP | GO:0006509 | membrane protein ectodomain proteolysis | 2/68 | 12.61 | 1.10E-02 | 7.80E-02 | GPLD1/APOE | 2 |
| BP | GO:0021762 | substantia nigra development | 2/68 | 12.61 | 1.10E-02 | 7.80E-02 | ACTB/HSPA5 | 2 |
| BP | GO:0006066 | alcohol metabolic process | 5/68 | 3.72 | 1.11E-02 | 7.83E-02 | APOA4/PON1/APOE/CETP/RBP4 | 5 |
| BP | GO:0090132 | epithelium migration | 5/68 | 3.70 | 1.13E-02 | 7.97E-02 | GPLD1/CDH5/AGT/PLG/APOE | 5 |
| BP | GO:0010595 | positive regulation of endothelial cell migration | 3/68 | 6.40 | 1.16E-02 | 8.10E-02 | GPLD1/AGT/PLG | 3 |
| BP | GO:0016485 | protein processing | 4/68 | 4.53 | 1.18E-02 | 8.24E-02 | F12/CLEC3B/MASP1/HGFAC | 4 |
| BP | GO:0090130 | tissue migration | 5/68 | 3.65 | 1.19E-02 | 8.27E-02 | GPLD1/CDH5/AGT/PLG/APOE | 5 |
| BP | GO:0085029 | extracellular matrix assembly | 2/68 | 12.07 | 1.20E-02 | 8.27E-02 | TNXB/AGT | 2 |
| BP | GO:0019730 | antimicrobial humoral response | 3/68 | 6.26 | 1.23E-02 | 8.39E-02 | TF/ANG/JCHAIN | 3 |
| BP | GO:0030168 | platelet activation | 3/68 | 6.26 | 1.23E-02 | 8.39E-02 | ACTB/HBB/APOE | 3 |
| BP | GO:0051896 | regulation of phosphatidylinositol 3-kinase/protein kinase B signal transduction | 4/68 | 4.48 | 1.23E-02 | 8.39E-02 | FN1/F10/AGT/CSF1R | 4 |
| BP | GO:0051353 | positive regulation of oxidoreductase activity | 2/68 | 11.81 | 1.25E-02 | 8.47E-02 | AGT/APOE | 2 |
| BP | GO:0044331 | cell-cell adhesion mediated by cadherin | 2/68 | 11.56 | 1.30E-02 | 8.78E-02 | CDH5/PLG | 2 |
| BP | GO:0035987 | endodermal cell differentiation | 2/68 | 11.33 | 1.35E-02 | 9.05E-02 | FN1/VTN | 2 |
| BP | GO:0046850 | regulation of bone remodeling | 2/68 | 11.33 | 1.35E-02 | 9.05E-02 | TF/CSF1R | 2 |
| BP | GO:0043114 | regulation of vascular permeability | 2/68 | 11.10 | 1.40E-02 | 9.30E-02 | CDH5/APOE | 2 |
| BP | GO:0044788 | modulation by host of viral process | 2/68 | 11.10 | 1.40E-02 | 9.30E-02 | APOE/CSF1R | 2 |
| BP | GO:0006898 | receptor-mediated endocytosis | 4/68 | 4.30 | 1.40E-02 | 9.30E-02 | TF/APOE/APOC3/VTN | 4 |
| BP | GO:0043407 | negative regulation of MAP kinase activity | 2/68 | 10.88 | 1.46E-02 | 9.52E-02 | AGT/APOE | 2 |
| BP | GO:0046460 | neutral lipid biosynthetic process | 2/68 | 10.88 | 1.46E-02 | 9.52E-02 | GPLD1/ANG | 2 |
| BP | GO:0046463 | acylglycerol biosynthetic process | 2/68 | 10.88 | 1.46E-02 | 9.52E-02 | GPLD1/ANG | 2 |
| BP | GO:0043405 | regulation of MAP kinase activity | 3/68 | 5.86 | 1.46E-02 | 9.54E-02 | AGT/APOE/ERN1 | 3 |
| BP | GO:0006968 | cellular defense response | 2/68 | 10.67 | 1.51E-02 | 9.72E-02 | LGALS3BP/LBP | 2 |
| BP | GO:0031103 | axon regeneration | 2/68 | 10.67 | 1.51E-02 | 9.72E-02 | APOA4/APOD | 2 |
| BP | GO:0035306 | positive regulation of dephosphorylation | 2/68 | 10.67 | 1.51E-02 | 9.72E-02 | GPLD1/CDH5 | 2 |
| BP | GO:0050714 | positive regulation of protein secretion | 3/68 | 5.78 | 1.52E-02 | 9.74E-02 | GPLD1/RBP4/ANG | 3 |
| BP | GO:0045834 | positive regulation of lipid metabolic process | 3/68 | 5.74 | 1.55E-02 | 9.82E-02 | APOA4/GPLD1/APOE | 3 |
| BP | GO:0050708 | regulation of protein secretion | 4/68 | 4.17 | 1.55E-02 | 9.82E-02 | GPLD1/APOE/RBP4/ANG | 4 |
| BP | GO:0048146 | positive regulation of fibroblast proliferation | 2/68 | 10.47 | 1.57E-02 | 9.82E-02 | FN1/AGT | 2 |
| BP | GO:0048260 | positive regulation of receptor-mediated endocytosis | 2/68 | 10.47 | 1.57E-02 | 9.82E-02 | TF/VTN | 2 |
| BP | GO:0071622 | regulation of granulocyte chemotaxis | 2/68 | 10.47 | 1.57E-02 | 9.82E-02 | LBP/CSF1R | 2 |
| BP | GO:0035296 | regulation of tube diameter | 3/68 | 5.70 | 1.58E-02 | 9.82E-02 | AGT/HBB/APOE | 3 |
| BP | GO:0097746 | blood vessel diameter maintenance | 3/68 | 5.70 | 1.58E-02 | 9.82E-02 | AGT/HBB/APOE | 3 |
| BP | GO:0035150 | regulation of tube size | 3/68 | 5.66 | 1.61E-02 | 9.92E-02 | AGT/HBB/APOE | 3 |
| BP | GO:0062013 | positive regulation of small molecule metabolic process | 3/68 | 5.66 | 1.61E-02 | 9.92E-02 | APOA4/GPLD1/APOE | 3 |
| BP | GO:0003018 | vascular process in circulatory system | 4/68 | 4.13 | 1.61E-02 | 9.92E-02 | CDH5/AGT/HBB/APOE | 4 |
| BP | GO:0002455 | humoral immune response mediated by circulating immunoglobulin | 2/68 | 10.09 | 1.68E-02 | 1.03E-01 | MBL2/C9 | 2 |
| BP | GO:0042304 | regulation of fatty acid biosynthetic process | 2/68 | 10.09 | 1.68E-02 | 1.03E-01 | APOA4/APOC3 | 2 |
| BP | GO:0001558 | regulation of cell growth | 5/68 | 3.33 | 1.72E-02 | 1.05E-01 | FN1/IGFBP3/PI16/AGT/APOE | 5 |
| BP | GO:0061097 | regulation of protein tyrosine kinase activity | 2/68 | 9.91 | 1.74E-02 | 1.05E-01 | AGT/CSF1R | 2 |
| BP | GO:0018108 | peptidyl-tyrosine phosphorylation | 4/68 | 4.02 | 1.76E-02 | 1.06E-01 | EFEMP1/AGT/VTN/CSF1R | 4 |
| BP | GO:0018212 | peptidyl-tyrosine modification | 4/68 | 3.99 | 1.80E-02 | 1.07E-01 | EFEMP1/AGT/VTN/CSF1R | 4 |
| BP | GO:0034332 | adherens junction organization | 2/68 | 9.74 | 1.80E-02 | 1.07E-01 | ACTB/CDH5 | 2 |
| BP | GO:0043113 | receptor clustering | 2/68 | 9.74 | 1.80E-02 | 1.07E-01 | PIGR/APOE | 2 |
| BP | GO:0008037 | cell recognition | 3/68 | 5.41 | 1.81E-02 | 1.08E-01 | FETUB/MBL2/LBP | 3 |
| BP | GO:0050727 | regulation of inflammatory response | 5/68 | 3.26 | 1.85E-02 | 1.09E-01 | F12/CDH5/AGT/APOE/LBP | 5 |
| BP | GO:0001706 | endoderm formation | 2/68 | 9.57 | 1.86E-02 | 1.09E-01 | FN1/VTN | 2 |
| BP | GO:0031102 | neuron projection regeneration | 2/68 | 9.57 | 1.86E-02 | 1.09E-01 | APOA4/APOD | 2 |
| BP | GO:0045860 | positive regulation of protein kinase activity | 4/68 | 3.94 | 1.88E-02 | 1.10E-01 | AGT/ANG/CSF1R/ERN1 | 4 |
| BP | GO:0010718 | positive regulation of epithelial to mesenchymal transition | 2/68 | 9.41 | 1.92E-02 | 1.11E-01 | TNXB/AGT | 2 |
| BP | GO:0033619 | membrane protein proteolysis | 2/68 | 9.41 | 1.92E-02 | 1.11E-01 | GPLD1/APOE | 2 |
| BP | GO:0071900 | regulation of protein serine/threonine kinase activity | 4/68 | 3.89 | 1.95E-02 | 1.13E-01 | ACTB/AGT/APOE/ERN1 | 4 |
| BP | GO:0035966 | response to topologically incorrect protein | 3/68 | 5.24 | 1.97E-02 | 1.14E-01 | F12/HSPA5/ERN1 | 3 |
| BP | GO:0060193 | positive regulation of lipase activity | 2/68 | 9.25 | 1.98E-02 | 1.14E-01 | APOA4/ANG | 2 |
| BP | GO:0071560 | cellular response to transforming growth factor beta stimulus | 4/68 | 3.87 | 2.00E-02 | 1.14E-01 | CLEC3B/CDH5/TNXB/HSPA5 | 4 |
| BP | GO:0032092 | positive regulation of protein binding | 2/68 | 9.10 | 2.04E-02 | 1.17E-01 | APOE/VTN | 2 |
| BP | GO:0043491 | phosphatidylinositol 3-kinase/protein kinase B signal transduction | 4/68 | 3.83 | 2.06E-02 | 1.17E-01 | FN1/F10/AGT/CSF1R | 4 |
| BP | GO:0048592 | eye morphogenesis | 3/68 | 5.14 | 2.07E-02 | 1.17E-01 | EFEMP1/MAN2A1/RBP4 | 3 |
| BP | GO:0022617 | extracellular matrix disassembly | 2/68 | 8.95 | 2.11E-02 | 1.18E-01 | PLG/LCP1 | 2 |
| BP | GO:1903053 | regulation of extracellular matrix organization | 2/68 | 8.95 | 2.11E-02 | 1.18E-01 | TNXB/AGT | 2 |
| BP | GO:0071559 | response to transforming growth factor beta | 4/68 | 3.79 | 2.13E-02 | 1.20E-01 | CLEC3B/CDH5/TNXB/HSPA5 | 4 |
| BP | GO:0010632 | regulation of epithelial cell migration | 4/68 | 3.76 | 2.18E-02 | 1.22E-01 | GPLD1/AGT/PLG/APOE | 4 |
| BP | GO:0031100 | animal organ regeneration | 2/68 | 8.67 | 2.23E-02 | 1.24E-01 | VTN/LCP1 | 2 |
| BP | GO:0006650 | glycerophospholipid metabolic process | 4/68 | 3.72 | 2.25E-02 | 1.24E-01 | APOA4/GPLD1/PON1/CETP | 4 |
| BP | GO:0007409 | axonogenesis | 5/68 | 3.10 | 2.27E-02 | 1.24E-01 | FN1/ACTB/CHL1/APOE/CSF1R | 5 |
| BP | GO:0006633 | fatty acid biosynthetic process | 3/68 | 4.96 | 2.28E-02 | 1.24E-01 | APOA4/APOC3/PTGDS | 3 |
| BP | GO:0050731 | positive regulation of peptidyl-tyrosine phosphorylation | 3/68 | 4.96 | 2.28E-02 | 1.24E-01 | AGT/VTN/CSF1R | 3 |
| BP | GO:0010812 | negative regulation of cell-substrate adhesion | 2/68 | 8.54 | 2.30E-02 | 1.24E-01 | APOD/PLG | 2 |
| BP | GO:0045453 | bone resorption | 2/68 | 8.54 | 2.30E-02 | 1.24E-01 | TF/CSF1R | 2 |
| BP | GO:0055021 | regulation of cardiac muscle tissue growth | 2/68 | 8.54 | 2.30E-02 | 1.24E-01 | PI16/RBP4 | 2 |
| BP | GO:0060042 | retina morphogenesis in camera-type eye | 2/68 | 8.54 | 2.30E-02 | 1.24E-01 | MAN2A1/RBP4 | 2 |
| BP | GO:0010611 | regulation of cardiac muscle hypertrophy | 2/68 | 8.28 | 2.43E-02 | 1.30E-01 | PI16/AGT | 2 |
| BP | GO:0048857 | neural nucleus development | 2/68 | 8.28 | 2.43E-02 | 1.30E-01 | ACTB/HSPA5 | 2 |
| BP | GO:0070613 | regulation of protein processing | 2/68 | 8.28 | 2.43E-02 | 1.30E-01 | F12/CLEC3B | 2 |
| BP | GO:0051055 | negative regulation of lipid biosynthetic process | 2/68 | 8.16 | 2.50E-02 | 1.33E-01 | APOE/APOC3 | 2 |
| BP | GO:0010634 | positive regulation of epithelial cell migration | 3/68 | 4.73 | 2.57E-02 | 1.36E-01 | GPLD1/AGT/PLG | 3 |
| BP | GO:0043409 | negative regulation of MAPK cascade | 3/68 | 4.73 | 2.57E-02 | 1.36E-01 | AMBP/AGT/APOE | 3 |
| BP | GO:0070830 | bicellular tight junction assembly | 2/68 | 8.04 | 2.57E-02 | 1.36E-01 | CDH5/CLDN10 | 2 |
| BP | GO:0002443 | leukocyte mediated immunity | 5/68 | 2.98 | 2.63E-02 | 1.38E-01 | MBL2/IGHV3-7/PTGDS/C9/IGHV1-45 | 5 |
| BP | GO:0002752 | cell surface pattern recognition receptor signaling pathway | 2/68 | 7.93 | 2.64E-02 | 1.38E-01 | MBL2/LBP | 2 |
| BP | GO:0014743 | regulation of muscle hypertrophy | 2/68 | 7.93 | 2.64E-02 | 1.38E-01 | PI16/AGT | 2 |
| BP | GO:0060420 | regulation of heart growth | 2/68 | 7.82 | 2.71E-02 | 1.41E-01 | PI16/RBP4 | 2 |
| BP | GO:1903317 | regulation of protein maturation | 2/68 | 7.82 | 2.71E-02 | 1.41E-01 | F12/CLEC3B | 2 |
| BP | GO:0032722 | positive regulation of chemokine production | 2/68 | 7.71 | 2.78E-02 | 1.43E-01 | LBP/CSF1R | 2 |
| BP | GO:0043954 | cellular component maintenance | 2/68 | 7.71 | 2.78E-02 | 1.43E-01 | APOE/CSF1R | 2 |
| BP | GO:0070527 | platelet aggregation | 2/68 | 7.71 | 2.78E-02 | 1.43E-01 | ACTB/HBB | 2 |
| BP | GO:0045661 | regulation of myoblast differentiation | 2/68 | 7.60 | 2.85E-02 | 1.46E-01 | ACTB/IGFBP3 | 2 |
| BP | GO:0050795 | regulation of behavior | 2/68 | 7.50 | 2.93E-02 | 1.49E-01 | APOE/PTGDS | 2 |
| BP | GO:0032102 | negative regulation of response to external stimulus | 5/68 | 2.88 | 2.96E-02 | 1.51E-01 | F12/CDH5/PLG/APOE/VTN | 5 |
| BP | GO:0030968 | endoplasmic reticulum unfolded protein response | 2/68 | 7.40 | 3.00E-02 | 1.51E-01 | HSPA5/ERN1 | 2 |
| BP | GO:0035924 | cellular response to vascular endothelial growth factor stimulus | 2/68 | 7.40 | 3.00E-02 | 1.51E-01 | TNXB/ERN1 | 2 |
| BP | GO:0120192 | tight junction assembly | 2/68 | 7.40 | 3.00E-02 | 1.51E-01 | CDH5/CLDN10 | 2 |
| BP | GO:0032091 | negative regulation of protein binding | 2/68 | 7.21 | 3.15E-02 | 1.58E-01 | ACTB/CFHR1 | 2 |
| BP | GO:0043297 | apical junction assembly | 2/68 | 7.21 | 3.15E-02 | 1.58E-01 | CDH5/CLDN10 | 2 |
| BP | GO:0032720 | negative regulation of tumor necrosis factor production | 2/68 | 7.12 | 3.22E-02 | 1.61E-01 | ORM1/LBP | 2 |
| BP | GO:0016042 | lipid catabolic process | 4/68 | 3.32 | 3.24E-02 | 1.61E-01 | APOA4/GPLD1/APOE/APOC3 | 4 |
| BP | GO:0070371 | ERK1 and ERK2 cascade | 4/68 | 3.30 | 3.30E-02 | 1.64E-01 | FN1/TF/APOE/CSF1R | 4 |
| BP | GO:0033674 | positive regulation of kinase activity | 4/68 | 3.29 | 3.33E-02 | 1.65E-01 | AGT/ANG/CSF1R/ERN1 | 4 |
| BP | GO:0016049 | cell growth | 5/68 | 2.79 | 3.34E-02 | 1.65E-01 | FN1/IGFBP3/PI16/AGT/APOE | 5 |
| BP | GO:1903556 | negative regulation of tumor necrosis factor superfamily cytokine production | 2/68 | 6.94 | 3.38E-02 | 1.65E-01 | ORM1/LBP | 2 |
| BP | GO:0001819 | positive regulation of cytokine production | 5/68 | 2.78 | 3.39E-02 | 1.65E-01 | TNXB/AGT/ORM1/LBP/CSF1R | 5 |
| BP | GO:0120193 | tight junction organization | 2/68 | 6.85 | 3.45E-02 | 1.65E-01 | CDH5/CLDN10 | 2 |
| BP | GO:1905897 | regulation of response to endoplasmic reticulum stress | 2/68 | 6.77 | 3.53E-02 | 1.65E-01 | HSPA5/ERN1 | 2 |
| BP | GO:0032429 | regulation of phospholipase A2 activity | 1/68 | 27.75 | 3.55E-02 | 1.65E-01 | ANG | 1 |
| BP | GO:0036093 | germ cell proliferation | 1/68 | 27.75 | 3.55E-02 | 1.65E-01 | PTGDS | 1 |
| BP | GO:0045541 | negative regulation of cholesterol biosynthetic process | 1/68 | 27.75 | 3.55E-02 | 1.65E-01 | APOE | 1 |
| BP | GO:0048251 | elastic fiber assembly | 1/68 | 27.75 | 3.55E-02 | 1.65E-01 | TNXB | 1 |
| BP | GO:0060068 | vagina development | 1/68 | 27.75 | 3.55E-02 | 1.65E-01 | RBP4 | 1 |
| BP | GO:0070391 | response to lipoteichoic acid | 1/68 | 27.75 | 3.55E-02 | 1.65E-01 | LBP | 1 |
| BP | GO:0071223 | cellular response to lipoteichoic acid | 1/68 | 27.75 | 3.55E-02 | 1.65E-01 | LBP | 1 |
| BP | GO:0071803 | positive regulation of podosome assembly | 1/68 | 27.75 | 3.55E-02 | 1.65E-01 | LCP1 | 1 |
| BP | GO:0097113 | AMPA glutamate receptor clustering | 1/68 | 27.75 | 3.55E-02 | 1.65E-01 | APOE | 1 |
| BP | GO:0097688 | glutamate receptor clustering | 1/68 | 27.75 | 3.55E-02 | 1.65E-01 | APOE | 1 |
| BP | GO:0106119 | negative regulation of sterol biosynthetic process | 1/68 | 27.75 | 3.55E-02 | 1.65E-01 | APOE | 1 |
| BP | GO:1903365 | regulation of fear response | 1/68 | 27.75 | 3.55E-02 | 1.65E-01 | APOE | 1 |
| BP | GO:1903596 | regulation of gap junction assembly | 1/68 | 27.75 | 3.55E-02 | 1.65E-01 | AGT | 1 |
| BP | GO:1904729 | regulation of intestinal lipid absorption | 1/68 | 27.75 | 3.55E-02 | 1.65E-01 | APOA4 | 1 |
| BP | GO:2000048 | negative regulation of cell-cell adhesion mediated by cadherin | 1/68 | 27.75 | 3.55E-02 | 1.65E-01 | PLG | 1 |
| BP | GO:0007492 | endoderm development | 2/68 | 6.69 | 3.61E-02 | 1.67E-01 | FN1/VTN | 2 |
| BP | GO:0035023 | regulation of Rho protein signal transduction | 2/68 | 6.69 | 3.61E-02 | 1.67E-01 | APOE/APOC3 | 2 |
| BP | GO:0002768 | immune response-regulating cell surface receptor signaling pathway | 4/68 | 3.21 | 3.62E-02 | 1.67E-01 | PIGR/GPLD1/MBL2/LBP | 4 |
| BP | GO:0001935 | endothelial cell proliferation | 3/68 | 4.12 | 3.65E-02 | 1.68E-01 | APOE/ANG/ERN1 | 3 |
| BP | GO:0032024 | positive regulation of insulin secretion | 2/68 | 6.61 | 3.69E-02 | 1.69E-01 | GPLD1/RBP4 | 2 |
| BP | GO:0048678 | response to axon injury | 2/68 | 6.61 | 3.69E-02 | 1.69E-01 | APOA4/APOD | 2 |
| BP | GO:0045862 | positive regulation of proteolysis | 4/68 | 3.17 | 3.75E-02 | 1.71E-01 | F12/CLEC3B/GPLD1/APOE | 4 |
| BP | GO:0034103 | regulation of tissue remodeling | 2/68 | 6.53 | 3.77E-02 | 1.71E-01 | TF/CSF1R | 2 |
| BP | GO:0006983 | ER overload response | 1/68 | 25.23 | 3.89E-02 | 1.71E-01 | HSPA5 | 1 |
| BP | GO:0010986 | positive regulation of lipoprotein particle clearance | 1/68 | 25.23 | 3.89E-02 | 1.71E-01 | GPLD1 | 1 |
| BP | GO:0034975 | protein folding in endoplasmic reticulum | 1/68 | 25.23 | 3.89E-02 | 1.71E-01 | HSPA5 | 1 |
| BP | GO:0035812 | renal sodium excretion | 1/68 | 25.23 | 3.89E-02 | 1.71E-01 | AGT | 1 |
| BP | GO:0042167 | heme catabolic process | 1/68 | 25.23 | 3.89E-02 | 1.71E-01 | AMBP | 1 |
| BP | GO:0044062 | regulation of excretion | 1/68 | 25.23 | 3.89E-02 | 1.71E-01 | AGT | 1 |
| BP | GO:0046149 | pigment catabolic process | 1/68 | 25.23 | 3.89E-02 | 1.71E-01 | AMBP | 1 |
| BP | GO:0051386 | regulation of neurotrophin TRK receptor signaling pathway | 1/68 | 25.23 | 3.89E-02 | 1.71E-01 | AGT | 1 |
| BP | GO:0090206 | negative regulation of cholesterol metabolic process | 1/68 | 25.23 | 3.89E-02 | 1.71E-01 | APOE | 1 |
| BP | GO:1903027 | regulation of opsonization | 1/68 | 25.23 | 3.89E-02 | 1.71E-01 | MBL2 | 1 |
| BP | GO:1903897 | regulation of PERK-mediated unfolded protein response | 1/68 | 25.23 | 3.89E-02 | 1.71E-01 | HSPA5 | 1 |
| BP | GO:2000644 | regulation of receptor catabolic process | 1/68 | 25.23 | 3.89E-02 | 1.71E-01 | APOE | 1 |
| BP | GO:0031330 | negative regulation of cellular catabolic process | 3/68 | 4.00 | 3.92E-02 | 1.72E-01 | GPLD1/APOC3/HP | 3 |
| BP | GO:0051851 | modulation by host of symbiont process | 2/68 | 6.38 | 3.93E-02 | 1.72E-01 | APOE/CSF1R | 2 |
| BP | GO:0032147 | activation of protein kinase activity | 2/68 | 6.31 | 4.02E-02 | 1.75E-01 | AGT/ANG | 2 |
| BP | GO:0014910 | regulation of smooth muscle cell migration | 2/68 | 6.24 | 4.10E-02 | 1.76E-01 | IGFBP3/VTN | 2 |
| BP | GO:0034620 | cellular response to unfolded protein | 2/68 | 6.24 | 4.10E-02 | 1.76E-01 | HSPA5/ERN1 | 2 |
| BP | GO:0046849 | bone remodeling | 2/68 | 6.24 | 4.10E-02 | 1.76E-01 | TF/CSF1R | 2 |
| BP | GO:0048145 | regulation of fibroblast proliferation | 2/68 | 6.24 | 4.10E-02 | 1.76E-01 | FN1/AGT | 2 |
| BP | GO:0055017 | cardiac muscle tissue growth | 2/68 | 6.24 | 4.10E-02 | 1.76E-01 | PI16/RBP4 | 2 |
| BP | GO:0034379 | very-low-density lipoprotein particle assembly | 1/68 | 23.13 | 4.24E-02 | 1.79E-01 | APOC3 | 1 |
| BP | GO:0035112 | genitalia morphogenesis | 1/68 | 23.13 | 4.24E-02 | 1.79E-01 | RBP4 | 1 |
| BP | GO:0035437 | maintenance of protein localization in endoplasmic reticulum | 1/68 | 23.13 | 4.24E-02 | 1.79E-01 | HSPA5 | 1 |
| BP | GO:0042117 | monocyte activation | 1/68 | 23.13 | 4.24E-02 | 1.79E-01 | FN1 | 1 |
| BP | GO:0071635 | negative regulation of transforming growth factor beta production | 1/68 | 23.13 | 4.24E-02 | 1.79E-01 | FN1 | 1 |
| BP | GO:1904478 | regulation of intestinal absorption | 1/68 | 23.13 | 4.24E-02 | 1.79E-01 | APOA4 | 1 |
| BP | GO:0002220 | innate immune response activating cell surface receptor signaling pathway | 2/68 | 6.10 | 4.27E-02 | 1.79E-01 | MBL2/LBP | 2 |
| BP | GO:0030901 | midbrain development | 2/68 | 6.10 | 4.27E-02 | 1.79E-01 | ACTB/HSPA5 | 2 |
| BP | GO:0030516 | regulation of axon extension | 2/68 | 6.03 | 4.35E-02 | 1.79E-01 | FN1/APOE | 2 |
| BP | GO:1903510 | mucopolysaccharide metabolic process | 2/68 | 6.03 | 4.35E-02 | 1.79E-01 | ITIH2/ITIH1 | 2 |
| BP | GO:0002449 | lymphocyte mediated immunity | 4/68 | 3.02 | 4.37E-02 | 1.79E-01 | MBL2/IGHV3-7/C9/IGHV1-45 | 4 |
| BP | GO:0009306 | protein secretion | 4/68 | 3.02 | 4.37E-02 | 1.79E-01 | GPLD1/APOE/RBP4/ANG | 4 |
| BP | GO:0035592 | establishment of protein localization to extracellular region | 4/68 | 3.01 | 4.41E-02 | 1.79E-01 | GPLD1/APOE/RBP4/ANG | 4 |
| BP | GO:0043502 | regulation of muscle adaptation | 2/68 | 5.97 | 4.44E-02 | 1.79E-01 | PI16/AGT | 2 |
| BP | GO:0002430 | complement receptor mediated signaling pathway | 1/68 | 21.35 | 4.59E-02 | 1.79E-01 | GPLD1 | 1 |
| BP | GO:0006787 | porphyrin-containing compound catabolic process | 1/68 | 21.35 | 4.59E-02 | 1.79E-01 | AMBP | 1 |
| BP | GO:0010642 | negative regulation of platelet-derived growth factor receptor signaling pathway | 1/68 | 21.35 | 4.59E-02 | 1.79E-01 | APOD | 1 |
| BP | GO:0014874 | response to stimulus involved in regulation of muscle adaptation | 1/68 | 21.35 | 4.59E-02 | 1.79E-01 | AGT | 1 |
| BP | GO:0031115 | negative regulation of microtubule polymerization | 1/68 | 21.35 | 4.59E-02 | 1.79E-01 | CDH5 | 1 |
| BP | GO:0032490 | detection of molecule of bacterial origin | 1/68 | 21.35 | 4.59E-02 | 1.79E-01 | LBP | 1 |
| BP | GO:0033015 | tetrapyrrole catabolic process | 1/68 | 21.35 | 4.59E-02 | 1.79E-01 | AMBP | 1 |
| BP | GO:0034145 | positive regulation of toll-like receptor 4 signaling pathway | 1/68 | 21.35 | 4.59E-02 | 1.79E-01 | LBP | 1 |
| BP | GO:0034333 | adherens junction assembly | 1/68 | 21.35 | 4.59E-02 | 1.79E-01 | ACTB | 1 |
| BP | GO:0034380 | high-density lipoprotein particle assembly | 1/68 | 21.35 | 4.59E-02 | 1.79E-01 | APOE | 1 |
| BP | GO:0036006 | cellular response to macrophage colony-stimulating factor stimulus | 1/68 | 21.35 | 4.59E-02 | 1.79E-01 | CSF1R | 1 |
| BP | GO:0042976 | activation of Janus kinase activity | 1/68 | 21.35 | 4.59E-02 | 1.79E-01 | AGT | 1 |
| BP | GO:0043568 | positive regulation of insulin-like growth factor receptor signaling pathway | 1/68 | 21.35 | 4.59E-02 | 1.79E-01 | IGFBP3 | 1 |
| BP | GO:0051657 | maintenance of organelle location | 1/68 | 21.35 | 4.59E-02 | 1.79E-01 | ALB | 1 |
| BP | GO:0051764 | actin crosslink formation | 1/68 | 21.35 | 4.59E-02 | 1.79E-01 | LCP1 | 1 |
| BP | GO:0061052 | negative regulation of cell growth involved in cardiac muscle cell development | 1/68 | 21.35 | 4.59E-02 | 1.79E-01 | PI16 | 1 |
| BP | GO:0061517 | macrophage proliferation | 1/68 | 21.35 | 4.59E-02 | 1.79E-01 | CSF1R | 1 |
| BP | GO:0071801 | regulation of podosome assembly | 1/68 | 21.35 | 4.59E-02 | 1.79E-01 | LCP1 | 1 |
| BP | GO:1900103 | positive regulation of endoplasmic reticulum unfolded protein response | 1/68 | 21.35 | 4.59E-02 | 1.79E-01 | ERN1 | 1 |
| BP | GO:1900272 | negative regulation of long-term synaptic potentiation | 1/68 | 21.35 | 4.59E-02 | 1.79E-01 | APOE | 1 |
| BP | GO:1902950 | regulation of dendritic spine maintenance | 1/68 | 21.35 | 4.59E-02 | 1.79E-01 | APOE | 1 |
| BP | GO:1905907 | negative regulation of amyloid fibril formation | 1/68 | 21.35 | 4.59E-02 | 1.79E-01 | APOE | 1 |
| BP | GO:0007044 | cell-substrate junction assembly | 2/68 | 5.84 | 4.61E-02 | 1.80E-01 | FN1/APOD | 2 |
| BP | GO:0006644 | phospholipid metabolic process | 4/68 | 2.96 | 4.63E-02 | 1.80E-01 | APOA4/GPLD1/PON1/CETP | 4 |
| BP | GO:0001906 | cell killing | 3/68 | 3.73 | 4.66E-02 | 1.80E-01 | MBL2/CFHR1/C9 | 3 |
| BP | GO:0044242 | cellular lipid catabolic process | 3/68 | 3.73 | 4.66E-02 | 1.80E-01 | APOA4/GPLD1/APOC3 | 3 |
| BP | GO:0002690 | positive regulation of leukocyte chemotaxis | 2/68 | 5.78 | 4.70E-02 | 1.80E-01 | LBP/CSF1R | 2 |
| BP | GO:0014909 | smooth muscle cell migration | 2/68 | 5.78 | 4.70E-02 | 1.80E-01 | IGFBP3/VTN | 2 |
| BP | GO:0034109 | homotypic cell-cell adhesion | 2/68 | 5.78 | 4.70E-02 | 1.80E-01 | ACTB/HBB | 2 |
| BP | GO:0071692 | protein localization to extracellular region | 4/68 | 2.94 | 4.71E-02 | 1.80E-01 | GPLD1/APOE/RBP4/ANG | 4 |
| BP | GO:0060419 | heart growth | 2/68 | 5.72 | 4.79E-02 | 1.83E-01 | PI16/RBP4 | 2 |
| BP | GO:0072330 | monocarboxylic acid biosynthetic process | 3/68 | 3.68 | 4.82E-02 | 1.83E-01 | APOA4/APOC3/PTGDS | 3 |
| BP | GO:0002460 | adaptive immune response based on somatic recombination of immune receptors built from immunoglobulin superfamily domains | 4/68 | 2.92 | 4.82E-02 | 1.83E-01 | MBL2/IGHV3-7/C9/IGHV1-45 | 4 |
| BP | GO:0001818 | negative regulation of cytokine production | 4/68 | 2.91 | 4.86E-02 | 1.84E-01 | FN1/APOD/ORM1/LBP | 4 |
| BP | GO:0002275 | myeloid cell activation involved in immune response | 2/68 | 5.66 | 4.87E-02 | 1.84E-01 | PTGDS/LBP | 2 |
| BP | GO:0000394 | RNA splicing, via endonucleolytic cleavage and ligation | 1/68 | 19.82 | 4.93E-02 | 1.84E-01 | ERN1 | 1 |
| BP | GO:0010745 | negative regulation of macrophage derived foam cell differentiation | 1/68 | 19.82 | 4.93E-02 | 1.84E-01 | CETP | 1 |
| BP | GO:0010867 | positive regulation of triglyceride biosynthetic process | 1/68 | 19.82 | 4.93E-02 | 1.84E-01 | GPLD1 | 1 |
| BP | GO:0032908 | regulation of transforming growth factor beta1 production | 1/68 | 19.82 | 4.93E-02 | 1.84E-01 | AGT | 1 |
| BP | GO:0035641 | locomotory exploration behavior | 1/68 | 19.82 | 4.93E-02 | 1.84E-01 | APOE | 1 |
| BP | GO:1903894 | regulation of IRE1-mediated unfolded protein response | 1/68 | 19.82 | 4.93E-02 | 1.84E-01 | HSPA5 | 1 |
| BP | GO:0003300 | cardiac muscle hypertrophy | 2/68 | 5.61 | 4.96E-02 | 1.84E-01 | PI16/AGT | 2 |
| BP | GO:0046620 | regulation of organ growth | 2/68 | 5.61 | 4.96E-02 | 1.84E-01 | PI16/RBP4 | 2 |
| BP | GO:0007179 | transforming growth factor beta receptor signaling pathway | 3/68 | 3.64 | 4.97E-02 | 1.84E-01 | CDH5/TNXB/HSPA5 | 3 |
| CC | GO:0072562 | blood microparticle | 27/70 | 53.27 | 1.76E-40 | 2.94E-38 | GC/HBD/FN1/ALB/APOA4/ACTB/TF/F13A1/AMBP/ITIH2/IGHV3-7/PON1/LGALS3BP/HBA2/AGT/PLG/HBB/APOE/CFHR1/ITIH1/CPN2/SERPINC1/VTN/ORM1/C9/JCHAIN/HP | 27 |
| CC | GO:0062023 | collagen-containing extracellular matrix | 21/70 | 13.91 | 9.23E-19 | 7.71E-17 | FN1/F12/APOA4/CLEC3B/SERPINA5/F13A1/AMBP/ITIH2/LGALS3BP/TNXB/EFEMP1/AGT/PLG/APOE/APOC3/ITIH1/SERPINA1/SERPINC1/VTN/ORM1/ANG | 21 |
| CC | GO:0005788 | endoplasmic reticulum lumen | 12/70 | 10.89 | 8.82E-10 | 4.91E-08 | FN1/ALB/APOA4/TF/IGFBP3/ITIH2/F10/HSPA5/APOE/SERPINA1/SERPINC1/VTN | 12 |
| CC | GO:0034358 | plasma lipoprotein particle | 6/70 | 47.35 | 2.74E-09 | 9.15E-08 | PCYOX1/APOA4/PON1/APOE/APOC3/CETP | 6 |
| CC | GO:1990777 | lipoprotein particle | 6/70 | 47.35 | 2.74E-09 | 9.15E-08 | PCYOX1/APOA4/PON1/APOE/APOC3/CETP | 6 |
| CC | GO:0032994 | protein-lipid complex | 6/70 | 43.71 | 4.55E-09 | 1.27E-07 | PCYOX1/APOA4/PON1/APOE/APOC3/CETP | 6 |
| CC | GO:0034774 | secretory granule lumen | 11/70 | 9.70 | 1.56E-08 | 3.30E-07 | FN1/ALB/CLEC3B/TF/F13A1/SERPINA4/LGALS3BP/PLG/SERPINA1/ORM1/HP | 11 |
| CC | GO:0060205 | cytoplasmic vesicle lumen | 11/70 | 9.62 | 1.72E-08 | 3.30E-07 | FN1/ALB/CLEC3B/TF/F13A1/SERPINA4/LGALS3BP/PLG/SERPINA1/ORM1/HP | 11 |
| CC | GO:0031983 | vesicle lumen | 11/70 | 9.59 | 1.78E-08 | 3.30E-07 | FN1/ALB/CLEC3B/TF/F13A1/SERPINA4/LGALS3BP/PLG/SERPINA1/ORM1/HP | 11 |
| CC | GO:0034364 | high-density lipoprotein particle | 5/70 | 54.63 | 2.90E-08 | 4.79E-07 | APOA4/PON1/APOE/APOC3/CETP | 5 |
| CC | GO:0031091 | platelet alpha granule | 7/70 | 21.85 | 3.15E-08 | 4.79E-07 | FN1/ALB/SERPINA5/F13A1/PLG/SERPINA1/ORM1 | 7 |
| CC | GO:0031838 | haptoglobin-hemoglobin complex | 4/70 | 103.30 | 4.56E-08 | 6.35E-07 | HBD/HBA2/HBB/HP | 4 |
| CC | GO:0031093 | platelet alpha granule lumen | 6/70 | 25.44 | 1.29E-07 | 1.65E-06 | FN1/ALB/F13A1/PLG/SERPINA1/ORM1 | 6 |
| CC | GO:0034361 | very-low-density lipoprotein particle | 4/70 | 56.82 | 6.54E-07 | 7.28E-06 | PCYOX1/APOA4/APOE/APOC3 | 4 |
| CC | GO:0034385 | triglyceride-rich plasma lipoprotein particle | 4/70 | 56.82 | 6.54E-07 | 7.28E-06 | PCYOX1/APOA4/APOE/APOC3 | 4 |
| CC | GO:0071682 | endocytic vesicle lumen | 4/70 | 49.41 | 1.19E-06 | 1.24E-05 | HBA2/HBB/APOE/HP | 4 |
| CC | GO:0005833 | hemoglobin complex | 3/70 | 71.02 | 8.98E-06 | 8.33E-05 | HBD/HBA2/HBB | 3 |
| CC | GO:0042627 | chylomicron | 3/70 | 71.02 | 8.98E-06 | 8.33E-05 | APOA4/APOE/APOC3 | 3 |
| CC | GO:0031089 | platelet dense granule lumen | 3/70 | 60.88 | 1.48E-05 | 1.30E-04 | CLEC3B/SERPINA4/LGALS3BP | 3 |
| CC | GO:0005791 | rough endoplasmic reticulum | 4/70 | 20.29 | 4.50E-05 | 3.76E-04 | F12/HGFAC/PTGDS/VTN | 4 |
| CC | GO:0042827 | platelet dense granule | 3/70 | 40.58 | 5.31E-05 | 4.22E-04 | CLEC3B/SERPINA4/LGALS3BP | 3 |
| CC | GO:0071745 | IgA immunoglobulin complex | 2/70 | 56.82 | 5.40E-04 | 4.10E-03 | PIGR/JCHAIN | 2 |
| CC | GO:1904090 | peptidase inhibitor complex | 2/70 | 51.65 | 6.58E-04 | 4.78E-03 | SERPINA5/VTN | 2 |
| CC | GO:0042571 | immunoglobulin complex, circulating | 2/70 | 47.35 | 7.88E-04 | 5.48E-03 | PIGR/JCHAIN | 2 |
| CC | GO:1904724 | tertiary granule lumen | 3/70 | 15.50 | 9.61E-04 | 6.42E-03 | HBB/ORM1/HP | 3 |
| CC | GO:0098637 | protein complex involved in cell-matrix adhesion | 2/70 | 33.42 | 1.61E-03 | 1.03E-02 | TNXB/VTN | 2 |
| CC | GO:0019814 | immunoglobulin complex | 4/70 | 6.89 | 2.74E-03 | 1.69E-02 | PIGR/IGHV3-7/JCHAIN/IGHV1-45 | 4 |
| CC | GO:0005604 | basement membrane | 3/70 | 9.47 | 3.94E-03 | 2.35E-02 | FN1/VTN/ANG | 3 |
| CC | GO:0030139 | endocytic vesicle | 5/70 | 4.08 | 7.62E-03 | 4.39E-02 | TF/HBA2/HBB/APOE/HP | 5 |
| CC | GO:0070160 | tight junction | 3/70 | 6.51 | 1.11E-02 | 6.15E-02 | ACTB/CDH5/CLDN10 | 3 |
| CC | GO:0009897 | external side of plasma membrane | 5/70 | 3.67 | 1.17E-02 | 6.15E-02 | SERPINA5/MBL2/CDH5/F10/PLG | 5 |
| CC | GO:0005793 | endoplasmic reticulum-Golgi intermediate compartment | 3/70 | 6.36 | 1.18E-02 | 6.15E-02 | FN1/HSPA5/SERPINA1 | 3 |
| CC | GO:0043296 | apical junction complex | 3/70 | 5.68 | 1.59E-02 | 8.06E-02 | ACTB/CDH5/CLDN10 | 3 |
| CC | GO:0098636 | protein complex involved in cell adhesion | 2/70 | 9.97 | 1.72E-02 | 8.45E-02 | TNXB/VTN | 2 |
| CC | GO:0070820 | tertiary granule | 3/70 | 5.20 | 2.01E-02 | 9.35E-02 | HBB/ORM1/HP | 3 |
| CC | GO:0035580 | specific granule lumen | 2/70 | 9.16 | 2.02E-02 | 9.35E-02 | ORM1/HP | 2 |
| CC | GO:0005771 | multivesicular body | 2/70 | 8.36 | 2.40E-02 | 1.06E-01 | MBL2/APOE | 2 |
| CC | GO:0031965 | nuclear membrane | 4/70 | 3.65 | 2.40E-02 | 1.06E-01 | AMBP/CDH5/PTGDS/ERN1 | 4 |
| CC | GO:0030669 | clathrin-coated endocytic vesicle membrane | 2/70 | 7.68 | 2.80E-02 | 1.20E-01 | TF/APOE | 2 |
| CC | GO:0099571 | postsynaptic cytoskeleton | 1/70 | 28.41 | 3.47E-02 | 1.41E-01 | ACTB | 1 |
| CC | GO:0140092 | bBAF complex | 1/70 | 28.41 | 3.47E-02 | 1.41E-01 | ACTB | 1 |
| CC | GO:0031094 | platelet dense tubular network | 1/70 | 25.83 | 3.81E-02 | 1.44E-01 | SERPINA5 | 1 |
| CC | GO:0034663 | endoplasmic reticulum chaperone complex | 1/70 | 25.83 | 3.81E-02 | 1.44E-01 | HSPA5 | 1 |
| CC | GO:1905370 | serine-type endopeptidase complex | 1/70 | 25.83 | 3.81E-02 | 1.44E-01 | MBL2 | 1 |
| CC | GO:0005797 | Golgi medial cisterna | 1/70 | 23.67 | 4.14E-02 | 1.48E-01 | MAN2A1 | 1 |
| CC | GO:0034362 | low-density lipoprotein particle | 1/70 | 23.67 | 4.14E-02 | 1.48E-01 | APOE | 1 |
| CC | GO:0045334 | clathrin-coated endocytic vesicle | 2/70 | 6.18 | 4.17E-02 | 1.48E-01 | TF/APOE | 2 |
| CC | GO:1905286 | serine-type peptidase complex | 1/70 | 21.85 | 4.48E-02 | 1.56E-01 | MBL2 | 1 |
| CC | GO:0035060 | brahma complex | 1/70 | 20.29 | 4.82E-02 | 1.58E-01 | ACTB | 1 |
| CC | GO:0071564 | npBAF complex | 1/70 | 20.29 | 4.82E-02 | 1.58E-01 | ACTB | 1 |
| CC | GO:0140288 | GBAF complex | 1/70 | 20.29 | 4.82E-02 | 1.58E-01 | ACTB | 1 |
| MF | GO:0005539 | glycosaminoglycan binding | 12/68 | 13.60 | 6.63E-11 | 1.35E-08 | FN1/CLEC3B/SERPINA5/ITIH2/HABP2/TNXB/APOE/ITIH1/SERPINC1/VTN/ANG/JCHAIN | 12 |
| MF | GO:0061134 | peptidase regulator activity | 11/68 | 13.12 | 6.49E-10 | 4.10E-08 | FN1/FETUB/PI16/SERPINA5/AMBP/SERPINA4/ITIH2/AGT/ITIH1/SERPINA1/SERPINC1 | 11 |
| MF | GO:0016209 | antioxidant activity | 8/68 | 26.22 | 6.92E-10 | 4.10E-08 | HBD/ALB/APOA4/GPX3/HBA2/HBB/APOE/HP | 8 |
| MF | GO:0030414 | peptidase inhibitor activity | 10/68 | 15.54 | 8.04E-10 | 4.10E-08 | FETUB/PI16/SERPINA5/AMBP/SERPINA4/ITIH2/AGT/ITIH1/SERPINA1/SERPINC1 | 10 |
| MF | GO:0004867 | serine-type endopeptidase inhibitor activity | 8/68 | 21.54 | 3.37E-09 | 1.37E-07 | SERPINA5/AMBP/SERPINA4/ITIH2/AGT/ITIH1/SERPINA1/SERPINC1 | 8 |
| MF | GO:0004866 | endopeptidase inhibitor activity | 9/68 | 14.57 | 1.06E-08 | 3.59E-07 | FETUB/SERPINA5/AMBP/SERPINA4/ITIH2/AGT/ITIH1/SERPINA1/SERPINC1 | 9 |
| MF | GO:0061135 | endopeptidase regulator activity | 9/68 | 13.30 | 2.33E-08 | 6.80E-07 | FETUB/SERPINA5/AMBP/SERPINA4/ITIH2/AGT/ITIH1/SERPINA1/SERPINC1 | 9 |
| MF | GO:0004857 | enzyme inhibitor activity | 11/68 | 7.75 | 1.50E-07 | 3.82E-06 | FETUB/PI16/SERPINA5/AMBP/SERPINA4/ITIH2/AGT/APOC3/ITIH1/SERPINA1/SERPINC1 | 11 |
| MF | GO:0008201 | heparin binding | 8/68 | 12.51 | 2.39E-07 | 5.41E-06 | FN1/CLEC3B/SERPINA5/TNXB/APOE/SERPINC1/VTN/ANG | 8 |
| MF | GO:1901681 | sulfur compound binding | 9/68 | 8.90 | 7.13E-07 | 1.46E-05 | FN1/CLEC3B/TF/SERPINA5/TNXB/APOE/SERPINC1/VTN/ANG | 9 |
| MF | GO:0004252 | serine-type endopeptidase activity | 7/68 | 10.94 | 3.46E-06 | 6.41E-05 | F12/HABP2/F10/PLG/MASP1/HGFAC/HP | 7 |
| MF | GO:0031210 | phosphatidylcholine binding | 4/68 | 36.27 | 4.26E-06 | 7.24E-05 | APOA4/SERPINA5/CETP/JCHAIN | 4 |
| MF | GO:0031720 | haptoglobin binding | 3/68 | 81.60 | 5.60E-06 | 8.79E-05 | HBD/HBA2/HBB | 3 |
| MF | GO:0008236 | serine-type peptidase activity | 7/68 | 9.92 | 6.61E-06 | 9.63E-05 | F12/HABP2/F10/PLG/MASP1/HGFAC/HP | 7 |
| MF | GO:0017171 | serine hydrolase activity | 7/68 | 9.71 | 7.57E-06 | 1.03E-04 | F12/HABP2/F10/PLG/MASP1/HGFAC/HP | 7 |
| MF | GO:0043177 | organic acid binding | 7/68 | 9.20 | 1.08E-05 | 1.38E-04 | HBD/ALB/SERPINA5/AMBP/HBA2/HBB/PTGDS | 7 |
| MF | GO:0019825 | oxygen binding | 4/68 | 27.90 | 1.25E-05 | 1.50E-04 | HBD/ALB/HBA2/HBB | 4 |
| MF | GO:0002020 | protease binding | 6/68 | 11.49 | 1.36E-05 | 1.54E-04 | FN1/CHL1/SERPINA5/PLG/SERPINA1/SERPINC1 | 6 |
| MF | GO:0005344 | oxygen carrier activity | 3/68 | 58.29 | 1.68E-05 | 1.80E-04 | HBD/HBA2/HBB | 3 |
| MF | GO:0050997 | quaternary ammonium group binding | 4/68 | 20.15 | 4.60E-05 | 4.69E-04 | APOA4/SERPINA5/CETP/JCHAIN | 4 |
| MF | GO:0004601 | peroxidase activity | 4/68 | 19.78 | 4.95E-05 | 4.81E-04 | HBD/GPX3/HBA2/HBB | 4 |
| MF | GO:0016684 | oxidoreductase activity, acting on peroxide as acceptor | 4/68 | 19.09 | 5.70E-05 | 5.28E-04 | HBD/GPX3/HBA2/HBB | 4 |
| MF | GO:0030246 | carbohydrate binding | 7/68 | 6.97 | 6.35E-05 | 5.63E-04 | CLEC3B/MBL2/AMBP/ATRN/MAN2A1/ITIH1/VTN | 7 |
| MF | GO:0120020 | cholesterol transfer activity | 3/68 | 37.09 | 6.96E-05 | 5.92E-04 | APOA4/APOE/CETP | 3 |
| MF | GO:0120015 | sterol transfer activity | 3/68 | 35.48 | 7.99E-05 | 6.52E-04 | APOA4/APOE/CETP | 3 |
| MF | GO:0015248 | sterol transporter activity | 3/68 | 22.67 | 3.11E-04 | 2.44E-03 | APOA4/APOE/CETP | 3 |
| MF | GO:0043178 | alcohol binding | 4/68 | 12.22 | 3.22E-04 | 2.44E-03 | APOD/APOC3/CETP/RBP4 | 4 |
| MF | GO:0005501 | retinoid binding | 3/68 | 21.47 | 3.66E-04 | 2.57E-03 | SERPINA5/PTGDS/RBP4 | 3 |
| MF | GO:0019840 | isoprenoid binding | 3/68 | 21.47 | 3.66E-04 | 2.57E-03 | SERPINA5/PTGDS/RBP4 | 3 |
| MF | GO:0005543 | phospholipid binding | 8/68 | 4.50 | 3.87E-04 | 2.63E-03 | APOA4/SERPINA5/PON1/F10/APOE/APOC3/CETP/JCHAIN | 8 |
| MF | GO:0140104 | molecular carrier activity | 4/68 | 11.45 | 4.14E-04 | 2.72E-03 | HBD/TF/HBA2/HBB | 4 |
| MF | GO:0005319 | lipid transporter activity | 5/68 | 7.91 | 4.26E-04 | 2.72E-03 | APOA4/APOD/APOE/CETP/RBP4 | 5 |
| MF | GO:0005496 | steroid binding | 4/68 | 10.67 | 5.42E-04 | 3.33E-03 | GC/APOD/APOC3/CETP | 4 |
| MF | GO:0004175 | endopeptidase activity | 7/68 | 4.89 | 5.55E-04 | 3.33E-03 | F12/HABP2/F10/PLG/MASP1/HGFAC/HP | 7 |
| MF | GO:0015485 | cholesterol binding | 3/68 | 15.69 | 9.23E-04 | 5.38E-03 | APOD/APOC3/CETP | 3 |
| MF | GO:0120013 | lipid transfer activity | 3/68 | 15.11 | 1.03E-03 | 5.84E-03 | APOA4/APOE/CETP | 3 |
| MF | GO:0051087 | protein-folding chaperone binding | 4/68 | 8.18 | 1.46E-03 | 8.04E-03 | ALB/HSPA5/PLG/ERN1 | 4 |
| MF | GO:0032934 | sterol binding | 3/68 | 13.16 | 1.54E-03 | 8.26E-03 | APOD/APOC3/CETP | 3 |
| MF | GO:0005507 | copper ion binding | 3/68 | 12.95 | 1.61E-03 | 8.43E-03 | ALB/APOA4/ANG | 3 |
| MF | GO:0020037 | heme binding | 4/68 | 7.72 | 1.81E-03 | 9.21E-03 | HBD/AMBP/HBA2/HBB | 4 |
| MF | GO:0005518 | collagen binding | 3/68 | 12.00 | 2.01E-03 | 9.99E-03 | FN1/TNXB/VTN | 3 |
| MF | GO:0046906 | tetrapyrrole binding | 4/68 | 7.21 | 2.32E-03 | 1.13E-02 | HBD/AMBP/HBA2/HBB | 4 |
| MF | GO:0051787 | misfolded protein binding | 2/68 | 27.20 | 2.42E-03 | 1.15E-02 | F12/HSPA5 | 2 |
| MF | GO:0005178 | integrin binding | 4/68 | 7.06 | 2.49E-03 | 1.15E-02 | FN1/TNXB/VTN/LCP1 | 4 |
| MF | GO:0033293 | monocarboxylic acid binding | 3/68 | 10.33 | 3.08E-03 | 1.40E-02 | ALB/SERPINA5/PTGDS | 3 |
| MF | GO:0005201 | extracellular matrix structural constituent | 4/68 | 6.55 | 3.26E-03 | 1.45E-02 | FN1/TNXB/EFEMP1/VTN | 4 |
| MF | GO:0005540 | hyaluronic acid binding | 2/68 | 22.67 | 3.49E-03 | 1.48E-02 | ITIH2/ITIH1 | 2 |
| MF | GO:0019865 | immunoglobulin binding | 2/68 | 22.67 | 3.49E-03 | 1.48E-02 | AMBP/JCHAIN | 2 |
| MF | GO:0003823 | antigen binding | 4/68 | 6.11 | 4.18E-03 | 1.74E-02 | IGHV3-7/PLG/JCHAIN/IGHV1-45 | 4 |
| MF | GO:0005044 | scavenger receptor activity | 2/68 | 20.15 | 4.41E-03 | 1.80E-02 | LGALS3BP/VTN | 2 |
| MF | GO:0070325 | lipoprotein particle receptor binding | 2/68 | 18.76 | 5.07E-03 | 2.03E-02 | APOE/APOC3 | 2 |
| MF | GO:0031406 | carboxylic acid binding | 4/68 | 5.58 | 5.77E-03 | 2.26E-02 | ALB/SERPINA5/AMBP/PTGDS | 4 |
| MF | GO:0090482 | vitamin transmembrane transporter activity | 2/68 | 15.54 | 7.33E-03 | 2.82E-02 | GC/RBP4 | 2 |
| MF | GO:0043394 | proteoglycan binding | 2/68 | 15.11 | 7.74E-03 | 2.92E-02 | FN1/APOE | 2 |
| MF | GO:0048156 | tau protein binding | 2/68 | 12.65 | 1.09E-02 | 4.05E-02 | ACTB/APOE | 2 |
| MF | GO:0005504 | fatty acid binding | 2/68 | 11.33 | 1.35E-02 | 4.91E-02 | ALB/PTGDS | 2 |
| MF | GO:0070851 | growth factor receptor binding | 3/68 | 5.91 | 1.43E-02 | 5.12E-02 | CDH5/EFEMP1/ERN1 | 3 |
| MF | GO:0019842 | vitamin binding | 3/68 | 5.44 | 1.78E-02 | 6.28E-02 | GC/ALB/RBP4 | 3 |
| MF | GO:0004714 | transmembrane receptor protein tyrosine kinase activity | 2/68 | 9.07 | 2.06E-02 | 7.11E-02 | EFEMP1/CSF1R | 2 |
| MF | GO:0038024 | cargo receptor activity | 2/68 | 8.50 | 2.32E-02 | 7.88E-02 | LGALS3BP/VTN | 2 |
| MF | GO:0004521 | RNA endonuclease activity | 2/68 | 7.35 | 3.04E-02 | 1.02E-01 | ANG/ERN1 | 2 |
| MF | GO:0019199 | transmembrane receptor protein kinase activity | 2/68 | 6.89 | 3.42E-02 | 1.07E-01 | EFEMP1/CSF1R | 2 |
| MF | GO:0048306 | calcium-dependent protein binding | 2/68 | 6.89 | 3.42E-02 | 1.07E-01 | MBL2/MASP1 | 2 |
| MF | GO:0015643 | toxic substance binding | 1/68 | 27.20 | 3.62E-02 | 1.07E-01 | ALB | 1 |
| MF | GO:0015924 | mannosyl-oligosaccharide mannosidase activity | 1/68 | 27.20 | 3.62E-02 | 1.07E-01 | MAN2A1 | 1 |
| MF | GO:0030957 | Tat protein binding | 1/68 | 27.20 | 3.62E-02 | 1.07E-01 | ACTB | 1 |
| MF | GO:0071723 | lipopeptide binding | 1/68 | 27.20 | 3.62E-02 | 1.07E-01 | LBP | 1 |
| MF | GO:0098973 | structural constituent of postsynaptic actin cytoskeleton | 1/68 | 27.20 | 3.62E-02 | 1.07E-01 | ACTB | 1 |
| MF | GO:0001851 | complement component C3b binding | 1/68 | 24.73 | 3.97E-02 | 1.07E-01 | CFHR1 | 1 |
| MF | GO:0008199 | ferric iron binding | 1/68 | 24.73 | 3.97E-02 | 1.07E-01 | TF | 1 |
| MF | GO:0016670 | oxidoreductase activity, acting on a sulfur group of donors, oxygen as acceptor | 1/68 | 24.73 | 3.97E-02 | 1.07E-01 | PCYOX1 | 1 |
| MF | GO:0016857 | racemase and epimerase activity, acting on carbohydrates and derivatives | 1/68 | 24.73 | 3.97E-02 | 1.07E-01 | GALE | 1 |
| MF | GO:0019855 | calcium channel inhibitor activity | 1/68 | 24.73 | 3.97E-02 | 1.07E-01 | AMBP | 1 |
| MF | GO:0050998 | nitric-oxide synthase binding | 1/68 | 24.73 | 3.97E-02 | 1.07E-01 | ACTB | 1 |
| MF | GO:0070700 | BMP receptor binding | 1/68 | 24.73 | 3.97E-02 | 1.07E-01 | CDH5 | 1 |
| MF | GO:1990459 | transferrin receptor binding | 1/68 | 24.73 | 3.97E-02 | 1.07E-01 | TF | 1 |
| MF | GO:0005527 | macrolide binding | 1/68 | 22.67 | 4.32E-02 | 1.15E-01 | ALB | 1 |
| MF | GO:0005172 | vascular endothelial growth factor receptor binding | 1/68 | 20.92 | 4.68E-02 | 1.21E-01 | CDH5 | 1 |
| MF | GO:0031994 | insulin-like growth factor I binding | 1/68 | 20.92 | 4.68E-02 | 1.21E-01 | IGFBP3 | 1 |
| Organismal Systems | hsa04610 | Complement and coagulation cascades | 13/42 | 31.18 | 7.33E-17 | 7.84E-15 | F12/SERPINA5/F13A1/MBL2/F10/F13B/PLG/CFHR1/MASP1/SERPINA1/SERPINC1/VTN/C9 | 13 |
| Organismal Systems | hsa04979 | Cholesterol metabolism | 4/42 | 16.55 | 9.26E-05 | 4.95E-03 | APOA4/APOE/APOC3/CETP | 4 |
| Human Diseases | hsa05171 | Coronavirus disease - COVID-19 | 5/42 | 4.43 | 5.06E-03 | 1.41E-01 | F13A1/MBL2/F13B/MASP1/C9 | 5 |
| Organismal Systems | hsa04918 | Thyroid hormone synthesis | 3/42 | 8.44 | 5.27E-03 | 1.41E-01 | ALB/GPX3/HSPA5 | 3 |
| Organismal Systems | hsa04977 | Vitamin digestion and absorption | 2/42 | 16.24 | 6.63E-03 | 1.42E-01 | APOA4/BTD | 2 |
| Environmental Information Processing | hsa04512 | ECM-receptor interaction | 3/42 | 7.11 | 8.47E-03 | 1.51E-01 | FN1/TNXB/VTN | 3 |
| Human Diseases | hsa05150 | Staphylococcus aureus infection | 3/42 | 6.21 | 1.23E-02 | 1.76E-01 | MBL2/PLG/MASP1 | 3 |
| Human Diseases | hsa05143 | African trypanosomiasis | 2/42 | 11.41 | 1.31E-02 | 1.76E-01 | HBA2/HBB | 2 |
| Cellular Processes | hsa04510 | Focal adhesion | 4/42 | 4.16 | 1.51E-02 | 1.80E-01 | FN1/ACTB/TNXB/VTN | 4 |
| Organismal Systems | hsa04670 | Leukocyte transendothelial migration | 3/42 | 5.46 | 1.73E-02 | 1.81E-01 | ACTB/CDH5/CLDN10 | 3 |
| Human Diseases | hsa05417 | Lipid and atherosclerosis | 4/42 | 3.91 | 1.86E-02 | 1.81E-01 | APOA4/HSPA5/LBP/ERN1 | 4 |
| Human Diseases | hsa05144 | Malaria | 2/42 | 8.44 | 2.33E-02 | 2.07E-01 | HBA2/HBB | 2 |
| Human Diseases | hsa05014 | Amyotrophic lateral sclerosis | 5/42 | 2.84 | 2.98E-02 | 2.45E-01 | ACTB/GPX3/HSPA5/ANG/ERN1 | 5 |

Table S13: Enrichment analysis results of common DEGs between the TB6 vs. TB0 group and the TB0 vs. HC group

| Category | ID | Description | GeneRatio | FoldEnrichment | pvalue | p.adjust | geneID | Count |
| --- | --- | --- | --- | --- | --- | --- | --- | --- |
| BP | GO:0006869 | lipid transport | 7/31 | 9.41 | 7.00E-06 | 4.53E-03 | LBP/SERPINA5/APOC3/APOD/APOA4/RBP4/PON1 | 7 |
| BP | GO:0001895 | retina homeostasis | 4/31 | 29.34 | 1.00E-05 | 4.53E-03 | JCHAIN/ALB/ACTB/TF | 4 |
| BP | GO:0042744 | hydrogen peroxide catabolic process | 3/31 | 58.91 | 1.75E-05 | 4.55E-03 | APOA4/GPX3/HBD | 3 |
| BP | GO:0098869 | cellular oxidant detoxification | 4/31 | 24.59 | 2.01E-05 | 4.55E-03 | ALB/APOA4/GPX3/HBD | 4 |
| BP | GO:0002526 | acute inflammatory response | 4/31 | 22.76 | 2.73E-05 | 4.95E-03 | LBP/ORM1/F12/FN1 | 4 |
| BP | GO:1990748 | cellular detoxification | 4/31 | 20.46 | 4.15E-05 | 6.26E-03 | ALB/APOA4/GPX3/HBD | 4 |
| BP | GO:0097237 | cellular response to toxic substance | 4/31 | 19.02 | 5.51E-05 | 6.60E-03 | ALB/APOA4/GPX3/HBD | 4 |
| BP | GO:0009636 | response to toxic substance | 5/31 | 11.80 | 5.84E-05 | 6.60E-03 | ALB/APOA4/GPX3/PON1/HBD | 5 |
| BP | GO:0006953 | acute-phase response | 3/31 | 38.04 | 6.60E-05 | 6.64E-03 | LBP/ORM1/FN1 | 3 |
| BP | GO:0001894 | tissue homeostasis | 5/31 | 10.91 | 8.45E-05 | 6.95E-03 | JCHAIN/ALB/ACTB/RBP4/TF | 5 |
| BP | GO:0060249 | anatomical structure homeostasis | 5/31 | 10.91 | 8.45E-05 | 6.95E-03 | JCHAIN/ALB/ACTB/RBP4/TF | 5 |
| BP | GO:0042743 | hydrogen peroxide metabolic process | 3/31 | 33.82 | 9.41E-05 | 7.10E-03 | APOA4/GPX3/HBD | 3 |
| BP | GO:0014012 | peripheral nervous system axon regeneration | 2/31 | 121.74 | 1.17E-04 | 8.07E-03 | APOD/APOA4 | 2 |
| BP | GO:0098754 | detoxification | 4/31 | 15.41 | 1.25E-04 | 8.07E-03 | ALB/APOA4/GPX3/HBD | 4 |
| BP | GO:0015850 | organic hydroxy compound transport | 5/31 | 9.88 | 1.34E-04 | 8.11E-03 | APOC3/ACTB/APOA4/RBP4/PON1 | 5 |
| BP | GO:0034370 | triglyceride-rich lipoprotein particle remodeling | 2/31 | 101.45 | 1.71E-04 | 9.08E-03 | APOC3/APOA4 | 2 |
| BP | GO:0034372 | very-low-density lipoprotein particle remodeling | 2/31 | 101.45 | 1.71E-04 | 9.08E-03 | APOC3/APOA4 | 2 |
| BP | GO:0010896 | regulation of triglyceride catabolic process | 2/31 | 93.65 | 2.01E-04 | 1.01E-02 | APOC3/APOA4 | 2 |
| BP | GO:0033344 | cholesterol efflux | 3/31 | 25.02 | 2.31E-04 | 1.06E-02 | APOC3/APOA4/PON1 | 3 |
| BP | GO:0033700 | phospholipid efflux | 2/31 | 86.96 | 2.35E-04 | 1.06E-02 | APOC3/APOA4 | 2 |
| BP | GO:0032371 | regulation of sterol transport | 3/31 | 22.83 | 3.03E-04 | 1.12E-02 | APOC3/APOA4/PON1 | 3 |
| BP | GO:0032374 | regulation of cholesterol transport | 3/31 | 22.83 | 3.03E-04 | 1.12E-02 | APOC3/APOA4/PON1 | 3 |
| BP | GO:0034375 | high-density lipoprotein particle remodeling | 2/31 | 76.09 | 3.09E-04 | 1.12E-02 | APOC3/APOA4 | 2 |
| BP | GO:0051004 | regulation of lipoprotein lipase activity | 2/31 | 76.09 | 3.09E-04 | 1.12E-02 | APOC3/APOA4 | 2 |
| BP | GO:0072378 | blood coagulation, fibrin clot formation | 2/31 | 76.09 | 3.09E-04 | 1.12E-02 | F13A1/F12 | 2 |
| BP | GO:0010755 | regulation of plasminogen activation | 2/31 | 67.63 | 3.93E-04 | 1.27E-02 | F12/CLEC3B | 2 |
| BP | GO:0033194 | response to hydroperoxide | 2/31 | 67.63 | 3.93E-04 | 1.27E-02 | APOA4/GPX3 | 2 |
| BP | GO:0060263 | regulation of respiratory burst | 2/31 | 67.63 | 3.93E-04 | 1.27E-02 | JCHAIN/LBP | 2 |
| BP | GO:0051346 | negative regulation of hydrolase activity | 4/31 | 11.17 | 4.27E-04 | 1.32E-02 | FETUB/SERPINA5/APOC3/SERPINA4 | 4 |
| BP | GO:0072376 | protein activation cascade | 2/31 | 64.07 | 4.39E-04 | 1.32E-02 | F13A1/F12 | 2 |
| BP | GO:0043691 | reverse cholesterol transport | 2/31 | 60.87 | 4.87E-04 | 1.42E-02 | APOC3/APOA4 | 2 |
| BP | GO:0033002 | muscle cell proliferation | 4/31 | 9.86 | 6.83E-04 | 1.89E-02 | ERN1/APOD/RBP4/IGFBP3 | 4 |
| BP | GO:0006641 | triglyceride metabolic process | 3/31 | 17.23 | 6.91E-04 | 1.89E-02 | APOC3/APOA4/TNXB | 3 |
| BP | GO:0010954 | positive regulation of protein processing | 2/31 | 48.70 | 7.65E-04 | 2.04E-02 | F12/CLEC3B | 2 |
| BP | GO:0031639 | plasminogen activation | 2/31 | 45.09 | 8.94E-04 | 2.12E-02 | F12/CLEC3B | 2 |
| BP | GO:0034377 | plasma lipoprotein particle assembly | 2/31 | 45.09 | 8.94E-04 | 2.12E-02 | APOC3/APOA4 | 2 |
| BP | GO:1903319 | positive regulation of protein maturation | 2/31 | 45.09 | 8.94E-04 | 2.12E-02 | F12/CLEC3B | 2 |
| BP | GO:0018149 | peptide cross-linking | 2/31 | 43.48 | 9.61E-04 | 2.12E-02 | F13A1/FN1 | 2 |
| BP | GO:0019433 | triglyceride catabolic process | 2/31 | 43.48 | 9.61E-04 | 2.12E-02 | APOC3/APOA4 | 2 |
| BP | GO:0055022 | negative regulation of cardiac muscle tissue growth | 2/31 | 43.48 | 9.61E-04 | 2.12E-02 | PI16/RBP4 | 2 |
| BP | GO:0061117 | negative regulation of heart growth | 2/31 | 43.48 | 9.61E-04 | 2.12E-02 | PI16/RBP4 | 2 |
| BP | GO:0065005 | protein-lipid complex assembly | 2/31 | 40.58 | 1.10E-03 | 2.38E-02 | APOC3/APOA4 | 2 |
| BP | GO:0010951 | negative regulation of endopeptidase activity | 3/31 | 14.27 | 1.19E-03 | 2.45E-02 | FETUB/SERPINA5/SERPINA4 | 3 |
| BP | GO:0030301 | cholesterol transport | 3/31 | 14.27 | 1.19E-03 | 2.45E-02 | APOC3/APOA4/PON1 | 3 |
| BP | GO:0034368 | protein-lipid complex remodeling | 2/31 | 36.89 | 1.34E-03 | 2.57E-02 | APOC3/APOA4 | 2 |
| BP | GO:0034369 | plasma lipoprotein particle remodeling | 2/31 | 36.89 | 1.34E-03 | 2.57E-02 | APOC3/APOA4 | 2 |
| BP | GO:0006639 | acylglycerol metabolic process | 3/31 | 13.63 | 1.36E-03 | 2.57E-02 | APOC3/APOA4/TNXB | 3 |
| BP | GO:0006638 | neutral lipid metabolic process | 3/31 | 13.53 | 1.39E-03 | 2.57E-02 | APOC3/APOA4/TNXB | 3 |
| BP | GO:0042157 | lipoprotein metabolic process | 3/31 | 13.53 | 1.39E-03 | 2.57E-02 | APOC3/APOD/APOA4 | 3 |
| BP | GO:0010466 | negative regulation of peptidase activity | 3/31 | 13.33 | 1.45E-03 | 2.62E-02 | FETUB/SERPINA5/SERPINA4 | 3 |
| BP | GO:0034367 | protein-containing complex remodeling | 2/31 | 34.78 | 1.50E-03 | 2.67E-02 | APOC3/APOA4 | 2 |
| BP | GO:0015918 | sterol transport | 3/31 | 12.95 | 1.57E-03 | 2.74E-02 | APOC3/APOA4/PON1 | 3 |
| BP | GO:0046621 | negative regulation of organ growth | 2/31 | 32.90 | 1.68E-03 | 2.87E-02 | PI16/RBP4 | 2 |
| BP | GO:0046461 | neutral lipid catabolic process | 2/31 | 32.04 | 1.77E-03 | 2.91E-02 | APOC3/APOA4 | 2 |
| BP | GO:0046464 | acylglycerol catabolic process | 2/31 | 32.04 | 1.77E-03 | 2.91E-02 | APOC3/APOA4 | 2 |
| BP | GO:0045730 | respiratory burst | 2/31 | 30.44 | 1.96E-03 | 3.16E-02 | JCHAIN/LBP | 2 |
| BP | GO:0032368 | regulation of lipid transport | 3/31 | 11.94 | 1.99E-03 | 3.16E-02 | APOC3/APOA4/PON1 | 3 |
| BP | GO:0071634 | regulation of transforming growth factor beta production | 2/31 | 28.99 | 2.16E-03 | 3.36E-02 | TNXB/FN1 | 2 |
| BP | GO:0045663 | positive regulation of myoblast differentiation | 2/31 | 28.31 | 2.26E-03 | 3.36E-02 | ACTB/IGFBP3 | 2 |
| BP | GO:0055090 | acylglycerol homeostasis | 2/31 | 28.31 | 2.26E-03 | 3.36E-02 | APOC3/APOA4 | 2 |
| BP | GO:0070328 | triglyceride homeostasis | 2/31 | 28.31 | 2.26E-03 | 3.36E-02 | APOC3/APOA4 | 2 |
| BP | GO:0090207 | regulation of triglyceride metabolic process | 2/31 | 27.67 | 2.37E-03 | 3.45E-02 | APOC3/APOA4 | 2 |
| BP | GO:0071604 | transforming growth factor beta production | 2/31 | 27.05 | 2.47E-03 | 3.55E-02 | TNXB/FN1 | 2 |
| BP | GO:0048660 | regulation of smooth muscle cell proliferation | 3/31 | 10.68 | 2.73E-03 | 3.86E-02 | ERN1/APOD/IGFBP3 | 3 |
| BP | GO:0048659 | smooth muscle cell proliferation | 3/31 | 10.44 | 2.91E-03 | 4.05E-02 | ERN1/APOD/IGFBP3 | 3 |
| BP | GO:0071827 | plasma lipoprotein particle organization | 2/31 | 24.35 | 3.05E-03 | 4.18E-02 | APOC3/APOA4 | 2 |
| BP | GO:0031103 | axon regeneration | 2/31 | 23.41 | 3.29E-03 | 4.39E-02 | APOD/APOA4 | 2 |
| BP | GO:1905952 | regulation of lipid localization | 3/31 | 9.98 | 3.30E-03 | 4.39E-02 | APOC3/APOA4/PON1 | 3 |
| BP | GO:0001818 | negative regulation of cytokine production | 4/31 | 6.39 | 3.34E-03 | 4.39E-02 | LBP/ORM1/APOD/FN1 | 4 |
| BP | GO:0071825 | protein-lipid complex organization | 2/31 | 22.97 | 3.42E-03 | 4.42E-02 | APOC3/APOA4 | 2 |
| BP | GO:0046486 | glycerolipid metabolic process | 4/31 | 6.24 | 3.64E-03 | 4.62E-02 | APOC3/APOA4/PON1/TNXB | 4 |
| BP | GO:0042304 | regulation of fatty acid biosynthetic process | 2/31 | 22.13 | 3.67E-03 | 4.62E-02 | APOC3/APOA4 | 2 |
| BP | GO:0031099 | regeneration | 3/31 | 9.56 | 3.72E-03 | 4.62E-02 | LCP1/APOD/APOA4 | 3 |
| BP | GO:0006006 | glucose metabolic process | 3/31 | 9.46 | 3.84E-03 | 4.69E-02 | APOD/RBP4/IGFBP3 | 3 |
| BP | GO:0006979 | response to oxidative stress | 4/31 | 6.09 | 3.98E-03 | 4.80E-02 | ERN1/APOD/APOA4/GPX3 | 4 |
| BP | GO:0031102 | neuron projection regeneration | 2/31 | 20.99 | 4.08E-03 | 4.86E-02 | APOD/APOA4 | 2 |
| BP | GO:0031638 | zymogen activation | 2/31 | 20.63 | 4.22E-03 | 4.96E-02 | F12/CLEC3B | 2 |
| BP | GO:0050994 | regulation of lipid catabolic process | 2/31 | 19.96 | 4.50E-03 | 5.20E-02 | APOC3/APOA4 | 2 |
| BP | GO:0000302 | response to reactive oxygen species | 3/31 | 8.91 | 4.54E-03 | 5.20E-02 | ERN1/APOD/APOA4 | 3 |
| BP | GO:0042060 | wound healing | 4/31 | 5.76 | 4.85E-03 | 5.49E-02 | F13A1/F12/ACTB/FN1 | 4 |
| BP | GO:0046503 | glycerolipid catabolic process | 2/31 | 18.73 | 5.09E-03 | 5.62E-02 | APOC3/APOA4 | 2 |
| BP | GO:0055021 | regulation of cardiac muscle tissue growth | 2/31 | 18.73 | 5.09E-03 | 5.62E-02 | PI16/RBP4 | 2 |
| BP | GO:0070613 | regulation of protein processing | 2/31 | 18.17 | 5.40E-03 | 5.89E-02 | F12/CLEC3B | 2 |
| BP | GO:0007596 | blood coagulation | 3/31 | 8.15 | 5.81E-03 | 6.12E-02 | F13A1/F12/ACTB | 3 |
| BP | GO:0034329 | cell junction assembly | 4/31 | 5.46 | 5.84E-03 | 6.12E-02 | APOD/CLDN10/ACTB/FN1 | 4 |
| BP | GO:0019731 | antibacterial humoral response | 2/31 | 17.39 | 5.88E-03 | 6.12E-02 | JCHAIN/TF | 2 |
| BP | GO:0048662 | negative regulation of smooth muscle cell proliferation | 2/31 | 17.39 | 5.88E-03 | 6.12E-02 | APOD/IGFBP3 | 2 |
| BP | GO:0060420 | regulation of heart growth | 2/31 | 17.15 | 6.05E-03 | 6.15E-02 | PI16/RBP4 | 2 |
| BP | GO:1903317 | regulation of protein maturation | 2/31 | 17.15 | 6.05E-03 | 6.15E-02 | F12/CLEC3B | 2 |
| BP | GO:0050817 | coagulation | 3/31 | 7.97 | 6.17E-03 | 6.21E-02 | F13A1/F12/ACTB | 3 |
| BP | GO:0007599 | hemostasis | 3/31 | 7.91 | 6.32E-03 | 6.28E-02 | F13A1/F12/ACTB | 3 |
| BP | GO:0045661 | regulation of myoblast differentiation | 2/31 | 16.68 | 6.38E-03 | 6.28E-02 | ACTB/IGFBP3 | 2 |
| BP | GO:0019318 | hexose metabolic process | 3/31 | 7.84 | 6.47E-03 | 6.30E-02 | APOD/RBP4/IGFBP3 | 3 |
| BP | GO:0072593 | reactive oxygen species metabolic process | 3/31 | 7.80 | 6.55E-03 | 6.31E-02 | APOA4/GPX3/HBD | 3 |
| BP | GO:0045861 | negative regulation of proteolysis | 3/31 | 7.77 | 6.63E-03 | 6.31E-02 | FETUB/SERPINA5/SERPINA4 | 3 |
| BP | GO:0035924 | cellular response to vascular endothelial growth factor stimulus | 2/31 | 16.23 | 6.73E-03 | 6.34E-02 | ERN1/TNXB | 2 |
| BP | GO:0046470 | phosphatidylcholine metabolic process | 2/31 | 16.02 | 6.90E-03 | 6.41E-02 | APOA4/PON1 | 2 |
| BP | GO:0007160 | cell-matrix adhesion | 3/31 | 7.64 | 6.94E-03 | 6.41E-02 | APOD/TNXB/FN1 | 3 |
| BP | GO:0032720 | negative regulation of tumor necrosis factor production | 2/31 | 15.61 | 7.26E-03 | 6.57E-02 | LBP/ORM1 | 2 |
| BP | GO:0060191 | regulation of lipase activity | 2/31 | 15.61 | 7.26E-03 | 6.57E-02 | APOC3/APOA4 | 2 |
| BP | GO:0043086 | negative regulation of catalytic activity | 4/31 | 5.07 | 7.55E-03 | 6.76E-02 | FETUB/SERPINA5/APOC3/SERPINA4 | 4 |
| BP | GO:1903556 | negative regulation of tumor necrosis factor superfamily cytokine production | 2/31 | 15.22 | 7.62E-03 | 6.76E-02 | LBP/ORM1 | 2 |
| BP | GO:0048678 | response to axon injury | 2/31 | 14.49 | 8.37E-03 | 7.36E-02 | APOD/APOA4 | 2 |
| BP | GO:0006959 | humoral immune response | 3/31 | 7.08 | 8.56E-03 | 7.45E-02 | JCHAIN/C9/TF | 3 |
| BP | GO:0005996 | monosaccharide metabolic process | 3/31 | 7.00 | 8.83E-03 | 7.61E-02 | APOD/RBP4/IGFBP3 | 3 |
| BP | GO:0055017 | cardiac muscle tissue growth | 2/31 | 13.68 | 9.36E-03 | 7.99E-02 | PI16/RBP4 | 2 |
| BP | GO:0097006 | regulation of plasma lipoprotein particle levels | 2/31 | 13.38 | 9.77E-03 | 8.26E-02 | APOC3/APOA4 | 2 |
| BP | GO:0007044 | cell-substrate junction assembly | 2/31 | 12.81 | 1.06E-02 | 8.89E-02 | APOD/FN1 | 2 |
| BP | GO:0060419 | heart growth | 2/31 | 12.55 | 1.10E-02 | 9.16E-02 | PI16/RBP4 | 2 |
| BP | GO:0046620 | regulation of organ growth | 2/31 | 12.30 | 1.15E-02 | 9.37E-02 | PI16/RBP4 | 2 |
| BP | GO:0052548 | regulation of endopeptidase activity | 3/31 | 6.34 | 1.15E-02 | 9.37E-02 | FETUB/SERPINA5/SERPINA4 | 3 |
| BP | GO:0019217 | regulation of fatty acid metabolic process | 2/31 | 12.17 | 1.17E-02 | 9.37E-02 | APOC3/APOA4 | 2 |
| BP | GO:0042632 | cholesterol homeostasis | 2/31 | 12.17 | 1.17E-02 | 9.37E-02 | APOC3/APOA4 | 2 |
| BP | GO:0030100 | regulation of endocytosis | 3/31 | 6.28 | 1.19E-02 | 9.38E-02 | APOC3/ACTB/TF | 3 |
| BP | GO:0055092 | sterol homeostasis | 2/31 | 12.05 | 1.19E-02 | 9.38E-02 | APOC3/APOA4 | 2 |
| BP | GO:0150115 | cell-substrate junction organization | 2/31 | 11.94 | 1.21E-02 | 9.48E-02 | APOD/FN1 | 2 |
| BP | GO:0015914 | phospholipid transport | 2/31 | 11.82 | 1.24E-02 | 9.49E-02 | APOC3/APOA4 | 2 |
| BP | GO:0022600 | digestive system process | 2/31 | 11.82 | 1.24E-02 | 9.49E-02 | APOA4/RBP4 | 2 |
| BP | GO:0032642 | regulation of chemokine production | 2/31 | 11.71 | 1.26E-02 | 9.59E-02 | LBP/APOD | 2 |
| BP | GO:0032602 | chemokine production | 2/31 | 11.59 | 1.28E-02 | 9.68E-02 | LBP/APOD | 2 |
| BP | GO:0002532 | production of molecular mediator involved in inflammatory response | 2/31 | 11.49 | 1.31E-02 | 9.70E-02 | LBP/APOD | 2 |
| BP | GO:0032760 | positive regulation of tumor necrosis factor production | 2/31 | 11.49 | 1.31E-02 | 9.70E-02 | LBP/ORM1 | 2 |
| BP | GO:0006766 | vitamin metabolic process | 2/31 | 11.17 | 1.38E-02 | 1.01E-01 | BTD/GC | 2 |
| BP | GO:0045445 | myoblast differentiation | 2/31 | 11.07 | 1.40E-02 | 1.02E-01 | ACTB/IGFBP3 | 2 |
| BP | GO:1903557 | positive regulation of tumor necrosis factor superfamily cytokine production | 2/31 | 11.07 | 1.40E-02 | 1.02E-01 | LBP/ORM1 | 2 |
| BP | GO:0052547 | regulation of peptidase activity | 3/31 | 5.85 | 1.43E-02 | 1.03E-01 | FETUB/SERPINA5/SERPINA4 | 3 |
| BP | GO:0045833 | negative regulation of lipid metabolic process | 2/31 | 10.87 | 1.45E-02 | 1.03E-01 | APOC3/APOD | 2 |
| BP | GO:0048259 | regulation of receptor-mediated endocytosis | 2/31 | 10.59 | 1.53E-02 | 1.06E-01 | APOC3/TF | 2 |
| BP | GO:0048640 | negative regulation of developmental growth | 2/31 | 10.59 | 1.53E-02 | 1.06E-01 | PI16/RBP4 | 2 |
| BP | GO:0008202 | steroid metabolic process | 3/31 | 5.58 | 1.62E-02 | 1.06E-01 | APOA4/GC/PON1 | 3 |
| BP | GO:0048251 | elastic fiber assembly | 1/31 | 60.87 | 1.63E-02 | 1.06E-01 | TNXB | 1 |
| BP | GO:0060068 | vagina development | 1/31 | 60.87 | 1.63E-02 | 1.06E-01 | RBP4 | 1 |
| BP | GO:0070391 | response to lipoteichoic acid | 1/31 | 60.87 | 1.63E-02 | 1.06E-01 | LBP | 1 |
| BP | GO:0071223 | cellular response to lipoteichoic acid | 1/31 | 60.87 | 1.63E-02 | 1.06E-01 | LBP | 1 |
| BP | GO:0071803 | positive regulation of podosome assembly | 1/31 | 60.87 | 1.63E-02 | 1.06E-01 | LCP1 | 1 |
| BP | GO:1901388 | regulation of transforming growth factor beta activation | 1/31 | 60.87 | 1.63E-02 | 1.06E-01 | TNXB | 1 |
| BP | GO:1904729 | regulation of intestinal lipid absorption | 1/31 | 60.87 | 1.63E-02 | 1.06E-01 | APOA4 | 1 |
| BP | GO:1905918 | regulation of CoA-transferase activity | 1/31 | 60.87 | 1.63E-02 | 1.06E-01 | APOA4 | 1 |
| BP | GO:0048638 | regulation of developmental growth | 3/31 | 5.57 | 1.63E-02 | 1.06E-01 | PI16/RBP4/FN1 | 3 |
| BP | GO:0042742 | defense response to bacterium | 3/31 | 5.53 | 1.66E-02 | 1.07E-01 | JCHAIN/LBP/TF | 3 |
| BP | GO:0062012 | regulation of small molecule metabolic process | 3/31 | 5.42 | 1.75E-02 | 1.13E-01 | APOC3/APOA4/IGFBP3 | 3 |
| BP | GO:0010982 | regulation of high-density lipoprotein particle clearance | 1/31 | 55.34 | 1.79E-02 | 1.14E-01 | APOC3 | 1 |
| BP | GO:0019216 | regulation of lipid metabolic process | 3/31 | 5.34 | 1.82E-02 | 1.15E-01 | APOC3/APOD/APOA4 | 3 |
| BP | GO:0034379 | very-low-density lipoprotein particle assembly | 1/31 | 50.73 | 1.95E-02 | 1.18E-01 | APOC3 | 1 |
| BP | GO:0035112 | genitalia morphogenesis | 1/31 | 50.73 | 1.95E-02 | 1.18E-01 | RBP4 | 1 |
| BP | GO:0042117 | monocyte activation | 1/31 | 50.73 | 1.95E-02 | 1.18E-01 | FN1 | 1 |
| BP | GO:0071635 | negative regulation of transforming growth factor beta production | 1/31 | 50.73 | 1.95E-02 | 1.18E-01 | FN1 | 1 |
| BP | GO:1904478 | regulation of intestinal absorption | 1/31 | 50.73 | 1.95E-02 | 1.18E-01 | APOA4 | 1 |
| BP | GO:0019730 | antimicrobial humoral response | 2/31 | 9.15 | 2.01E-02 | 1.18E-01 | JCHAIN/TF | 2 |
| BP | GO:0031589 | cell-substrate adhesion | 3/31 | 5.13 | 2.03E-02 | 1.18E-01 | APOD/TNXB/FN1 | 3 |
| BP | GO:0007586 | digestion | 2/31 | 9.09 | 2.03E-02 | 1.18E-01 | APOA4/RBP4 | 2 |
| BP | GO:0010642 | negative regulation of platelet-derived growth factor receptor signaling pathway | 1/31 | 46.82 | 2.12E-02 | 1.18E-01 | APOD | 1 |
| BP | GO:0032490 | detection of molecule of bacterial origin | 1/31 | 46.82 | 2.12E-02 | 1.18E-01 | LBP | 1 |
| BP | GO:0034145 | positive regulation of toll-like receptor 4 signaling pathway | 1/31 | 46.82 | 2.12E-02 | 1.18E-01 | LBP | 1 |
| BP | GO:0034333 | adherens junction assembly | 1/31 | 46.82 | 2.12E-02 | 1.18E-01 | ACTB | 1 |
| BP | GO:0036363 | transforming growth factor beta activation | 1/31 | 46.82 | 2.12E-02 | 1.18E-01 | TNXB | 1 |
| BP | GO:0043568 | positive regulation of insulin-like growth factor receptor signaling pathway | 1/31 | 46.82 | 2.12E-02 | 1.18E-01 | IGFBP3 | 1 |
| BP | GO:0051657 | maintenance of organelle location | 1/31 | 46.82 | 2.12E-02 | 1.18E-01 | ALB | 1 |
| BP | GO:0051764 | actin crosslink formation | 1/31 | 46.82 | 2.12E-02 | 1.18E-01 | LCP1 | 1 |
| BP | GO:0061052 | negative regulation of cell growth involved in cardiac muscle cell development | 1/31 | 46.82 | 2.12E-02 | 1.18E-01 | PI16 | 1 |
| BP | GO:0071801 | regulation of podosome assembly | 1/31 | 46.82 | 2.12E-02 | 1.18E-01 | LCP1 | 1 |
| BP | GO:1900103 | positive regulation of endoplasmic reticulum unfolded protein response | 1/31 | 46.82 | 2.12E-02 | 1.18E-01 | ERN1 | 1 |
| BP | GO:0050878 | regulation of body fluid levels | 3/31 | 5.00 | 2.16E-02 | 1.20E-01 | F13A1/F12/ACTB | 3 |
| BP | GO:0008203 | cholesterol metabolic process | 2/31 | 8.70 | 2.21E-02 | 1.22E-01 | APOA4/PON1 | 2 |
| BP | GO:0000394 | RNA splicing, via endonucleolytic cleavage and ligation | 1/31 | 43.48 | 2.28E-02 | 1.25E-01 | ERN1 | 1 |
| BP | GO:0006066 | alcohol metabolic process | 3/31 | 4.90 | 2.29E-02 | 1.25E-01 | APOA4/RBP4/PON1 | 3 |
| BP | GO:0010565 | regulation of cellular ketone metabolic process | 2/31 | 8.45 | 2.33E-02 | 1.26E-01 | APOC3/APOA4 | 2 |
| BP | GO:0010989 | negative regulation of low-density lipoprotein particle clearance | 1/31 | 40.58 | 2.44E-02 | 1.26E-01 | APOC3 | 1 |
| BP | GO:0015670 | carbon dioxide transport | 1/31 | 40.58 | 2.44E-02 | 1.26E-01 | HBD | 1 |
| BP | GO:0015671 | oxygen transport | 1/31 | 40.58 | 2.44E-02 | 1.26E-01 | HBD | 1 |
| BP | GO:0042308 | negative regulation of protein import into nucleus | 1/31 | 40.58 | 2.44E-02 | 1.26E-01 | APOD | 1 |
| BP | GO:0045176 | apical protein localization | 1/31 | 40.58 | 2.44E-02 | 1.26E-01 | ACTB | 1 |
| BP | GO:0060347 | heart trabecula formation | 1/31 | 40.58 | 2.44E-02 | 1.26E-01 | RBP4 | 1 |
| BP | GO:0090209 | negative regulation of triglyceride metabolic process | 1/31 | 40.58 | 2.44E-02 | 1.26E-01 | APOC3 | 1 |
| BP | GO:2000402 | negative regulation of lymphocyte migration | 1/31 | 40.58 | 2.44E-02 | 1.26E-01 | APOD | 1 |
| BP | GO:1902652 | secondary alcohol metabolic process | 2/31 | 8.12 | 2.51E-02 | 1.29E-01 | APOA4/PON1 | 2 |
| BP | GO:0015748 | organophosphate ester transport | 2/31 | 8.01 | 2.57E-02 | 1.29E-01 | APOC3/APOA4 | 2 |
| BP | GO:0002679 | respiratory burst involved in defense response | 1/31 | 38.04 | 2.60E-02 | 1.29E-01 | LBP | 1 |
| BP | GO:0051895 | negative regulation of focal adhesion assembly | 1/31 | 38.04 | 2.60E-02 | 1.29E-01 | APOD | 1 |
| BP | GO:0060044 | negative regulation of cardiac muscle cell proliferation | 1/31 | 38.04 | 2.60E-02 | 1.29E-01 | RBP4 | 1 |
| BP | GO:0150118 | negative regulation of cell-substrate junction organization | 1/31 | 38.04 | 2.60E-02 | 1.29E-01 | APOD | 1 |
| BP | GO:1901142 | insulin metabolic process | 1/31 | 38.04 | 2.60E-02 | 1.29E-01 | ERN1 | 1 |
| BP | GO:0008037 | cell recognition | 2/31 | 7.91 | 2.64E-02 | 1.29E-01 | FETUB/LBP | 2 |
| BP | GO:0016125 | sterol metabolic process | 2/31 | 7.91 | 2.64E-02 | 1.29E-01 | APOA4/PON1 | 2 |
| BP | GO:0034614 | cellular response to reactive oxygen species | 2/31 | 7.91 | 2.64E-02 | 1.29E-01 | ERN1/APOA4 | 2 |
| BP | GO:0007043 | cell-cell junction assembly | 2/31 | 7.75 | 2.73E-02 | 1.30E-01 | CLDN10/ACTB | 2 |
| BP | GO:0008228 | opsonization | 1/31 | 35.81 | 2.76E-02 | 1.30E-01 | LBP | 1 |
| BP | GO:0032488 | Cdc42 protein signal transduction | 1/31 | 35.81 | 2.76E-02 | 1.30E-01 | APOC3 | 1 |
| BP | GO:0050746 | regulation of lipoprotein metabolic process | 1/31 | 35.81 | 2.76E-02 | 1.30E-01 | APOD | 1 |
| BP | GO:0060192 | negative regulation of lipase activity | 1/31 | 35.81 | 2.76E-02 | 1.30E-01 | APOC3 | 1 |
| BP | GO:0098974 | postsynaptic actin cytoskeleton organization | 1/31 | 35.81 | 2.76E-02 | 1.30E-01 | ACTB | 1 |
| BP | GO:0006631 | fatty acid metabolic process | 3/31 | 4.55 | 2.76E-02 | 1.30E-01 | APOC3/APOA4/TNXB | 3 |
| BP | GO:0035966 | response to topologically incorrect protein | 2/31 | 7.66 | 2.80E-02 | 1.31E-01 | ERN1/F12 | 2 |
| BP | GO:0006957 | complement activation, alternative pathway | 1/31 | 33.82 | 2.92E-02 | 1.33E-01 | C9 | 1 |
| BP | GO:0030299 | intestinal cholesterol absorption | 1/31 | 33.82 | 2.92E-02 | 1.33E-01 | APOA4 | 1 |
| BP | GO:0034384 | high-density lipoprotein particle clearance | 1/31 | 33.82 | 2.92E-02 | 1.33E-01 | APOC3 | 1 |
| BP | GO:0045780 | positive regulation of bone resorption | 1/31 | 33.82 | 2.92E-02 | 1.33E-01 | TF | 1 |
| BP | GO:0051917 | regulation of fibrinolysis | 1/31 | 33.82 | 2.92E-02 | 1.33E-01 | F12 | 1 |
| BP | GO:0090493 | catecholamine uptake | 1/31 | 33.82 | 2.92E-02 | 1.33E-01 | ACTB | 1 |
| BP | GO:0007338 | single fertilization | 2/31 | 7.33 | 3.03E-02 | 1.35E-01 | FETUB/SERPINA5 | 2 |
| BP | GO:0001558 | regulation of cell growth | 3/31 | 4.38 | 3.05E-02 | 1.35E-01 | PI16/IGFBP3/FN1 | 3 |
| BP | GO:0002281 | macrophage activation involved in immune response | 1/31 | 32.04 | 3.08E-02 | 1.35E-01 | LBP | 1 |
| BP | GO:0002523 | leukocyte migration involved in inflammatory response | 1/31 | 32.04 | 3.08E-02 | 1.35E-01 | LBP | 1 |
| BP | GO:0030540 | female genitalia development | 1/31 | 32.04 | 3.08E-02 | 1.35E-01 | RBP4 | 1 |
| BP | GO:0071800 | podosome assembly | 1/31 | 32.04 | 3.08E-02 | 1.35E-01 | LCP1 | 1 |
| BP | GO:1900242 | regulation of synaptic vesicle endocytosis | 1/31 | 32.04 | 3.08E-02 | 1.35E-01 | ACTB | 1 |
| BP | GO:0006633 | fatty acid biosynthetic process | 2/31 | 7.25 | 3.09E-02 | 1.35E-01 | APOC3/APOA4 | 2 |
| BP | GO:0036498 | IRE1-mediated unfolded protein response | 1/31 | 30.44 | 3.24E-02 | 1.39E-01 | ERN1 | 1 |
| BP | GO:0042359 | vitamin D metabolic process | 1/31 | 30.44 | 3.24E-02 | 1.39E-01 | GC | 1 |
| BP | GO:0061050 | regulation of cell growth involved in cardiac muscle cell development | 1/31 | 30.44 | 3.24E-02 | 1.39E-01 | PI16 | 1 |
| BP | GO:0099188 | postsynaptic cytoskeleton organization | 1/31 | 30.44 | 3.24E-02 | 1.39E-01 | ACTB | 1 |
| BP | GO:0055088 | lipid homeostasis | 2/31 | 6.96 | 3.34E-02 | 1.41E-01 | APOC3/APOA4 | 2 |
| BP | GO:0010985 | negative regulation of lipoprotein particle clearance | 1/31 | 28.99 | 3.40E-02 | 1.41E-01 | APOC3 | 1 |
| BP | GO:0035461 | vitamin transmembrane transport | 1/31 | 28.99 | 3.40E-02 | 1.41E-01 | GC | 1 |
| BP | GO:0071605 | monocyte chemotactic protein-1 production | 1/31 | 28.99 | 3.40E-02 | 1.41E-01 | APOD | 1 |
| BP | GO:0071637 | regulation of monocyte chemotactic protein-1 production | 1/31 | 28.99 | 3.40E-02 | 1.41E-01 | APOD | 1 |
| BP | GO:0032635 | interleukin-6 production | 2/31 | 6.88 | 3.41E-02 | 1.41E-01 | LBP/ORM1 | 2 |
| BP | GO:0032675 | regulation of interleukin-6 production | 2/31 | 6.88 | 3.41E-02 | 1.41E-01 | LBP/ORM1 | 2 |
| BP | GO:0035265 | organ growth | 2/31 | 6.88 | 3.41E-02 | 1.41E-01 | PI16/RBP4 | 2 |
| BP | GO:0010988 | regulation of low-density lipoprotein particle clearance | 1/31 | 27.67 | 3.55E-02 | 1.42E-01 | APOC3 | 1 |
| BP | GO:0015669 | gas transport | 1/31 | 27.67 | 3.55E-02 | 1.42E-01 | HBD | 1 |
| BP | GO:0015874 | norepinephrine transport | 1/31 | 27.67 | 3.55E-02 | 1.42E-01 | ACTB | 1 |
| BP | GO:0044241 | lipid digestion | 1/31 | 27.67 | 3.55E-02 | 1.42E-01 | APOA4 | 1 |
| BP | GO:0090208 | positive regulation of triglyceride metabolic process | 1/31 | 27.67 | 3.55E-02 | 1.42E-01 | APOA4 | 1 |
| BP | GO:0098856 | intestinal lipid absorption | 1/31 | 27.67 | 3.55E-02 | 1.42E-01 | APOA4 | 1 |
| BP | GO:0150105 | protein localization to cell-cell junction | 1/31 | 27.67 | 3.55E-02 | 1.42E-01 | ACTB | 1 |
| BP | GO:0007409 | axonogenesis | 3/31 | 4.08 | 3.66E-02 | 1.44E-01 | ACTB/CHL1/FN1 | 3 |
| BP | GO:0016338 | calcium-independent cell-cell adhesion via plasma membrane cell-adhesion molecules | 1/31 | 26.47 | 3.71E-02 | 1.44E-01 | CLDN10 | 1 |
| BP | GO:0019430 | removal of superoxide radicals | 1/31 | 26.47 | 3.71E-02 | 1.44E-01 | APOA4 | 1 |
| BP | GO:0046823 | negative regulation of nucleocytoplasmic transport | 1/31 | 26.47 | 3.71E-02 | 1.44E-01 | APOD | 1 |
| BP | GO:0060065 | uterus development | 1/31 | 26.47 | 3.71E-02 | 1.44E-01 | RBP4 | 1 |
| BP | GO:0071636 | positive regulation of transforming growth factor beta production | 1/31 | 26.47 | 3.71E-02 | 1.44E-01 | TNXB | 1 |
| BP | GO:0032640 | tumor necrosis factor production | 2/31 | 6.55 | 3.73E-02 | 1.44E-01 | LBP/ORM1 | 2 |
| BP | GO:0032680 | regulation of tumor necrosis factor production | 2/31 | 6.55 | 3.73E-02 | 1.44E-01 | LBP/ORM1 | 2 |
| BP | GO:0046890 | regulation of lipid biosynthetic process | 2/31 | 6.44 | 3.84E-02 | 1.47E-01 | APOC3/APOA4 | 2 |
| BP | GO:0010640 | regulation of platelet-derived growth factor receptor signaling pathway | 1/31 | 25.36 | 3.87E-02 | 1.47E-01 | APOD | 1 |
| BP | GO:0030277 | maintenance of gastrointestinal epithelium | 1/31 | 25.36 | 3.87E-02 | 1.47E-01 | RBP4 | 1 |
| BP | GO:0045723 | positive regulation of fatty acid biosynthetic process | 1/31 | 25.36 | 3.87E-02 | 1.47E-01 | APOA4 | 1 |
| BP | GO:0071706 | tumor necrosis factor superfamily cytokine production | 2/31 | 6.37 | 3.91E-02 | 1.47E-01 | LBP/ORM1 | 2 |
| BP | GO:1903555 | regulation of tumor necrosis factor superfamily cytokine production | 2/31 | 6.37 | 3.91E-02 | 1.47E-01 | LBP/ORM1 | 2 |
| BP | GO:0043567 | regulation of insulin-like growth factor receptor signaling pathway | 1/31 | 24.35 | 4.03E-02 | 1.48E-01 | IGFBP3 | 1 |
| BP | GO:0051788 | response to misfolded protein | 1/31 | 24.35 | 4.03E-02 | 1.48E-01 | F12 | 1 |
| BP | GO:0071450 | cellular response to oxygen radical | 1/31 | 24.35 | 4.03E-02 | 1.48E-01 | APOA4 | 1 |
| BP | GO:0071451 | cellular response to superoxide | 1/31 | 24.35 | 4.03E-02 | 1.48E-01 | APOA4 | 1 |
| BP | GO:0071676 | negative regulation of mononuclear cell migration | 1/31 | 24.35 | 4.03E-02 | 1.48E-01 | APOD | 1 |
| BP | GO:0090023 | positive regulation of neutrophil chemotaxis | 1/31 | 24.35 | 4.03E-02 | 1.48E-01 | LBP | 1 |
| BP | GO:0006929 | substrate-dependent cell migration | 1/31 | 23.41 | 4.19E-02 | 1.50E-01 | FN1 | 1 |
| BP | GO:0033622 | integrin activation | 1/31 | 23.41 | 4.19E-02 | 1.50E-01 | FN1 | 1 |
| BP | GO:0045717 | negative regulation of fatty acid biosynthetic process | 1/31 | 23.41 | 4.19E-02 | 1.50E-01 | APOC3 | 1 |
| BP | GO:0060343 | trabecula formation | 1/31 | 23.41 | 4.19E-02 | 1.50E-01 | RBP4 | 1 |
| BP | GO:0098581 | detection of external biotic stimulus | 1/31 | 23.41 | 4.19E-02 | 1.50E-01 | LBP | 1 |
| BP | GO:1903421 | regulation of synaptic vesicle recycling | 1/31 | 23.41 | 4.19E-02 | 1.50E-01 | ACTB | 1 |
| BP | GO:0010875 | positive regulation of cholesterol efflux | 1/31 | 22.54 | 4.35E-02 | 1.53E-01 | PON1 | 1 |
| BP | GO:0042730 | fibrinolysis | 1/31 | 22.54 | 4.35E-02 | 1.53E-01 | F12 | 1 |
| BP | GO:0050995 | negative regulation of lipid catabolic process | 1/31 | 22.54 | 4.35E-02 | 1.53E-01 | APOC3 | 1 |
| BP | GO:0050996 | positive regulation of lipid catabolic process | 1/31 | 22.54 | 4.35E-02 | 1.53E-01 | APOA4 | 1 |
| BP | GO:0000303 | response to superoxide | 1/31 | 21.74 | 4.50E-02 | 1.53E-01 | APOA4 | 1 |
| BP | GO:0002227 | innate immune response in mucosa | 1/31 | 21.74 | 4.50E-02 | 1.53E-01 | APOA4 | 1 |
| BP | GO:0003094 | glomerular filtration | 1/31 | 21.74 | 4.50E-02 | 1.53E-01 | JCHAIN | 1 |
| BP | GO:0016540 | protein autoprocessing | 1/31 | 21.74 | 4.50E-02 | 1.53E-01 | F12 | 1 |
| BP | GO:0030194 | positive regulation of blood coagulation | 1/31 | 21.74 | 4.50E-02 | 1.53E-01 | F12 | 1 |
| BP | GO:0044342 | type B pancreatic cell proliferation | 1/31 | 21.74 | 4.50E-02 | 1.53E-01 | IGFBP3 | 1 |
| BP | GO:0071624 | positive regulation of granulocyte chemotaxis | 1/31 | 21.74 | 4.50E-02 | 1.53E-01 | LBP | 1 |
| BP | GO:1900048 | positive regulation of hemostasis | 1/31 | 21.74 | 4.50E-02 | 1.53E-01 | F12 | 1 |
| BP | GO:1903055 | positive regulation of extracellular matrix organization | 1/31 | 21.74 | 4.50E-02 | 1.53E-01 | TNXB | 1 |
| BP | GO:2000819 | regulation of nucleotide-excision repair | 1/31 | 21.74 | 4.50E-02 | 1.53E-01 | ACTB | 1 |
| BP | GO:0009566 | fertilization | 2/31 | 5.88 | 4.52E-02 | 1.53E-01 | FETUB/SERPINA5 | 2 |
| BP | GO:0000305 | response to oxygen radical | 1/31 | 20.99 | 4.66E-02 | 1.54E-01 | APOA4 | 1 |
| BP | GO:0006910 | phagocytosis, recognition | 1/31 | 20.99 | 4.66E-02 | 1.54E-01 | LBP | 1 |
| BP | GO:0007342 | fusion of sperm to egg plasma membrane involved in single fertilization | 1/31 | 20.99 | 4.66E-02 | 1.54E-01 | SERPINA5 | 1 |
| BP | GO:1900746 | regulation of vascular endothelial growth factor signaling pathway | 1/31 | 20.99 | 4.66E-02 | 1.54E-01 | TNXB | 1 |
| BP | GO:0016049 | cell growth | 3/31 | 3.67 | 4.73E-02 | 1.54E-01 | PI16/IGFBP3/FN1 | 3 |
| BP | GO:0001819 | positive regulation of cytokine production | 3/31 | 3.66 | 4.78E-02 | 1.54E-01 | LBP/ORM1/TNXB | 3 |
| BP | GO:0045216 | cell-cell junction organization | 2/31 | 5.69 | 4.80E-02 | 1.54E-01 | CLDN10/ACTB | 2 |
| BP | GO:0003298 | physiological muscle hypertrophy | 1/31 | 20.29 | 4.82E-02 | 1.54E-01 | PI16 | 1 |
| BP | GO:0003301 | physiological cardiac muscle hypertrophy | 1/31 | 20.29 | 4.82E-02 | 1.54E-01 | PI16 | 1 |
| BP | GO:0010039 | response to iron ion | 1/31 | 20.29 | 4.82E-02 | 1.54E-01 | TF | 1 |
| BP | GO:0035640 | exploration behavior | 1/31 | 20.29 | 4.82E-02 | 1.54E-01 | CHL1 | 1 |
| BP | GO:0043032 | positive regulation of macrophage activation | 1/31 | 20.29 | 4.82E-02 | 1.54E-01 | LBP | 1 |
| BP | GO:0050820 | positive regulation of coagulation | 1/31 | 20.29 | 4.82E-02 | 1.54E-01 | F12 | 1 |
| BP | GO:0061049 | cell growth involved in cardiac muscle cell development | 1/31 | 20.29 | 4.82E-02 | 1.54E-01 | PI16 | 1 |
| BP | GO:0097205 | renal filtration | 1/31 | 20.29 | 4.82E-02 | 1.54E-01 | JCHAIN | 1 |
| BP | GO:1900101 | regulation of endoplasmic reticulum unfolded protein response | 1/31 | 20.29 | 4.82E-02 | 1.54E-01 | ERN1 | 1 |
| BP | GO:0032372 | negative regulation of sterol transport | 1/31 | 19.64 | 4.97E-02 | 1.55E-01 | APOC3 | 1 |
| BP | GO:0032375 | negative regulation of cholesterol transport | 1/31 | 19.64 | 4.97E-02 | 1.55E-01 | APOC3 | 1 |
| BP | GO:0032682 | negative regulation of chemokine production | 1/31 | 19.64 | 4.97E-02 | 1.55E-01 | APOD | 1 |
| BP | GO:0034143 | regulation of toll-like receptor 4 signaling pathway | 1/31 | 19.64 | 4.97E-02 | 1.55E-01 | LBP | 1 |
| BP | GO:0034755 | iron ion transmembrane transport | 1/31 | 19.64 | 4.97E-02 | 1.55E-01 | TF | 1 |
| BP | GO:0043507 | positive regulation of JUN kinase activity | 1/31 | 19.64 | 4.97E-02 | 1.55E-01 | ERN1 | 1 |
| BP | GO:1902547 | regulation of cellular response to vascular endothelial growth factor stimulus | 1/31 | 19.64 | 4.97E-02 | 1.55E-01 | TNXB | 1 |
| CC | GO:0072562 | blood microparticle | 12/33 | 50.22 | 4.06E-18 | 5.20E-16 | JCHAIN/ORM1/C9/F13A1/ALB/ACTB/APOA4/GC/PON1/HBD/FN1/TF | 12 |
| CC | GO:0062023 | collagen-containing extracellular matrix | 9/33 | 12.64 | 2.27E-08 | 1.45E-06 | ORM1/SERPINA5/F13A1/APOC3/F12/APOA4/CLEC3B/TNXB/FN1 | 9 |
| CC | GO:0031091 | platelet alpha granule | 5/33 | 33.11 | 3.85E-07 | 1.64E-05 | ORM1/SERPINA5/F13A1/ALB/FN1 | 5 |
| CC | GO:0034774 | secretory granule lumen | 7/33 | 13.10 | 8.14E-07 | 1.89E-05 | ORM1/F13A1/SERPINA4/ALB/CLEC3B/FN1/TF | 7 |
| CC | GO:0060205 | cytoplasmic vesicle lumen | 7/33 | 12.98 | 8.66E-07 | 1.89E-05 | ORM1/F13A1/SERPINA4/ALB/CLEC3B/FN1/TF | 7 |
| CC | GO:0031983 | vesicle lumen | 7/33 | 12.94 | 8.84E-07 | 1.89E-05 | ORM1/F13A1/SERPINA4/ALB/CLEC3B/FN1/TF | 7 |
| CC | GO:0031093 | platelet alpha granule lumen | 4/33 | 35.98 | 4.47E-06 | 8.18E-05 | ORM1/F13A1/ALB/FN1 | 4 |
| CC | GO:0034364 | high-density lipoprotein particle | 3/33 | 69.53 | 1.05E-05 | 1.69E-04 | APOC3/APOA4/PON1 | 3 |
| CC | GO:0034358 | plasma lipoprotein particle | 3/33 | 50.22 | 2.86E-05 | 3.67E-04 | APOC3/APOA4/PON1 | 3 |
| CC | GO:1990777 | lipoprotein particle | 3/33 | 50.22 | 2.86E-05 | 3.67E-04 | APOC3/APOA4/PON1 | 3 |
| CC | GO:0032994 | protein-lipid complex | 3/33 | 46.35 | 3.65E-05 | 4.25E-04 | APOC3/APOA4/PON1 | 3 |
| CC | GO:0005788 | endoplasmic reticulum lumen | 5/33 | 9.63 | 1.55E-04 | 1.65E-03 | ALB/APOA4/IGFBP3/FN1/TF | 5 |
| CC | GO:0042627 | chylomicron | 2/33 | 100.43 | 1.74E-04 | 1.72E-03 | APOC3/APOA4 | 2 |
| CC | GO:0031089 | platelet dense granule lumen | 2/33 | 86.09 | 2.40E-04 | 2.19E-03 | SERPINA4/CLEC3B | 2 |
| CC | GO:0034361 | very-low-density lipoprotein particle | 2/33 | 60.26 | 4.98E-04 | 3.98E-03 | APOC3/APOA4 | 2 |
| CC | GO:0034385 | triglyceride-rich plasma lipoprotein particle | 2/33 | 60.26 | 4.98E-04 | 3.98E-03 | APOC3/APOA4 | 2 |
| CC | GO:0042827 | platelet dense granule | 2/33 | 57.39 | 5.50E-04 | 4.14E-03 | SERPINA4/CLEC3B | 2 |
| CC | GO:0005884 | actin filament | 2/33 | 10.21 | 1.64E-02 | 9.43E-02 | LCP1/ACTB | 2 |
| CC | GO:0071745 | IgA immunoglobulin complex | 1/33 | 60.26 | 1.65E-02 | 9.43E-02 | JCHAIN | 1 |
| CC | GO:0099571 | postsynaptic cytoskeleton | 1/33 | 60.26 | 1.65E-02 | 9.43E-02 | ACTB | 1 |
| CC | GO:0140092 | bBAF complex | 1/33 | 60.26 | 1.65E-02 | 9.43E-02 | ACTB | 1 |
| CC | GO:0031094 | platelet dense tubular network | 1/33 | 54.78 | 1.81E-02 | 9.43E-02 | SERPINA5 | 1 |
| CC | GO:0031838 | haptoglobin-hemoglobin complex | 1/33 | 54.78 | 1.81E-02 | 9.43E-02 | HBD | 1 |
| CC | GO:1904090 | peptidase inhibitor complex | 1/33 | 54.78 | 1.81E-02 | 9.43E-02 | SERPINA5 | 1 |
| CC | GO:0005833 | hemoglobin complex | 1/33 | 50.22 | 1.97E-02 | 9.43E-02 | HBD | 1 |
| CC | GO:0042571 | immunoglobulin complex, circulating | 1/33 | 50.22 | 1.97E-02 | 9.43E-02 | JCHAIN | 1 |
| CC | GO:0070160 | tight junction | 2/33 | 9.20 | 1.99E-02 | 9.43E-02 | CLDN10/ACTB | 2 |
| CC | GO:0035060 | brahma complex | 1/33 | 43.04 | 2.30E-02 | 9.81E-02 | ACTB | 1 |
| CC | GO:0071564 | npBAF complex | 1/33 | 43.04 | 2.30E-02 | 9.81E-02 | ACTB | 1 |
| CC | GO:0140288 | GBAF complex | 1/33 | 43.04 | 2.30E-02 | 9.81E-02 | ACTB | 1 |
| CC | GO:0016586 | RSC-type complex | 1/33 | 40.17 | 2.46E-02 | 1.02E-01 | ACTB | 1 |
| CC | GO:0043296 | apical junction complex | 2/33 | 8.03 | 2.56E-02 | 1.02E-01 | CLDN10/ACTB | 2 |
| CC | GO:0071565 | nBAF complex | 1/33 | 37.66 | 2.62E-02 | 1.02E-01 | ACTB | 1 |
| CC | GO:0098637 | protein complex involved in cell-matrix adhesion | 1/33 | 35.45 | 2.79E-02 | 1.05E-01 | TNXB | 1 |
| CC | GO:0044305 | calyx of Held | 1/33 | 31.72 | 3.11E-02 | 1.14E-01 | ACTB | 1 |
| CC | GO:0005769 | early endosome | 3/33 | 4.23 | 3.33E-02 | 1.18E-01 | APOC3/APOA4/TF | 3 |
| CC | GO:0045177 | apical part of cell | 3/33 | 3.85 | 4.22E-02 | 1.42E-01 | CHL1/FN1/TF | 3 |
| CC | GO:0046930 | pore complex | 1/33 | 23.18 | 4.23E-02 | 1.42E-01 | C9 | 1 |
| CC | GO:0001891 | phagocytic cup | 1/33 | 21.52 | 4.55E-02 | 1.49E-01 | LCP1 | 1 |
| CC | GO:0016514 | SWI/SNF complex | 1/33 | 20.09 | 4.86E-02 | 1.49E-01 | ACTB | 1 |
| MF | GO:0005539 | glycosaminoglycan binding | 6/33 | 14.01 | 3.70E-06 | 4.85E-04 | JCHAIN/HABP2/SERPINA5/CLEC3B/TNXB/FN1 | 6 |
| MF | GO:0016209 | antioxidant activity | 4/33 | 27.01 | 1.40E-05 | 8.88E-04 | ALB/APOA4/GPX3/HBD | 4 |
| MF | GO:0031210 | phosphatidylcholine binding | 3/33 | 56.05 | 2.03E-05 | 8.88E-04 | JCHAIN/SERPINA5/APOA4 | 3 |
| MF | GO:0061134 | peptidase regulator activity | 5/33 | 12.29 | 4.88E-05 | 1.60E-03 | FETUB/SERPINA5/SERPINA4/PI16/FN1 | 5 |
| MF | GO:1901681 | sulfur compound binding | 5/33 | 10.19 | 1.18E-04 | 2.63E-03 | SERPINA5/CLEC3B/TNXB/FN1/TF | 5 |
| MF | GO:0050997 | quaternary ammonium group binding | 3/33 | 31.14 | 1.21E-04 | 2.63E-03 | JCHAIN/SERPINA5/APOA4 | 3 |
| MF | GO:0008201 | heparin binding | 4/33 | 12.88 | 2.50E-04 | 4.19E-03 | SERPINA5/CLEC3B/TNXB/FN1 | 4 |
| MF | GO:0030414 | peptidase inhibitor activity | 4/33 | 12.81 | 2.56E-04 | 4.19E-03 | FETUB/SERPINA5/SERPINA4/PI16 | 4 |
| MF | GO:0043178 | alcohol binding | 3/33 | 18.89 | 5.29E-04 | 7.41E-03 | APOC3/APOD/RBP4 | 3 |
| MF | GO:0004857 | enzyme inhibitor activity | 5/33 | 7.26 | 5.66E-04 | 7.41E-03 | FETUB/SERPINA5/APOC3/SERPINA4/PI16 | 5 |
| MF | GO:0005496 | steroid binding | 3/33 | 16.48 | 7.88E-04 | 9.38E-03 | APOC3/APOD/GC | 3 |
| MF | GO:0005543 | phospholipid binding | 5/33 | 5.79 | 1.56E-03 | 1.69E-02 | JCHAIN/SERPINA5/APOC3/APOA4/PON1 | 5 |
| MF | GO:0090482 | vitamin transmembrane transporter activity | 2/33 | 32.03 | 1.77E-03 | 1.69E-02 | RBP4/GC | 2 |
| MF | GO:0002020 | protease binding | 3/33 | 11.84 | 2.04E-03 | 1.69E-02 | SERPINA5/CHL1/FN1 | 3 |
| MF | GO:0005501 | retinoid binding | 2/33 | 29.50 | 2.08E-03 | 1.69E-02 | SERPINA5/RBP4 | 2 |
| MF | GO:0019840 | isoprenoid binding | 2/33 | 29.50 | 2.08E-03 | 1.69E-02 | SERPINA5/RBP4 | 2 |
| MF | GO:0019825 | oxygen binding | 2/33 | 28.74 | 2.19E-03 | 1.69E-02 | ALB/HBD | 2 |
| MF | GO:0019842 | vitamin binding | 3/33 | 11.21 | 2.39E-03 | 1.74E-02 | ALB/RBP4/GC | 3 |
| MF | GO:0005178 | integrin binding | 3/33 | 10.92 | 2.57E-03 | 1.77E-02 | LCP1/TNXB/FN1 | 3 |
| MF | GO:0004866 | endopeptidase inhibitor activity | 3/33 | 10.01 | 3.29E-03 | 2.15E-02 | FETUB/SERPINA5/SERPINA4 | 3 |
| MF | GO:0005319 | lipid transporter activity | 3/33 | 9.78 | 3.51E-03 | 2.19E-02 | APOD/APOA4/RBP4 | 3 |
| MF | GO:0015485 | cholesterol binding | 2/33 | 21.56 | 3.87E-03 | 2.31E-02 | APOC3/APOD | 2 |
| MF | GO:0061135 | endopeptidase regulator activity | 3/33 | 9.14 | 4.24E-03 | 2.36E-02 | FETUB/SERPINA5/SERPINA4 | 3 |
| MF | GO:0004601 | peroxidase activity | 2/33 | 20.38 | 4.32E-03 | 2.36E-02 | GPX3/HBD | 2 |
| MF | GO:0016684 | oxidoreductase activity, acting on peroxide as acceptor | 2/33 | 19.67 | 4.63E-03 | 2.43E-02 | GPX3/HBD | 2 |
| MF | GO:0032934 | sterol binding | 2/33 | 18.08 | 5.46E-03 | 2.73E-02 | APOC3/APOD | 2 |
| MF | GO:0005507 | copper ion binding | 2/33 | 17.79 | 5.63E-03 | 2.73E-02 | ALB/APOA4 | 2 |
| MF | GO:0043177 | organic acid binding | 3/33 | 8.12 | 5.89E-03 | 2.75E-02 | SERPINA5/ALB/HBD | 3 |
| MF | GO:0005518 | collagen binding | 2/33 | 16.48 | 6.53E-03 | 2.95E-02 | TNXB/FN1 | 2 |
| MF | GO:0033293 | monocarboxylic acid binding | 2/33 | 14.19 | 8.73E-03 | 3.81E-02 | SERPINA5/ALB | 2 |
| MF | GO:0140104 | molecular carrier activity | 2/33 | 11.80 | 1.24E-02 | 5.25E-02 | HBD/TF | 2 |
| MF | GO:0004867 | serine-type endopeptidase inhibitor activity | 2/33 | 11.10 | 1.40E-02 | 5.72E-02 | SERPINA5/SERPINA4 | 2 |
| MF | GO:0015643 | toxic substance binding | 1/33 | 56.05 | 1.77E-02 | 6.27E-02 | ALB | 1 |
| MF | GO:0030957 | Tat protein binding | 1/33 | 56.05 | 1.77E-02 | 6.27E-02 | ACTB | 1 |
| MF | GO:0031720 | haptoglobin binding | 1/33 | 56.05 | 1.77E-02 | 6.27E-02 | HBD | 1 |
| MF | GO:0071723 | lipopeptide binding | 1/33 | 56.05 | 1.77E-02 | 6.27E-02 | LBP | 1 |
| MF | GO:0098973 | structural constituent of postsynaptic actin cytoskeleton | 1/33 | 56.05 | 1.77E-02 | 6.27E-02 | ACTB | 1 |
| MF | GO:0008199 | ferric iron binding | 1/33 | 50.95 | 1.95E-02 | 6.37E-02 | TF | 1 |
| MF | GO:0050998 | nitric-oxide synthase binding | 1/33 | 50.95 | 1.95E-02 | 6.37E-02 | ACTB | 1 |
| MF | GO:1990459 | transferrin receptor binding | 1/33 | 50.95 | 1.95E-02 | 6.37E-02 | TF | 1 |
| MF | GO:0005527 | macrolide binding | 1/33 | 46.71 | 2.12E-02 | 6.78E-02 | ALB | 1 |
| MF | GO:0031994 | insulin-like growth factor I binding | 1/33 | 43.11 | 2.30E-02 | 7.03E-02 | IGFBP3 | 1 |
| MF | GO:0051087 | protein-folding chaperone binding | 2/33 | 8.43 | 2.34E-02 | 7.03E-02 | ERN1/ALB | 2 |
| MF | GO:0005161 | platelet-derived growth factor receptor binding | 1/33 | 40.03 | 2.47E-02 | 7.03E-02 | ERN1 | 1 |
| MF | GO:0005344 | oxygen carrier activity | 1/33 | 40.03 | 2.47E-02 | 7.03E-02 | HBD | 1 |
| MF | GO:0016530 | metallochaperone activity | 1/33 | 40.03 | 2.47E-02 | 7.03E-02 | TF | 1 |
| MF | GO:0008191 | metalloendopeptidase inhibitor activity | 1/33 | 35.03 | 2.82E-02 | 7.38E-02 | FETUB | 1 |
| MF | GO:0019841 | retinol binding | 1/33 | 35.03 | 2.82E-02 | 7.38E-02 | RBP4 | 1 |
| MF | GO:0034987 | immunoglobulin receptor binding | 1/33 | 35.03 | 2.82E-02 | 7.38E-02 | JCHAIN | 1 |
| MF | GO:0072542 | protein phosphatase activator activity | 1/33 | 35.03 | 2.82E-02 | 7.38E-02 | IGFBP3 | 1 |
| MF | GO:0055102 | lipase inhibitor activity | 1/33 | 32.97 | 2.99E-02 | 7.68E-02 | APOC3 | 1 |
| MF | GO:0005520 | insulin-like growth factor binding | 1/33 | 29.50 | 3.34E-02 | 7.95E-02 | IGFBP3 | 1 |
| MF | GO:0016918 | retinal binding | 1/33 | 29.50 | 3.34E-02 | 7.95E-02 | RBP4 | 1 |
| MF | GO:0019211 | phosphatase activator activity | 1/33 | 29.50 | 3.34E-02 | 7.95E-02 | IGFBP3 | 1 |
| MF | GO:0042834 | peptidoglycan binding | 1/33 | 29.50 | 3.34E-02 | 7.95E-02 | JCHAIN | 1 |
| MF | GO:0001972 | retinoic acid binding | 1/33 | 28.02 | 3.51E-02 | 7.96E-02 | SERPINA5 | 1 |
| MF | GO:0051787 | misfolded protein binding | 1/33 | 28.02 | 3.51E-02 | 7.96E-02 | F12 | 1 |
| MF | GO:0005201 | extracellular matrix structural constituent | 2/33 | 6.75 | 3.52E-02 | 7.96E-02 | TNXB/FN1 | 2 |
| MF | GO:0099186 | structural constituent of postsynapse | 1/33 | 26.69 | 3.68E-02 | 8.14E-02 | ACTB | 1 |
| MF | GO:0004252 | serine-type endopeptidase activity | 2/33 | 6.44 | 3.84E-02 | 8.14E-02 | HABP2/F12 | 2 |
| MF | GO:0004602 | glutathione peroxidase activity | 1/33 | 25.48 | 3.85E-02 | 8.14E-02 | GPX3 | 1 |
| MF | GO:0120020 | cholesterol transfer activity | 1/33 | 25.48 | 3.85E-02 | 8.14E-02 | APOA4 | 1 |
| MF | GO:0120015 | sterol transfer activity | 1/33 | 24.37 | 4.03E-02 | 8.37E-02 | APOA4 | 1 |
| MF | GO:0019865 | immunoglobulin binding | 1/33 | 23.35 | 4.20E-02 | 8.59E-02 | JCHAIN | 1 |
| MF | GO:0008236 | serine-type peptidase activity | 2/33 | 5.84 | 4.59E-02 | 9.04E-02 | HABP2/F12 | 2 |
| MF | GO:0008198 | ferrous iron binding | 1/33 | 20.76 | 4.71E-02 | 9.04E-02 | TF | 1 |
| MF | GO:0098918 | structural constituent of synapse | 1/33 | 20.76 | 4.71E-02 | 9.04E-02 | ACTB | 1 |
| MF | GO:0031406 | carboxylic acid binding | 2/33 | 5.75 | 4.72E-02 | 9.04E-02 | SERPINA5/ALB | 2 |
| MF | GO:0017171 | serine hydrolase activity | 2/33 | 5.72 | 4.76E-02 | 9.04E-02 | HABP2/F12 | 2 |
| Organismal Systems | hsa04610 | Complement and coagulation cascades | 4/17 | 23.69 | 1.96E-05 | 1.45E-03 | C9/SERPINA5/F13A1/F12 | 4 |
| Organismal Systems | hsa04977 | Vitamin digestion and absorption | 2/17 | 40.09 | 1.10E-03 | 4.06E-02 | BTD/APOA4 | 2 |
| Organismal Systems | hsa04979 | Cholesterol metabolism | 2/17 | 20.44 | 4.18E-03 | 8.84E-02 | APOC3/APOA4 | 2 |
| Cellular Processes | hsa04510 | Focal adhesion | 3/17 | 7.70 | 6.36E-03 | 8.84E-02 | ACTB/TNXB/FN1 | 3 |
| Human Diseases | hsa05417 | Lipid and atherosclerosis | 3/17 | 7.24 | 7.55E-03 | 8.84E-02 | ERN1/LBP/APOA4 | 3 |
| Organismal Systems | hsa04918 | Thyroid hormone synthesis | 2/17 | 13.90 | 8.86E-03 | 8.84E-02 | ALB/GPX3 | 2 |
| Cellular Processes | hsa04810 | Regulation of actin cytoskeleton | 3/17 | 6.80 | 8.97E-03 | 8.84E-02 | C9/ACTB/FN1 | 3 |
| Human Diseases | hsa05100 | Bacterial invasion of epithelial cells | 2/17 | 13.36 | 9.55E-03 | 8.84E-02 | ACTB/FN1 | 2 |
| Environmental Information Processing | hsa04512 | ECM-receptor interaction | 2/17 | 11.71 | 1.23E-02 | 1.01E-01 | TNXB/FN1 | 2 |
| Human Diseases | hsa05146 | Amoebiasis | 2/17 | 10.12 | 1.62E-02 | 1.20E-01 | C9/FN1 | 2 |
| Organismal Systems | hsa04670 | Leukocyte transendothelial migration | 2/17 | 8.98 | 2.03E-02 | 1.37E-01 | CLDN10/ACTB | 2 |
| Cellular Processes | hsa04210 | Apoptosis | 2/17 | 7.66 | 2.74E-02 | 1.60E-01 | ERN1/ACTB | 2 |
| Human Diseases | hsa05135 | Yersinia infection | 2/17 | 7.55 | 2.81E-02 | 1.60E-01 | ACTB/FN1 | 2 |
| Human Diseases | hsa05014 | Amyotrophic lateral sclerosis | 3/17 | 4.21 | 3.20E-02 | 1.69E-01 | ERN1/ACTB/GPX3 | 3 |
| Cellular Processes | hsa04530 | Tight junction | 2/17 | 6.13 | 4.12E-02 | 2.03E-01 | CLDN10/ACTB | 2 |

Table S14: Enrichment analysis results of transcriptomics DEGs

| Category | ID | Description | GeneRatio | FoldEnrichment | pvalue | p.adjust | geneID | Count |
| --- | --- | --- | --- | --- | --- | --- | --- | --- |
| BP | GO:0002764 | immune response-regulating signaling pathway | 26/235 | 4.18 | 8.19E-10 | 2.00E-06 | GBP1/GBP5/NCR3/BLK/AIM2/CD19/CD8A/GATA3/CLEC4D/CD22/ZAP70/FOXP1/MS4A1/NAIP/BMX/TIFA/PLSCR1/LY96/ALPK1/IGHD/LAT/LTF/CD8B/FGR/NLRC3/CTSS | 26 |
| BP | GO:0002757 | immune response-activating signaling pathway | 25/235 | 4.24 | 1.27E-09 | 2.00E-06 | GBP1/GBP5/NCR3/BLK/AIM2/CD19/CD8A/GATA3/CD22/ZAP70/FOXP1/MS4A1/NAIP/BMX/TIFA/PLSCR1/LY96/ALPK1/IGHD/LAT/LTF/CD8B/FGR/NLRC3/CTSS | 25 |
| BP | GO:0042742 | defense response to bacterium | 19/235 | 4.62 | 3.50E-08 | 3.68E-05 | GBP1/GBP5/IL7R/CLEC4D/IL23A/ANXA3/FOXP1/NAIP/GBP3/BPI/S100A8/S100A12/LCN2/CEBPB/LTF/FGR/HP/DEFA3/DEFA4 | 19 |
| BP | GO:0071356 | cellular response to tumor necrosis factor | 16/235 | 5.40 | 5.26E-08 | 3.98E-05 | GBP1/AIM2/GAS6/TNFRSF25/JAK2/GATA3/LRRK2/FAS/TCL1A/FOXP1/NAIP/STAT1/GBP3/CYP1B1/MMP8/CARD16 | 16 |
| BP | GO:0002768 | immune response-regulating cell surface receptor signaling pathway | 19/235 | 4.41 | 7.40E-08 | 3.98E-05 | GBP1/NCR3/BLK/CD19/CD8A/GATA3/CLEC4D/CD22/ZAP70/FOXP1/MS4A1/BMX/PLSCR1/LY96/IGHD/LAT/LTF/CD8B/FGR | 19 |
| BP | GO:0001819 | positive regulation of cytokine production | 23/235 | 3.70 | 7.56E-08 | 3.98E-05 | GBP5/CD274/HPSE/AIM2/TIGIT/C5/JAK2/TBX21/GATA3/LRRK2/IL23A/FOXP1/NAIP/TRIM6/STAT1/LY96/CYP1B1/LPL/IGHD/MMP8/CEBPB/FGR/RBM47 | 23 |
| BP | GO:0002429 | immune response-activating cell surface receptor signaling pathway | 18/235 | 4.55 | 1.05E-07 | 4.73E-05 | GBP1/NCR3/BLK/CD19/CD8A/GATA3/CD22/ZAP70/FOXP1/MS4A1/BMX/PLSCR1/LY96/IGHD/LAT/LTF/CD8B/FGR | 18 |
| BP | GO:0034612 | response to tumor necrosis factor | 16/235 | 4.96 | 1.68E-07 | 6.64E-05 | GBP1/AIM2/GAS6/TNFRSF25/JAK2/GATA3/LRRK2/FAS/TCL1A/FOXP1/NAIP/STAT1/GBP3/CYP1B1/MMP8/CARD16 | 16 |
| BP | GO:0002274 | myeloid leukocyte activation | 15/235 | 5.00 | 3.75E-07 | 1.32E-04 | BATF2/JAK2/LRRK2/CLEC4D/CD177/ANXA3/C1QA/FOXP1/PLSCR1/DYSF/BPI/S100A12/LAT/MMP8/FGR | 15 |
| BP | GO:0031349 | positive regulation of defense response | 21/235 | 3.51 | 6.71E-07 | 2.12E-04 | GBP5/NCR3/AIM2/LRRK2/IL23A/NAIP/TRIM6/TIFA/PLSCR1/LY96/LPL/ALPK1/S100A8/S100A12/ARG1/MMP8/CEBPB/LTF/NLRC3/CTSS/RBM47 | 21 |
| BP | GO:0033674 | positive regulation of kinase activity | 17/235 | 4.05 | 1.20E-06 | 3.43E-04 | EPHA4/DOK7/CD19/LTK/GAS6/JAK2/LRRK2/NRG1/EGF/IL23A/TCL1A/NAB2/S100A12/LAT/LTF/FGR/MT3 | 17 |
| BP | GO:0002460 | adaptive immune response based on somatic recombination of immune receptors built from immunoglobulin superfamily domains | 18/235 | 3.80 | 1.42E-06 | 3.73E-04 | CD274/CCR6/CD19/SERPING1/IL7R/CD8A/C5/JAK2/TBX21/GATA3/C1QB/IL23A/C1QA/C1QC/IGHD/ARG1/JUNB/C4BPA | 18 |
| BP | GO:0002443 | leukocyte mediated immunity | 20/235 | 3.45 | 1.68E-06 | 3.90E-04 | NCR3/CCR6/CD19/SERPING1/IL7R/CD8A/C5/TBX21/GATA3/C1QB/CD177/IL23A/ANXA3/C1QA/C1QC/IGHD/LAT/ARG1/FGR/C4BPA | 20 |
| BP | GO:0032496 | response to lipopolysaccharide | 17/235 | 3.92 | 1.85E-06 | 3.90E-04 | GBP5/CD274/JAK2/FOXP1/ADM/TRIM6/GBP3/LY96/BPI/S100A8/MMP8/CEBPB/LTF/TH/CARD16/DEFA3/DEFA4 | 17 |
| BP | GO:0050851 | antigen receptor-mediated signaling pathway | 13/235 | 5.09 | 1.85E-06 | 3.90E-04 | GBP1/BLK/CD19/CD8A/GATA3/CD22/ZAP70/FOXP1/MS4A1/BMX/IGHD/LAT/CD8B | 13 |
| BP | GO:0031640 | killing of cells of another organism | 9/235 | 7.86 | 2.19E-06 | 4.06E-04 | GBP1/GBP5/C5/GBP3/S100A12/ARG1/LTF/DEFA3/DEFA4 | 9 |
| BP | GO:0141061 | disruption of cell in another organism | 9/235 | 7.86 | 2.19E-06 | 4.06E-04 | GBP1/GBP5/C5/GBP3/S100A12/ARG1/LTF/DEFA3/DEFA4 | 9 |
| BP | GO:1903131 | mononuclear cell differentiation | 20/235 | 3.34 | 2.72E-06 | 4.77E-04 | BATF2/CCR6/CD19/IL7R/GAS6/CD8A/TCF7/VNN1/TBX21/GATA3/CLEC4D/CCR9/IL23A/ZAP70/FOXP1/MS4A1/ZNF683/CEBPB/JUNB/CMTM7 | 20 |
| BP | GO:0141060 | disruption of anatomical structure in another organism | 9/235 | 7.30 | 4.05E-06 | 6.42E-04 | GBP1/GBP5/C5/GBP3/S100A12/ARG1/LTF/DEFA3/DEFA4 | 9 |
| BP | GO:0002237 | response to molecule of bacterial origin | 17/235 | 3.70 | 4.07E-06 | 6.42E-04 | GBP5/CD274/JAK2/FOXP1/ADM/TRIM6/GBP3/LY96/BPI/S100A8/MMP8/CEBPB/LTF/TH/CARD16/DEFA3/DEFA4 | 17 |
| BP | GO:0071706 | tumor necrosis factor superfamily cytokine production | 12/235 | 5.04 | 5.11E-06 | 7.33E-04 | CD274/GAS6/JAK2/LRRK2/IL23A/FOXP1/LY96/BPI/LPL/MMP8/LTF/NLRC3 | 12 |
| BP | GO:1903555 | regulation of tumor necrosis factor superfamily cytokine production | 12/235 | 5.04 | 5.11E-06 | 7.33E-04 | CD274/GAS6/JAK2/LRRK2/IL23A/FOXP1/LY96/BPI/LPL/MMP8/LTF/NLRC3 | 12 |
| BP | GO:0050832 | defense response to fungus | 7/235 | 10.04 | 5.86E-06 | 8.04E-04 | CLEC4D/S100A8/S100A12/ARG1/LTF/DEFA3/DEFA4 | 7 |
| BP | GO:0030098 | lymphocyte differentiation | 18/235 | 3.37 | 7.71E-06 | 1.01E-03 | CCR6/CD19/IL7R/GAS6/CD8A/TCF7/VNN1/TBX21/GATA3/CLEC4D/CCR9/IL23A/ZAP70/FOXP1/MS4A1/ZNF683/JUNB/CMTM7 | 18 |
| BP | GO:0033209 | tumor necrosis factor-mediated signaling pathway | 9/235 | 6.51 | 1.04E-05 | 1.31E-03 | AIM2/GAS6/TNFRSF25/JAK2/FAS/NAIP/STAT1/MMP8/CARD16 | 9 |
| BP | GO:0006958 | complement activation, classical pathway | 6/235 | 11.47 | 1.26E-05 | 1.45E-03 | SERPING1/C5/C1QB/C1QA/C1QC/C4BPA | 6 |
| BP | GO:0045860 | positive regulation of protein kinase activity | 14/235 | 3.99 | 1.28E-05 | 1.45E-03 | EPHA4/DOK7/GAS6/JAK2/LRRK2/NRG1/EGF/IL23A/TCL1A/NAB2/S100A12/LAT/LTF/MT3 | 14 |
| BP | GO:1902105 | regulation of leukocyte differentiation | 15/235 | 3.75 | 1.28E-05 | 1.45E-03 | IL7R/GAS6/TCF7/VNN1/TBX21/GATA3/IL23A/ZAP70/FOXP1/TNFAIP6/C1QC/ZNF683/CEBPB/LTF/JUNB | 15 |
| BP | GO:0002449 | lymphocyte mediated immunity | 16/235 | 3.49 | 1.61E-05 | 1.76E-03 | NCR3/CCR6/CD19/SERPING1/IL7R/CD8A/C5/TBX21/GATA3/C1QB/IL23A/C1QA/C1QC/IGHD/ARG1/C4BPA | 16 |
| BP | GO:0051347 | positive regulation of transferase activity | 17/235 | 3.30 | 1.83E-05 | 1.92E-03 | EPHA4/DOK7/CD19/LTK/GAS6/JAK2/LRRK2/NRG1/EGF/IL23A/TCL1A/NAB2/S100A12/LAT/LTF/FGR/MT3 | 17 |
| BP | GO:0009620 | response to fungus | 7/235 | 8.39 | 1.96E-05 | 1.99E-03 | CLEC4D/S100A8/S100A12/ARG1/LTF/DEFA3/DEFA4 | 7 |
| BP | GO:0098883 | synapse pruning | 4/235 | 22.94 | 2.13E-05 | 2.02E-03 | EPHA4/C1QB/C1QA/C1QC | 4 |
| BP | GO:0032640 | tumor necrosis factor production | 11/235 | 4.75 | 2.24E-05 | 2.02E-03 | GAS6/JAK2/LRRK2/IL23A/FOXP1/LY96/BPI/LPL/MMP8/LTF/NLRC3 | 11 |
| BP | GO:0032680 | regulation of tumor necrosis factor production | 11/235 | 4.75 | 2.24E-05 | 2.02E-03 | GAS6/JAK2/LRRK2/IL23A/FOXP1/LY96/BPI/LPL/MMP8/LTF/NLRC3 | 11 |
| BP | GO:0006959 | humoral immune response | 13/235 | 4.05 | 2.24E-05 | 2.02E-03 | CCR6/SERPING1/C5/GATA3/C1QB/C1QA/MS4A1/C1QC/S100A12/LTF/C4BPA/DEFA3/DEFA4 | 13 |
| BP | GO:0001906 | cell killing | 12/235 | 4.32 | 2.43E-05 | 2.13E-03 | GBP1/GBP5/NCR3/IL7R/C5/IL23A/GBP3/S100A12/ARG1/LTF/DEFA3/DEFA4 | 12 |
| BP | GO:0002366 | leukocyte activation involved in immune response | 14/235 | 3.73 | 2.64E-05 | 2.25E-03 | CCR6/CD19/TBX21/GATA3/CLEC4D/CD177/IL23A/ANXA3/FOXP1/DYSF/ZNF683/LAT/FGR/JUNB | 14 |
| BP | GO:0002263 | cell activation involved in immune response | 14/235 | 3.69 | 3.05E-05 | 2.53E-03 | CCR6/CD19/TBX21/GATA3/CLEC4D/CD177/IL23A/ANXA3/FOXP1/DYSF/ZNF683/LAT/FGR/JUNB | 14 |
| BP | GO:0030217 | T cell differentiation | 14/235 | 3.66 | 3.28E-05 | 2.65E-03 | CCR6/IL7R/CD8A/TCF7/VNN1/TBX21/GATA3/CLEC4D/CCR9/IL23A/ZAP70/FOXP1/ZNF683/JUNB | 14 |
| BP | GO:0032612 | interleukin-1 production | 9/235 | 5.56 | 3.69E-05 | 2.84E-03 | GBP5/AIM2/GAS6/JAK2/FOXP1/NAIP/LPL/IGHD/CARD16 | 9 |
| BP | GO:0032652 | regulation of interleukin-1 production | 9/235 | 5.56 | 3.69E-05 | 2.84E-03 | GBP5/AIM2/GAS6/JAK2/FOXP1/NAIP/LPL/IGHD/CARD16 | 9 |
| BP | GO:0050853 | B cell receptor signaling pathway | 7/235 | 7.30 | 4.87E-05 | 3.66E-03 | BLK/CD19/CD22/FOXP1/MS4A1/BMX/IGHD | 7 |
| BP | GO:0002455 | humoral immune response mediated by circulating immunoglobulin | 6/235 | 8.76 | 6.09E-05 | 4.47E-03 | SERPING1/C5/C1QB/C1QA/C1QC/C4BPA | 6 |
| BP | GO:0002833 | positive regulation of response to biotic stimulus | 15/235 | 3.26 | 6.41E-05 | 4.60E-03 | GBP5/NCR3/CD274/AIM2/NAIP/TRIM6/TIFA/PLSCR1/LY96/ALPK1/ARG1/LTF/NLRC3/CTSS/RBM47 | 15 |
| BP | GO:1903706 | regulation of hemopoiesis | 16/235 | 3.08 | 7.24E-05 | 5.08E-03 | IL7R/GAS6/TCF7/VNN1/TBX21/GATA3/IL23A/ZAP70/FOXP1/TNFAIP6/C1QC/STAT1/ZNF683/CEBPB/LTF/JUNB | 16 |
| BP | GO:2001235 | positive regulation of apoptotic signaling pathway | 9/235 | 4.98 | 8.64E-05 | 5.78E-03 | TNFSF10/JAK2/VNN1/FAS/S100A8/G0S2/PLEKHF1/MCL1/CTSS | 9 |
| BP | GO:0045088 | regulation of innate immune response | 16/235 | 3.02 | 9.04E-05 | 5.78E-03 | GBP5/NCR3/AIM2/SERPING1/NAIP/TRIM6/TIFA/PLSCR1/LY96/ALPK1/ARG1/LTF/FGR/NLRC3/CTSS/RBM47 | 16 |
| BP | GO:0050727 | regulation of inflammatory response | 16/235 | 3.02 | 9.04E-05 | 5.78E-03 | AIM2/JAK2/GATA3/LRRK2/IL23A/FOXP1/TNFAIP6/NAIP/LPL/S100A8/S100A12/MMP8/CEBPB/FGR/NLRC3/CARD16 | 16 |
| BP | GO:0070269 | pyroptosis | 5/235 | 10.85 | 9.04E-05 | 5.78E-03 | GBP1/GBP5/AIM2/NAIP/GBP3 | 5 |
| BP | GO:0050863 | regulation of T cell activation | 15/235 | 3.16 | 9.16E-05 | 5.78E-03 | CD274/IL7R/TIGIT/JAK2/TCF7/VNN1/TBX21/GATA3/IL23A/ZAP70/ZNF683/LAT/ARG1/CEBPB/JUNB | 15 |
| BP | GO:0046634 | regulation of alpha-beta T cell activation | 8/235 | 5.54 | 1.03E-04 | 6.36E-03 | CD274/JAK2/TBX21/GATA3/IL23A/ZAP70/ZNF683/JUNB | 8 |
| BP | GO:0009615 | response to virus | 16/235 | 2.95 | 1.21E-04 | 7.37E-03 | GBP1/GBP5/AIM2/JAK2/TBX21/GATA3/IL23A/TRIM6/STAT1/PLSCR1/GBP3/IFIT3/FGR/NLRC3/IRF2/DEFA3 | 16 |
| BP | GO:0050729 | positive regulation of inflammatory response | 9/235 | 4.75 | 1.24E-04 | 7.38E-03 | AIM2/LRRK2/IL23A/NAIP/LPL/S100A8/S100A12/MMP8/CEBPB | 9 |
| BP | GO:0043122 | regulation of canonical NF-kappaB signal transduction | 12/235 | 3.64 | 1.28E-04 | 7.48E-03 | TNFSF10/CLEC4D/SECTM1/TRIM6/STAT1/TIFA/ALPK1/S100A12/S100B/LTF/NLRC3/CARD16 | 12 |
| BP | GO:0071222 | cellular response to lipopolysaccharide | 11/235 | 3.91 | 1.30E-04 | 7.48E-03 | GBP5/CD274/JAK2/GBP3/LY96/BPI/MMP8/LTF/CARD16/DEFA3/DEFA4 | 11 |
| BP | GO:0052547 | regulation of peptidase activity | 13/235 | 3.35 | 1.55E-04 | 8.72E-03 | EPHA4/AIM2/SERPING1/GAS6/TNFSF10/JAK2/FAS/NAIP/S100A8/SPINK2/LTF/CARD16/CTSS | 13 |
| BP | GO:0043123 | positive regulation of canonical NF-kappaB signal transduction | 10/235 | 4.10 | 1.80E-04 | 9.95E-03 | TNFSF10/CLEC4D/SECTM1/TRIM6/TIFA/ALPK1/S100A12/S100B/LTF/CARD16 | 10 |
| BP | GO:0006956 | complement activation | 6/235 | 7.19 | 1.85E-04 | 1.00E-02 | SERPING1/C5/C1QB/C1QA/C1QC/C4BPA | 6 |
| BP | GO:0032814 | regulation of natural killer cell activation | 5/235 | 9.34 | 1.88E-04 | 1.00E-02 | GAS6/JAK2/IL23A/ZNF683/FGR | 5 |
| BP | GO:0071219 | cellular response to molecule of bacterial origin | 11/235 | 3.70 | 2.12E-04 | 1.12E-02 | GBP5/CD274/JAK2/GBP3/LY96/BPI/MMP8/LTF/CARD16/DEFA3/DEFA4 | 11 |
| BP | GO:0030101 | natural killer cell activation | 7/235 | 5.68 | 2.39E-04 | 1.24E-02 | NCR3/GAS6/JAK2/GATA3/IL23A/ZNF683/FGR | 7 |
| BP | GO:0016064 | immunoglobulin mediated immune response | 10/235 | 3.92 | 2.58E-04 | 1.31E-02 | CCR6/CD19/SERPING1/C5/TBX21/C1QB/C1QA/C1QC/IGHD/C4BPA | 10 |
| BP | GO:0052548 | regulation of endopeptidase activity | 12/235 | 3.35 | 2.77E-04 | 1.39E-02 | EPHA4/AIM2/SERPING1/GAS6/TNFSF10/JAK2/FAS/NAIP/S100A8/SPINK2/LTF/CARD16 | 12 |
| BP | GO:0019724 | B cell mediated immunity | 10/235 | 3.86 | 2.90E-04 | 1.43E-02 | CCR6/CD19/SERPING1/C5/TBX21/C1QB/C1QA/C1QC/IGHD/C4BPA | 10 |
| BP | GO:0032732 | positive regulation of interleukin-1 production | 6/235 | 6.60 | 2.97E-04 | 1.44E-02 | GBP5/AIM2/JAK2/NAIP/LPL/IGHD | 6 |
| BP | GO:0002696 | positive regulation of leukocyte activation | 14/235 | 2.96 | 3.10E-04 | 1.47E-02 | CD274/IL7R/GAS6/JAK2/VNN1/TBX21/GATA3/LRRK2/CLEC4D/CD177/IL23A/ZAP70/MMP8/FGR | 14 |
| BP | GO:0019722 | calcium-mediated signaling | 10/235 | 3.82 | 3.13E-04 | 1.47E-02 | GBP1/CXCR3/CCR6/LRRK2/CCR9/CD22/ZAP70/CXCR5/LAT/CLIC2 | 10 |
| BP | GO:0046637 | regulation of alpha-beta T cell differentiation | 6/235 | 6.51 | 3.20E-04 | 1.48E-02 | TBX21/GATA3/IL23A/ZAP70/ZNF683/JUNB | 6 |
| BP | GO:0150146 | cell junction disassembly | 4/235 | 11.90 | 3.29E-04 | 1.50E-02 | EPHA4/C1QB/C1QA/C1QC | 4 |
| BP | GO:0045619 | regulation of lymphocyte differentiation | 10/235 | 3.79 | 3.38E-04 | 1.51E-02 | IL7R/GAS6/TCF7/VNN1/TBX21/GATA3/IL23A/ZAP70/ZNF683/JUNB | 10 |
| BP | GO:0030099 | myeloid cell differentiation | 15/235 | 2.80 | 3.40E-04 | 1.51E-02 | BATF2/JAK2/GATA3/IL23A/FOXP1/TNFAIP6/C1QC/STAT1/RHAG/SNX10/MAF/CEBPB/LTF/JUNB/BPGM | 15 |
| BP | GO:0030316 | osteoclast differentiation | 7/235 | 5.30 | 3.64E-04 | 1.60E-02 | IL23A/FOXP1/TNFAIP6/SNX10/CEBPB/LTF/JUNB | 7 |
| BP | GO:0042832 | defense response to protozoan | 4/235 | 11.47 | 3.80E-04 | 1.64E-02 | GBP1/BATF2/ARG1/BPGM | 4 |
| BP | GO:0045089 | positive regulation of innate immune response | 13/235 | 3.04 | 3.87E-04 | 1.65E-02 | GBP5/NCR3/AIM2/NAIP/TRIM6/TIFA/PLSCR1/LY96/ALPK1/LTF/NLRC3/CTSS/RBM47 | 13 |
| BP | GO:0002697 | regulation of immune effector process | 14/235 | 2.89 | 3.92E-04 | 1.65E-02 | NCR3/SERPING1/IL7R/TBX21/GATA3/CD177/IL23A/CD22/TRIM6/ZNF683/ARG1/FGR/JUNB/C4BPA | 14 |
| BP | GO:0007249 | canonical NF-kappaB signal transduction | 12/235 | 3.21 | 4.00E-04 | 1.66E-02 | TNFSF10/CLEC4D/SECTM1/TRIM6/STAT1/TIFA/ALPK1/S100A12/S100B/LTF/NLRC3/CARD16 | 12 |
| BP | GO:0045580 | regulation of T cell differentiation | 9/235 | 3.99 | 4.55E-04 | 1.82E-02 | IL7R/TCF7/VNN1/TBX21/GATA3/IL23A/ZAP70/ZNF683/JUNB | 9 |
| BP | GO:0046631 | alpha-beta T cell activation | 9/235 | 3.99 | 4.55E-04 | 1.82E-02 | CD274/JAK2/TBX21/GATA3/IL23A/ZAP70/FOXP1/ZNF683/JUNB | 9 |
| BP | GO:0042116 | macrophage activation | 7/235 | 5.11 | 4.56E-04 | 1.82E-02 | JAK2/LRRK2/C1QA/FOXP1/DYSF/BPI/MMP8 | 7 |
| BP | GO:0050867 | positive regulation of cell activation | 14/235 | 2.84 | 4.68E-04 | 1.84E-02 | CD274/IL7R/GAS6/JAK2/VNN1/TBX21/GATA3/LRRK2/CLEC4D/CD177/IL23A/ZAP70/MMP8/FGR | 14 |
| BP | GO:0032611 | interleukin-1 beta production | 7/235 | 5.06 | 4.81E-04 | 1.85E-02 | GBP5/AIM2/JAK2/FOXP1/NAIP/LPL/CARD16 | 7 |
| BP | GO:0032651 | regulation of interleukin-1 beta production | 7/235 | 5.06 | 4.81E-04 | 1.85E-02 | GBP5/AIM2/JAK2/FOXP1/NAIP/LPL/CARD16 | 7 |
| BP | GO:0035745 | T-helper 2 cell cytokine production | 3/235 | 18.53 | 4.97E-04 | 1.87E-02 | TBX21/GATA3/ARG1 | 3 |
| BP | GO:2000551 | regulation of T-helper 2 cell cytokine production | 3/235 | 18.53 | 4.97E-04 | 1.87E-02 | TBX21/GATA3/ARG1 | 3 |
| BP | GO:0071216 | cellular response to biotic stimulus | 11/235 | 3.33 | 5.11E-04 | 1.90E-02 | GBP5/CD274/JAK2/GBP3/LY96/BPI/MMP8/LTF/CARD16/DEFA3/DEFA4 | 11 |
| BP | GO:0001562 | response to protozoan | 4/235 | 10.36 | 5.66E-04 | 2.08E-02 | GBP1/BATF2/ARG1/BPGM | 4 |
| BP | GO:0002292 | T cell differentiation involved in immune response | 6/235 | 5.74 | 6.33E-04 | 2.27E-02 | TBX21/GATA3/CLEC4D/IL23A/FOXP1/JUNB | 6 |
| BP | GO:0002230 | positive regulation of defense response to virus by host | 4/235 | 10.04 | 6.41E-04 | 2.27E-02 | AIM2/IL23A/TRIM6/STAT1 | 4 |
| BP | GO:2000316 | regulation of T-helper 17 type immune response | 4/235 | 10.04 | 6.41E-04 | 2.27E-02 | JAK2/TBX21/IL23A/JUNB | 4 |
| BP | GO:0002822 | regulation of adaptive immune response based on somatic recombination of immune receptors built from immunoglobulin superfamily domains | 9/235 | 3.74 | 7.22E-04 | 2.53E-02 | CD274/IL7R/JAK2/TBX21/GATA3/IL23A/ARG1/JUNB/C4BPA | 9 |
| BP | GO:0002573 | myeloid leukocyte differentiation | 10/235 | 3.42 | 7.54E-04 | 2.61E-02 | BATF2/GATA3/IL23A/FOXP1/TNFAIP6/C1QC/SNX10/CEBPB/LTF/JUNB | 10 |
| BP | GO:2000116 | regulation of cysteine-type endopeptidase activity | 9/235 | 3.71 | 7.77E-04 | 2.67E-02 | AIM2/GAS6/TNFSF10/JAK2/FAS/NAIP/S100A8/LTF/CARD16 | 9 |
| BP | GO:0035456 | response to interferon-beta | 4/235 | 9.45 | 8.11E-04 | 2.75E-02 | AIM2/TRIM6/STAT1/PLSCR1 | 4 |
| BP | GO:0046632 | alpha-beta T cell differentiation | 7/235 | 4.53 | 9.31E-04 | 3.13E-02 | TBX21/GATA3/IL23A/ZAP70/FOXP1/ZNF683/JUNB | 7 |
| BP | GO:0051770 | positive regulation of nitric-oxide synthase biosynthetic process | 3/235 | 15.06 | 9.47E-04 | 3.15E-02 | JAK2/LRRK2/STAT1 | 3 |
| BP | GO:0051607 | defense response to virus | 12/235 | 2.88 | 1.03E-03 | 3.37E-02 | GBP1/GBP5/AIM2/IL23A/TRIM6/STAT1/PLSCR1/GBP3/IFIT3/NLRC3/IRF2/DEFA3 | 12 |
| BP | GO:0032731 | positive regulation of interleukin-1 beta production | 5/235 | 6.48 | 1.04E-03 | 3.39E-02 | GBP5/AIM2/JAK2/NAIP/LPL | 5 |
| BP | GO:0140546 | defense response to symbiont | 12/235 | 2.88 | 1.05E-03 | 3.39E-02 | GBP1/GBP5/AIM2/IL23A/TRIM6/STAT1/PLSCR1/GBP3/IFIT3/NLRC3/IRF2/DEFA3 | 12 |
| BP | GO:0051099 | positive regulation of binding | 7/235 | 4.43 | 1.07E-03 | 3.41E-02 | EPHA4/BLK/GATA3/LRRK2/EGF/TRIM6/CLIC2 | 7 |
| BP | GO:0051896 | regulation of phosphatidylinositol 3-kinase/protein kinase B signal transduction | 10/235 | 3.24 | 1.14E-03 | 3.58E-02 | HPSE/AIM2/LTK/GAS6/JAK2/GATA3/EGF/PPP1R16B/FGR/NLRC3 | 10 |
| BP | GO:0002456 | T cell mediated immunity | 7/235 | 4.36 | 1.17E-03 | 3.67E-02 | IL7R/CD8A/TBX21/GATA3/IL23A/ARG1/C4BPA | 7 |
| BP | GO:0002819 | regulation of adaptive immune response | 9/235 | 3.47 | 1.22E-03 | 3.79E-02 | CD274/IL7R/JAK2/TBX21/GATA3/IL23A/ARG1/JUNB/C4BPA | 9 |
| BP | GO:1990845 | adaptive thermogenesis | 8/235 | 3.82 | 1.24E-03 | 3.79E-02 | GADD45G/JAK2/GRB10/G0S2/LCN2/S100B/CEBPB/ACVR2B | 8 |
| BP | GO:0002285 | lymphocyte activation involved in immune response | 9/235 | 3.39 | 1.44E-03 | 4.36E-02 | CCR6/CD19/TBX21/GATA3/CLEC4D/IL23A/FOXP1/ZNF683/JUNB | 9 |
| BP | GO:0050900 | leukocyte migration | 13/235 | 2.64 | 1.45E-03 | 4.36E-02 | CXCR3/CCR6/GAS6/C5/TBX21/GATA3/CD177/IL23A/ZAP70/TNFAIP6/CXCR5/S100A8/S100A12 | 13 |
| BP | GO:0035743 | CD4-positive, alpha-beta T cell cytokine production | 3/235 | 12.68 | 1.59E-03 | 4.73E-02 | TBX21/GATA3/ARG1 | 3 |
| BP | GO:0031214 | biomineral tissue development | 8/235 | 3.67 | 1.61E-03 | 4.73E-02 | FAM20A/GAS6/SNX10/CEBPB/LTF/FGR/ACVR2B/ZBTB40 | 8 |
| BP | GO:0120162 | positive regulation of cold-induced thermogenesis | 6/235 | 4.77 | 1.66E-03 | 4.84E-02 | GADD45G/JAK2/GRB10/G0S2/LCN2/CEBPB | 6 |
| BP | GO:0032733 | positive regulation of interleukin-10 production | 4/235 | 7.65 | 1.81E-03 | 5.19E-02 | CD274/TIGIT/IL23A/RBM47 | 4 |
| BP | GO:0140467 | integrated stress response signaling | 4/235 | 7.65 | 1.81E-03 | 5.19E-02 | BATF2/MAF/CEBPB/JUNB | 4 |
| BP | GO:0051767 | nitric-oxide synthase biosynthetic process | 3/235 | 12.04 | 1.86E-03 | 5.24E-02 | JAK2/LRRK2/STAT1 | 3 |
| BP | GO:0051769 | regulation of nitric-oxide synthase biosynthetic process | 3/235 | 12.04 | 1.86E-03 | 5.24E-02 | JAK2/LRRK2/STAT1 | 3 |
| BP | GO:0045622 | regulation of T-helper cell differentiation | 4/235 | 7.47 | 1.98E-03 | 5.52E-02 | TBX21/GATA3/IL23A/JUNB | 4 |
| BP | GO:0032760 | positive regulation of tumor necrosis factor production | 6/235 | 4.55 | 2.12E-03 | 5.79E-02 | JAK2/LRRK2/IL23A/LY96/LPL/MMP8 | 6 |
| BP | GO:0001659 | temperature homeostasis | 8/235 | 3.51 | 2.13E-03 | 5.79E-02 | GADD45G/NMU/JAK2/GRB10/G0S2/LCN2/CEBPB/ACVR2B | 8 |
| BP | GO:0046640 | regulation of alpha-beta T cell proliferation | 4/235 | 7.30 | 2.15E-03 | 5.79E-02 | CD274/JAK2/IL23A/ZAP70 | 4 |
| BP | GO:0050691 | regulation of defense response to virus by host | 4/235 | 7.30 | 2.15E-03 | 5.79E-02 | AIM2/IL23A/TRIM6/STAT1 | 4 |
| BP | GO:0042093 | T-helper cell differentiation | 5/235 | 5.50 | 2.17E-03 | 5.79E-02 | TBX21/GATA3/IL23A/FOXP1/JUNB | 5 |
| BP | GO:0006935 | chemotaxis | 14/235 | 2.40 | 2.29E-03 | 6.07E-02 | RAB13/CXCR3/CCR6/GAS6/C5/WNT3/NRG1/CCR9/IL23A/TNFAIP6/CXCR5/S100A8/S100A12/CMTM7 | 14 |
| BP | GO:0030574 | collagen catabolic process | 4/235 | 7.14 | 2.34E-03 | 6.16E-02 | MMP1/MMP8/FAP/CTSS | 4 |
| BP | GO:0007159 | leukocyte cell-cell adhesion | 13/235 | 2.49 | 2.38E-03 | 6.16E-02 | CD274/IL7R/TIGIT/JAK2/VNN1/TBX21/GATA3/CD177/IL23A/ZAP70/S100A8/ARG1/CEBPB | 13 |
| BP | GO:0042330 | taxis | 14/235 | 2.39 | 2.38E-03 | 6.16E-02 | RAB13/CXCR3/CCR6/GAS6/C5/WNT3/NRG1/CCR9/IL23A/TNFAIP6/CXCR5/S100A8/S100A12/CMTM7 | 14 |
| BP | GO:0002294 | CD4-positive, alpha-beta T cell differentiation involved in immune response | 5/235 | 5.35 | 2.44E-03 | 6.17E-02 | TBX21/GATA3/IL23A/FOXP1/JUNB | 5 |
| BP | GO:0015669 | gas transport | 3/235 | 10.95 | 2.47E-03 | 6.17E-02 | RHAG/BPGM/HBE1 | 3 |
| BP | GO:0021756 | striatum development | 3/235 | 10.95 | 2.47E-03 | 6.17E-02 | LRRK2/FOXP1/CNTNAP2 | 3 |
| BP | GO:2000319 | regulation of T-helper 17 cell differentiation | 3/235 | 10.95 | 2.47E-03 | 6.17E-02 | TBX21/IL23A/JUNB | 3 |
| BP | GO:0018108 | peptidyl-tyrosine phosphorylation | 10/235 | 2.91 | 2.50E-03 | 6.20E-02 | EPHA4/DOK7/BLK/LTK/JAK2/EGF/IL23A/ZAP70/FGR/NCL | 10 |
| BP | GO:1903557 | positive regulation of tumor necrosis factor superfamily cytokine production | 6/235 | 4.38 | 2.55E-03 | 6.28E-02 | JAK2/LRRK2/IL23A/LY96/LPL/MMP8 | 6 |
| BP | GO:0002287 | alpha-beta T cell activation involved in immune response | 5/235 | 5.28 | 2.59E-03 | 6.28E-02 | TBX21/GATA3/IL23A/FOXP1/JUNB | 5 |
| BP | GO:0002293 | alpha-beta T cell differentiation involved in immune response | 5/235 | 5.28 | 2.59E-03 | 6.28E-02 | TBX21/GATA3/IL23A/FOXP1/JUNB | 5 |
| BP | GO:0060326 | cell chemotaxis | 11/235 | 2.72 | 2.62E-03 | 6.29E-02 | RAB13/CXCR3/CCR6/GAS6/C5/CCR9/IL23A/TNFAIP6/CXCR5/S100A8/S100A12 | 11 |
| BP | GO:0018212 | peptidyl-tyrosine modification | 10/235 | 2.89 | 2.63E-03 | 6.29E-02 | EPHA4/DOK7/BLK/LTK/JAK2/EGF/IL23A/ZAP70/FGR/NCL | 10 |
| BP | GO:0046633 | alpha-beta T cell proliferation | 4/235 | 6.83 | 2.75E-03 | 6.47E-02 | CD274/JAK2/IL23A/ZAP70 | 4 |
| BP | GO:0072538 | T-helper 17 type immune response | 4/235 | 6.83 | 2.75E-03 | 6.47E-02 | JAK2/TBX21/IL23A/JUNB | 4 |
| BP | GO:0120161 | regulation of cold-induced thermogenesis | 7/235 | 3.75 | 2.77E-03 | 6.48E-02 | GADD45G/JAK2/GRB10/G0S2/LCN2/CEBPB/ACVR2B | 7 |
| BP | GO:0050855 | regulation of B cell receptor signaling pathway | 3/235 | 10.47 | 2.81E-03 | 6.52E-02 | CD19/CD22/FOXP1 | 3 |
| BP | GO:0031345 | negative regulation of cell projection organization | 8/235 | 3.35 | 2.86E-03 | 6.53E-02 | EPHA4/WNT3/LRRK2/FGF13/ZNF365/RAP1GAP/LIMK2/MT3 | 8 |
| BP | GO:0106106 | cold-induced thermogenesis | 7/235 | 3.72 | 2.88E-03 | 6.53E-02 | GADD45G/JAK2/GRB10/G0S2/LCN2/CEBPB/ACVR2B | 7 |
| BP | GO:0002221 | pattern recognition receptor signaling pathway | 9/235 | 3.06 | 2.89E-03 | 6.53E-02 | GBP5/AIM2/NAIP/TIFA/LY96/ALPK1/LTF/NLRC3/CTSS | 9 |
| BP | GO:2000514 | regulation of CD4-positive, alpha-beta T cell activation | 5/235 | 5.15 | 2.90E-03 | 6.53E-02 | CD274/TBX21/GATA3/IL23A/JUNB | 5 |
| BP | GO:0032102 | negative regulation of response to external stimulus | 14/235 | 2.34 | 2.93E-03 | 6.56E-02 | EPHA4/SERPING1/C5/GATA3/WNT3/TNFAIP6/PROS1/CNTNAP2/ARG1/LTF/FAP/FGR/NLRC3/CARD16 | 14 |
| BP | GO:0042551 | neuron maturation | 4/235 | 6.69 | 2.97E-03 | 6.60E-02 | SCARF1/LRRK2/C1QA/CNTNAP2 | 4 |
| BP | GO:0001818 | negative regulation of cytokine production | 12/235 | 2.53 | 3.07E-03 | 6.77E-02 | GBP1/CD274/GAS6/TIGIT/TBX21/GATA3/IL23A/BPI/ARG1/LTF/NLRC3/CARD16 | 12 |
| BP | GO:0001774 | microglial cell activation | 4/235 | 6.55 | 3.20E-03 | 6.97E-02 | JAK2/LRRK2/C1QA/MMP8 | 4 |
| BP | GO:0046427 | positive regulation of receptor signaling pathway via JAK-STAT | 4/235 | 6.55 | 3.20E-03 | 6.97E-02 | IL7R/JAK2/IL23A/CYP1B1 | 4 |
| BP | GO:1903556 | negative regulation of tumor necrosis factor superfamily cytokine production | 5/235 | 5.02 | 3.23E-03 | 6.99E-02 | CD274/GAS6/BPI/LTF/NLRC3 | 5 |
| BP | GO:0043491 | phosphatidylinositol 3-kinase/protein kinase B signal transduction | 10/235 | 2.77 | 3.56E-03 | 7.62E-02 | HPSE/AIM2/LTK/GAS6/JAK2/GATA3/EGF/PPP1R16B/FGR/NLRC3 | 10 |
| BP | GO:0046641 | positive regulation of alpha-beta T cell proliferation | 3/235 | 9.64 | 3.58E-03 | 7.62E-02 | JAK2/IL23A/ZAP70 | 3 |
| BP | GO:0150076 | neuroinflammatory response | 5/235 | 4.90 | 3.60E-03 | 7.62E-02 | JAK2/LRRK2/C1QA/MMP8/BPGM | 5 |
| BP | GO:0030100 | regulation of endocytosis | 10/235 | 2.76 | 3.64E-03 | 7.66E-02 | GAS6/LRRK2/CD177/EGF/CD22/PLSCR1/DYSF/SDCBP/FGR/BTBD9 | 10 |
| BP | GO:0035710 | CD4-positive, alpha-beta T cell activation | 6/235 | 4.05 | 3.77E-03 | 7.88E-02 | CD274/TBX21/GATA3/IL23A/FOXP1/JUNB | 6 |
| BP | GO:0071346 | cellular response to type II interferon | 6/235 | 4.01 | 3.93E-03 | 8.06E-02 | GBP1/GBP5/JAK2/STAT1/GBP3/ARG1 | 6 |
| BP | GO:0002269 | leukocyte activation involved in inflammatory response | 4/235 | 6.18 | 3.98E-03 | 8.06E-02 | JAK2/LRRK2/C1QA/MMP8 | 4 |
| BP | GO:0006968 | cellular defense response | 4/235 | 6.18 | 3.98E-03 | 8.06E-02 | CCR6/CCR9/LY96/KIR3DL2 | 4 |
| BP | GO:0032148 | activation of protein kinase B activity | 3/235 | 9.27 | 4.01E-03 | 8.06E-02 | GAS6/NRG1/MT3 | 3 |
| BP | GO:0032816 | positive regulation of natural killer cell activation | 3/235 | 9.27 | 4.01E-03 | 8.06E-02 | GAS6/JAK2/IL23A | 3 |
| BP | GO:0060333 | type II interferon-mediated signaling pathway | 3/235 | 9.27 | 4.01E-03 | 8.06E-02 | JAK2/STAT1/ARG1 | 3 |
| BP | GO:0002703 | regulation of leukocyte mediated immunity | 9/235 | 2.90 | 4.12E-03 | 8.22E-02 | NCR3/IL7R/TBX21/GATA3/CD177/IL23A/ARG1/FGR/C4BPA | 9 |
| BP | GO:0061515 | myeloid cell development | 5/235 | 4.72 | 4.20E-03 | 8.29E-02 | FOXP1/RHAG/CEBPB/LTF/BPGM | 5 |
| BP | GO:0007157 | heterophilic cell-cell adhesion via plasma membrane cell adhesion molecules | 4/235 | 6.06 | 4.26E-03 | 8.29E-02 | SCARF1/CEACAM8/CEACAM6/SCARF2 | 4 |
| BP | GO:0045058 | T cell selection | 4/235 | 6.06 | 4.26E-03 | 8.29E-02 | TBX21/GATA3/IL23A/ZAP70 | 4 |
| BP | GO:1904894 | positive regulation of receptor signaling pathway via STAT | 4/235 | 6.06 | 4.26E-03 | 8.29E-02 | IL7R/JAK2/IL23A/CYP1B1 | 4 |
| BP | GO:0043281 | regulation of cysteine-type endopeptidase activity involved in apoptotic process | 7/235 | 3.45 | 4.39E-03 | 8.40E-02 | GAS6/TNFSF10/JAK2/FAS/NAIP/S100A8/CARD16 | 7 |
| BP | GO:0002286 | T cell activation involved in immune response | 6/235 | 3.92 | 4.44E-03 | 8.40E-02 | TBX21/GATA3/CLEC4D/IL23A/FOXP1/JUNB | 6 |
| BP | GO:0006541 | glutamine metabolic process | 3/235 | 8.92 | 4.47E-03 | 8.40E-02 | GFPT2/NIT2/ASNS | 3 |
| BP | GO:0010804 | negative regulation of tumor necrosis factor-mediated signaling pathway | 3/235 | 8.92 | 4.47E-03 | 8.40E-02 | GAS6/NAIP/CARD16 | 3 |
| BP | GO:0035458 | cellular response to interferon-beta | 3/235 | 8.92 | 4.47E-03 | 8.40E-02 | AIM2/TRIM6/STAT1 | 3 |
| BP | GO:0042730 | fibrinolysis | 3/235 | 8.92 | 4.47E-03 | 8.40E-02 | SERPING1/PROS1/FAP | 3 |
| BP | GO:0070661 | leukocyte proliferation | 11/235 | 2.52 | 4.59E-03 | 8.55E-02 | CD274/CD19/IL7R/JAK2/IL23A/CD22/ZAP70/MS4A1/ARG1/CEBPB/JUNB | 11 |
| BP | GO:0002761 | regulation of myeloid leukocyte differentiation | 6/235 | 3.89 | 4.61E-03 | 8.55E-02 | IL23A/FOXP1/TNFAIP6/C1QC/CEBPB/LTF | 6 |
| BP | GO:0033077 | T cell differentiation in thymus | 5/235 | 4.61 | 4.63E-03 | 8.55E-02 | CCR6/IL7R/VNN1/GATA3/ZAP70 | 5 |
| BP | GO:0002698 | negative regulation of immune effector process | 6/235 | 3.85 | 4.80E-03 | 8.76E-02 | SERPING1/IL7R/TBX21/CD22/ARG1/C4BPA | 6 |
| BP | GO:0002758 | innate immune response-activating signaling pathway | 9/235 | 2.83 | 4.80E-03 | 8.76E-02 | GBP5/AIM2/NAIP/TIFA/LY96/ALPK1/LTF/NLRC3/CTSS | 9 |
| BP | GO:0032147 | activation of protein kinase activity | 5/235 | 4.56 | 4.86E-03 | 8.80E-02 | GAS6/JAK2/NRG1/IL23A/MT3 | 5 |
| BP | GO:0002227 | innate immune response in mucosa | 3/235 | 8.60 | 4.96E-03 | 8.80E-02 | LTF/DEFA3/DEFA4 | 3 |
| BP | GO:0021544 | subpallium development | 3/235 | 8.60 | 4.96E-03 | 8.80E-02 | LRRK2/FOXP1/CNTNAP2 | 3 |
| BP | GO:0031664 | regulation of lipopolysaccharide-mediated signaling pathway | 3/235 | 8.60 | 4.96E-03 | 8.80E-02 | LY96/LTF/CARD16 | 3 |
| BP | GO:0032703 | negative regulation of interleukin-2 production | 3/235 | 8.60 | 4.96E-03 | 8.80E-02 | GBP1/TBX21/GATA3 | 3 |
| BP | GO:0050829 | defense response to Gram-negative bacterium | 5/235 | 4.51 | 5.10E-03 | 8.99E-02 | IL23A/BPI/LTF/DEFA3/DEFA4 | 5 |
| BP | GO:0001836 | release of cytochrome c from mitochondria | 4/235 | 5.74 | 5.19E-03 | 9.04E-02 | TNFSF10/HRK/MCL1/BCL2A1 | 4 |
| BP | GO:0043370 | regulation of CD4-positive, alpha-beta T cell differentiation | 4/235 | 5.74 | 5.19E-03 | 9.04E-02 | TBX21/GATA3/IL23A/JUNB | 4 |
| BP | GO:0046651 | lymphocyte proliferation | 10/235 | 2.62 | 5.29E-03 | 9.17E-02 | CD274/CD19/IL7R/JAK2/IL23A/CD22/ZAP70/MS4A1/ARG1/CEBPB | 10 |
| BP | GO:0010951 | negative regulation of endopeptidase activity | 6/235 | 3.76 | 5.38E-03 | 9.28E-02 | SERPING1/GAS6/NAIP/SPINK2/LTF/CARD16 | 6 |
| BP | GO:0002710 | negative regulation of T cell mediated immunity | 3/235 | 8.31 | 5.49E-03 | 9.32E-02 | IL7R/TBX21/ARG1 | 3 |
| BP | GO:0051354 | negative regulation of oxidoreductase activity | 3/235 | 8.31 | 5.49E-03 | 9.32E-02 | NOSIP/HP/MT3 | 3 |
| BP | GO:0010952 | positive regulation of peptidase activity | 7/235 | 3.31 | 5.51E-03 | 9.32E-02 | EPHA4/AIM2/TNFSF10/JAK2/FAS/S100A8/CTSS | 7 |
| BP | GO:0061900 | glial cell activation | 4/235 | 5.63 | 5.53E-03 | 9.32E-02 | JAK2/LRRK2/C1QA/MMP8 | 4 |
| BP | GO:0071695 | anatomical structure maturation | 9/235 | 2.77 | 5.57E-03 | 9.35E-02 | SCARF1/CCR6/BAIAP3/GATA3/LRRK2/C1QA/CNTNAP2/SNX10/LTF | 9 |
| BP | GO:0001959 | regulation of cytokine-mediated signaling pathway | 7/235 | 3.29 | 5.68E-03 | 9.43E-02 | GAS6/NAIP/TRIM6/ARG1/MMP8/CARD16/RBM47 | 7 |
| BP | GO:0051897 | positive regulation of phosphatidylinositol 3-kinase/protein kinase B signal transduction | 7/235 | 3.29 | 5.68E-03 | 9.43E-02 | HPSE/LTK/GAS6/JAK2/GATA3/EGF/FGR | 7 |
| BP | GO:0051098 | regulation of binding | 9/235 | 2.76 | 5.71E-03 | 9.43E-02 | EPHA4/BLK/JAK2/GATA3/LRRK2/EGF/TRIM6/CARD16/CLIC2 | 9 |
| BP | GO:0045621 | positive regulation of lymphocyte differentiation | 6/235 | 3.71 | 5.80E-03 | 9.51E-02 | IL7R/GAS6/VNN1/GATA3/IL23A/ZAP70 | 6 |
| BP | GO:0002707 | negative regulation of lymphocyte mediated immunity | 4/235 | 5.54 | 5.88E-03 | 9.51E-02 | IL7R/TBX21/ARG1/C4BPA | 4 |
| BP | GO:0031102 | neuron projection regeneration | 4/235 | 5.54 | 5.88E-03 | 9.51E-02 | EPHA4/SCARF1/JAK2/ADM | 4 |
| BP | GO:0031663 | lipopolysaccharide-mediated signaling pathway | 4/235 | 5.54 | 5.88E-03 | 9.51E-02 | LY96/BPI/LTF/CARD16 | 4 |
| BP | GO:0010575 | positive regulation of vascular endothelial growth factor production | 3/235 | 8.03 | 6.04E-03 | 9.72E-02 | HPSE/C5/CYP1B1 | 3 |
| BP | GO:0051346 | negative regulation of hydrolase activity | 8/235 | 2.95 | 6.12E-03 | 9.78E-02 | SERPING1/GAS6/LRRK2/NAIP/SPINK2/LTF/CARD16/CRY2 | 8 |
| BP | GO:0043367 | CD4-positive, alpha-beta T cell differentiation | 5/235 | 4.32 | 6.14E-03 | 9.78E-02 | TBX21/GATA3/IL23A/FOXP1/JUNB | 5 |
| BP | GO:0032943 | mononuclear cell proliferation | 10/235 | 2.56 | 6.17E-03 | 9.78E-02 | CD274/CD19/IL7R/JAK2/IL23A/CD22/ZAP70/MS4A1/ARG1/CEBPB | 10 |
| BP | GO:0002823 | negative regulation of adaptive immune response based on somatic recombination of immune receptors built from immunoglobulin superfamily domains | 4/235 | 5.44 | 6.25E-03 | 9.85E-02 | IL7R/TBX21/ARG1/C4BPA | 4 |
| BP | GO:0030879 | mammary gland development | 6/235 | 3.62 | 6.46E-03 | 9.97E-02 | JAK2/GATA3/WNT3/NRG1/EGF/CEBPB | 6 |
| BP | GO:0032819 | positive regulation of natural killer cell proliferation | 2/235 | 16.06 | 6.51E-03 | 9.97E-02 | JAK2/IL23A | 2 |
| BP | GO:0071287 | cellular response to manganese ion | 2/235 | 16.06 | 6.51E-03 | 9.97E-02 | LRRK2/TH | 2 |
| BP | GO:0140975 | disruption of cellular anatomical structure in another organism | 2/235 | 16.06 | 6.51E-03 | 9.97E-02 | DEFA3/DEFA4 | 2 |
| BP | GO:1903980 | positive regulation of microglial cell activation | 2/235 | 16.06 | 6.51E-03 | 9.97E-02 | LRRK2/MMP8 | 2 |
| BP | GO:2000510 | positive regulation of dendritic cell chemotaxis | 2/235 | 16.06 | 6.51E-03 | 9.97E-02 | CCR6/GAS6 | 2 |
| BP | GO:0042744 | hydrogen peroxide catabolic process | 3/235 | 7.77 | 6.63E-03 | 1.00E-01 | HP/HBE1/MT3 | 3 |
| BP | GO:0001961 | positive regulation of cytokine-mediated signaling pathway | 4/235 | 5.35 | 6.63E-03 | 1.00E-01 | GAS6/TRIM6/MMP8/RBM47 | 4 |
| BP | GO:0010803 | regulation of tumor necrosis factor-mediated signaling pathway | 4/235 | 5.35 | 6.63E-03 | 1.00E-01 | GAS6/NAIP/MMP8/CARD16 | 4 |
| BP | GO:0007596 | blood coagulation | 8/235 | 2.87 | 7.16E-03 | 1.08E-01 | HPSE/SERPING1/GAS6/JAK2/PROS1/PLSCR1/FUNDC2/FAP | 8 |
| BP | GO:0050688 | regulation of defense response to virus | 5/235 | 4.14 | 7.32E-03 | 1.09E-01 | AIM2/IL23A/TRIM6/STAT1/NLRC3 | 5 |
| BP | GO:0010466 | negative regulation of peptidase activity | 6/235 | 3.52 | 7.44E-03 | 1.10E-01 | SERPING1/GAS6/NAIP/SPINK2/LTF/CARD16 | 6 |
| BP | GO:0022617 | extracellular matrix disassembly | 4/235 | 5.18 | 7.44E-03 | 1.10E-01 | MMP1/MMP8/FAP/CTSS | 4 |
| BP | GO:0002275 | myeloid cell activation involved in immune response | 5/235 | 4.10 | 7.63E-03 | 1.13E-01 | CD177/ANXA3/DYSF/LAT/FGR | 5 |
| BP | GO:0008631 | intrinsic apoptotic signaling pathway in response to oxidative stress | 4/235 | 5.10 | 7.87E-03 | 1.13E-01 | JAK2/VNN1/CYP1B1/MCL1 | 4 |
| BP | GO:0002725 | negative regulation of T cell cytokine production | 2/235 | 14.60 | 7.89E-03 | 1.13E-01 | TBX21/ARG1 | 2 |
| BP | GO:0033089 | positive regulation of T cell differentiation in thymus | 2/235 | 14.60 | 7.89E-03 | 1.13E-01 | IL7R/VNN1 | 2 |
| BP | GO:0051133 | regulation of NK T cell activation | 2/235 | 14.60 | 7.89E-03 | 1.13E-01 | JAK2/IL23A | 2 |
| BP | GO:0072178 | nephric duct morphogenesis | 2/235 | 14.60 | 7.89E-03 | 1.13E-01 | EPHA4/GATA3 | 2 |
| BP | GO:0048799 | animal organ maturation | 3/235 | 7.30 | 7.90E-03 | 1.13E-01 | GATA3/SNX10/LTF | 3 |
| BP | GO:0072539 | T-helper 17 cell differentiation | 3/235 | 7.30 | 7.90E-03 | 1.13E-01 | TBX21/IL23A/JUNB | 3 |
| BP | GO:0002218 | activation of innate immune response | 9/235 | 2.62 | 7.92E-03 | 1.13E-01 | GBP5/AIM2/NAIP/TIFA/LY96/ALPK1/LTF/NLRC3/CTSS | 9 |
| BP | GO:0002709 | regulation of T cell mediated immunity | 5/235 | 4.06 | 7.96E-03 | 1.13E-01 | IL7R/TBX21/GATA3/IL23A/ARG1 | 5 |
| BP | GO:0060759 | regulation of response to cytokine stimulus | 7/235 | 3.07 | 8.13E-03 | 1.14E-01 | GAS6/NAIP/TRIM6/ARG1/MMP8/CARD16/RBM47 | 7 |
| BP | GO:0050817 | coagulation | 8/235 | 2.81 | 8.14E-03 | 1.14E-01 | HPSE/SERPING1/GAS6/JAK2/PROS1/PLSCR1/FUNDC2/FAP | 8 |
| BP | GO:0010977 | negative regulation of neuron projection development | 6/235 | 3.44 | 8.24E-03 | 1.14E-01 | EPHA4/WNT3/LRRK2/FGF13/ZNF365/MT3 | 6 |
| BP | GO:0042113 | B cell activation | 9/235 | 2.60 | 8.29E-03 | 1.14E-01 | CCR6/CD19/IL7R/TBX21/CD22/ZAP70/MS4A1/CXCR5/CMTM7 | 9 |
| BP | GO:0002820 | negative regulation of adaptive immune response | 4/235 | 5.02 | 8.31E-03 | 1.14E-01 | IL7R/TBX21/ARG1/C4BPA | 4 |
| BP | GO:0031100 | animal organ regeneration | 4/235 | 5.02 | 8.31E-03 | 1.14E-01 | GAS6/ANXA3/ADM/CEBPB | 4 |
| BP | GO:2001244 | positive regulation of intrinsic apoptotic signaling pathway | 4/235 | 5.02 | 8.31E-03 | 1.14E-01 | VNN1/S100A8/PLEKHF1/MCL1 | 4 |
| BP | GO:0007599 | hemostasis | 8/235 | 2.78 | 8.55E-03 | 1.16E-01 | HPSE/SERPING1/GAS6/JAK2/PROS1/PLSCR1/FUNDC2/FAP | 8 |
| BP | GO:0002828 | regulation of type 2 immune response | 3/235 | 7.09 | 8.59E-03 | 1.16E-01 | TBX21/GATA3/ARG1 | 3 |
| BP | GO:0021700 | developmental maturation | 10/235 | 2.43 | 8.61E-03 | 1.16E-01 | SCARF1/CCR6/BAIAP3/GATA3/LRRK2/C1QA/CNTNAP2/SNX10/LTF/ATP6V1C1 | 10 |
| BP | GO:1903037 | regulation of leukocyte cell-cell adhesion | 11/235 | 2.31 | 8.64E-03 | 1.16E-01 | CD274/IL7R/TIGIT/JAK2/VNN1/TBX21/GATA3/IL23A/ZAP70/ARG1/CEBPB | 11 |
| BP | GO:0032613 | interleukin-10 production | 4/235 | 4.94 | 8.77E-03 | 1.17E-01 | CD274/TIGIT/IL23A/RBM47 | 4 |
| BP | GO:0032653 | regulation of interleukin-10 production | 4/235 | 4.94 | 8.77E-03 | 1.17E-01 | CD274/TIGIT/IL23A/RBM47 | 4 |
| BP | GO:2000117 | negative regulation of cysteine-type endopeptidase activity | 4/235 | 4.94 | 8.77E-03 | 1.17E-01 | GAS6/NAIP/LTF/CARD16 | 4 |
| BP | GO:0034341 | response to type II interferon | 6/235 | 3.37 | 9.09E-03 | 1.17E-01 | GBP1/GBP5/JAK2/STAT1/GBP3/ARG1 | 6 |
| BP | GO:0050854 | regulation of antigen receptor-mediated signaling pathway | 4/235 | 4.87 | 9.25E-03 | 1.17E-01 | GBP1/CD19/CD22/FOXP1 | 4 |
| BP | GO:0061180 | mammary gland epithelium development | 4/235 | 4.87 | 9.25E-03 | 1.17E-01 | JAK2/GATA3/WNT3/CEBPB | 4 |
| BP | GO:0002446 | neutrophil mediated immunity | 3/235 | 6.88 | 9.31E-03 | 1.17E-01 | CD177/ANXA3/ARG1 | 3 |
| BP | GO:0030947 | regulation of vascular endothelial growth factor receptor signaling pathway | 3/235 | 6.88 | 9.31E-03 | 1.17E-01 | TMEM204/GRB10/MT3 | 3 |
| BP | GO:0002706 | regulation of lymphocyte mediated immunity | 7/235 | 2.99 | 9.36E-03 | 1.17E-01 | NCR3/IL7R/TBX21/GATA3/IL23A/ARG1/C4BPA | 7 |
| BP | GO:0031223 | auditory behavior | 2/235 | 13.38 | 9.39E-03 | 1.17E-01 | SLC1A3/CNTNAP2 | 2 |
| BP | GO:0032817 | regulation of natural killer cell proliferation | 2/235 | 13.38 | 9.39E-03 | 1.17E-01 | JAK2/IL23A | 2 |
| BP | GO:0042117 | monocyte activation | 2/235 | 13.38 | 9.39E-03 | 1.17E-01 | FOXP1/DYSF | 2 |
| BP | GO:0043312 | neutrophil degranulation | 2/235 | 13.38 | 9.39E-03 | 1.17E-01 | CD177/ANXA3 | 2 |
| BP | GO:0044341 | sodium-dependent phosphate transport | 2/235 | 13.38 | 9.39E-03 | 1.17E-01 | CEBPB/CRY2 | 2 |
| BP | GO:0048755 | branching morphogenesis of a nerve | 2/235 | 13.38 | 9.39E-03 | 1.17E-01 | LRRK2/FGF13 | 2 |
| BP | GO:0072182 | regulation of nephron tubule epithelial cell differentiation | 2/235 | 13.38 | 9.39E-03 | 1.17E-01 | GATA3/STAT1 | 2 |
| BP | GO:0072488 | ammonium transmembrane transport | 2/235 | 13.38 | 9.39E-03 | 1.17E-01 | RHAG/RHCE | 2 |
| BP | GO:2000508 | regulation of dendritic cell chemotaxis | 2/235 | 13.38 | 9.39E-03 | 1.17E-01 | CCR6/GAS6 | 2 |
| BP | GO:2001267 | regulation of cysteine-type endopeptidase activity involved in apoptotic signaling pathway | 2/235 | 13.38 | 9.39E-03 | 1.17E-01 | JAK2/FAS | 2 |
| BP | GO:0050852 | T cell receptor signaling pathway | 6/235 | 3.35 | 9.39E-03 | 1.17E-01 | GBP1/CD8A/GATA3/ZAP70/LAT/CD8B | 6 |
| BP | GO:0045861 | negative regulation of proteolysis | 8/235 | 2.73 | 9.43E-03 | 1.17E-01 | EPHA4/SERPING1/GAS6/LRRK2/NAIP/SPINK2/LTF/CARD16 | 8 |
| BP | GO:0060760 | positive regulation of response to cytokine stimulus | 4/235 | 4.79 | 9.74E-03 | 1.20E-01 | GAS6/TRIM6/MMP8/RBM47 | 4 |
| BP | GO:0033687 | osteoblast proliferation | 3/235 | 6.69 | 1.01E-02 | 1.23E-01 | HPSE/LTF/JUNB | 3 |
| BP | GO:0031099 | regeneration | 7/235 | 2.94 | 1.02E-02 | 1.24E-01 | EPHA4/SCARF1/GAS6/JAK2/ANXA3/ADM/CEBPB | 7 |
| BP | GO:0030193 | regulation of blood coagulation | 4/235 | 4.72 | 1.03E-02 | 1.25E-01 | HPSE/SERPING1/PROS1/FAP | 4 |
| BP | GO:0071466 | cellular response to xenobiotic stimulus | 7/235 | 2.93 | 1.04E-02 | 1.26E-01 | AIM2/GAS6/CBR3/CMBL/CYP1B1/S100A12/TH | 7 |
| BP | GO:0046425 | regulation of receptor signaling pathway via JAK-STAT | 5/235 | 3.79 | 1.05E-02 | 1.26E-01 | IL7R/JAK2/EGF/IL23A/CYP1B1 | 5 |
| BP | GO:0030595 | leukocyte chemotaxis | 8/235 | 2.68 | 1.06E-02 | 1.26E-01 | CCR6/GAS6/C5/IL23A/TNFAIP6/CXCR5/S100A8/S100A12 | 8 |
| BP | GO:0002704 | negative regulation of leukocyte mediated immunity | 4/235 | 4.65 | 1.08E-02 | 1.26E-01 | IL7R/TBX21/ARG1/C4BPA | 4 |
| BP | GO:0045576 | mast cell activation | 4/235 | 4.65 | 1.08E-02 | 1.26E-01 | PLSCR1/S100A12/LAT/FGR | 4 |
| BP | GO:0045670 | regulation of osteoclast differentiation | 4/235 | 4.65 | 1.08E-02 | 1.26E-01 | IL23A/TNFAIP6/CEBPB/LTF | 4 |
| BP | GO:0140888 | interferon-mediated signaling pathway | 5/235 | 3.75 | 1.09E-02 | 1.26E-01 | JAK2/TRIM6/STAT1/ARG1/RBM47 | 5 |
| BP | GO:0015838 | amino-acid betaine transport | 2/235 | 12.35 | 1.10E-02 | 1.26E-01 | SLC22A4/SLC6A12 | 2 |
| BP | GO:0032823 | regulation of natural killer cell differentiation | 2/235 | 12.35 | 1.10E-02 | 1.26E-01 | GAS6/ZNF683 | 2 |
| BP | GO:0033690 | positive regulation of osteoblast proliferation | 2/235 | 12.35 | 1.10E-02 | 1.26E-01 | HPSE/LTF | 2 |
| BP | GO:0042492 | gamma-delta T cell differentiation | 2/235 | 12.35 | 1.10E-02 | 1.26E-01 | TCF7/CCR9 | 2 |
| BP | GO:0042976 | activation of Janus kinase activity | 2/235 | 12.35 | 1.10E-02 | 1.26E-01 | JAK2/IL23A | 2 |
| BP | GO:0044406 | adhesion of symbiont to host | 2/235 | 12.35 | 1.10E-02 | 1.26E-01 | GAS6/LTF | 2 |
| BP | GO:0045916 | negative regulation of complement activation | 2/235 | 12.35 | 1.10E-02 | 1.26E-01 | SERPING1/C4BPA | 2 |
| BP | GO:0051132 | NK T cell activation | 2/235 | 12.35 | 1.10E-02 | 1.26E-01 | JAK2/IL23A | 2 |
| BP | GO:0070486 | leukocyte aggregation | 2/235 | 12.35 | 1.10E-02 | 1.26E-01 | ZAP70/S100A8 | 2 |
| BP | GO:1901678 | iron coordination entity transport | 2/235 | 12.35 | 1.10E-02 | 1.26E-01 | FLVCR2/LCN2 | 2 |
| BP | GO:1900046 | regulation of hemostasis | 4/235 | 4.59 | 1.13E-02 | 1.29E-01 | HPSE/SERPING1/PROS1/FAP | 4 |
| BP | GO:0051092 | positive regulation of NF-kappaB transcription factor activity | 6/235 | 3.21 | 1.13E-02 | 1.29E-01 | AIM2/TRIM6/S100A8/S100A12/LTF/CARD16 | 6 |
| BP | GO:0002369 | T cell cytokine production | 3/235 | 6.34 | 1.17E-02 | 1.31E-01 | TBX21/GATA3/ARG1 | 3 |
| BP | GO:0002724 | regulation of T cell cytokine production | 3/235 | 6.34 | 1.17E-02 | 1.31E-01 | TBX21/GATA3/ARG1 | 3 |
| BP | GO:0002705 | positive regulation of leukocyte mediated immunity | 6/235 | 3.19 | 1.17E-02 | 1.31E-01 | NCR3/TBX21/GATA3/CD177/IL23A/ARG1 | 6 |
| BP | GO:0002444 | myeloid leukocyte mediated immunity | 5/235 | 3.65 | 1.22E-02 | 1.37E-01 | CD177/ANXA3/LAT/ARG1/FGR | 5 |
| BP | GO:0046777 | protein autophosphorylation | 7/235 | 2.82 | 1.25E-02 | 1.38E-01 | EPHA4/PASK/LTK/JAK2/LRRK2/BMX/FGR | 7 |
| BP | GO:0002385 | mucosal immune response | 3/235 | 6.18 | 1.25E-02 | 1.38E-01 | LTF/DEFA3/DEFA4 | 3 |
| BP | GO:0032350 | regulation of hormone metabolic process | 3/235 | 6.18 | 1.25E-02 | 1.38E-01 | GATA3/ADM/H6PD | 3 |
| BP | GO:0007638 | mechanosensory behavior | 2/235 | 11.47 | 1.27E-02 | 1.38E-01 | SLC1A3/CNTNAP2 | 2 |
| BP | GO:0009404 | toxin metabolic process | 2/235 | 11.47 | 1.27E-02 | 1.38E-01 | CYP1B1/TH | 2 |
| BP | GO:0031665 | negative regulation of lipopolysaccharide-mediated signaling pathway | 2/235 | 11.47 | 1.27E-02 | 1.38E-01 | LTF/CARD16 | 2 |
| BP | GO:0031958 | corticosteroid receptor signaling pathway | 2/235 | 11.47 | 1.27E-02 | 1.38E-01 | JAK2/CRY2 | 2 |
| BP | GO:0035630 | bone mineralization involved in bone maturation | 2/235 | 11.47 | 1.27E-02 | 1.38E-01 | SNX10/LTF | 2 |
| BP | GO:0042415 | norepinephrine metabolic process | 2/235 | 11.47 | 1.27E-02 | 1.38E-01 | GATA3/TH | 2 |
| BP | GO:0072160 | nephron tubule epithelial cell differentiation | 2/235 | 11.47 | 1.27E-02 | 1.38E-01 | GATA3/STAT1 | 2 |
| BP | GO:0050818 | regulation of coagulation | 4/235 | 4.40 | 1.31E-02 | 1.41E-01 | HPSE/SERPING1/PROS1/FAP | 4 |
| BP | GO:0051090 | regulation of DNA-binding transcription factor activity | 11/235 | 2.18 | 1.32E-02 | 1.41E-01 | AIM2/LRRK2/ANXA3/TRIM6/CYP1B1/S100A8/S100A12/LTF/NLRC3/CARD16/TAF3 | 11 |
| BP | GO:0009595 | detection of biotic stimulus | 3/235 | 6.02 | 1.34E-02 | 1.43E-01 | NAIP/LY96/FAP | 3 |
| BP | GO:0043368 | positive T cell selection | 3/235 | 6.02 | 1.34E-02 | 1.43E-01 | TBX21/IL23A/ZAP70 | 3 |
| BP | GO:0002695 | negative regulation of leukocyte activation | 7/235 | 2.78 | 1.35E-02 | 1.43E-01 | CD274/TIGIT/TBX21/BPI/ARG1/CEBPB/FGR | 7 |
| BP | GO:0010950 | positive regulation of endopeptidase activity | 6/235 | 3.09 | 1.36E-02 | 1.43E-01 | EPHA4/AIM2/TNFSF10/JAK2/FAS/S100A8 | 6 |
| BP | GO:0043388 | positive regulation of DNA binding | 3/235 | 5.88 | 1.44E-02 | 1.49E-01 | GATA3/EGF/TRIM6 | 3 |
| BP | GO:0050777 | negative regulation of immune response | 7/235 | 2.74 | 1.45E-02 | 1.49E-01 | SERPING1/IL7R/TBX21/ARG1/FGR/NLRC3/C4BPA | 7 |
| BP | GO:0001787 | natural killer cell proliferation | 2/235 | 10.71 | 1.46E-02 | 1.49E-01 | JAK2/IL23A | 2 |
| BP | GO:0002829 | negative regulation of type 2 immune response | 2/235 | 10.71 | 1.46E-02 | 1.49E-01 | TBX21/ARG1 | 2 |
| BP | GO:0015670 | carbon dioxide transport | 2/235 | 10.71 | 1.46E-02 | 1.49E-01 | RHAG/HBE1 | 2 |
| BP | GO:0015671 | oxygen transport | 2/235 | 10.71 | 1.46E-02 | 1.49E-01 | BPGM/HBE1 | 2 |
| BP | GO:0016322 | neuron remodeling | 2/235 | 10.71 | 1.46E-02 | 1.49E-01 | SCARF1/C1QA | 2 |
| BP | GO:0032352 | positive regulation of hormone metabolic process | 2/235 | 10.71 | 1.46E-02 | 1.49E-01 | GATA3/ADM | 2 |
| BP | GO:0046007 | negative regulation of activated T cell proliferation | 2/235 | 10.71 | 1.46E-02 | 1.49E-01 | CD274/ARG1 | 2 |
| BP | GO:0072176 | nephric duct development | 2/235 | 10.71 | 1.46E-02 | 1.49E-01 | EPHA4/GATA3 | 2 |
| BP | GO:0048640 | negative regulation of developmental growth | 5/235 | 3.49 | 1.46E-02 | 1.49E-01 | WNT3/FGF13/FOXP1/WWC3/MT3 | 5 |
| BP | GO:0045582 | positive regulation of T cell differentiation | 5/235 | 3.46 | 1.51E-02 | 1.53E-01 | IL7R/VNN1/GATA3/IL23A/ZAP70 | 5 |
| BP | GO:0006836 | neurotransmitter transport | 7/235 | 2.72 | 1.53E-02 | 1.55E-01 | BAIAP3/LRRK2/SLC22A4/SLC1A3/DYSF/SLC6A12/TH | 7 |
| BP | GO:0042092 | type 2 immune response | 3/235 | 5.74 | 1.53E-02 | 1.55E-01 | TBX21/GATA3/ARG1 | 3 |
| BP | GO:0002260 | lymphocyte homeostasis | 4/235 | 4.17 | 1.56E-02 | 1.58E-01 | AIM2/IL7R/FAS/LAT | 4 |
| BP | GO:0043410 | positive regulation of MAPK cascade | 12/235 | 2.03 | 1.60E-02 | 1.61E-01 | MARCO/GADD45G/GAS6/JAK2/LRRK2/NRG1/EGF/NAIP/S100A12/SDCBP/GADD45B/MT3 | 12 |
| BP | GO:0002251 | organ or tissue specific immune response | 3/235 | 5.60 | 1.63E-02 | 1.63E-01 | LTF/DEFA3/DEFA4 | 3 |
| BP | GO:0010453 | regulation of cell fate commitment | 3/235 | 5.60 | 1.63E-02 | 1.63E-01 | TBX21/IL23A/MBD3 | 3 |
| BP | GO:0072540 | T-helper 17 cell lineage commitment | 2/235 | 10.04 | 1.65E-02 | 1.63E-01 | TBX21/IL23A | 2 |
| BP | GO:2000318 | positive regulation of T-helper 17 type immune response | 2/235 | 10.04 | 1.65E-02 | 1.63E-01 | JAK2/IL23A | 2 |
| BP | GO:2000696 | regulation of epithelial cell differentiation involved in kidney development | 2/235 | 10.04 | 1.65E-02 | 1.63E-01 | GATA3/STAT1 | 2 |
| BP | GO:0007162 | negative regulation of cell adhesion | 9/235 | 2.32 | 1.66E-02 | 1.63E-01 | GBP1/EPHA4/CD274/TIGIT/JAK2/TBX21/CYP1B1/ARG1/CEBPB | 9 |
| BP | GO:0032609 | type II interferon production | 5/235 | 3.37 | 1.67E-02 | 1.63E-01 | CD274/GAS6/JAK2/GATA3/IL23A | 5 |
| BP | GO:0032649 | regulation of type II interferon production | 5/235 | 3.37 | 1.67E-02 | 1.63E-01 | CD274/GAS6/JAK2/GATA3/IL23A | 5 |
| BP | GO:1904892 | regulation of receptor signaling pathway via STAT | 5/235 | 3.37 | 1.67E-02 | 1.63E-01 | IL7R/JAK2/EGF/IL23A/CYP1B1 | 5 |
| BP | GO:0009064 | glutamine family amino acid metabolic process | 4/235 | 4.07 | 1.70E-02 | 1.66E-01 | GFPT2/ARG1/NIT2/ASNS | 4 |
| BP | GO:0032689 | negative regulation of type II interferon production | 3/235 | 5.47 | 1.74E-02 | 1.68E-01 | CD274/GAS6/GATA3 | 3 |
| BP | GO:0046006 | regulation of activated T cell proliferation | 3/235 | 5.47 | 1.74E-02 | 1.68E-01 | CD274/IL23A/ARG1 | 3 |
| BP | GO:0045637 | regulation of myeloid cell differentiation | 7/235 | 2.64 | 1.76E-02 | 1.70E-01 | IL23A/FOXP1/TNFAIP6/C1QC/STAT1/CEBPB/LTF | 7 |
| BP | GO:0071887 | leukocyte apoptotic process | 5/235 | 3.32 | 1.78E-02 | 1.71E-01 | CD274/IL7R/GAS6/FAS/FOXP1 | 5 |
| BP | GO:0048872 | homeostasis of number of cells | 9/235 | 2.29 | 1.79E-02 | 1.71E-01 | AIM2/IL7R/JAK2/GATA3/FAS/STAT1/RHAG/LAT/BPGM | 9 |
| BP | GO:0030518 | intracellular steroid hormone receptor signaling pathway | 5/235 | 3.29 | 1.84E-02 | 1.76E-01 | JAK2/FOXP1/DDX54/DEFA3/CRY2 | 5 |
| BP | GO:0043299 | leukocyte degranulation | 4/235 | 3.97 | 1.85E-02 | 1.76E-01 | CD177/ANXA3/LAT/FGR | 4 |
| BP | GO:0002921 | negative regulation of humoral immune response | 2/235 | 9.45 | 1.86E-02 | 1.76E-01 | SERPING1/C4BPA | 2 |
| BP | GO:0006491 | N-glycan processing | 2/235 | 9.45 | 1.86E-02 | 1.76E-01 | ST8SIA1/MAN2A2 | 2 |
| BP | GO:0097193 | intrinsic apoptotic signaling pathway | 9/235 | 2.27 | 1.89E-02 | 1.78E-01 | JAK2/VNN1/LRRK2/CYP1B1/S100A8/CEBPB/PLEKHF1/MCL1/BCL2A1 | 9 |
| BP | GO:0030282 | bone mineralization | 5/235 | 3.26 | 1.90E-02 | 1.79E-01 | SNX10/LTF/FGR/ACVR2B/ZBTB40 | 5 |
| BP | GO:0070227 | lymphocyte apoptotic process | 4/235 | 3.92 | 1.93E-02 | 1.81E-01 | CD274/IL7R/FAS/FOXP1 | 4 |
| BP | GO:0051250 | negative regulation of lymphocyte activation | 6/235 | 2.85 | 1.94E-02 | 1.81E-01 | CD274/TIGIT/TBX21/ARG1/CEBPB/FGR | 6 |
| BP | GO:0097028 | dendritic cell differentiation | 3/235 | 5.24 | 1.95E-02 | 1.82E-01 | BATF2/GAS6/CEBPB | 3 |
| BP | GO:0002824 | positive regulation of adaptive immune response based on somatic recombination of immune receptors built from immunoglobulin superfamily domains | 5/235 | 3.24 | 1.96E-02 | 1.82E-01 | CD274/JAK2/TBX21/GATA3/IL23A | 5 |
| BP | GO:0072676 | lymphocyte migration | 5/235 | 3.24 | 1.96E-02 | 1.82E-01 | CCR6/GAS6/TBX21/GATA3/ZAP70 | 5 |
| BP | GO:0050848 | regulation of calcium-mediated signaling | 4/235 | 3.87 | 2.00E-02 | 1.83E-01 | GBP1/LRRK2/CD22/ZAP70 | 4 |
| BP | GO:0061844 | antimicrobial humoral immune response mediated by antimicrobial peptide | 4/235 | 3.87 | 2.00E-02 | 1.83E-01 | S100A12/LTF/DEFA3/DEFA4 | 4 |
[truncated: 140,070 more chars]
